# Axial Ligand Effects on Electronic Structures and H‑Atom Abstraction by Oxidoiron(IV) Complexes Featuring Triplet-Only Reactivity

**DOI:** 10.1021/jacsau.6c00781

**Published:** 2026-09-14

**Authors:** Isabelle Becker, Massimiliano Morganti, Haowei Chen, Akhil Bhardwaj, Pilar Bologna, Guilherme Tripodi, Serhiy Demeshko, Sebastian Dechert, Jana Roithová, Frank Neese, Bhaskar Mondal, Shengfa Ye, Franc Meyer

**Affiliations:** † 9375 University of Göttingen, Institute of Inorganic Chemistry, Tammannstraße 4, 37077 Göttingen, Germany; ‡ Institute for Molecules and Materials, Radboud University, Heyendaalseweg 135, Nijmegen 6525, Netherlands; § School of Chemical Sciences, 231997 Indian Institute of Technology Mandi, Kamand, Mandi, Himachal Pradesh 175075, India; ∥ 28314 Max-Planck-Institut für Kohlenforschung, 45470 Mülheim an der Ruhr, Germany; ⊥ Key Laboratory of Bioinorganic and Synthetic Chemistry of Ministry of Education, Guangdong Basic Research Center of Excellence for Functional Molecular Engineering, School of Chemistry, IGCME, 26469 Sun Yat-Sen University, Guangzhou 510275, P.R. China; # University of Göttingen, International Center for Advanced Studies of Energy Conversion (ICASEC), Tammannstr. 6, 37077 Göttingen, Germany

**Keywords:** oxidoiron(IV) complexes, N-heterocyclic carbene ligands, electronic structure, hydrogen atom abstraction, axial ligand effects, triplet-only reactivity

## Abstract

Oxidoiron­(IV) species are crucial intermediates for the functionalization of C–H bonds by biological oxidases and oxygenases, and understanding the relations between ligation, electronic structure, and reactivity of the Fe^IV^=O unit is key to the rational design of bioinspired catalysts. Here, a series of Fe^IV^=O complexes based on a macrocyclic tetracarbene ligand and with different anionic *trans*-axial ligands (L^ax^ = CF_3_COO^–^, ^*t*^BuS^–^, AdaS^–^) has been comprehensively characterized, including X-ray crystallography in two cases, as well as zero-field and applied-field ^57^Fe Mössbauer spectroscopy, magnetic susceptibility measurements, magnetic circular dichroism (MCD), and helium-tagging infrared photodissociation (IRPD). This establishes a detailed picture of the electronic structures of these ferryl complexes, featuring a well-separated *S* = 1 ground state, and the influence of the *trans*-ligand. The confinement to triplet-only pathways in C–H bond activations and comparison with the parent Fe^IV^=O complex (L^ax^ = MeCN) allow uncovering trends in hydrogen atom abstraction (HAA) ability without perturbations from different extents of two-state reactivity (TSR). Kinetic studies on HAA for C–H substrates with BDEs up to 78 kcal mol^–1^ show relatively minor effects of the axial ligands, with rates following an electrophilic trend in the order L^ax^ = MeCN > CF_3_COO^–^ ≈ RS^–^. This contrasts the antielectrophilic trend found for a related series of tetramethylcyclam (TMC)-based Fe^IV^=O complexes for which TSR is dominant. For the present systems, temperature-dependent KIEs, in combination with computations at the DFT and ab initio levels of theory, suggest a semiclassical model with modest tunneling contributions that are most pronounced in the case of a strongly donating *trans*-thiolate. This study thus provides a combined experimental and computational basis for understanding intrinsic axial ligand effects and the effect of a *trans*-axial thiolate in *S* = 1 ferryl intermediates.

## Graphical abstract

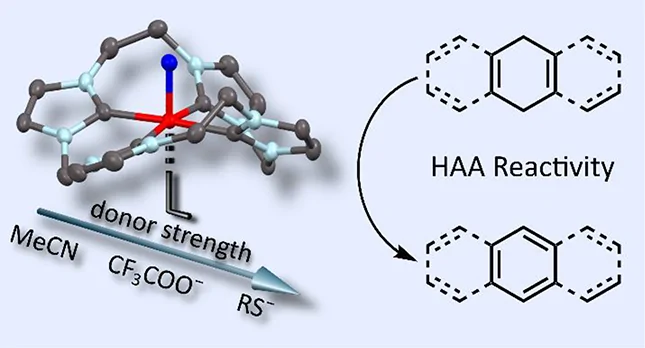

## Introduction

High-valent oxidoiron species are key intermediates in many transformations in nature, most prominently in the activation of O_2_ by heme and nonheme oxidases and oxygenases.^1^ These enzymes, such as Cytochrome P450, Taurine dioxygenase, halogenase CycC3, or phenylalanine hydroxylase, are known to catalyze challenging substrate transformations like the oxygenation of unactivated C–H bonds.^2^ In heme enzymes, the axial ligand coordinated to the central iron plays an important role for tuning the reactivity of the oxidoiron­(IV) (ferryl) intermediates in compounds I and II. Especially, a cysteinato ligand is proposed to enable the hydroxylation of strong C–H bonds by Cytochrome P450,^3^ because the axial thiolate ligand is assumed to increase the basicity of the Fe^IV^=O unit by a push effect allowing the hydrogen atom abstraction (HAA) to proceed despite relatively low redox potentials.^4,5^ Therefore, understanding the influence of different axial ligands on ferryl reactivity has been an essential aim, both for the biological systems and for synthetic analogues that emulate key features of the natural blueprints and may be employed for synthetically useful C–H bond functionalization.^6^

Most oxidoiron­(IV) nonheme model systems that have been investigated over the years feature chelating N-donor ligand scaffolds, most prominently macrocyclic ligands based on 1,4,8,11-tetraazacyclotetradecane (cyclam), pentadentate ligands with a central tertiary amine and four pyridine arms (N4Py) or tripodal tetradentate ligands like tris­(2-pyridylmethyl)­amine (TPA).^7^ These high-valent oxidoiron­(IV) complexes can engage in oxygen atom transfer (OAT), hydrogen atom abstraction (HAA), and electron transfer (ET) reactivity. In contrast to natural nonheme enzymes that have *S* = 2 spin states, most synthetic analogues exhibit an *S* = 1 ground state.^8^ However, the quintet state for nonheme enzymes leads to higher reactivity in C–H activation via the σ-pathway, where the σ*­(Fe-d_*z* ^2^_) is the electron-accepting orbital (EAO) interacting with the target C–H σ-bond and exchange energy is maximized.^9,10^ The steric strain and the activation barrier are hereby reduced in comparison to the π-pathway, where the π*_x/y_(Fe-d_*xz*,yz_) serves as EAO. *S* = 1 ferryl species are usually expected to react over the higher-barrier π-trajectory, but such model systems often exhibit an unexpectedly high reactivity. This has been explained by the two-state reactivity (TSR) scenario where, during the interaction with the C–H bond, the *S* = 1 complex undergoes a spin crossover to the more reactive quintet (*S* = 2) state, enhancing the overall reaction rate (Figure 1; left).^11,12^

**Figure 1:**
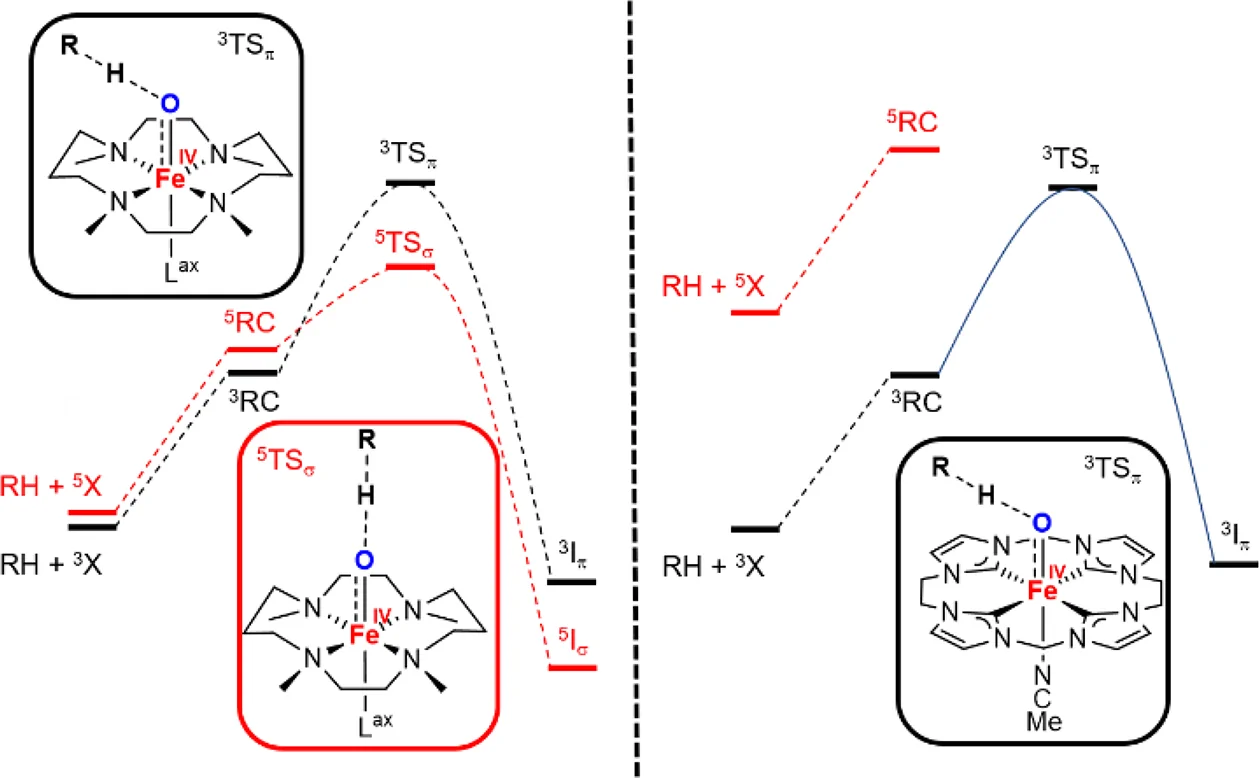
Schematic free energy profiles for HAA processes with R–H substrates for TMC-based *S* = 1 ferryl complexes featuring two-state reactivity (TSR) due to a small triplet-quintet gap (left) and for *S* = 1 ferryl complexes based on macrocyclic tetracarbenes with a very large triplet-quintet gap (right).

In principle, HAA by high-valent oxidoiron­(IV) intermediates is an electrophilic reaction, and hence strongly electron-donating supporting ligands that decrease the electrophilicity of the Fe^IV^=O unit would be expected to increase the transition state barrier.^11^ Ligands *trans* to the oxo group are more influential than the donors of the equatorial ligand scaffold, which are *cis* to the oxo group, because the *trans*-ligand directly interacts with both σ-type (Fe-d_*z* ^2^_) and π-type (Fe-d_*xz*,yz_) metal fragment orbitals. However, when Que, Shaik and Nam et al. experimentally studied the effect of different *trans*-ligands on the HAA reactivity for a series of *S* = 1 ferryl complexes bearing an equatorial tetramethylcyclam (TMC = 1,4,8,11-tetramethyl-1,4,8,11-tetraaza-cyclotetradecane), a counterintuitive antielectrophilic trend was observed.^13^ The reactivity of complexes [(TMC)­Fe^IV^O­(L^ax^)]^n+^ toward C–H substrates such 9,10-dihydroanthracene (DHA) increases in the order L^ax^ = NCCH_3_ (**A** ^**NCMe**^)^14^ < CF_3_COO^–^ (**A** ^**TFA**^)^15^ < N_3_^–^ (**A** ^**N3**^)^16^ < **A** ^**TMCS**^ (Figure 2, where TMCS represents a tethered *trans*-thiolate),^17^ which has been explained on the basis of the TSR model. Increasing electron donation by the axial ligand to the Fe^IV^=O group destabilizes the nb­(Fe-d_*xy*_) orbital relative to the σ*­(Fe-d_*x* ^2^–*y* ^2^_) orbital, which reduces the orbital energy gap and thus decreases the quintet-triplet separation Δ*E*_Q‑T_. This leads to enhanced participation of the more reactive S = 2 state in the HAA reaction, which overshadows the intrinsic axial ligand effect.^13,18^ Hence, **A** ^**TMCS**^ is the least electrophilic species but has the lowest Δ*E*_Q‑T_. In line with this reasoning, the reactivity of analogous ruthenium complexes [(TMC)­Ru^IV^O­(L^ax^)]^n+^ that all feature well-separated triplet (*S* = 1) ground states, was shown to follow the reverse trend, i.e., the expected electrophilicity pattern, although overall reactivity is rather sluggish.^19^ However, the antielectrophilic trend in HAA by ferryl complexes [(TMC)­Fe^IV^O­(L^ax^)]^n+^ may be significantly affected by the dependence of tunneling contributions on the electron-donating ability of the *trans*-axial ligand L^ax^.^20^ Therefore, a series of *S* = 1 ferryl complexes without involvement of two-state reactivity would be of particular interest to experimentally uncover the intrinsic effects of the axial ligands on Fe^IV^=O reactivity.

**Figure 2:**
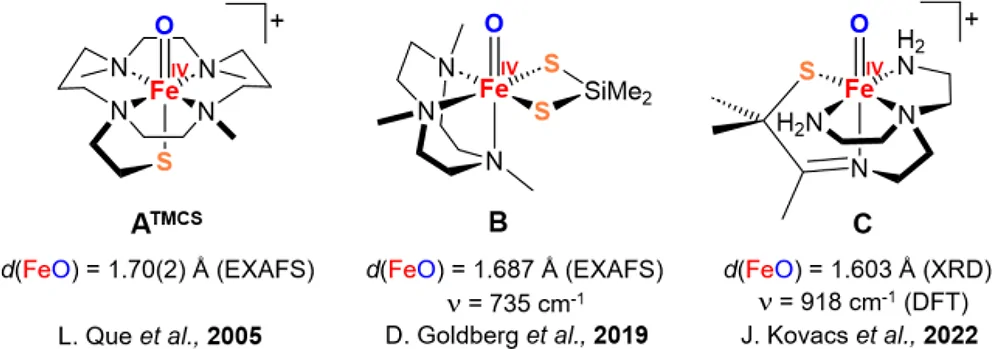
Previously reported oxidoiron­(IV) complexes featuring a thiolato ligand; only complex **C** has been characterized by X-ray diffraction.

In 2013, some of us reported the isolation of an organometallic oxidoiron­(IV) complex that features in the equatorial plane a macrocyclic ligand with four N-heterocyclic carbene donors, [L^NHC^Fe^IV^O­(MeCN)]­(OTf)_2_ (**3**; L^NHC^ = 3,9,14,20-tetraaza-1,6,12,17-tetraazoniapenta-cyclohex-acosane-1 (23),4,6(26),10,12(25),15,17(24),21-octaene; Figure 3).^21^ Complex **3** is topologically reminiscent of TMC-based **A** ^**NCMe**^, and it was shown that the bonding within the Fe^IV^=O unit is not significantly affected by the different equatorial scaffolds.^22^ Furthermore, a series of {Fe-NO}^*n*^ complexes of L^NHC^ demonstrated that redox changes are largely confined to the metal ion.^23^ However, the very strong σ-donation of the macrocyclic tetracarbene in oxidoiron­(IV) complex **3** raises the energy of its d_*x* ^2^–*y* ^2^_ orbital above the d_*z* ^2^_, which gives rise to a very large triplet-quintet energy gap, preventing accessibility of the quintet state (Figure 3; note that the lowest-energy quintet excited states of **A** ^**NCMe**^ and **3** have different electron configurations, nb­(Fe-d_*xy*_)^1^π*­(Fe-d_*xz*,yz_)^2^σ*­(Fe-d_*x* ^2^–*y* ^2^_)^1^σ*­(Fe-d_*z* ^2^_)^0^ versus a unique nb­(Fe-d_*xy*_)^1^π*­(Fe-d_*xz*,yz_)^2^σ*­(Fe-d_*z* ^2^_)^1^σ*­(Fe-d_*x* ^2^–*y* ^2^_)^0^ in the case of **3**).^22^ As a consequence, the HAA reactivity of **3** is confined to the triplet surface, and any two-state reactivity is quenched (Figure 1, right). While representing the intrinsic reactivity of a ferryl species in its triplet state, **3** was shown to exhibit HAA reactivity toward C–H substrates DHA or 1,4-cyclohexadiene (CHD) that are 2–3 orders of magnitude higher than for **A** ^**NCMe**^ under the same experimental conditions.^24^ L^NHC^-based oxidoiron­(IV) complexes therefore represent an ideal platform for the systematic investigation of intrinsic axial ligand effects on *S* = 1 ferryl reactivity without the ambiguities arising from different extents of two-state reactivity. A recent computational study indeed predicted that different axial ligands barely alter the triplet state orbital ordering of [(L^NHC^)­Fe^IV^O­(L^ax^)]^n+^ complexes and do not significantly influence the reactivity.^25^

**Figure 3:**
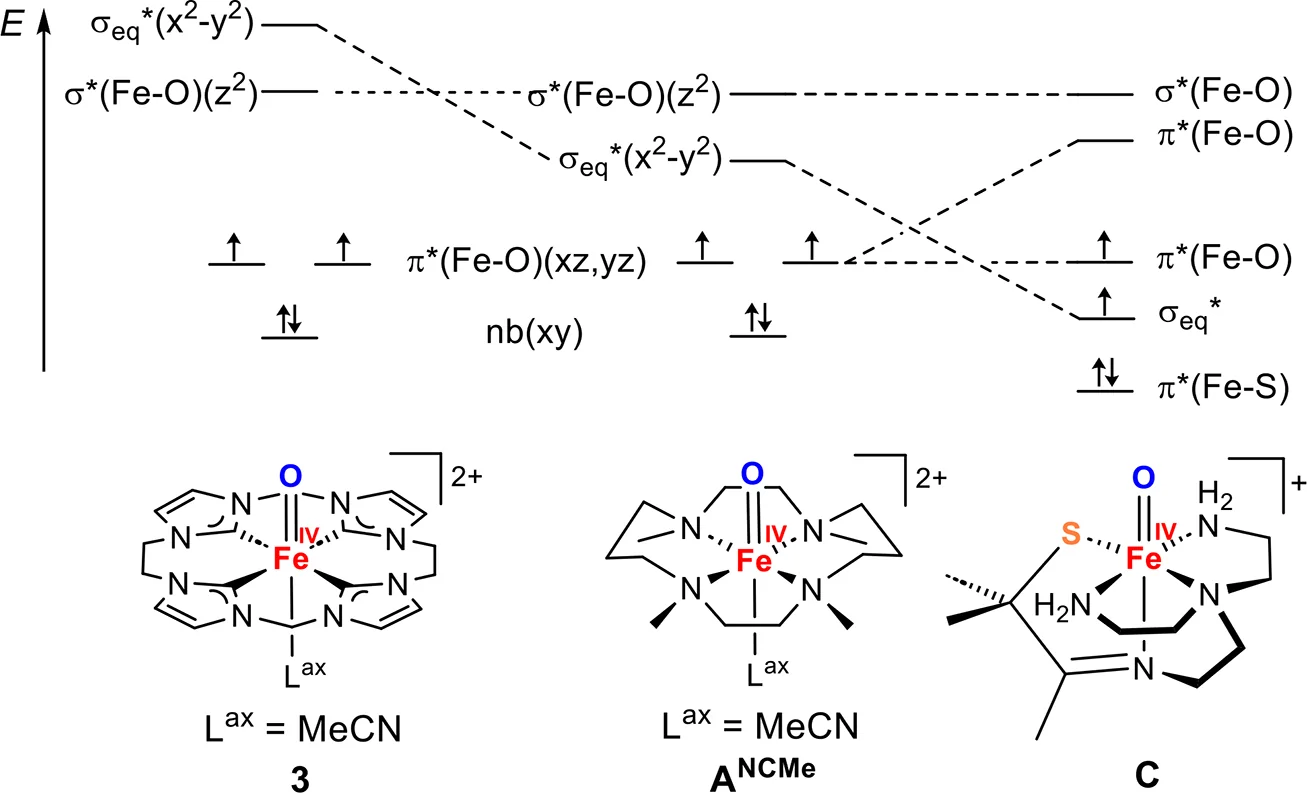
Structures and electronic ground states of **3**, **A^NCMe^**, and **C**.

In the present work, we now synthesize several new oxidoiron­(IV) complexes based on the tetracarbene scaffold L^NHC^, namely [L^NHC^Fe^IV^O­(L^ax^)]­(OTf) with varying anionic *trans*-ligands L^ax^ = CF_3_COO^–^, ^*t*^BuS^–^ and AdaS^–^ (Ada is 1-adamantyl); these L^ax^ were chosen to enable a direct comparison with the related TMC-based systems. Since oxidoiron­(IV) intermediates with S-bound cysteine are the key oxidants in the catalytic cycles of many heme and nonheme iron enzymes, a focus of this study lies on species [L^NHC^Fe^IV^O­(SR)]^+^ with a *trans*-thiolate. During the last two decades, extensive efforts have been devoted to the generation and isolation of synthetic nonheme oxidoiron­(IV) complexes with thiolato ligation, aiming at understanding how thiolates provide favorable conditions for O_2_ binding and reactivity enhancement. However, the number of well-characterized nonheme oxidoiron­(IV) complexes with a thiolato ligand has remained very limited, mainly because the stabilization of a thiolato group in a highly oxidizing environment in the absence of a protecting protein pocket is challenging; previously reported examples are shown in Figure 2.

A crystallographically characterized *cis*-thiolato-ligated high-valent oxidoiron­(IV) complex **C** with an extremely short Fe–O bond (1.603(7) Å) was reported by Kovacs et al. and was shown to be capable of cleaving strong C–H bonds (96 kcal mol^–1^).^26^ Experimental spectroscopic data for **C** are lacking, but its properties were rationalized by DFT calculations, which indicated that **C** is an *S* = 1 Fe^IV^=O species with the σ*­(Fe–O) and one of the π*­(Fe–O) orbitals being empty, while the lower-lying π*­(Fe–S) orbital is doubly occupied and the σ*­(Fe–S) orbital is singly occupied (Figure 3), leading to a long Fe–S bond and a very short Fe–O bond as well as an unusually high DFT-calculated ν_Fe–O_ stretching frequency of 918 cm^–1^. This situation clearly differs from the situation in the only nonheme Fe^IV^=O model complex featuring a *trans*-thiolato ligand, [(TMCS)­FeO]^+^ (**A** ^**TMCS**^) reported by Que and coworkers.^17^ The structure of complex **A** ^**TMCS**^, which is based on a cyclam derivative with a pendant thiolato arm (TMCS), has been determined by extended X-ray absorption fine structure (EXAFS), showing *d*(Fe–O) = 1.70(2) Å and *d*(Fe–S) = 2.33(2) Å. Unfortunately, no experimental vibrational data for ν_Fe–O_ of **A** ^**TMCS**^ are available. Complex **A** ^**TMCS**^ is the current benchmark system that has been used to investigate the effect of axial thiolato ligation on Fe^IV^=O reactivity.^27^ Oxidoiron­(IV) complex **B** with a silanedithiolato ligand *cis* to the Fe=O unit has also been characterized by EXAFS and shows *d*(Fe–O) = 1.687(6) Å.^28^ The Fe–O stretch was identified by resonance Raman (rR) spectroscopy at ν_Fe–O_ = 735 cm^–1^. However, the authors indicated that some Fe^III^(O)­(thiyl radical) character for **B** cannot be ruled out. **B** was found to be unreactive toward weak C–H bonds in substrates such as xanthene or acridine.^28^ In view of the very limited number of reported thiolato-ligated oxidoiron­(IV) species, the isolation of further Fe^IV^=O complexes with a *trans*-thiolate and their structural characterization by single-crystal X-ray diffraction (scXRD) as well as studies on their HAA reactivity would be highly desirable.

Here, the new complexes [L^NHC^Fe^IV^O­(L^ax^)]­(OTf) are comprehensively characterized by different spectroscopic methods including applied-field ^57^Fe Mössbauer as well as MCD spectroscopy, and some of them are also structurally characterized by single-crystal X-ray diffraction (scXRD). Furthermore, the HAA reactivity toward a set of C–H substrates has been studied, and temperature-dependent kinetic isotope effects (KIEs) are examined, and the results are evaluated and rationalized computationally. Together with **3**, this series of new [L^NHC^Fe^IV^O­(L^ax^)]­(OTf) complexes thus allows us to experimentally assess the intrinsic axial ligand effect for pure “triplet-only” ferryl model systems.

## Results and Discussion

### Synthesis and Structural Characterization of Fe^IV^=O Complexes

[L^NHC^Fe^IV^O­(NCMe)]­(OTf)_2_ (**3**) was synthesized by the addition of 2.8 eq of the soluble iodosobenzene derivative 2-(^*t*^BuSO_2_)­C_6_H_4_IO (sPhIO) to the Fe^II^ complex [L^NHC^Fe­(NCMe)_2_]­(OTf)_2_ (**1**) at −35 °C in MeCN, as reported previously.^21,24^ The new complex [L^NHC^Fe^IV^O­(OOCCF_3_)]­OTf (**4**) with an exogeneous trifluoroacetate (TFA) was now obtained by adding 1 eq of NaOOCCF_3_ to the reaction mixture of freshly prepared **3** (Scheme 1). For installing a *trans*-axial thiolate, the thiolato ligands have to be bound prior to the formation of the oxidizing ferryl species to avoid the oxidation of free thiolate. Sterically demanding ^*t*^BuS^–^ and AdaS^–^ were used in this work in order to disfavor intermolecular degradation pathways involving disulfide bond formation. Hence, Fe^II^ complexes [L^NHC^Fe­(SR)­(NCMe)]­OTf (**2a**: R = ^*t*^Bu; **2b**: R = Ada) were prepared according to recently reported procedures^29^ by adding the corresponding NaSR to a solution of **1** in MeCN, and the resulting thiolato complexes **2a**/**2b** were subsequently oxygenated by the addition of 2.8 eq of sPhIO at −35 °C to give complexes [L^NHC^Fe^IV^O­(SR)]­OTf (**5a**, **5b**; Scheme 1). Alternatively, NBu_4_[IO_4_] can also be used as an oxidant.

**Scheme 1:**
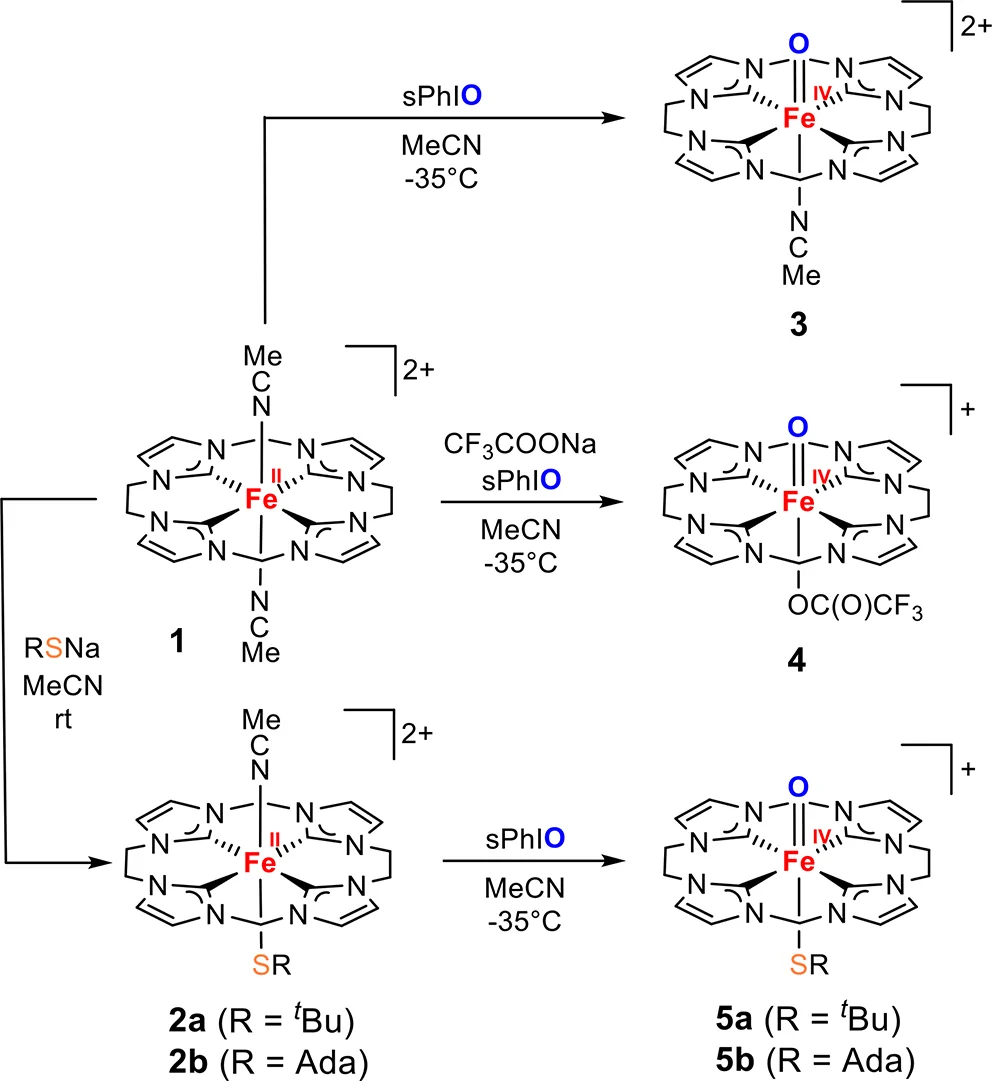
Complexes Studied in This Work and Their Syntheses

Oxidation of the Fe^II^ complexes and generation of the oxoferryl species can be conveniently monitored by UV–vis spectroscopy (Figure 4 and Figure S1). The parent complex [L^NHC^Fe^IV^O­(NCMe)]­(OTf)_2_ (**3**) shows characteristic absorption bands at 400 nm and 600 nm.^21,24,30^ In the case of [L^NHC^Fe^IV^O­(OOCCF_3_)]­OTf (**4**), these bands are slightly red-shifted to 408 and 629 nm, respectively (the assignment of these absorptions is discussed below). Both thiolato complexes [L^NHC^Fe^IV^O­(S^*t*^Bu)]­OTf (**5a**) and [L^NHC^Fe^IV^O­(SAda)]­OTf (**5b**) show almost identical UV–vis spectra with absorption bands at 442 and 577 nm (**5a**) or 447 and 586 nm (**5b**). In contrast to complexes **3** and **4**, however, **5a**/**5b** feature an additional weaker absorption around 710 nm, which will be discussed in more detail below. In case of thiolato-ligated Fe^II^ complexes **2a**/**2b** and using NBu_4_[IO_4_] as an oxidant for UV–vis monitoring (see SI for details), a transient intermediate with an absorption around 800 nm can be observed en route to **5a**/**5b** (Figure 4, bottom). This intermediate could not be isolated or characterized so far, but one may speculate that it is an initial IO_4_^–^ adduct of **2a**/**2b**.

**Figure 4:**
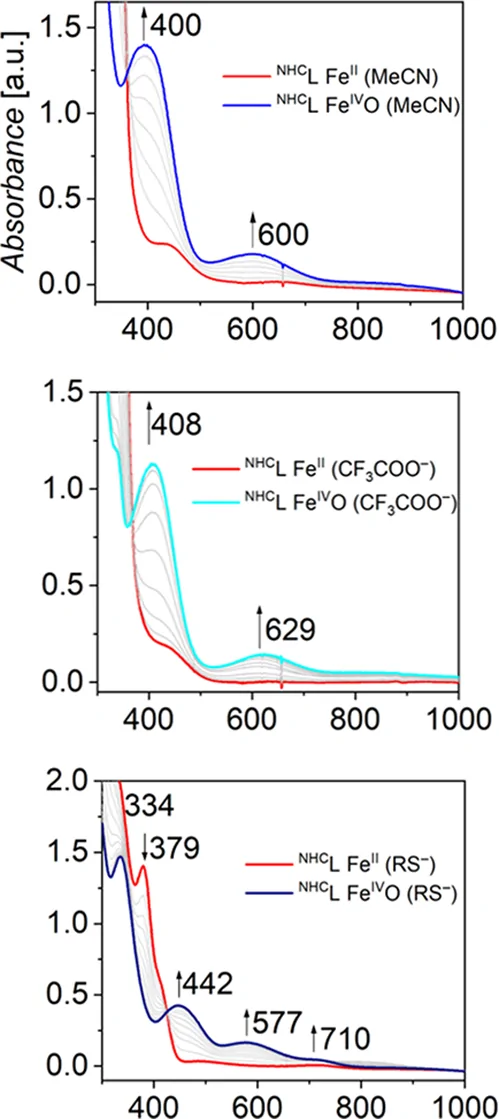
*In situ* formation of complexes **3** (1.0 mM; top), **4** (1.0 mM; middle), and **5a** (0.25 mM; bottom) at −40 °C upon the addition of excess oxidant (sPhIO or NBu_4_[IO_4_]).

Single crystals of **4** could be obtained by the slow diffusion of diethyl ether into a MeCN solution of the complex at −35 °C and were analyzed by X-ray diffraction. The molecular structure of the cation is shown in Figure 5 (left) and confirms the presence of the O-bound TFA *trans* to the oxido ligand. The Fe=O bond length is 1.671(1) Å, which is only slightly longer than in the parent complex 3* (1.661(3) Å; 3* is the derivative of **3** with *trans*-axial EtCN, which was characterized by scXRD).^21^ The tetra­(NHC) macrocycle L^NHC^ adopts a saddle-shaped nonplanar structure, and each −CH_2_CH_2_– linker forms one short C–H···O contact with the ferryl oxygen atom (dashed lines in Figure 5), indicative of a weak interaction that was also observed for the parent complex 3* and was computationally suggested to contribute to the relative stability of **3**/3*,^21,25^ similar to the stabilization of a tripodal oxidoiron­(IV) complex with three N–H···O hydrogen-bonding interactions.^31^

**Figure 5:**
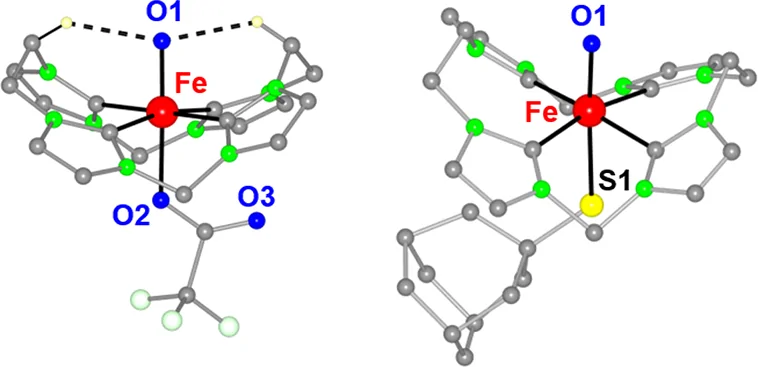
Plot (30% probability thermal ellipsoids) of the molecular structure of the cationic parts of **4** (left) and **5b** (right); hydrogen atoms omitted for clarity.

Dark green single crystals of **5b** could be grown by layering diethyl ether onto a solution of the complex in PrCN at −35 °C. X-ray diffraction analysis confirms the molecular structure with equatorial ligation of the central iron atom by the L^NHC^ macrocycle, a terminal oxido ligand and a *trans*-axial thiolate (Figure 5, right). However, the quality of the crystallographic data is rather poor, so we refrain from a detailed discussion of metric parameters.

The new complex **5b** is only the second isolated nonheme oxidoiron­(IV) species with a *trans*-thiolate, and the first to be structurally authenticated by scXRD, with **A** ^**TMCS**^ (Figure 2) reported by Que, Nam, and coworkers being the benchmark system so far.^17^ While structural parameters of **A** ^**TMCS**^ such as *d*(Fe–O) = 1.70(2) Å and *d*(Fe–S) = 2.33(2) Å were determined by EXAFS, vibrational data for ν_Fe–O_ of **A** ^**TMCS**^ are unknown. On the other hand, the electronic structure of **C** (Figure 2) featuring a *cis*-thiolato-ligated *S* = 1 Fe^IV^=O unit is clearly very different from that of Fe^IV^=O complexes with a *trans*-thiolate (vide supra). To identify the effect of the *trans* axial thiolate on the tetra­(NHC)-ligated Fe^IV^=O core and to provide a foundation for evaluating reactivity trends, the spectroscopic properties and electronic structures of the new complexes **4** and **5a**/**5b** have been studied in detail in this work and are compared with those of parent **3**.

### Spectroscopic Characterization and Electronic Structures of Fe^IV^=O Complexes

^57^Fe Mössbauer spectroscopy of the newly synthesized oxidoiron complexes **4**, **5a**, and **5b** confirmed the iron­(IV) character. **4** features a very low isomer shift of δ = −0.14 mm s^–1^ and a large quadrupole splitting of Δ*E*_Q_ = 3.13 mm s^–1^ (Figure 6, left). These values are very similar to those of the parent oxidoiron­(IV) compound [L^NHC^FeO­(NCMe)]­(OTf)_2_ (**3**: δ = −0.13 mm s^–1^, Δ*E*_Q_ = 3.08 mm s^–1^),^21^ which have previously been attributed to the extremely strong equatorial σ-donor strength of the macrocyclic tetra­(NHC) ligand with pronounced charge donation into the iron Fe­(4s) orbital as well as to the oblate charge distribution around the Fe nucleus arising from the strongly asymmetric covalent bonds and the large electronic charge donated by the equatorial NHCs into the d_*x* ^2^‑*y* ^2^_ orbital. In contrast, oxidoiron­(IV) complexes based on the TMC ligand scaffold show isomer shifts within the narrow range δ = 0.15–0.20 mm s^–1^ and much smaller quadrupole splittings in the range Δ*E*_Q_ = 0.16–1.39 mm s^–1^.^32^ Complexes **5a** and **5b** show less negative isomer shifts δ = −0.09 mm s^–1^ and significantly smaller Δ*E*_Q_ = 2.21 mm s^–1^ or Δ*E*_Q_ = 2.23 mm s^–1^, respectively (Figure 6, right, and Figure S3). This can be attributed to the electronic nature of the axial thiolato ligand, which acts both as σ- and strong π-donor. Due to the latter interaction, the electron density in the d-orbitals of Fe increases, causing a higher shielding of the s-electron density and a lowering of the anisotropy of charge distribution around the iron core; this increases the isomer shift and lowers the quadrupole splitting. A similar effect on Δ*E*_Q_ was found for TMC-based oxidoiron­(IV) complexes, where **A** ^**NCMe**^ and **A** ^**TFA**^ (the latter having a *trans*-axial CF_3_COO^–^) show similar quadrupole splittings of 1.23 mm s^–1^ and 1.39 mm s^–1^, respectively, while **A** ^**TMCS**^ has a much smaller Δ*E*_Q_ = 0.22 mm s^–1^ that reflects the larger basicity of the axial thiolato ligand.^17,32^

**Figure 6:**
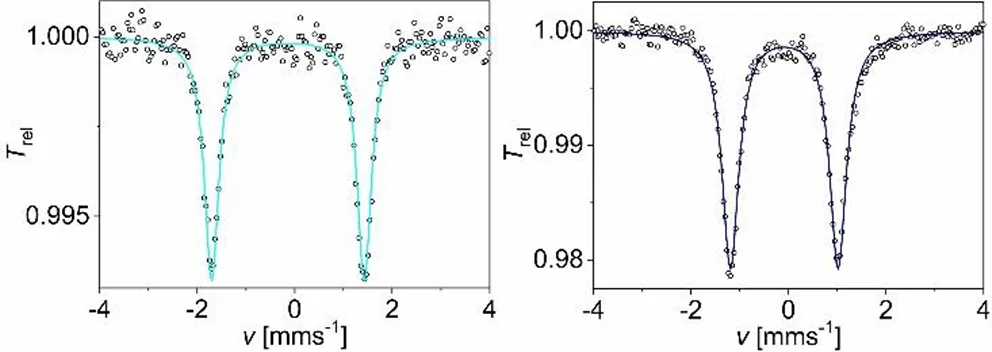
Zero-field ^57^Fe Mössbauer spectra of solid samples of [L^NHC^Fe^IV^O­(OOCCF_3_)]­OTf (**4**) and [L^NHC^Fe^IV^O­(S^*t*^Bu)]­OTf (**5a**) at 80 K.

Magnetic susceptibility and variable temperature–variable field (VTVH) data for solid samples of **4**, **5a**, and **5b**, obtained by SQUID measurements, are shown in Figure 7 and Figure S4. They confirm an *S* = 1 ground state for all three compounds and rather small zero-field splitting parameters of *D* = 19.0 cm^–1^ (for **4**), 14.5 cm^–1^ (for **5a**) and 15.7 cm^–1^ (for **5b**). These *D* values are similar to that of parent **3** (*D* = 16.4 cm^–1^)^21^ but much smaller than for TMC-based systems, which exhibit values around *D* = 30 cm^–1^ (**A** ^**NCMe**^: *D* = 29 cm^–1^, **A** ^**TFA**^: *D* = 31 cm^–1^ and **A** ^**TMCS**^: *D* = 35 cm^–1^).^14,17,32^ This reflects a large triplet-quintet gap in the tetra­(NHC)-ligated oxidoiron­(IV) species, where the mixing of the *S* = 1 and *S* = 2 states is minimal. Theoretical results are consistent with a large triplet-quintet gap for the new complexes **4**, **5a**, and **5b**, ∼15–20 kcal mol^−1^ at the DFT-B3LYP level and ∼25–30 kcal mol^−1^ at the CASSCF­(12,12)/NEVPT2 level (Table S11).

**Figure 7:**
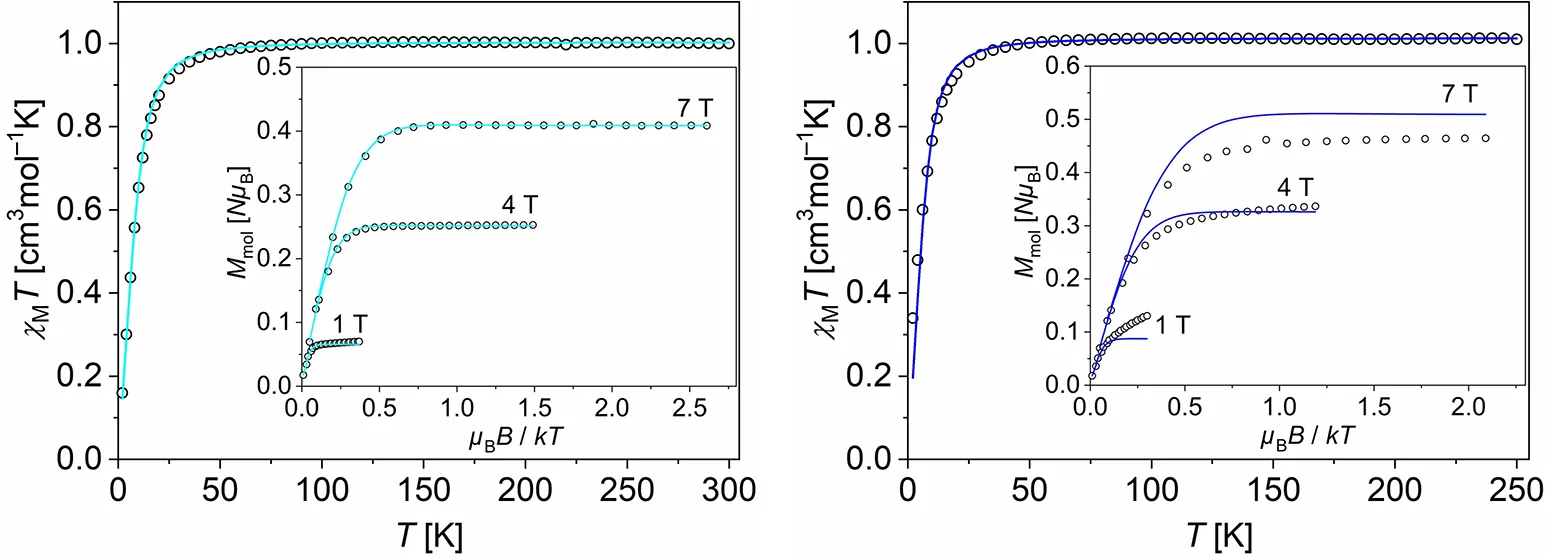
Magnetic susceptibility and variable temperature–variable field (VTVH) measurements of solid samples of [L^NHC^Fe^IV^O­(OOCCF_3_)]­OTf (**4**) (left) and [L^NHC^Fe^IV^(O)­(S*^t^* Bu)]­OTf (**5a**) (right).

The above zero-field splitting parameter values *D* were confirmed by applied magnetic field Mössbauer measurements on **4** and **5b**, as shown in Figure 8. Simulation of the spectra (see SI for details) gave *D* = 17.6 cm^–1^ for **4** and *D* = 18.4 cm^–1^ for **5b**, in good agreement with the values derived from the SQUID data. The magnetic hyperfine coupling constants *A*/*g*_N_μ_N_ [xx, yy, zz] for **4** [−20.6, −24.6, −2.8 T] and for **5b** [−24.9, −22.2, 3.2 T] are quite similar. The weak rhombicity is evident from the small difference between *A*_*xx*_ and *A*_*yy*_, whereas the tensor remains predominantly axial. The values obtained for **4** and **5b** are in good agreement with those reported for **3** [−17.1, −18.8, −2.0 T] or **A** ^**TMCS**^ [−23, −25, −5 T], except for the sign of *A*_*zz*_.^16,20^ The sign change may reflect the pronounced sensitivity of the z component of the *A* tensor to the axial ligand field, where even subtle changes in axial bonding can modify the balance between the isotropic Fermi-contact term and anisotropic spin-dipolar and spin–orbit contributions along the L^ax^–Fe=O axis, thereby affecting both the magnitude and sign of *A*_*zz*_.

**Figure 8:**
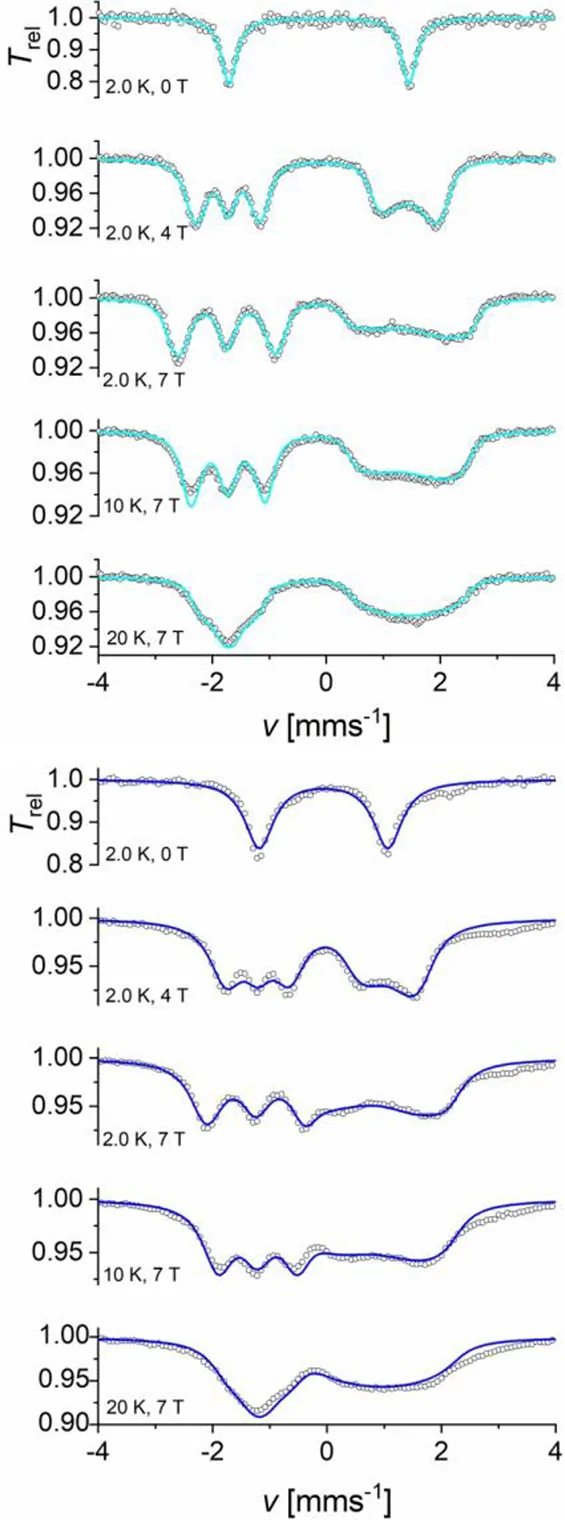
Field-dependent ^57^Fe Mössbauer measurements of solid samples of [L^NHC^Fe^IV^O­(OOCCF_3_)]­OTf (**4**; top) and [L^NHC^Fe^IV^O­(SAda)]­OTf (**5b**; bottom). For the fitting, *g*-values were fixed to 2.0 to avoid overparameterization. Note that insertion of the sample into the spectrometer at room temperature led to a small impurity of degradation product in the case of complex **5b**, which, however, could be easily distinguished during the fitting of the spectrum; it was not possible to record spectra of **5a** due to its higher instability at rt.

The Fe=O stretching vibration of the new complexes **4** and **5a**/**5b** was identified by helium-tagging infrared photodissociation (IRPD) measurements in combination with ^18^O labeling,^22,33^ where the Fe=^18^O species were synthesized by using ^18^O-labeled sPhIO (for **5a**/**5b**) or by the addition of H_2_^18^O into the cold MeCN solution of the complexes (for **3** and **4**). This method gives gas-phase ν̃_Fe–O_ values for the cations [L^NHC^FeO­(X)]^+^ (X^–^ = CF_3_COO^–^, ^*t*^BuS^–^, AdaS^–^) unperturbed by solvation. In solution, the oxygen atom of the Fe=O bond interacts with solvent molecules, leading to slightly more polarized Fe=O bonds. On average, in acetonitrile, the gas-phase Fe=O stretching frequency blue-shifts by +9 cm^–1^.^33^ Hence, the effect is small and consistent across similar complexes in a given solvent. The same method was previously used to determine ν̃_Fe–O_ = 832 cm^–1^ (Δ­(^18^O–^16^O) = −33 cm^–1^) for the parent complex **3** with a *trans*-axial MeCN (Figure 9A),^22^ which is comparable to the Fe=O stretching vibration of TMC-based **A** ^**NCMe**^ (ν̃_Fe–O_ = 839 cm^–1^, Δ­(^18^O–^16^O) = −35 cm^–1^).^32^

**Figure 9:**
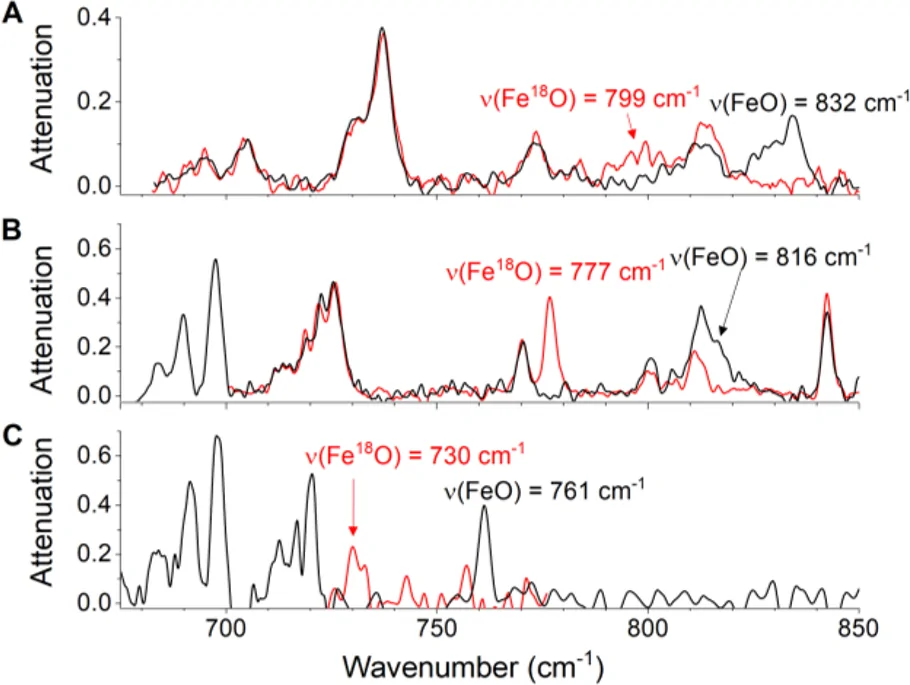
Infrared photodissociation (IRPD) spectra for complexes A) **3**,^22^ B) **4**, and C) **5a** (black) and their respective Fe=^18^O isotopologues (red).

In case of **4**, the Fe=O stretch appears at slightly lower energy at ν̃_Fe–O_ = 816 cm^–1^ (Δ­(^18^O–^16^O) = −39 cm^–1^; Figure 9B), which is in line with the slightly elongated Fe=O bond length determined by scXRD (1.671(1) Å for **4** vs 1.661(3) Å for 3*); the difference in ν̃_Fe–O_ indeed translates into Δ­[*d*(Fe–O)] of ∼0.01 Å according to a Badger’s rule analysis.^34,35^ The slight weakening of the Fe=O bond in **4** can be explained by the weak π-donor CF_3_COO^–^ in the *trans*-axial position competing with the oxido moiety, in contrast to the weak π-acceptor MeCN in **3** (or EtCN in 3*). However, the Fe=O stretch for **A** ^**TFA**^ (854 cm^–1^, Δ­(^18^O–^16^O) = −37 cm^–1^)^32^ occurs at higher energy than for **A** ^**NCMe**^ (839 cm^–1^). This difference is likely due to solvation of the CF_3_COO^–^ anion, which reduces its π-donor ability, an effect that is absent in the IRPD measurements (note that IRPD affords ν̃_Fe–O_ for the bare cation in the gas phase, while the Raman spectra for **A** ^**NCMe**^ and **A** ^**TFA**^ were recorded in MeCN solution).

In the present work it was possible for the first time to determine the Fe=O stretching frequency for a synthetic nonheme oxidoiron­(IV) complex with *trans*-axial thiolate: a very low ν̃_Fe–O_ = 761 cm^–1^ is observed for **5a**, shifting to 730 cm^–1^ upon ^18^O-labeling (Δ­(^18^O–^16^O) = −31 cm^–1^; Figure 9C). A similar value ν̃_Fe–O_ = 753 cm^–1^ was obtained for complex [L^NHC^FeO­(SAda)]^+^ (**5b**; see Figure S5). According to a normalized Badger’s rule analysis,^34,35^ this would correspond to a long Fe=O bond of *d*(Fe–O) ≈ 1.69/1.70 Å in **5a/5b**, very similar to *d*(Fe–O) = 1.70(2) determined by EXAFS for **A** ^**TMCS**^. Also, DFT calculations give a long Fe=O bond for **5a** (1.672 Å) compared to **3** and **4** (1.645 and 1.644 Å, respectively; see Table S12). These values indicate substantial weakening of the Fe=O bond in **5a**/**5b**, which has been assumed to translate into higher reactivity of oxidoiron­(IV) intermediates with *trans*-axial thiolate toward hydrogen atom abstraction (HAA). The results also underline the very different electronic structures for **5a**/**5b** and oxidoiron­(IV) complex **C** with a *cis*-thiolato ligand,^26^ the latter showing an extremely short Fe–O bond (vide supra). All the important experimental parameters are summarized in Table 1.

**Table 1:** Experimental Structural and Spectroscopic Parameters for All Complexes [L^NHC^FeO­(L)]­(OTf)_2_ (L = MeCN/EtCN) and [L^NHC^FeO­(X)]­OTf (X = TFA, S*^t^* Bu, SAda)

complex	_λmax_ [nm]	*d*(Fe–O) [Å]	δ [mm s^–1^]	Δ*E*_Q_ [mm s^–1^]	*D* [cm^–1^] (SQUID)	*D* [cm^–1^] (MB)	ν̃_Fe−O_ [cm^–1^]
**3**/3* ^21,22^	400, 610	1.661	–0.13	3.08	16.4	15	832
**4**	408, 629	1.671	–0.14	3.13	19.0	17.6	816
**5a**	442, 577	-	–0.09	2.21	14.5	-	761
**5b**	447, 586	-	–0.09	2.23	15.7	18.4	753

### Magnetic Circular Dichroism (MCD) and UV−vis Studies

Magnetic Circular Dichroism Spectroscopy (MCD) is a powerful technique to investigate the electronic structures of the ground state as well as the excited states, especially for transition metal complexes. Earlier detailed investigations^22^ showed that the MCD spectra of complex **3** are dominated by three ligand field transitions that were attributed to promoting an electron from Fe-3d_*xy*_ to π*_x/y_ (Fe-d_*xz*,*yz*_), from π*_x/y_ to Fe-3d_*x* ^2^‑*y* ^2^_, and from π*_x/y_ to Fe-3d_z^2^_, respectively. The first excitation is situated at ∼12000 cm^–1^ in the near-infrared region and is distinguished by a characteristic vibronic progression along the Fe–O stretch of the associated excited state. The MCD signals of the transitions π*_x/y_→Fe-3d_x^2^‑y^2^_ and π*_x/y_→Fe-3d_z^2^_ at 20 K are opposite in sign; the former one gives a positive pseudo-*A* feature at ∼24000 cm^–1^ and the latter a negative one at ∼17000 cm^–1^. On the basis of these findings, the MCD spectra of the new complexes [L^NHC^FeO­(X)]­OTf with X^–^ = CF_3_COO^–^ (**4**) or ^*t*^BuS^–^ (**5a**) can be unequivocally assigned. As depicted in Figures S7–S10, the relative energies of the three transitions all follow the same ordering. Therefore, all L^NHC^-ligated oxidoiron­(IV) complexes **3**, **4,** and **5a** feature comparable electronic structures in the sense that the Fe-3d_x^2^‑y^2^_ orbital lies energetically above Fe-3d_z^2^_ due to the very strong σ-donating ability of the equatorial L^NHC^ ligand.

MCD spectra typically exhibit higher resolution compared to UV–vis spectra and allow for a more reliable determination of the energy-level variation due to the ligand variance (shown in Figure 10). Compared with parent complex **3** (MeCN), the excitation energy for π*_x/y_→Fe-3d_x^2^‑y^2^_ in complex **4** (CF_3_COO^–^) is slightly lower, while this transition is significantly lower in energy by about 2000 cm^–1^ for complex **5a** (^*t*^BuS^–^). This is reflected in the UV–vis absorptions at 400, 408, and 442 nm for **3**, **4** and **5a**, respectively (cf. Figure 4). Since Fe-3d_x^2^–y^2^_ is a σ-antibonding orbital in the equatorial plane, its energy is hardly affected by the variation of the axial ligand. Therefore, this ordering discloses the varying destabilization degree of π*_x/y_ resulting from the differential π-donating ability of the axial ligand, congruent with the well-accepted sequence MeCN < CF_3_COO^–^ < ^*t*^BuS^–^. However, the transition energy for π*_x/y_→Fe-3d_z^2^_ follows a different order, namely ^*t*^BuS^–^ (**5a**) > MeCN (**3**) > CF_3_COO^–^ (**4**) (cf. UV–vis absorptions at 577, 610, and 629 nm). This reflects the combined effect of σ- and π-donating capability of the axial ligand, which ought to destabilize Fe-3d_z2_ and π*_x/y_, respectively, yet to a varying extent. CASSCF/NEVPT2 calculations nicely reproduced the observed trend of the transition energy variations (Table S10), although the energy differences are not significant, presumably due to the weak binding of the axial ligand.

**Figure 10:**
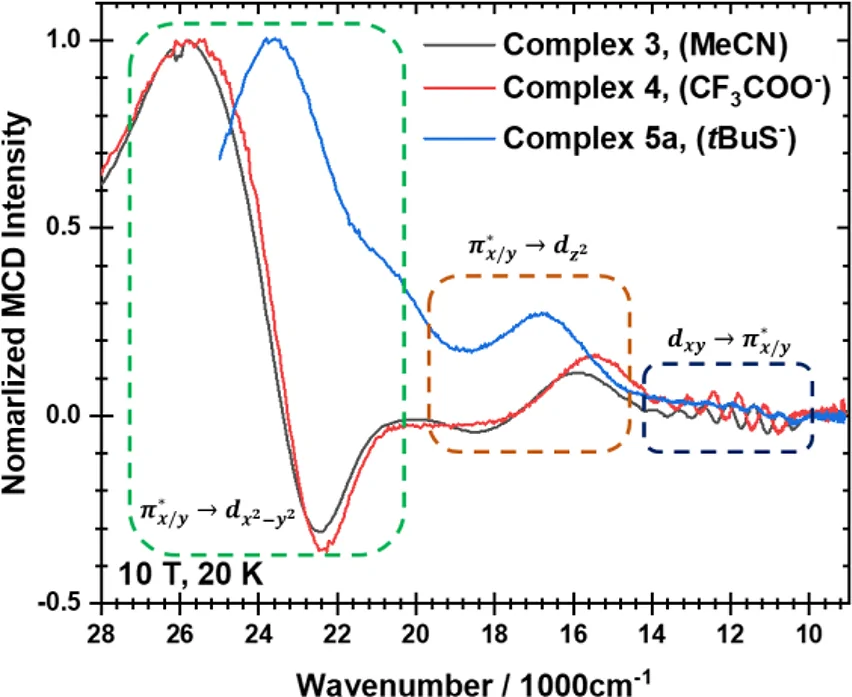
Overlay of the 20 K MCD spectra of complexes 3 (black), 4 (red), and 5a (blue).

We performed a Franck–Condon analysis of the vibronic progression observed for the transition Fe-3d_*xy*_→π*_x/y_ for **4** and **5a**, following the same procedure previously employed (data for **3** are included in Figure 10 for comparison).^22,36^ The average spacing of vibronic peaks represents the excited-state Fe–O stretching frequency. Moreover, the intensity of the *n* ^th^ progression lines follows a Poisson distribution,

$$\frac{I_{0-n}}{I_{0-0}}=\frac{{S_{HR}}^{n}}{n!}$$

in which *I*_0–0_ and *I*_0–*n*_ are the intensities of the 0–0 and 0–n vibronic transitions, while *S_HR_* denotes the Huang–Rhys factor that describes the displacement of the excited state potential energy surface relative to that of the ground state,

$$S_{HR}=\frac{\frac{1}{2}k_{es}\Delta r^{2}}{hv_{es}}$$

in which *k*_es_ is the force constant of the excited-state Fe–O stretching mode, *hν*_es_ is the corresponding vibrational energy, and Δ*r* is the elongation fraction of the Fe–O distance.

Since the intensity of this transition first increases and then decreases upon elevating the temperature, the spectra recorded at 20 K and under a 10 T magnetic field were chosen for vibronic analyses, and the best simulations are shown in Figure 11 and Figures S11–S13. Because *hν*_es_, *S*_HR_, and the line width strongly correlate with each other, especially when the spectral resolution is not sufficiently high, the error bar of the simulation is somewhat underestimated. Despite this difficulty, the excited-state Fe–O stretching frequencies obtained from the simulations (Figure 11) are all substantially lower than those determined for the ground state, because this Fe-3d_*xy*_→π*_x/y_ excitation weakens the Fe–O π bond. The vibrational energy of this series follows the order ^*t*^BuS^–^ (**5a**) < MeCN (**3**) ≈ CF_3_COO^–^ (**4**), consistent with the relative ν_Fe–O_ measured for their ground states.

**Figure 11:**
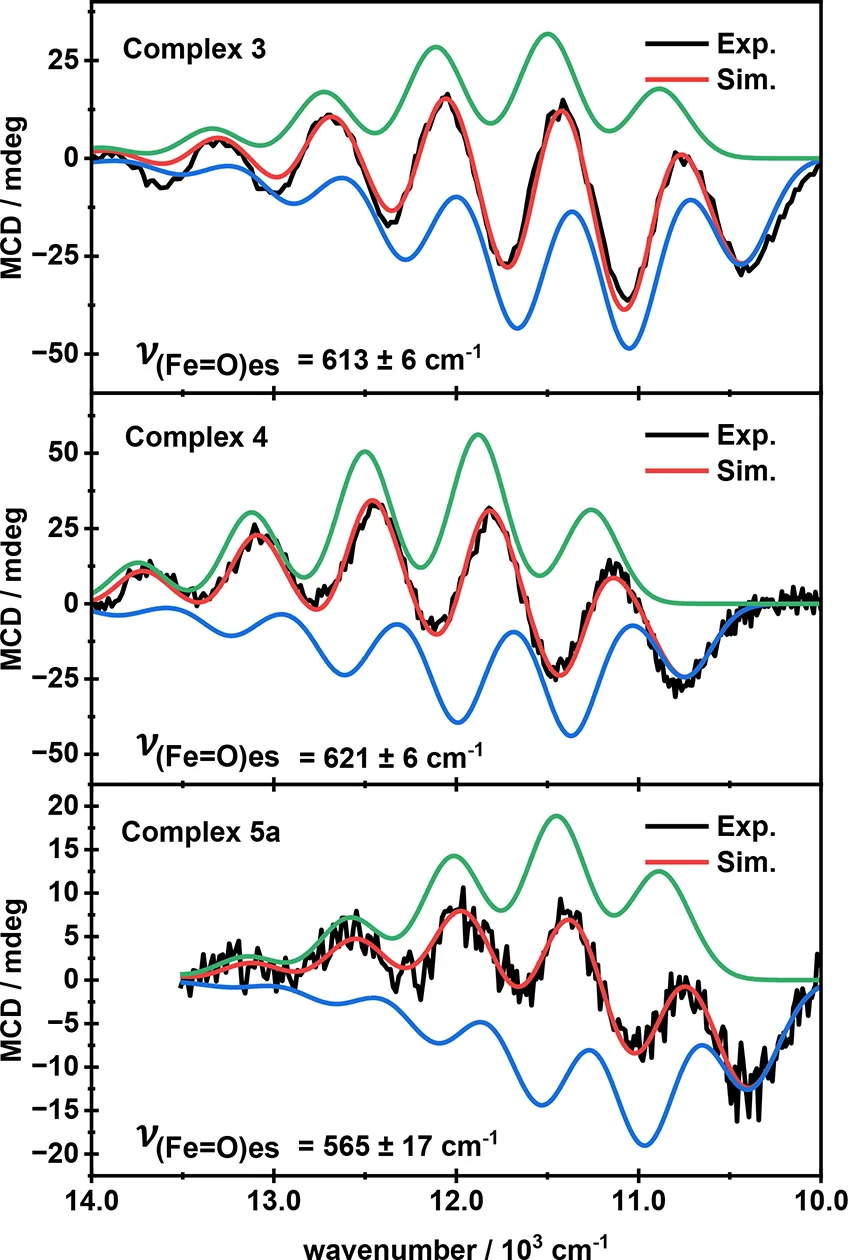
Franck–Condon progression simulation of 20 K MCD spectra of intra-t_2g_ transitions in [Fe^IV^O­(L^ax^)]^n+^ series.

In addition to those ligand field transitions discussed above, complexes **5a**/**5b** exhibit a new band at ∼14000 cm^–1^ (∼710 nm), which possesses a comparable intensity to the intra-t_2g_ transition in the UV−vis and MCD spectra, while no analogous transitions were detected for **3** or **4**. We thus tentatively attribute it to a weak charge transfer transition from the thiolate to the Fe^IV^O unit (S→Fe^IV^O). In line with this reasoning, a similar band at 1040 nm was also observed in **A** ^**TMCS**^,^17^ whereas complex **A** ^**NCMe**^ is devoid of such a low-energy NIR feature.^32^

### C–H Bond Activation Reactivity

The present series of oxidoiron­(IV) complexes with equatorial tetra­(NHC) ligation features a unique electronic structure with the Fe-3d_x^2^‑y^2^_ orbital high in energy and a very large triplet-quintet gap, leading to triplet-state-only reactivity.^24^ Hence, a comparison of their HAA reactivity should reveal intrinsic axial ligand effects not being overshadowed by different extents of two-state reactivity. As previously shown, complex **3** is able to activate weak C–H bonds up to around 80 kcal mol^–1^.^24^ Therefore, the reactivity of **3**, **4,** and **5a** ^37^ was studied with a set of substrates containing relatively weak C–H bonds, viz. 1,4-cyclohexadiene (CHD; BDE = 78 kcal mol^–1^), 9,10-dihydroanthracene (DHA; BDE = 77 kcal mol^–1^), 9H-xanthene (XanH; BDE = 75.5 kcal mol^–1^) and 10-methyl-9,10-dihydroacridine (AcrH; BDE = 73.7 kcal mol^–1^).^38^

For the previously studied parent complex **3**, upon addition of the above substrates, the formation of a hydroxidoiron­(III) intermediate with UV–vis absorptions at 448 and 558 nm was observed. In a slower follow-up process, this intermediate reacted further to give a μ-oxidodiiron­(III) product, [(L^NHC^Fe)_2_O]^4+^, with five-coordinate ferric ions.^24,30^ In contrast to this, upon addition of the C–H substrates to a solution of **4** in MeCN, the UV–vis features of **4** at 408 and 629 nm decrease, and a band at 339 nm is rising (Figure 12, top), which can be attributed to the formation of iron­(II) complex [L^NHC^Fe­(NCMe)_2_]^2+^ (**1**).^21^ Isosbestic points are present only during a first phase of the reaction (around 400 s at −40 °C for the substrate CHD), indicating that this reaction is a multistep process proceeding via a first HAA and a different follow-up reactivity compared to complex **3**, likely a second HAA by the initially formed hydroxidoiron­(III) intermediate to finally give **1**. Stronger binding of the anionic CF_3_COO^–^ compared to MeCN may prevent axial ligand dissociation from the initial hydroxidoiron­(III) intermediate and subsequent formation of the dimeric μ-oxidodiiron­(III) species with five-coordinate ferric ions. Upon addition of substrate to the *in situ* formed complex **5a** (Figure 12, bottom), the characteristic band at 447 nm for **5a** vanishes and a prominent new absorption band at 609 nm arises. The presence of multiple isosbestic points suggests a single-step transformation during HAA, forming the hydroxidoiron­(III) intermediate [L^NHC^Fe^III^(OH)­(S^*t*^Bu)]^+^. This is further supported by EPR spectroscopy of the frozen solution after HAA, which shows a rhombic spectrum characteristic for a low-spin (*S* = 1/2) Fe^III^ species (Figure S2). In contrast to the other hydroxidoiron­(III) intermediates lacking the *trans*-axial thiolate, [L^NHC^Fe^III^(OH)­(S^*t*^Bu)]^+^ is stable over the course of 4 h at −40 °C.

**Figure 12:**
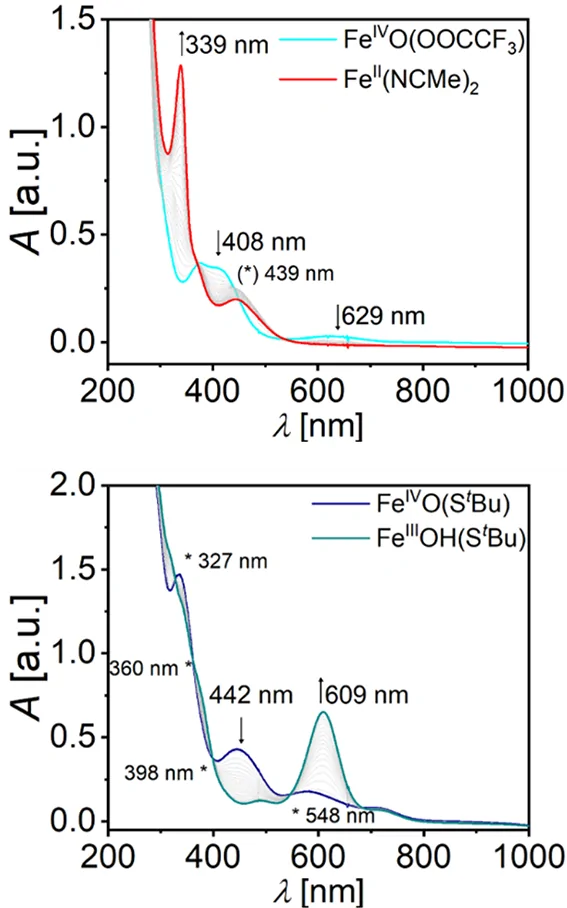
Spectral evolution of the optical features during the reaction of complex **4** (top) and **5a** (bottom) with 200 eq CHD in MeCN at −40 °C.

Analysis of the products obtained from the oxidation of DHA and CHD by oxidoiron­(IV) complexes **3**, **4**, and **5a** showed the formation of anthracene and benzene in yields of 82–87% and 88–100%, respectively (Table 2). These yields were calculated for a nonrebound process, in which a maximum of 0.5 eq anthracene/benzene per 1 eq Fe^IV^O complex can be obtained since two H atoms need to be abstracted from either substrate, and no evidence for any oxygenated products could be found.^39^ For quantifying the products in these experiments, crystalline material of the oxidoiron­(IV) complex was used in the case of **3** and **4**. Due to its instability, however, complex **5a** had to prepared *in situ* from its Fe^II^ precursor, which required the use of 2.8 eq of sPhIO for full conversion. Hence, some reoxidation of the hydroxidoiron­(III) intermediate (which forms after the HAA step) by excess sPhIO cannot be fully excluded, and product yields for **5a** should thus be viewed with caution.^40^ For substrates AcrH and XanH, some oxygenated products were observed, likely resulting from radical rebound after the HAA step. This has been confirmed by using ^18^O-labeled oxidoiron­(IV) complexes (prepared from sPhI^18^O) and ESI mass spectrometry, showing the formation of some Xan^18^O (^18^O-labeled 9*H*-xanthen-9-one) and Acr^18^O (^18^O-labeled 10-methylacridin-9­(10*H*)-one, see SI for more information about the product quantification and labeling experiments).

**Table 2:** Product Quantification of the Oxidation of DHA and CHD for Complexes 3, 4, and 5a

	3	4	5a
Yield anthracene [%]	84	87	82
Yield benzene [%]	100	90	88

Kinetic data were obtained using *in situ* prepared complexes **4** and **5a** (0.25 mM; prepared with sPhIO at −40 °C in MeCN) and varying excess of CHD under *pseudo*-first-order conditions. In the case of **4**, the decrease in the absorption of the oxidoiron­(IV) species at 408 nm was monitored until the break of the isosbestic points (at 0.25 mM concentration, it was not reliable to follow the absorption at 629 nm due to its low intensity). For complex **5a**, the increase in the absorption band corresponding to the formed hydroxidoiron­(III) intermediate (609 nm) was monitored. Similarly, for **3**, the bands corresponding to the formation of the hydroxidoiron­(III) intermediate (448 or in the range 525−550 nm) were chosen for reaction monitoring.^24^ As expected, complexes **5a** and **5b** show very similar reactivities (reactivity with DHA at 25 °C is depicted in Figure S20; see also Table S6), and therefore only complex **5a** was studied in greater detail. Plots of the observed rates *k*_obs_ versus the concentration of CHD for complexes **4** and **5a** are shown in Figure 13 (left). The obtained second-order rate constants *k*_2_ were corrected by the number of abstractable hydrogen atoms of the substrate used (four abstractable hydrogen atoms for CHD) to obtain *k*_2_’ constants compiled in Table 3. The parent complex **3** and the new complexes **4** and **5a**, with *trans*-axial CF_3_COO^–^ and ^*t*^BuS^–^, respectively, exhibit second-order rate constants that differ by not more than a factor of 10. Remarkably, the parent complex **3** shows the fastest rates among the complexes studied, while rates for **4** and **5a** are similar. This is contrary to the antielectrophilic trend observed for type **A** complexes based on the TMC macrocycle, where more donating axial ligands increased the reaction rate: compared to **A** ^**NCMe**^, the rate for HAA from the substrate CHD was higher by 1 order of magnitude in the case of **A** ^**TFA**^, and using DHA as the substrate, the reaction proceeded even 38 times faster with **A** ^**TMCS**^.^13^ This antielectrophilic trend for the TMC-based series of complexes was rationalized by the reduction of the orbital energy gap between the triplet and the more reactive quintet states, Δ*E*_Q‑T_, with increasing donor strength of the axial ligand (vide supra).^13,18^ For the present tetra­(NHC)-ligated systems participation of the quintet state is prohibited, and hence the most electrophilic **3** clearly shows the highest reaction rates. i.e., the trend of HAA reactivity is inverted compared to the TMC-based systems and follows the expected electrophilicity pattern. It should also be noted that **3** (with *trans*-axial MeCN) reacts 2–3 orders of magnitude faster compared to the corresponding TMC-based **A** ^**NCMe**^, despite **3** being confined to the triplet pathway.

**Figure 13:**
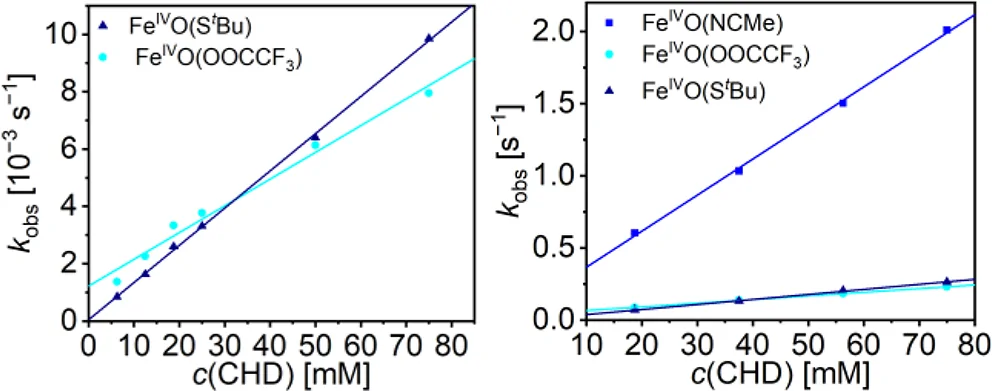
Plots of the observed reaction rates *k*_obs_ vs. concentration of CHD in MeCN at −40 °C (left) for complexes **4** (0.25 mM, following the band at 408 nm) and **5a** (0.25 mM, following the band at 609 nm) and at 25 °C (right) for complexes **3** (1 mM, following band at 550 nm), **4** (1 mM, following the band at 629 nm), and **5a** (1 mM, following the band at 609 nm).

**Table 3:** Second-Order Rates *k*_2_ and *k*_2_’ for Complexes 3, 4, and 5a with CHD at −40 and 25 °C in MeCN

	**3** ^24^		**4**		**5a**	
*T* [°C]	–40	25	–40	25	–40	25
*k*_2_(CHD) [M^–1^ s^–1^]	0.48	25.0	0.09	2.5	0.13	3.5
*k*_2_’(CHD) [M^–1^ s^–1^]	0.12	6.3	0.02	0.6	0.03	0.9

Figure 13 and Table 3 also contain data from kinetic traces recorded at 25 °C and 1 mM complex concentrations; in case of **4,** this allowed to monitor the disappearance of the band at 629 nm, which does not overlap with spectroscopic changes during follow-up processes. Trends in *k*_obs_ and *k*_2_ are similar at 25 °C, with **3** featuring around 10 times higher rates, while differences between the rates of **4** and **5a** are rather minor. Computational studies suggested that a five-coordinate oxidoiron­(IV) complex [L^NHC^FeO]^2+^ (**3′**) devoid of any *trans*-axial ligand should be significantly more reactive because dissociation of the *trans* ligand stabilizes the σ*­(Fe-3d_z^2^_) that could then serve as a more efficient electron-accepting orbital (EAO) in the HAA reaction step.^24,25,41^ Despite the relatively small Gibbs energy for MeCN binding (2.3 kcal mol^−1^ based on calculations, vide infra), we consider potential dissociation of the *trans*-MeCN from **3** and involvement of **3′** to be unlikely under the present conditions because the experiments were performed in MeCN solution; this is also supported by the much better agreement of the experimental Gibbs free activation barrier with that calculated for MeCN-ligated **3** (vide infra).

Eyring analyses of the reactions of complexes **3**, **4**, and **5a** with CHD (Figure 14, left) reveal that Δ*G* ^‡^_20 °C_ is lowest for **3**, but overall, the three complexes show rather similar free energy barriers (Table 4), reflecting the relatively minor variations in HAA rates.

**Figure 14:**
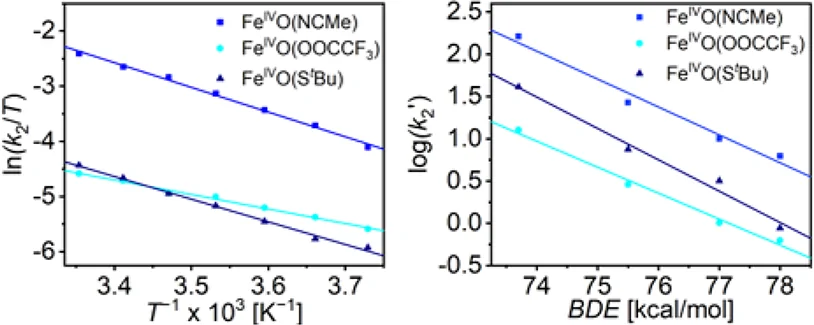
Left: Eyring plot of the reactions of complexes **3**, **4**, and **5a** with 75 eq CHD at different temperatures. Right: Plot of log­(*k*_2_’) against the BDE of different substrates (AcrH, XanH, DHA, CHD) for the three complexes.

**Table 4:** Activation Parameters for the Reaction of Complexes 3, 4, and 5a with CHD, Derived from an Eyring Analysis

	3	4	5a
Δ*H* ^‡^ [ kcal mol^−1^]	8.9 ± 0.3	5.2 ± 0.2	8.2 ± 0.3
Δ*S* ^‡^ [cal K/mol]	–22.1 ± 1.0	–38.8 ± 0.7	–28.7 ± 0.9
Δ*G* ^‡^_20 °C_ [ kcal mol^−1^]	15.4 ± 0.6	17.6 ± 0.4	16.6 ± 0.5

Plots of log­(*k*_2_’) against the bond dissociation energy (BDE) of the four different substrates (Figure 14, right) indicate linear Bell-Evans-Polanyi type correlations with slopes of −0.31 (complex **3**), −0.33 (complex **4**) and −0.37 (complex **5a**),^42^ which is in the range of previously studied oxidoiron­(IV) complexes^13,24,43^ and is strong evidence for HAA as the rate-limiting step. It also suggests that the proton-coupled electron transfer (PCET) process is concerted and not significantly biased toward a mechanism of initial proton transfer (PT) or electron transfer (ET).

### Kinetic Isotope Effects and Tunneling Contributions

To gain further mechanistic information, temperature-dependent kinetic isotope effects (KIEs) were explored. Reactions of complexes **4** and **5a** with DHA and deuterated DHA (DHA-d_4_) at different temperatures were compared with previously obtained data for complex **3** (three temperatures were already studied before,^24^ and the rt measurements for **3** were added in this work); the results are shown in Figure 15 (left). KIE values obtained at rt (Table 5 and Figure 15) are higher with increasing donor strength of the axial ligand, from 4.8 for complex **3** (MeCN) via 8.7 (CF_3_COO^–^) for complex **4** to 17.1 for complex **5a** (^*t*^BuS^–^). At −40 °C a rather high KIE for complex **3** of 32 is obtained (Table 6).^24^ Similarly high values were observed for the natural enzyme taurine α-KG dioxygenase (∼35),^44^ whereas most TMC-coordinated iron complexes show values between 10 and 20 (at 0 °C in MeCN).^13,27^ Linear extrapolation of KIE values for **4** and **5a** to −40 °C gives values around 39 (±9) for complex **4** and 109 (±29) for complex **5a** (see SI for details of the extrapolation; note that below 10 °C, **4** and **5a** react too slowly with DHA-d_4_ to determine KIEs experimentally). These enormously high KIE values in the reaction with DHA, in particular for **5a**, support HAA as a rate-determining step in these reactions and suggest increased nonclassical hydrogen tunneling effects. This is in line with reports for TMC-based ferryl complexes [(TMC)­Fe^IV^O­(L^ax^)]^n+^ where **A** ^**TMCS**^ with a strongly donating *trans*-axial thiolate showed the largest tunneling-augmented KIE (80 at −20 °C in MeOH).^27^ It should be noted that the Δ*H* ^‡^ extracted from Eyring plots of the H-transfer rate (Table 4) may be smaller than the “true” (zero-point-corrected) barrier when substantial quantum-mechanical tunneling occurs, because tunneling allows the reaction to proceed below the classical transition-state energy; the corresponding apparent Δ*S* ^‡^ may consequently be driven in the opposite direction.

**Figure 15:**
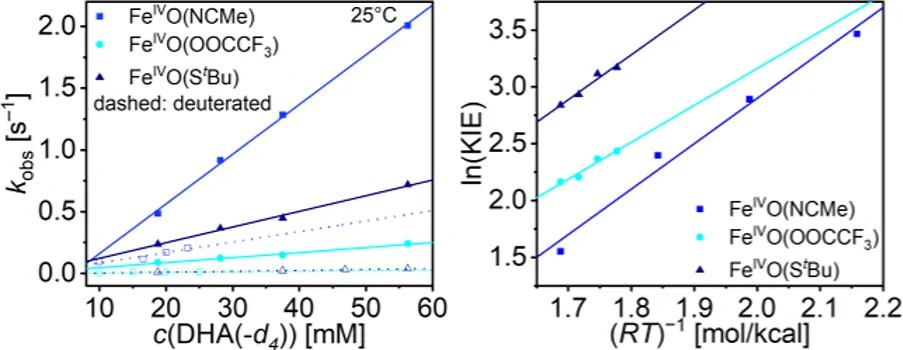
Left: Plot of *k*_obs_ against *c*(DHA-(*d*_4_)) at 25 °C; right: plot of ln­(KIE) vs. RT^–1^.

**Table 5:** Second-Order Rate Constants for the Reaction of Complexes 3, 4, and 5a with DHA and Deuterated DHA-d_4_ at 25 °C and the Corresponding KIE Values

	**3**	**4**	**5a**
*k*_2_’(DHA) [M^–1^ s^–1^]	10.0	1.0	3.2
*k*_2_’(DHA-*d*_4_) [M^–1^ s^–1^]	2.1	0.1	0.2
KIE	4.8	8.7	17.1

**Table 6:** Experimental Temperature-Dependent KIE Values for Complexes 3, 4, and 5a and Their Analysis

	**3**	**4**	**5a**
KIE (−40 °C)	32	-	-
KIE (−20 °C)	18	-	-
KIE (0 °C)	11	-	-
KIE (10 °C)	-	11.4	23.8
KIE (15 °C)	-	10.6	22.5
KIE (20 °C)	-	9.1	18.8
KIE (25 °C)	4.8	8.7	17.1
*E*_a_(D)–*E*_a_(H) [kcal mol^−1^]	4.0 ± 0.4	3.2 ± 0.5	4.0 ± 0.6
ln(*A*_H_/*A*_D_)	–5.1 ± 0.7	–3.3 ± 0.9	–3.8 ± 1.0

The importance of tunneling can be evaluated by temperature-dependent KIE analysis, which allows to assess the influence of the tunneling effects on the activation energy and on the Arrhenius prefactor.^45,46^ Therefore, ln­(KIE) was plotted versus (RT)^−1^, where the slope of this plot gives the differences in activation energy (*E*_a_(D)–*E*_a_(H)) and the intercept represents the difference between the exponential prefactors (ln­(*A*_H_/*A*_D_)). With these parameters, different regimes with increasing importance of tunneling can be defined. In semiclassical treatments, KIEs are often seen as the loss of zero-point energy in the transition state. This scenario mainly affects the isotope effect on the activation energy, whereas there should be just a minimal effect on *A*. In this case, the intercept is expected to be zero, while the slope should be smaller than or equal to 1.4 kcal mol^–1^.^45^ In this context, proton coupling would increase the magnitude of *k*_H_ and decrease the temperature dependence, yielding values for the slope greater than 1.4 kcal mol^–1^ and extrapolated intercepts below zero. If quantum mechanical treatments are applied, both H and D can tunnel intensively. This would lead to a small effect on the slope and a positive intercept.^46^ All complexes **3**, **4**, and **5a** studied in this work show an extrapolated intercept lower than zero and a temperature dependence steeper than 1.4 kcal mol^–1^ (Figure 15 right and Table 6). This is in agreement with a semiclassical transition state theory with modest tunneling contributions. Therefore, experimental findings indicate that all complexes show similar tunneling behavior, and that differences are rather subtle, in accordance with the overall rather similar free energy barriers derived from Eyring analyses. A more detailed comparison was then pursued computationally.

### Theoretical Investigations on HAA Reactivity

To obtain detailed insights into the reactivity differences between complexes **3**, **4** and **5a**, we performed DFT-based investigations of the reaction energetics and electronic structures. Along with complexes **3**, **4,** and **5a**, the five-coordinated version of complex **3** devoid of the *trans* ligand (**3′**) was also investigated for the HAA reactivity with the substrate CHD. The reaction energetics (Δ*G*, kcal mol^−1^) are presented with respect to the free species (complex + CHD) to approximately account for the entropic contribution to the reaction barriers (Figure 16). For complexes **3** and **3′**, both HAA transition states for the σ- (^3^TS_σ_) and π-pathway (^3^TS_π_) were successfully located. In line with previous calculations,^24,25^ for the five-coordinated complex **3′** the σ-pathway involves a lower barrier (11.0 kcal mol^−1^) than the π-pathway (15.7 kcal mol^−1^), whereas the scenario is the opposite in the case of the six-coordinated species **3** with *trans*-bound MeCN, where the π-pathway features a slightly lower barrier (15.1 kcal mol^−1^) than the corresponding σ-pathway (15.3 kcal mol^−1^). Most importantly, this direct comparison of the HAA barriers reflects the destabilization of the σ*­(Fe-3d_z^2^_) by the *trans*-axial ligand, which disfavors the σ-pathway where the Fe-3d_z^2^_ serves as EAO. While the formation of the five-coordinate species **3′** through the dissociation of MeCN appears feasible in light of the almost thermoneutral MeCN binding free energy of +2.3 kcal mol^−1^, the experimentally determined value of Δ*G* ^‡^_20 °C_ (15.4 ± 0.6 kcal mol^−1^) matches closely with the DFT calculated value for six-coordinate **3** (15.1 kcal mol^−1^), while the calculated barrier for the five-coordinate complex **3′** is much lower (11.0 kcal mol^−1^). This strongly supports the assumption that **3′** is not relevant under the present conditions when the reactions are performed in MeCN solution (vide supra).

**Figure 16:**
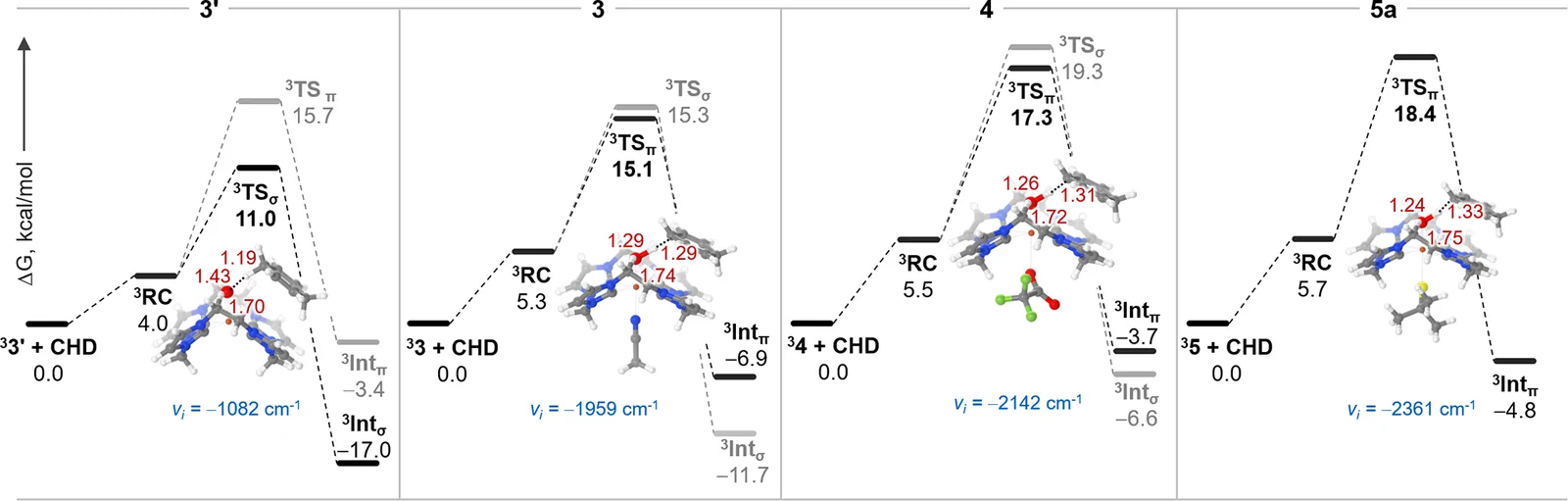
Computed Gibbs free energy (Δ*G*, kcal mol^−1^) profiles of the HAA from CHD by complexes **3′** (five-coordinated), **3**, **4**, and **5a** at the DFT-B3LYP level of theory. Optimized geometries of the lowest energy transition states, their key geometrical parameters (in red), and imaginary frequency are presented along with the corresponding reaction profile. The lower energy pathway is presented with black lines, and the higher energy one is presented with gray lines.

For complex **4** with a *trans*-CF_3_COO^–^ both the σ and π HAA transition states could be successfully located, with the π-pathway lying marginally lower in energy (Figure 16). The calculated Gibbs free energy barrier for the lower-energy π-pathway is 17.3 kcal mol^−1^, which is in excellent agreement with the experimentally determined value of 17.6 ± 0.4 kcal mol^−1^ (Table 4). Unlike the five-coordinate complex, **3′**, both six-coordinate complexes **3** and **4** have the π-pathway (^3^TS_π_) lower in energy as compared to the σ-pathway, because any *trans*-ligand raises the energy of the EAO, Fe-d_z^2^_, involved in the σ-pathway.

The CASSCF­(12,12)-derived natural orbitals do not show any appreciable differences in the electronic structures of complexes **3′**, **3**, **4**, and **5a**. However, the calculated d-d transition energy d_*xz*,yz_ → d_z^2^_ is appreciably higher for complex **5a** (Table S10). This indicates an energetically high-lying Fe-d_z^2^_ orbital for complex **5a** as compared to the other complexes, in agreement with the UV–vis and MCD data (vide supra; Figures 4 and 10). Therefore, the Fe-d_z^2^_ EAO for the σ-pathway becomes inaccessible for **5a**. In line with this reasoning, the σ transition state for HAA could not be located in case of **5a** despite the best of our efforts. Only the π transition state (^3^TS_π_) involving a Gibbs free energy of 18.4 kcal mol^−1^ could be located, which is about ∼1 kcal mol^−1^ higher as compared to that of complex **4**. While this DFT-calculated HAA barrier for **5a** agrees reasonably well with the experimentally determined value (16.6 ± 0.5 kcal mol^−1^), the agreement is worse than for **3** and **4**, and the relative order of Δ*G* ^‡^_20 °C_ for **4** and **5a** in the calculations and experiments differs. We attribute this to a more significant tunneling contribution for the HAA pathway of **5a**, which effectively lowers the barrier compared to complex **4**, in agreement with the significantly higher KIE value determined for complex **5a** (KIE­(25 °C) = 17.1 for **5a** vs 8.7 for **4**; Table 5). Indeed, if we take a closer look at the calculated intrinsic reaction coordinates (IRCs) of complexes **3**, **4**, and **5a** (Figure 17), the transition state (^3^TS_π_) associated with complex **5a** involves an appreciably smaller width as compared to complex **4**, suggesting a lower effective barrier due to increased H-tunneling. This is in accordance with reports on the TMC-based ferryl complexes, where **A** ^**TMCS**^ with a strongly electron-donating *trans*-thiolate favors tunneling in comparison to other [(TMC)­Fe^IV^O­(L^ax^)]^n+^ complexes.^20,27^

**Figure 17:**
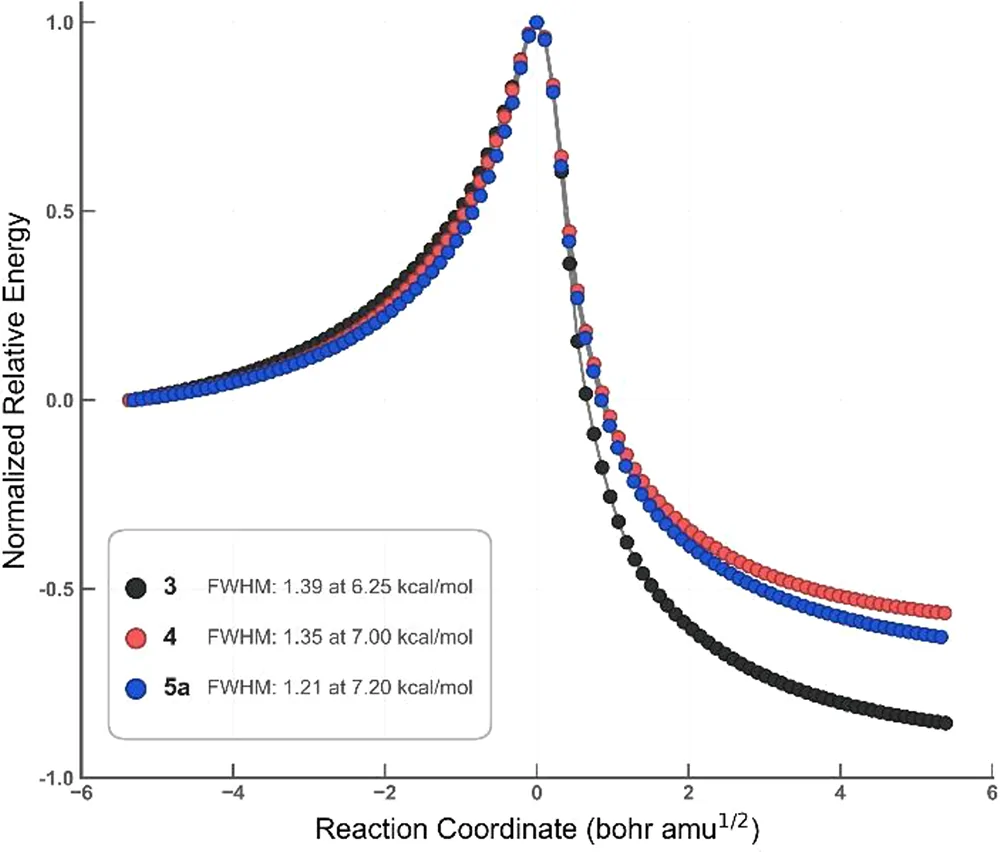
Intrinsic reaction coordinates (IRCs) calculated for the lowest energy transition states ^3^TS_π_ for complexes **3**, **4**, and **5a**. The estimated widths (in bohr·amu^1/2^) and full width at half maxima (fwhm), are shown along with the IRCs.

According to the Bell–Evans–Polanyi (BEP) principle, the reaction barrier is correlated to the driving force. Therefore, the bond dissociation free energies (BDFE) of the Fe^III^–OH intermediates that result after the HAA step would be a reflection of the relative HAA reactivity. The calculated BDFEs of the intermediates associated with the lowest energy ^3^TS_π_ pathways for complexes **3**, **4**, and **5a** (^3^Int_π_) are 80.0, 75.6, and 77.8 kcal mol^−1^, respectively (and 88.2 kcal mol^−1^ for **3′**, ^3^Int_σ_). This nicely mirrors the trends in reactivity observed experimentally (**3** > **5a** > **4**) and the relative Δ*G* ^‡^_20 °C_ values derived from the Eyring analysis. Importantly, a higher driving force is found for the π-pathway of **5a** as compared to that of **4**.

## Summary and Conclusions

In this work, a set of Fe^IV^=O complexes supported by a macrocyclic tetracarbene ligand and bearing different anionic *trans*-axial ligands CF_3_COO^–^ (**4**), ^*t*^BuS^–^ (**5a**) and AdaS^–^ (**b**) has been isolated and thoroughly investigated. Together with our previously reported tetracarbene-ligated Fe^IV^=O complex with *trans*-axial MeCN (**3**) (or EtCN in **3′**) this constitutes a valuable series [(L^NHC^)­Fe^IV^O­(L^ax^)]^n+^ to experimentally probe the effect of axial ligands on the HAA reactivity of biorelevant Fe^IV^=O intermediates toward C–H substrates, complementing and contrasting the findings for the only other comparable series of Fe^IV^=O species in a tetragonal ligand field, viz. the cyclam-based [(TMC)­Fe^IV^O­(L^ax^)]^n+^. The molecular structures of **4** and **5b** have been established crystallographically, which in combination with the previously reported structure of **3** provides a unique set of structurally authenticated organometallic Fe^IV^=O species that differ only by the *trans*-axial ligand, including a *trans*-thiolate in **5b**. Their electronic structures have been comprehensively investigated using a bouquet of methods including magnetic measurements, field-dependent ^57^Fe Mössbauer and MCD spectroscopies, and all [(L^NHC^)­Fe^IV^O­(L^ax^)]^n+^ exhibit a well-isolated *S* = 1 ground state with nb­(Fe-d_*xy*_)^2^π*­(Fe-d_*xz*,yz_)^2^σ*­(Fe-d_*z* ^2^_)^0^σ*­(Fe-d_*x* ^2^–*y* ^2^_)^0^ configuration where the strong σ-donor character of the equatorial tetracarbene ligand lifts the Fe-d_*x* ^2^–*y* ^2^_ energetically above the Fe-d_*z* ^2^_. In agreement with DFT and CASSCF calculations in this and prior work,^22,24,25^ this unusual orbital ordering leads to a very large triplet-quintet gap that makes the quintet state inaccessible. This prevents significant mixing of the *S* = 1 and *S* = 2 states, which translates into rather small zero-field splitting parameters *D* < 20 cm^–1^ determined by SQUID magnetometry and applied field Mössbauer spectroscopy. Both experiment and theory show Fe^IV^=O stretching frequencies in the order **3** ≥ **4** > **5a**, with a very low ν̃_Fe–O_ = 761 cm^–1^ for **5a** (the first experimentally determined ν̃_Fe–O_ for a synthetic nonheme oxidoiron­(IV) complex with *trans*-axial thiolate). This correlates with the order of Fe=O bond lengths, and the elongation of the Fe^IV^=O bond upon *trans*-thiolato coordination likely contributes to the increase of the ^57^Fe Mössbauer isomer shift δ in **5a**/**5b** (−0.09 mm s^–1^) compared to **3** and **4** (−0.13 and −0.14 mm s^–1^, respectively).

Because of their very large triplet-quintet gap, the HAA reactivity of all L^NHC^-based Fe^IV^=O complexes is restricted to triplet pathways, which allows intrinsic trends in hydrogen atom abstraction (HAA) to be identified experimentally without interference from two-state reactivity (TSR). DFT calculations suggest that for all these six-coordinate complexes the π-pathway, where the π*_x/y_(Fe-d_*xz*,yz_) are the EAOs, is favored. For **3** and **4** with weak σ-donor *trans*-axial MeCN and CF_3_COO^–^, ^3^TS_σ_ is only slightly above ^3^TS_π_, but in the case of **5a**/**5b** the destabilization of the σ*­(Fe-3d_*z* ^2^_) by the *trans*-axial thiolato ligand is so pronounced that the Fe-d_*z* ^2^_ EAO for the σ-pathway becomes inaccessible. Kinetic measurements with substrates featuring C–H bond dissociation energies up to 78 kcal mol^–1^ reveal that the different axial ligands have only a modest impact on reactivity, following roughly an electrophilic order of L^ax^ = MeCN > CF_3_COO^–^ ≈ RS^–^. This unusual minor effect of axial ligands on HAA corroborates that TSR is not involved for the present series of L^NHC^-ligated complexes, and it contrasts the findings for the related series of TMC-based systems, where TSR dominates and leads to opposite HAA reactivity trends (L^ax^ = MeCN < CF_3_COO^–^ < RS^–^).^13,18^

The L^NHC^-based Fe^IV^=O complexes show high KIE values in the HAA reaction with DHA, the KIE being highest for **5a** with a *trans*-thiolate. Such large KIEs for HAA processes are usually indicative of reactive *S* = 1 spin states of the TS.^12^ A similarly large KIE has also been observed for the TMC-based Fe^IV^=O complex with a *trans*-thiolate, **A** ^**TMCS**^, which proceeds via an *S* = 2 TS. This was reported to reflect increased tunneling with stronger donicity of the *trans*-axial ligand L^ax^, which was proposed to be responsible for the antielectrophilic HAA reactivity trend found for the TMC-based systems.^27^ For the present series of L^NHC^-based Fe^IV^=O complexes, experimentally determined temperature-dependent KIEs, supported by density functional theory and ab initio calculations, are consistent with a semiclassical reaction model involving moderate tunneling contributions. Indeed, calculated intrinsic reaction coordinates show a barrier for the relevant (^3^TS_π_) transition state that is only slightly narrower in the case of **5a** with a *trans*-thiolate, which we surmise is related to the Fe=O bond elongation in **5a**/**5b** inducing a slightly more symmetric O···H···C transition state.

For TMC-based systems with various axial ligands, ^5^TS_σ_ on the *S* = 2 surface generally is the lowest energy TS, with a close-to-linear Fe–O···H angle and σ*­(Fe-d_*z* ^2^_) being the EAO. In that case, a stronger donicity of the *trans*-axial ligand, L^ax^, was found to have a major effect on the slimness of the potential energy barrier along the reaction coordinate by increasing the alternating charges in the O···H···R arrangement, thus leading to a prevailing tunneling effect on HAA (with **A** ^**TMCS**^ being much more reactive than **A** ^**NCMe**^).^12,20,27^ In contrast, the present L^NHC^-based triplet-only systems follow a π-trajectory with a bent Fe–O···H angle and the π*_x/y_(Fe-d_*xz*,yz_) serving as EAO. Here, the donicity of L^ax^ appears to have a much smaller effect on the barrier width along the reaction coordinate, and hence tunneling only attenuates the electrophilic trend, but it does not reverse it. Hence, **3** with L^ax^ = MeCN remains more reactive in HAA than thiolato-ligated **5a**/**5b**. We conclude that TSR remains a dominant factor leading to the antielectrophilic trend in the case of the TMC-based Fe^IV^=O complexes, while the present series of L^NHC^-based Fe^IV^=O systems reveals the intrinsic and rather small effect of the *trans*-axial ligands for a pure triplet state scenario. Overall, this combined experimental and computational study clarifies how axial ligands—including thiolates—affect the spectroscopic signatures, properties, and reactivity of *S* = 1 ferryl intermediates.

## Supplementary Material



## Notes

Synthetic procedures and materials; further spectroscopic (Mössbauer, UV–vis, EPR, MCD) and magnetic data; details of the kinetic experiments; detailed crystallographic information; details about DFT and CASSCF calculations; Cartesian coordinates of all optimized structures (PDF)

## Notes

All other data for this paper, including data from experimental methods and computational studies, are available at the Göttingen Research Online server at https://doi.org/10.25625/SS9KST.

## Abbreviations

AcrH: 9-methylacridine
BDE: bond dissociation energy
CHD: cyclohexadiene
DFT: density functional theory
DHA: dihydroanthracene
ET: electron transfer
HAA: hydrogen atom abstraction
HAT: hydrogen atom transfer
IRPD: infrared photodissociation
KIE: kinetic isotope effect
MCD: magnetic circular dichroism
OAT: oxygen atom transfer
TFA: trifluoroacetate
Xan: xanthene
TPA: tris­(2-pyridylmethyl)­amine
