# Supplementary material for: Axial Ligand Effects on Electronic Structures and H‑Atom Abstraction by Oxidoiron(IV) Complexes Featuring Triplet-Only Reactivity
Source: JACS Au. 2026 Sep 14;6(9):5130–44. doi: 10.1021/jacsau.6c00781 (PMC13625729; doi:10.1021/jacsau.6c00781)
Supplement: Supplementary file 1 [file au6c00781_si_001.pdf]

Supporting Information

for

Axial Ligand Effects on Electronic Structures and H-Atom Abstraction by Oxidoiron(IV) Complexes Featuring Triplet-Only Reactivity

Isabelle Becker,[†,&] Massimiliano Morganti,[†,&] Haowei Chen,[#] Akhil Bhardwaj,[§] Pilar Bologna,[†] Guilherme Tripodi,[⊥] Serhiy Demeshko,[†] Sebastian Dechert,[†] Jana Roithová,[⊥] Frank Neese,[#] Bhaskar Mondal,[§,] Shengfa Ye,[‖,*] Franc Meyer[†,$*]*

[†] University of Göttingen, Institute of Inorganic Chemistry, Tammannstraße 4, 37077 Göttingen, Germany

[⊥] Institute for Molecules and Materials, Radboud University, Heyendaalseweg 135, 6525 Nijmegen, Netherlands

[§] School of Chemical Sciences, Indian Institute of Technology Mandi, Kamand, Himachal Pradesh 175075, India

[#] Max-Planck-Institut für Kohlenforschung, 45470 Mülheim an der Ruhr, Germany

[‖] Key Laboratory of Bioinorganic and Synthetic Chemistry of Ministry of Education, Guangdong Basic Research Center of Excellence for Functional Molecular Engineering, School of Chemistry, IGCME, Sun Yat-sen University, Guangzhou 510275, P.R. China

[$] University of Göttingen, International Center for Advanced Studies of Energy Conversion (ICASEC), Tammannstr. 6, 37077 Göttingen, Germany

[&] these authors contributed equally to the work

* To whom correspondence should be addressed
Email: franc.meyer@chemie.uni-goettingen.de
bhaskarmondal@iitmandi.ac.in
shengfa.ye@dicp.ac.cn

Table of Contents

1 Methods and Instruments S2
2 Synthetic Procedures S4
3 Further Spectroscopic and Magnetic Data S5
4 MCD Spectra S8
5 X-Ray Crystallography S14
6 Further Kinetic Data and Product Quantification S16
7 DFT Calculations S25
8 References S29
9 Exemplary Kinetic Traces and Fits S32
10 Optimized Coordinates S101

1 Methods and Instruments

All reactions were carried out under dry argon using standard Schlenk techniques, or in a glovebox (MBRAUN UNIlab plus ECO or GS MEGA E-Line) under dinitrogen or argon atmosphere with less than 0.5 ppm O_2 and H_2O. Solvents were dried and degassed by standard procedures before use. Chemicals were purchased from commercial sources and used as received. Complexes **1**,[1] **2a/2b**[2] and **3**[1] were prepared according to the procedures previously reported. 1,4-Cyclohexadiene was stirred over $CaCl_2$ for 2d and vacuum distilled at rt. 9,10-Dihydroanthracene and xanthene were recrystallized twice from hot EtOH. 9,10-Tetradeutero-9,10-dihydroanthracene (DHA-d_4) was prepared through a previously reported procedure and recrystallized from hot EtOH.[3] 10-Methyl-9,10-dihydroacridine was synthesized according to the previously published procedure and was recrystallized from hot EtOH.[4]

UV-vis spectra of solutions were recorded using quartz cuvettes with 1 cm pathlength. A Cary 8454 spectrometer from Agilent Technologies equipped with an Unisoku Cryostat (CoolSpek, Unisoku Scientific Instruments) for temperature control and magnetic stirring was used for single spectra as well as kinetic traces in the kinetic mode. UV-vis-NIR spectra were collected with a Varian Cary 5000 instrument. Spectra were analyzed by Cary Win UV software.

For gas chromatography (GC), GC-FID measurements were performed in collaboration with the mass spectrometry facility of the Department of Chemistry of the Georg-August-University Göttingen using an Agilent 7890A gas chromatograph including an FID detector.

Temperature dependent stopped-flow analysis was performed in a range from 268 K to 298 K. The cryo unit allowed for precise temperature control before mixing the solutions in the optical cell. The kinetic data were obtained using a TgK Scientific Hi-Tech KinetAsyst™ CSF-61DX2 double mixing cryo-stopped-flow system in single-mixing mode. A xenon lamp was used as the light source and interfaced with a KinetaScan Rapid Scanning CCD (Charge Coupled Device) detector to provide UV-vis spectra in the range of approximately 300-850 nm. Data acquisition and processing were performed using the Kinetic Studio software (TgK Scientific). To ensure anaerobic conditions, all mixing chambers were rinsed with dry, deoxygenated solvent. Complex and substrate solutions were prepared in syringes in the glovebox and then anaerobically loaded into one of the reagent reservoirs.

X-band EPR spectra were recorded using a Bruker E500 ELEXSYS spectrometer equipped with a standard cavity (ER4102ST, 9.45 GHz). Temperature control was achieved by the use of an Oxford Instruments Helium flow cryostat (ESP910) and an ITC-4 Oxford temperature controller. Simulations of experimental data were performed using the EasySpin program.[5]

Zero-field Mössbauer spectra were recorded with a ^{57}Co source in a Rh matrix using an alternating constant acceleration Wissel Mössbauer spectrometer operated in the transmission mode and equipped with a Janis closed-cycle helium cryostat. Isomer shifts are given relative to iron metal at ambient temperature. Simulation of the experimental data was performed with the Mfit program (E. Bill, Max-Planck Institute for Chemical Energy Conversion, Mülheim/Ruhr, Germany) using Lorentzian line doublets.

Magnetic field Mössbauer spectra were recorded with a ^{57}Co source in a Rh matrix using an alternating constant acceleration Wissel Mössbauer spectrometer operated in the transmission mode perpendicular to the γ-rays and equipped with a OptiCool closed-cycle helium cryostat

from Quantum Design. Because of the design of our Mössbauer cryomagnet system, samples for the VF/VT Mössbauer measurements have to be loaded at rt, because the magnet and the sample space are not independent load-lock compartments. Consequently, changing the sample requires opening the relevant part of the instrument, which can only be done safely when the magnet/sample environment is at room temperature. Therefore, the exposure time was kept as short as possible, and the samples were cooled immediately after insertion in order to minimize decomposition. Isomer shifts are given relative to iron metal at ambient temperature. Simulation of the experimental data was performed with the MX program (E. Bill, Max-Planck Institute for Chemical Energy Conversion, Mülheim/Ruhr, Germany) by diagonalization of the spin Hamiltonian for the electronic and nuclear spins:

$$\widehat{H} = \mu_B \vec{S} \boldsymbol{g} \vec{B} + D\left[\hat{S}_z^2 - \frac{1}{3}S(S+1)\right] + \vec{I}\boldsymbol{A}\vec{S}.$$

Temperature-dependent magnetic susceptibility measurements were carried out with a Quantum-Design MPMS3 SQUID magnetometer equipped with a 7 Tesla magnet in the range from 300 (for **4**), 250 (for **5a**) or 210 (for **5b**) to 2.0 K at a magnetic field of 0.5 T. The powdered samples were contained in a polycarbonate capsule and fixed in a non-magnetic sample holder. Each raw data file for the measured magnetic moment was corrected for the diamagnetic contribution of the sample holder and the polycarbonate capsule. The molar susceptibility data were corrected for the diamagnetic contribution. Experimental susceptibility and variable temperature – variable field (VTVH) data were simultaneously modelled by using a fitting procedure to the spin Hamiltonian for Zeeman splitting and zero-field splitting: $\widehat{H} = g\mu_B \vec{B}\vec{S} + D\left[\hat{S}_z^2 - \frac{1}{3}S(S+1)\right]$. Temperature-independent paramagnetism (*TIP*) was included according to $\chi_{calc} = \chi_{mono} + TIP$. Simulation of the experimental magnetic data was performed with the *julX_2s* program (E. Bill: Max-Planck Institute for Chemical Energy Conversion, Mülheim/Ruhr, Germany).

IRPD measurements were performed with the ISORI instrument described elsewhere.[6,7] The ions were transferred to the gas phase with a ESI ionization source. A first quadrupole allowed for the isolation of the oxoiron(IV) species, that were subsequently injected in a cryo-cooled wire quadrupole trap operating at a temperature of 3 K and a frequency of 1 Hz. Ions were trapped with a helium buffer gas for 130 ms and partially transformed into the helium adducts. After a delay of 400 ms an optical parametric oscillator/amplifier (OPO/OPA) operating at 10 Hz was used to irradiate the ions with 8 photon pulses on alternate cycles. After an additional delay, the ions were extracted from the cryo-cooled wire quadrupole trap and mass analysed by a second quadrupole and a Daly type detector operating in ion-counting mode determined their number *N* when analysing the irradiated ions, and the total number of unirradiated ions N_0 when the irradiation was blocked with a mechanical shutter. The spectra were plotted reporting values of $(1-N/N_0)$ vs the wavenumber of the irradiation. The accuracy of the measurement is ±3 cm^{-1}. ^{18}O labeled complexes were obtained by using ^{18}O-labeled sPhIO (for **5a**/**5b**) or by the addition of $H_2{}^{18}O$ into the cold MeCN solution of the complexes (for **3** and **4**).

General procedure for the HAA Kinetic Measurements for the Reaction of Complexes 3, 4, 5a and 5b (1 mM) with substrates at 25 °C. 2 mM stock solutions were prepared in a glovebox by dissolving in 30 mL of dry and degassed MeCN respectively: a) 47.1 mg (60 µmol)

of complex $[L^{NHC}Fe(NCMe)_2](OTf)_2$ (**1**); b) 47.1 mg (60 µmol) of complex $[L^{NHC}Fe(NCMe)_2](OTf)_2$ (**1**) and 8.2 mg (60 µmol) of dry sodium trifluoroacetate; c) 41.0 mg (60 µmol) of complex $[L^{NHC}Fe(S^tBu)(NCMe)](OTf)$ (**2a**); d) 45.7 mg (60 µmol) of complex $[L^{NHC}Fe(SAdamantyl)(NCMe)](OTf)$ (**2b**). The stock solutions were cooled down to −35 °C and 3 mL of each were treated with 5.7 mg (17 µmol, 2.8 eq.) of solid sPhIO. The reaction mixture was left reacting for 1 h at low temperature. In the meantime, substrate stock solutions were prepared by dissolving the appropriate amount of substrate in MeCN (for CHD) or into a MeCN/DCM mixture (up to 1:1, for DHA, Xanthene and 9-Methylacridine). Then, 3 mL of the substrate solution and 3 mL of the filtrated oxoiron(IV) complex solution were quickly charged into the syringes of the stopped-flow instrument with thermostated reaction cell. The reactions took place after mixing of equal amounts of oxoiron(IV) and substrate solutions into the reaction cell, obtaining reaction mixtures with 1 mM complex concentration. The equivalents of substrate were varied in the range of 10–75 eq. and each experiment with 3 to 13 trials was carried out at least twice. In case of deuterated substrate, the reaction was carried out in a regular UV/Vis spectrometer if the reaction became too slow.

2 Synthetic Procedures

Synthesis of $[L^{NHC}Fe(O)(CF_3COO)](OTf)$ (4)

Complex **1** $[L^{NHC}Fe(MeCN)_2](OTf)_2$ (30.0 mg, 38 µmol, 1.0 eq.) and CF_3COONa (5.5 mg, 40 µmol, 1.1 eq.) was dissolved in 3 mL of dry and degassed MeCN and was cooled to −35 °C. Solid sPhIO (36.4 mg, 107 µmol, 2.8 eq.) was added and the reaction mixture was left reacting at −35 °C for 1 h. During the reaction the color of the solution changed from yellow to green. Unreacted sPhIO was filtered away and slow diffusion of diethyl ether into the MeCN solution allowed for the isolation of crystalline material of complex **5** suitable for XRD analysis (19.9 mg, 29 µmol, 76%).

MB (solid, 80 K): $\delta = -0.14$ mm s^{-1}; $\Delta E_Q = 3.13$ mm s^{-1}.

Magn. MB (solid, 2-20K): $\delta = -0.13$ mm s^{-1}; $\Delta E_Q = 3.16$ mm s^{-1}, $S = 1$, $D = 17.6$ cm^{-1}, $A/g_N\mu_N$ [xx, yy, zz] = −20.6, −24.6, −2.8 T.

UV-vis (solution, MeCN): λ_{max} [nm] (ε_{rel} [L mol^{-1}cm^{-1}]) = 408 (1130), 629 (140).

SQUID (solid): $S = 1$, $g_{av} = 2.00$, $D = 19.0$ cm^{-1}, $TIP = 390 \cdot 10^{-6}$ cm^3 mol^{-1}, $\chi_M T_{200K} = 1.00$ cm^3 K mol^{-1}.

IRPD: $\nu(Fe^{16}O) = 816$ cm^{-1}, $\nu(Fe^{18}O) = 777$ cm^{-1}.

Syntheses of $[L^{NHC}Fe(O)(S^tBu)](OTf)$ (5a) and $[L^{NHC}Fe(O)(SAda)](OTf)$ (5b)

$[L^{NHC}Fe(O)(S^tBu)](OTf)$ (**5a**): Complex $[L^{NHC}Fe(S^tBu)(MeCN)](OTf)$ (**2a**; 25.0 mg, 37 µmol, 1.0 eq.) was dissolved in 2 mL of dry and degassed MeCN and was cooled to −35 °C. Solid sPhIO (35.2 mg, 104 µmol, 2.8 eq.) was added and the reaction mixture was left reacting at −35 °C for 1 h. During the reaction the color of the solution changed from red to brown. Unreacted sPhIO was filtered away and the brown product **5a** was precipitated with cold diethyl ether (18.0 mg, 27 µmol, 74%).

MB (solid, 80 K): $\delta = -0.09$ mm s^{-1}; $\Delta E_Q = 2.21$ mm s^{-1}.

UV-vis (solution, MeCN): λ_{max} [nm] (ε_{rel} [L mol^{-1}cm^{-1}]) = 335 (5880), 442 (1700), 577 (660), 710 (190).

SQUID (solid): $S = 1$, $g_{av} = 2.01$, $D = 14.5$ cm^{-1}, $TIP = 900 \cdot 10^{-6}$ cm^3 mol^{-1}, $\chi_M T_{200K} = 1.01$ cm^3 K mol^{-1}.

IRPD: ν(Fe^{16}O) = 761 cm^{-1}, ν(Fe^{18}O) = 730 cm^{-1}.

[L^{NHC}Fe(O)(SAda)](OTf) (**5b**): this was obtained similarly by using [L^{NHC}Fe(SAda)(MeCN)](OTf) (**2b**; 20.0 mg, 26 µmol, 1.0 eq.) and solid sPhIO (25.1 mg, 74 µmol, 2.8 eq.). The product **5b** was isolated by precipitation with diethyl ether as brown powder (16.5 mg, 22 µmol, 85%). Crystals suitable for XRD analysis were obtained by layering of diethyl ether onto a PrCN solution of **5b** at −35 °C.

MB (solid, 80 K): $\delta = -0.09$ mm s^{-1}; $\Delta E_Q = 2.23$ mm s^{-1}.

Magn. MB (solid, 2-20K): $\delta = -0.06$ mm s^{-1}; $\Delta E_Q = 2.25$ mm s^{-1}, $S = 1$, $D = 18.4$ cm^{-1}, $A/g_N\mu_N$ [xx, yy, zz] = −24.9, −22.2, 3.2 T.

UV-vis (solution, MeCN): λ_{max} [nm] (ε_{rel} [L mol^{-1}cm^{-1}]) = 337 (5520), 447 (1300), 586 (580), 715 (340).

SQUID (solid): $S = 1$, $g_{av} = 1.66$, $D = 15.7$ cm^{-1}, $TIP = 520 \cdot 10^{-6}$ cm^3 mol^{-1}, $\chi_M T_{200K} = 0.68$ cm^3 K mol^{-1}.

IRPD: ν(Fe^{16}O) = 753 cm^{-1}, ν(Fe^{18}O) = 730 cm^{-1}.

3 Further Spectroscopic and Magnetic Data

For UV-vis monitoring of the formation of **5a** and **5b** at low temperatures (Figures 4, bottom, and Figure S1), NBu$_4$[IO$_4$] was used as oxidant. To that end, to a 0.25 mM MeCN solution of **2a** or **2b**, respectively, was added 1.0 eq. of NBu$_4$[IO$_4$] dissolved in MeCN, and the progress of the transformation was then monitored by UV-vis spectroscopy. Using sPhIO gave the same product spectra of **5a** and **5b** but required the use of DCM for dissolving sPhIO, which is case of the thiolato-ligated starting complexes **2a** and **2b** led to baseline shifts that hampered the identification of isosbestic points.

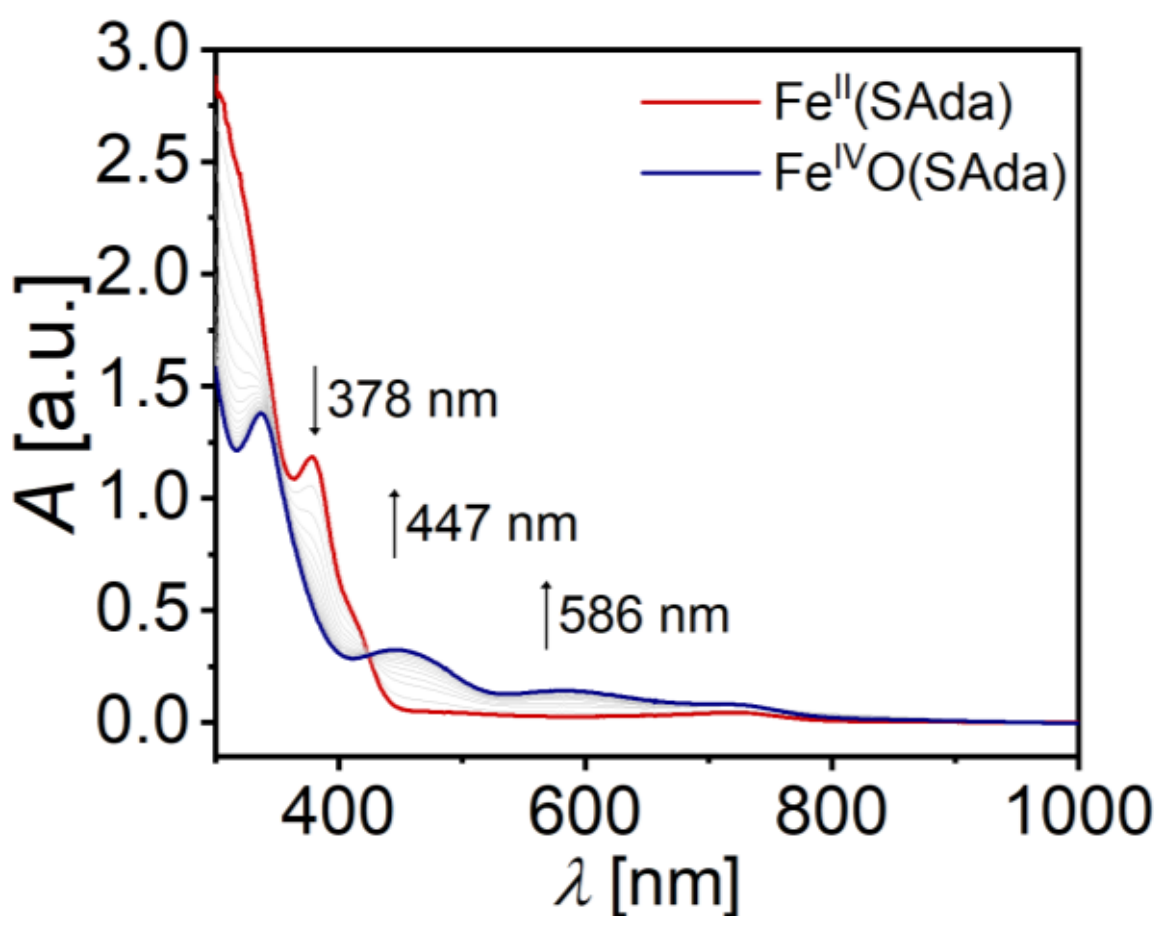

Figure S1. UV-Vis monitoring of the formation of complex **5b** (0.25 mM) starting from **2b** by addition of 1.0 eq. of NBu$_4$[IO$_4$] in MeCN at −35 °C.

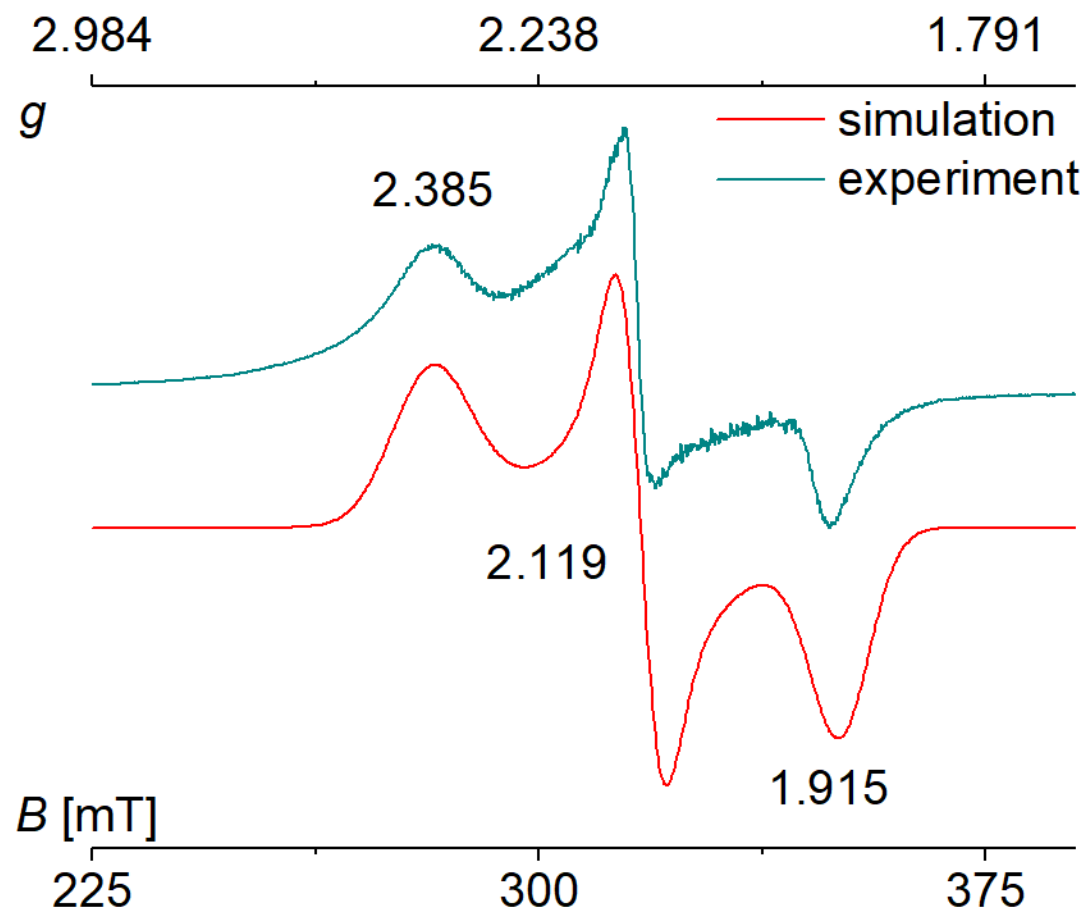

Figure S2. EPR spectrum of a solution of **5b** (6 mM) in PrCN after addition of 1.0 eq. of DHA at −40 °C and formation of the product with characteristic absorption at λ_{max} = 609 nm; the EPR spectrum was recorded on the frozen solution at 5 K and is assigned to the corresponding *S* = ½ hydroxidoiron(III) complex.

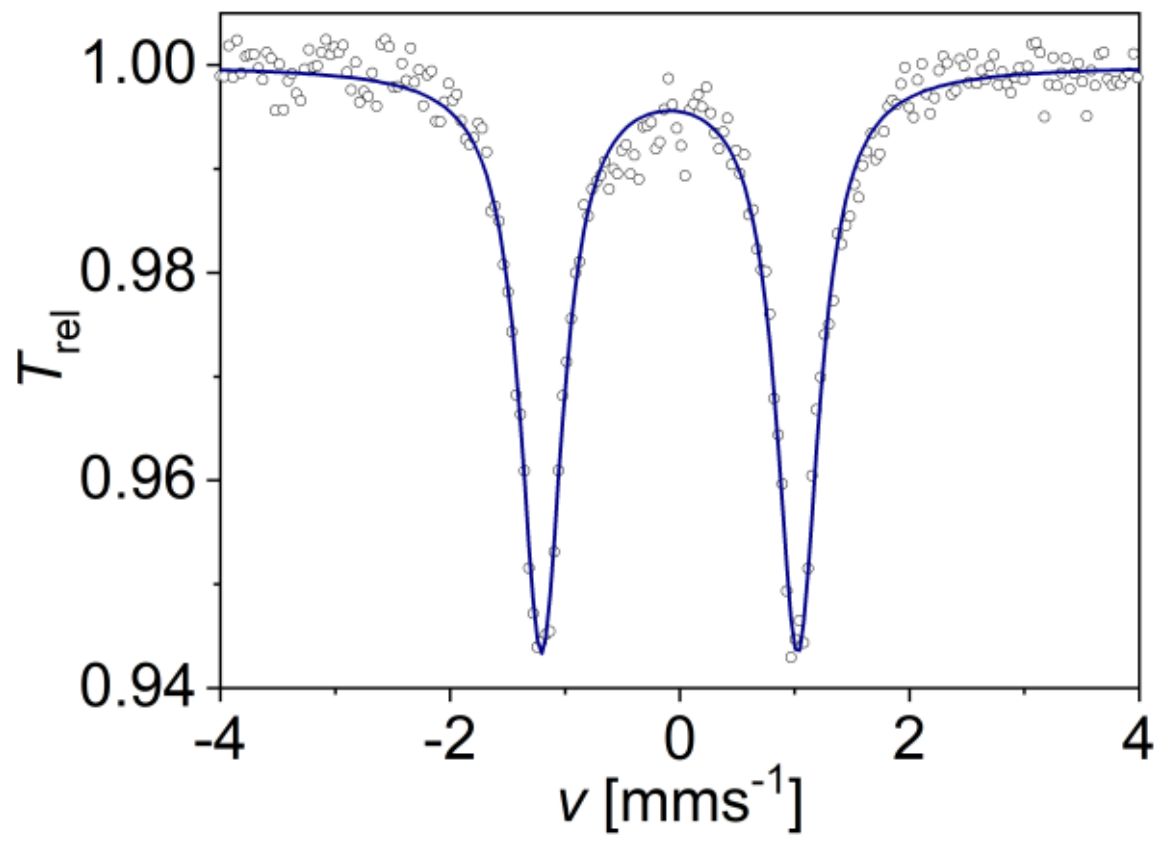

Figure S3. Zero-field ^{57}Fe Mössbauer spectrum of a solid sample of complex **5b** at 80 K.

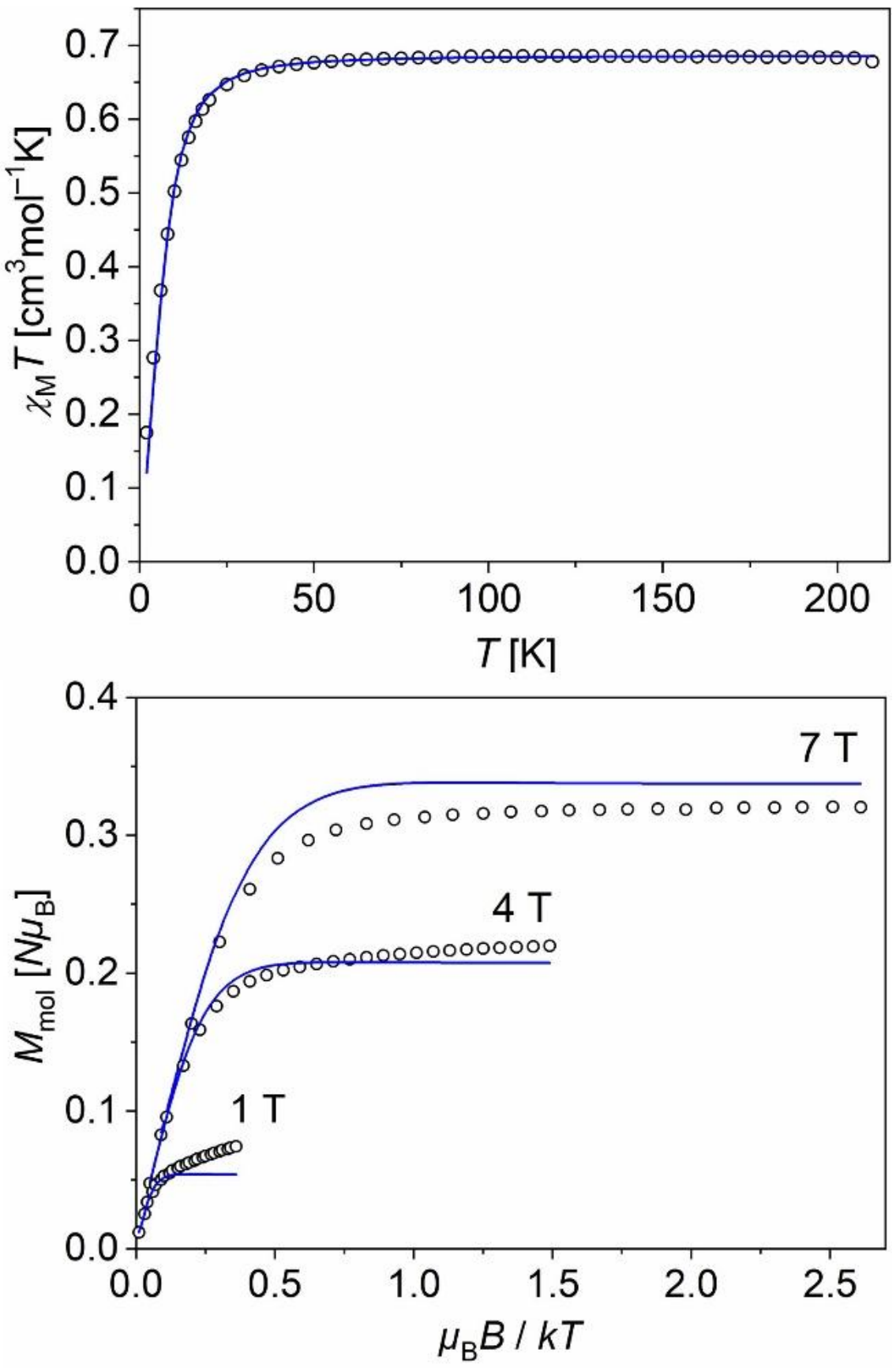

Figure S4. $\chi_M T$ vs T curve for a solid sample of complex **5b**. The empty circles represent the experimental data while the solid line is a simulation using equation 1 ($S = 1$, $g_{av} = 1.66$, $D = 15.7$ cm^{-1}). The somewhat low $\chi_M T$ and g_{av} values are probably caused by some diamagnetic impurity.

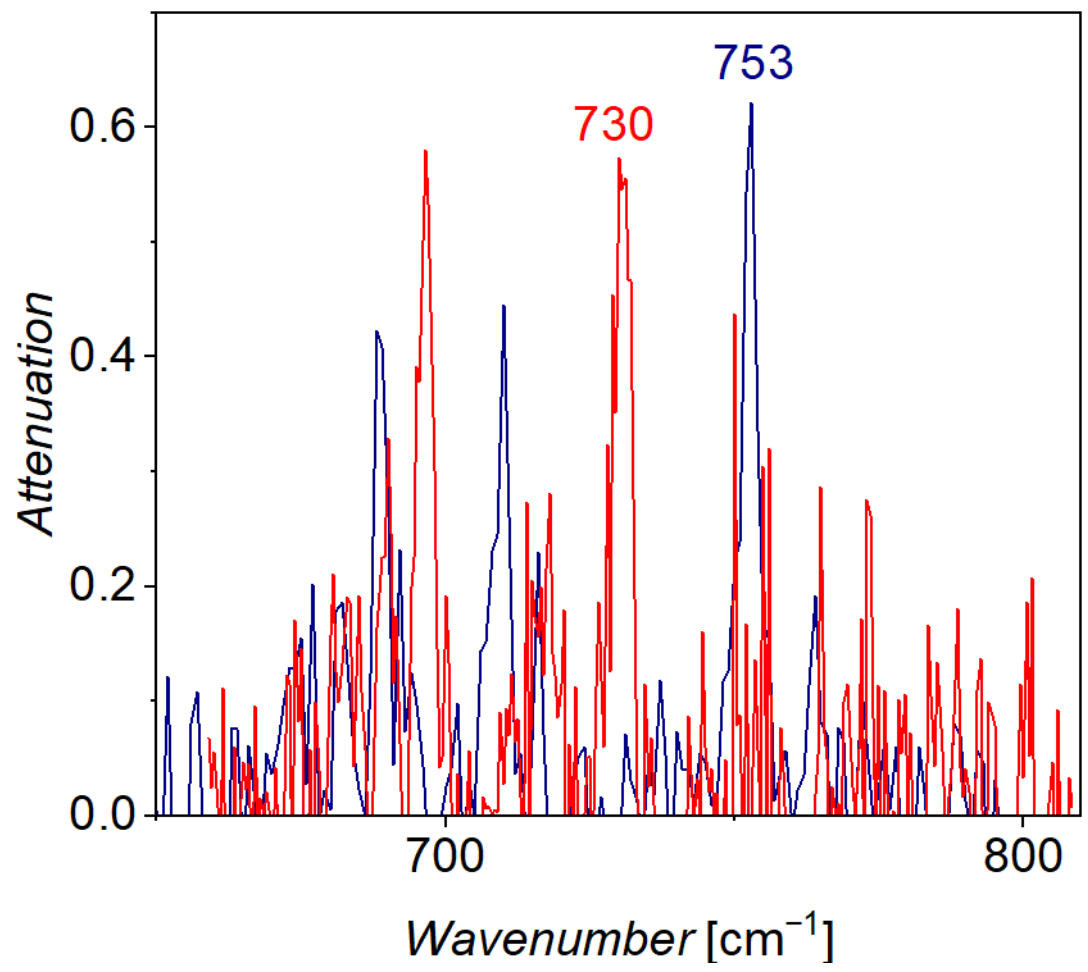

Figure S5. Helium tagging IRPD spectra of complex **5b**. The blue spectrum corresponds to naturally abundant [$L^{NHC}Fe(^{16}O)(SAda)]^+$, the red spectrum corresponds to the ^{18}O-labelled [$L^{NHC}Fe(^{18}O)(SAda)]^+$.

4 MCD Spectra

The S = 1 oxidoiron(IV) species have near-axial symmetry, which means that its MCD pattern would be quite different from those atom-like or cubic systems such as $[Fe^{III}(CN)_6]^{3-}$, and the orientation average plays an important role in its MCD spectra. If the Fe-O bond axis was chosen as the z-axis/direction, complexes **3**, **4**, and **5a** shows three characteristic ligand-field transitions, denoting as Fe-3d_{xy} to $\pi^*_{x/y}$ (Fe-$d_{xz,yz}$), from $\pi^*_{x/y}$ to Fe-3d_{x2-y2}, and from $\pi^*_{x/y}$ to Fe-3d_{z2}, respectively. These three transitions are well separated in the energy region and have negligible overlap, making the assignment rather simple and direct.

The transition of Fe-3d_{xy} to $\pi^*_{x/y}$ (Fe-$d_{xz,yz}$) is also referred to the intra-t_{2g} transition, which is strongly coupled to the vibration of the Fe-O stretch and shows clear Franck-Condon progression. The Franck-Condon progression simulations are shown in Figures S11-S13. Because $h\nu_{es}$, $S_{HR,}$ and the line width strongly correlate with each other, especially when the transitions are weak and the spectrum resolution is not sufficiently high; consequently, the error bar of the simulation is somewhat underestimated. Therefore, the difference between complexes **3** and **4** falls within the error bar, but the Franck-Condon progression frequency of them are clearly higher than complex **5a**.

The S = 1 ground state of complexes **3**, **4** and **5a** have three magnetic sublevels, namely, M_S = 0 and M_S = ±1, with a zero-field splitting of around 16 cm^{-1}. Even at an external field of 10 T, only the M_S = 0 gets substantially populated at 2 K. This magnetic sublevel has large transverse spin expectation values in the equatorial plane and elicits positive C-term signals for transitions of $\pi^*_{x/y}$ to Fe-3d_{x2-y2} and $\pi^*_{x/y}$ to Fe-3d_{z2}. Upon elevating the temperature, the M_S = ±1 levels gets gradually populated and gives different MCD signals compared to the M_S = 0 sublevel. Specifically, since the M_S = ±1 levels possess a large longitudinal spin expectation value along the z-axis, it shows intense MCD signals along this direction. More importantly, they show a positive and a negative pseudo-A term for the transitions of $\pi^*_{x/y}$ to Fe-3d_{x2-y2} and that of $\pi^*_{x/y}$ to Fe-3d_{z2}, respectively, due to the different symmetry of the electron accepting orbitals. As a result, the final MCD pattern of these two transitions is an overlap of a C-term feature and a pseudo-A term one. The pseudo-A term signal have a positive and a negative peak with almost equal intensities, while a pure C-term should register either a positive or a negative "Gaussian-like" peak. Consequently, the final MCD signal shows unequal areas for the positive and negative peaks, and the position of the maximum intensity varies with the temperature. As shown in Figure S6, the CASSCF/NEPVT2 calculations of a simplified model clearly show this sophisticated MCD behavior.

The MCD spectra show several new features when the ligand is switched to ^{t}BuS⁻(**5a**), the sulfur may induce some sulfur-to-iron charge transfer character in transitions of $\pi^*_{x/y}$ to Fe-3d_{z2} and $\pi^*_{x/y}$ to Fe-3d_{x2-y2}, mixing some z-polarized transition dipole moment. The MCD intensity along one direction is proportional to the product of spin expectation value and the transition dipole moment product of two other perpendicular directions. For example, the MCD intensity along x direction is proportional to $\langle S_x \rangle \cdot \mu_y \cdot \mu_z$, where $\langle S_x \rangle$ is the spin expectation value along x direction and μ_i is the transition dipole moment along i direction. As a consequence, the increase of the z-component in the transition dipole moment would intensify the positive C-term signal situated in the xy-plane. Therefore, the negative signal in pseudo-A at high temperature is buried in the strong positive C-term signals, as shown in Figure S6, the bumps replaced the negative signals.

One should be aware that, the accurate transition energy of such an electronic state transition (for example, the transition from $\pi^*_{x/y}$ to Fe-3d_{z2}) is difficult to accurately “define” on the experimental MCD spectra. The excited states have orbital near-degeneracy and therefore strong spin-orbit coupling, resulting in a series of magnetic sublevels with the energy variance of around 2000 to 3000 cm^{-1} (e.g. the energy difference of the positive and negative peaks of the pseudo-*A* term). Therefore, we superimposed the normalized MCD spectra of complexes **3**, **4**, and **5a** recorded at 20 K, and estimated the energy shift by measuring the peak position shift as shown in Figure 10.

We also show the superimposed UV-Vis spectra of complexes **3**, **4**, and **5a** in Figure S7. One may notice that the $\pi^*_{x/y}$ to Fe-3d_{x2-y2} transition seems to show a different order compared to the MCD spectra. This is understandable because this transition strongly overlaps with the ligand charge-transfer on the ABS spectrum. Due to the higher resolved MCD bands on the MCD spectra, the energy level shift should be best discussed based on the MCD spectra.

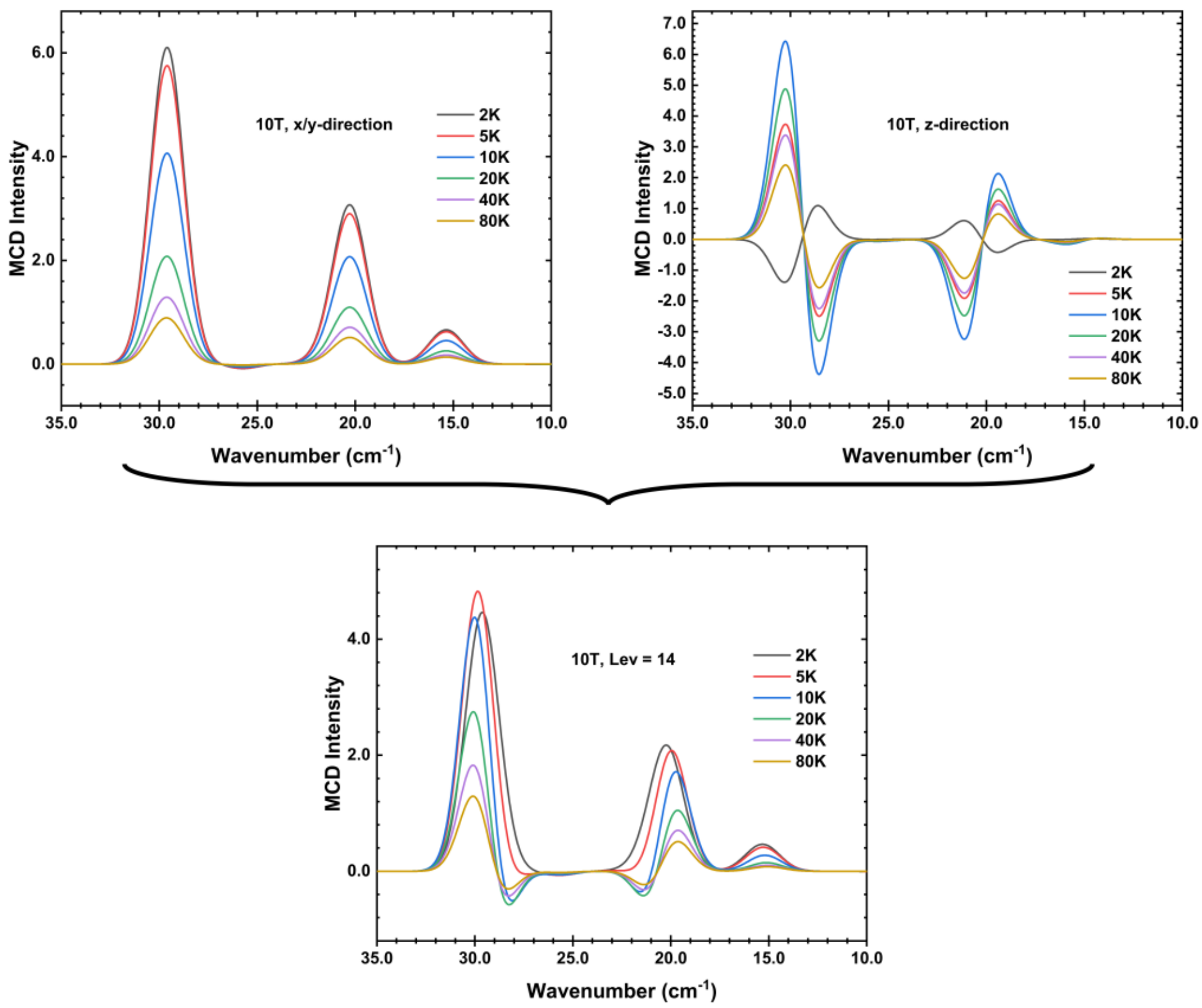

Figure S6. The calculated angular-dependent MCD spectrum of the simplified oxidoiron(IV) model at 10 T. (above) MCD spectra along x/y and z-direction. (below) The orientation averaged spectrum.

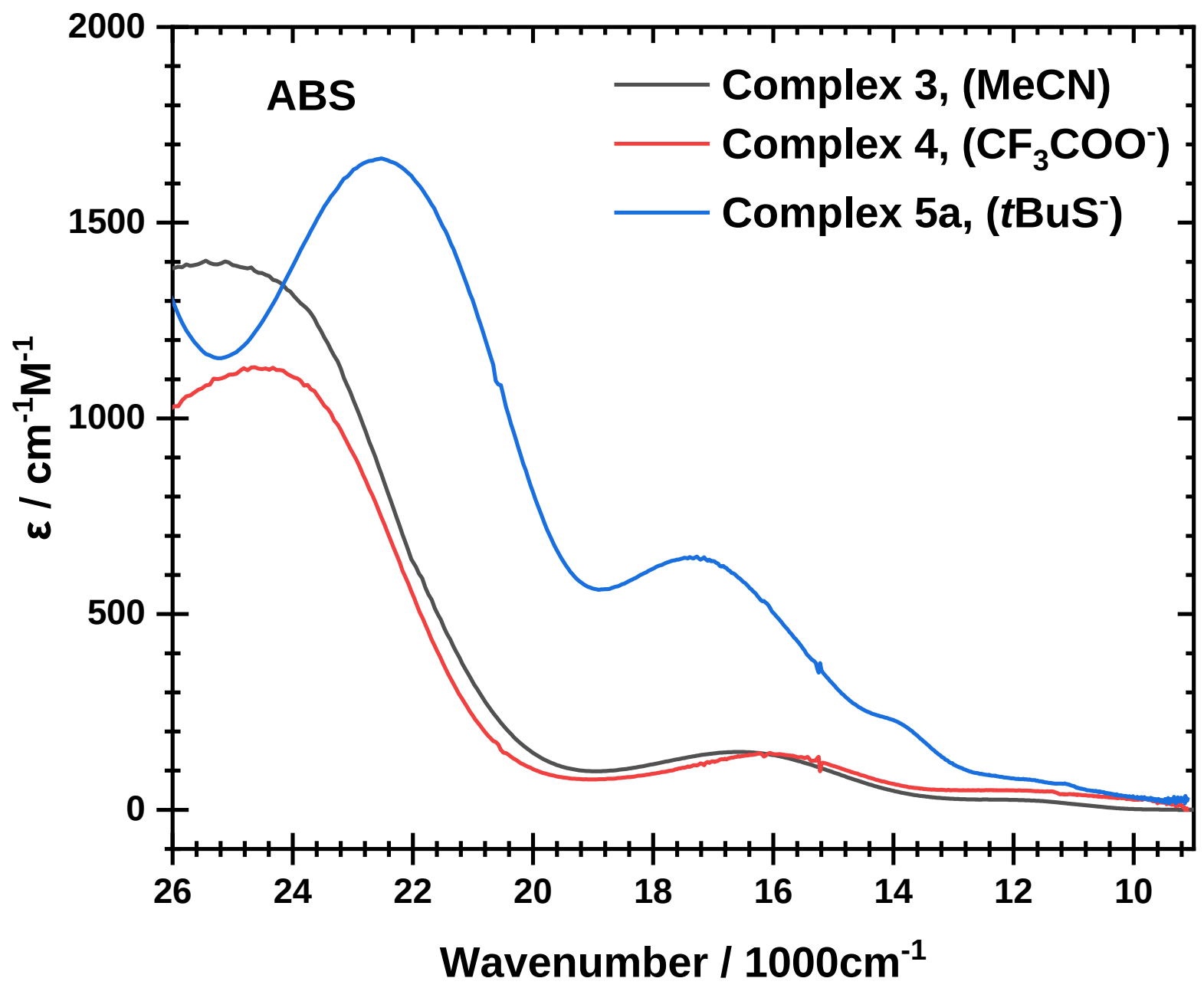

Figure S7. Overlay of the absorption spectra of complexes **3**, **4**, and **5a**.

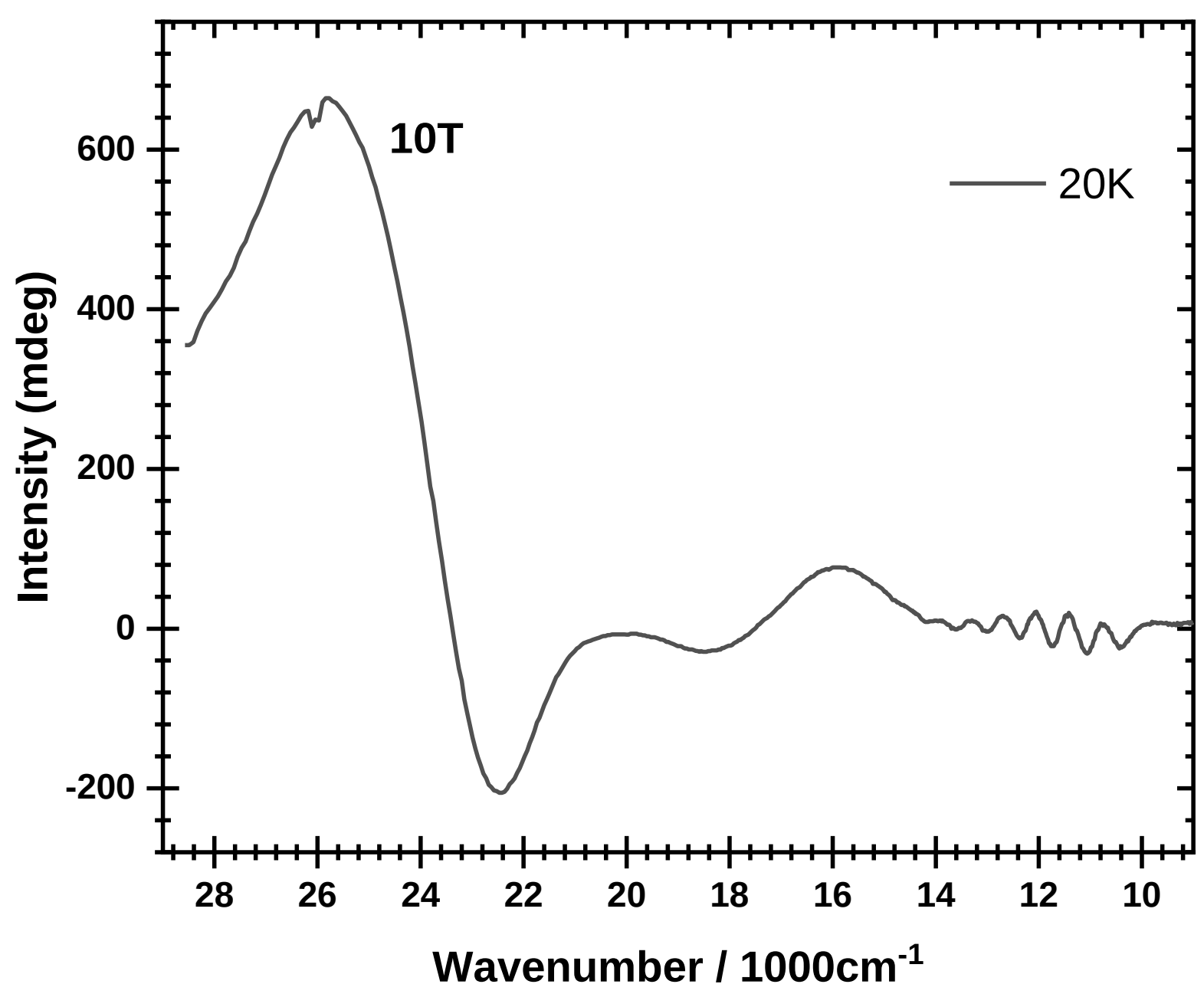

Figure S8. The MCD spectrum of [$L^{NHC}Fe^{IV}(O)(NCMe)](OTf)_2$ (**3**) at 10 T and 20 K.

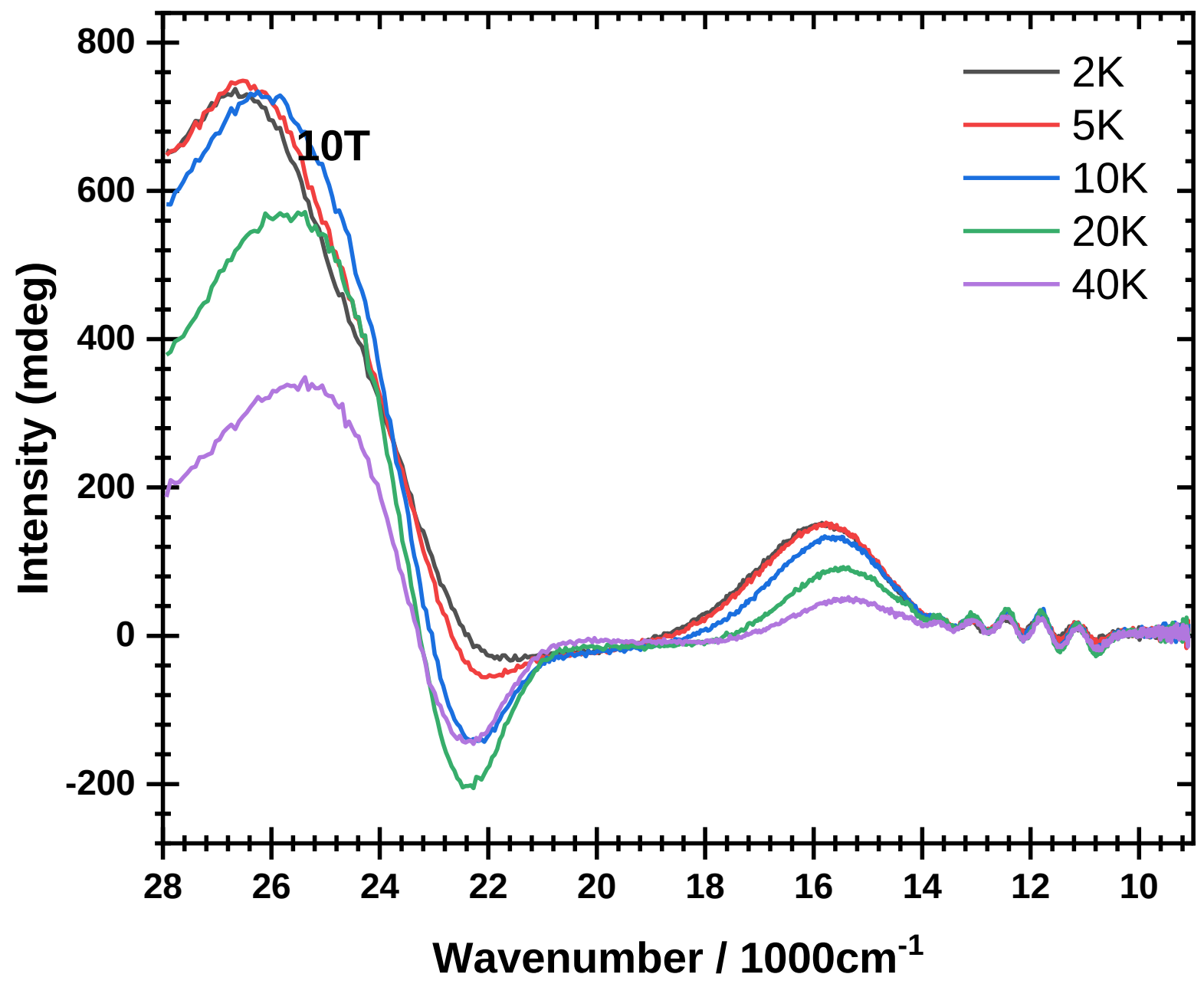

Figure S9. The VTVH-MCD spectrum of [$L^{NHC}Fe^{IV}(O)(OOCCF_3)$]OTf (**4**).

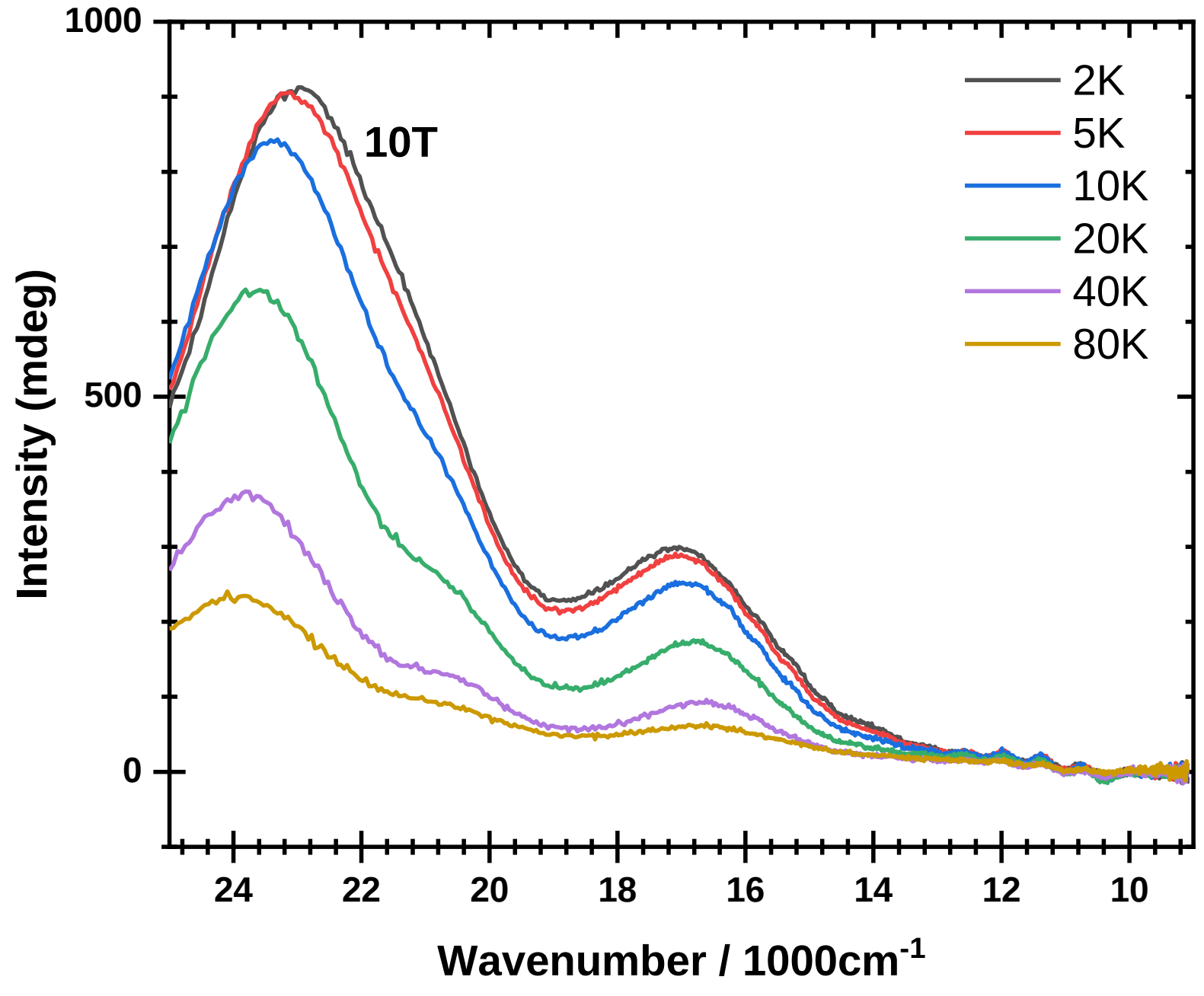

Figure S10. The VTVH-MCD spectrum of [$L^{NHC}Fe^{IV}(O)(S^{t}Bu)$]OTf (**5a**).

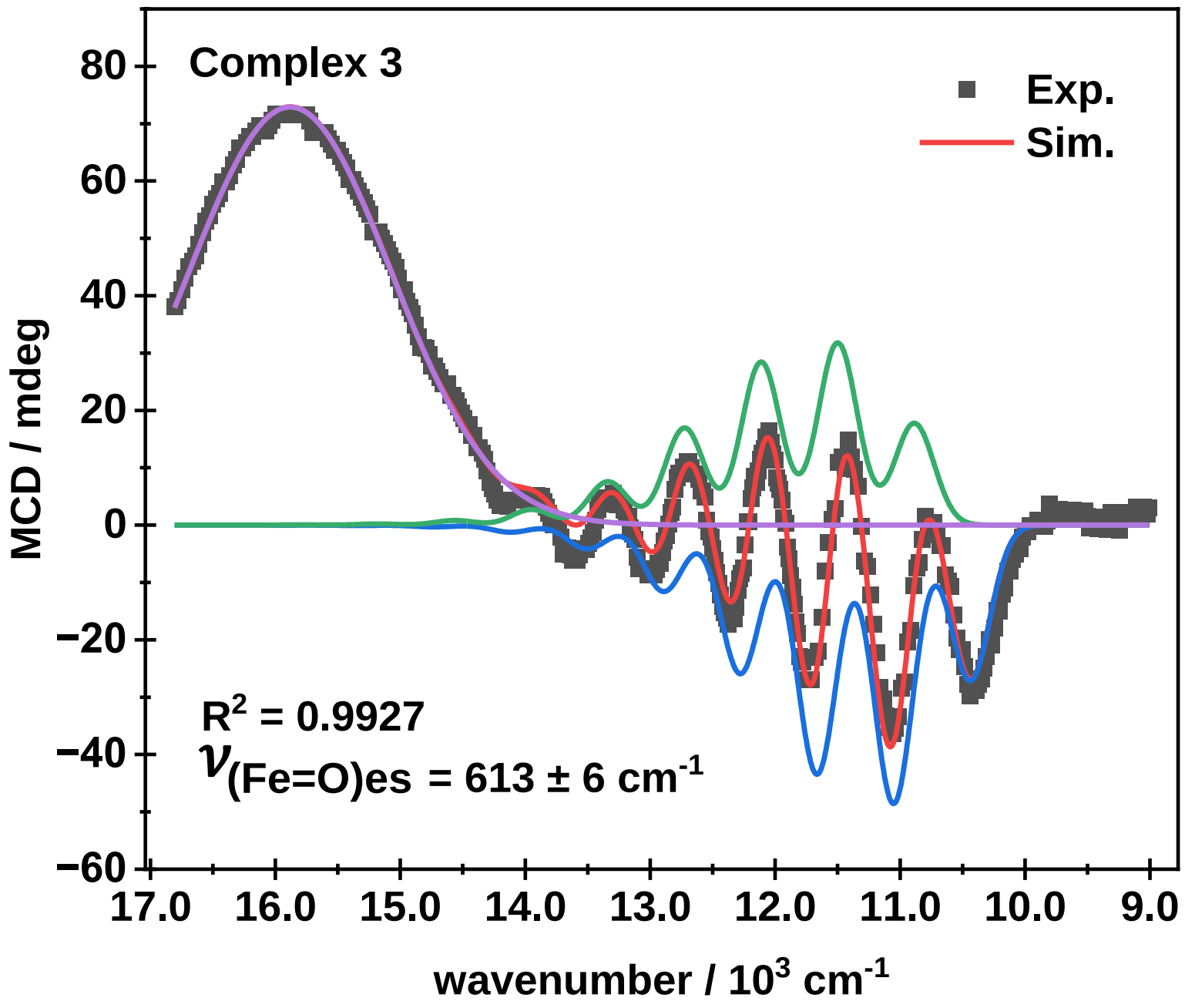

Figure S11. The Franck-Condon progression simulation of $[L^{NHC}Fe^{IV}(O)(NCMe)](OTf)_2$ (**3**).

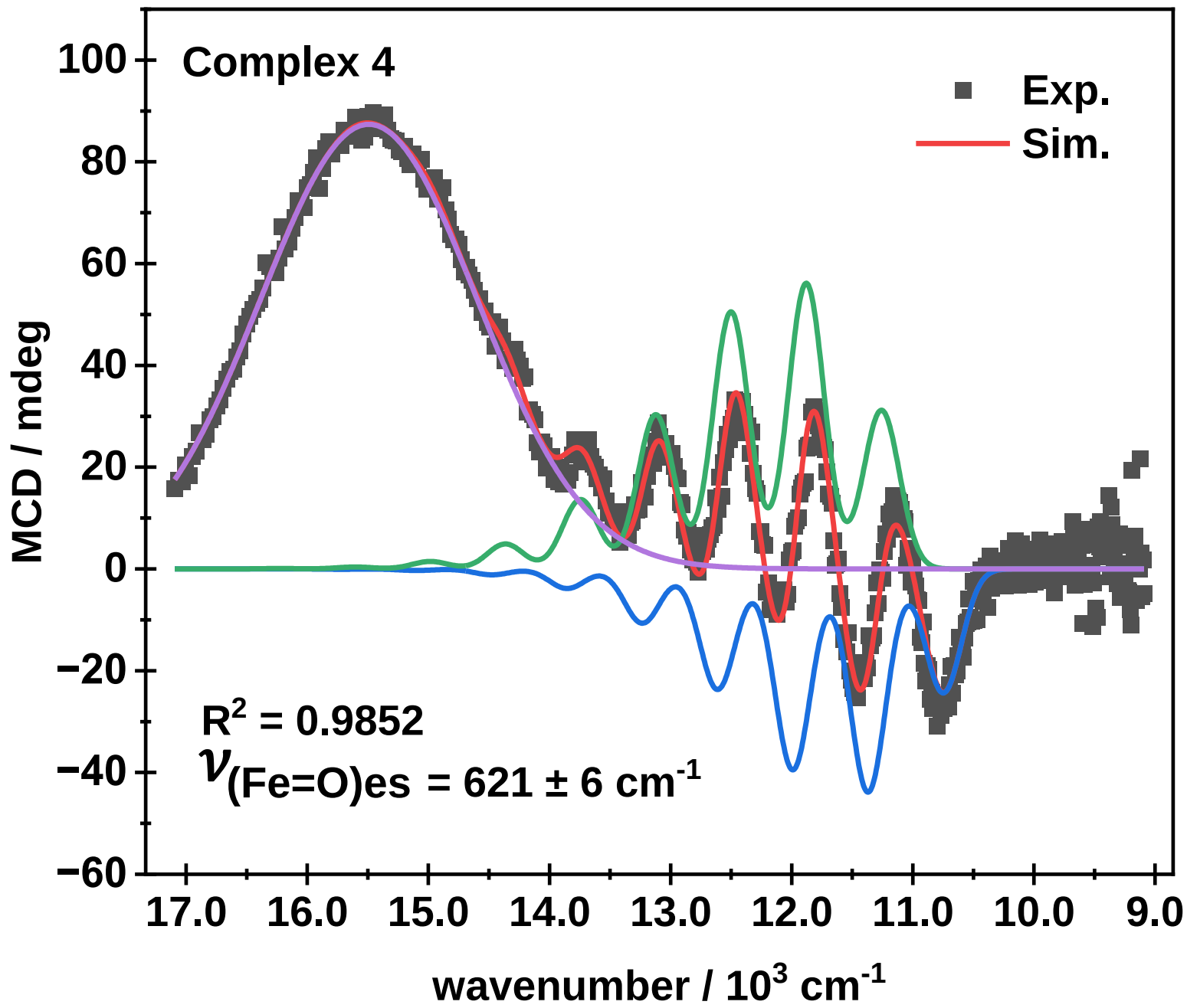

Figure S12. The Franck-Condon progression simulation of $[L^{NHC}Fe^{IV}(O)(OOCCF_3)]OTf$ (**4**).

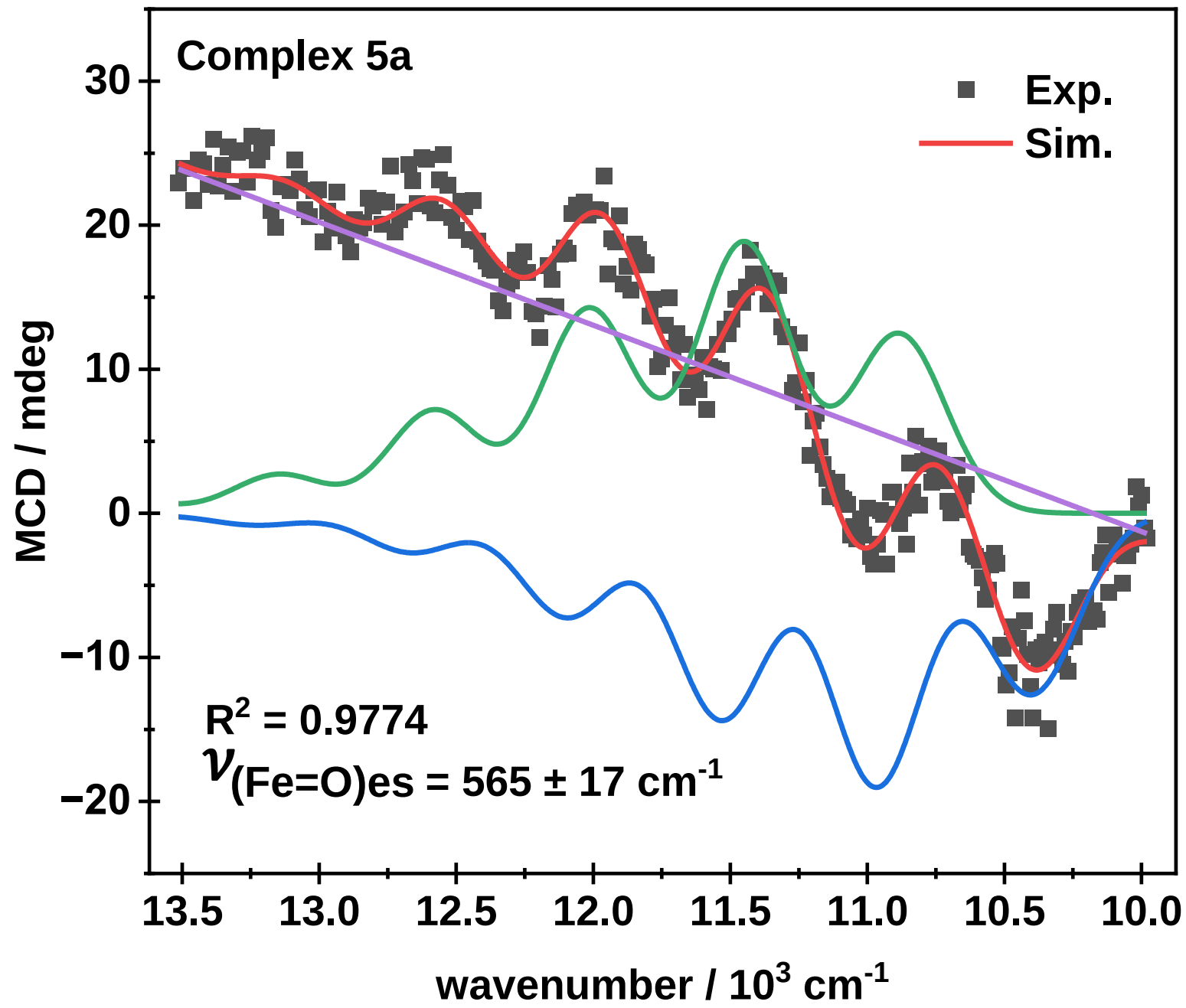

Figure S13. The Franck-Condon progression simulation of [$L^{NHC}Fe^{IV}(O)(S^{t}Bu)$]OTf (**5a**). Owing to the spectral overlap with the assigned charge transfer transition from thiolate to iron center, the background in the region shown should be characterized with several broad Gaussian peaks. To avoid over parameterization, a linear background was employed (purple line).

5 X-Ray Crystallography

X-ray data were collected on a STOE IPDS II diffractometer (graphite monochromated Mo-Kα radiation, λ = 0.71073 Å) by use of scans at –140 °C (Table S1). The structure was solved with SHELXT[8] and refined on F^2 using all reflections with SHELXL.[9] Non-hydrogen atoms were refined anisotropically. Hydrogen atoms were placed in calculated positions and assigned to an isotropic displacement parameter of 1.2 / 1.5 U_{eq}(C). Face-indexed absorption corrections were performed numerically with the program X-RED.[10] The overall molecular structure of **5b** could be established (SG: $P2_1/n$, Z = 4, a = 20.47(4) Å, b = 8.51(2) Å, c = 24.40(5) Å, β = 111.1(1)°, V = 3966(15) Å^3); however, the data quality of the diffraction experiment was not sufficient to allow a meaningful discussion of parameters such as bond lengths or angles.

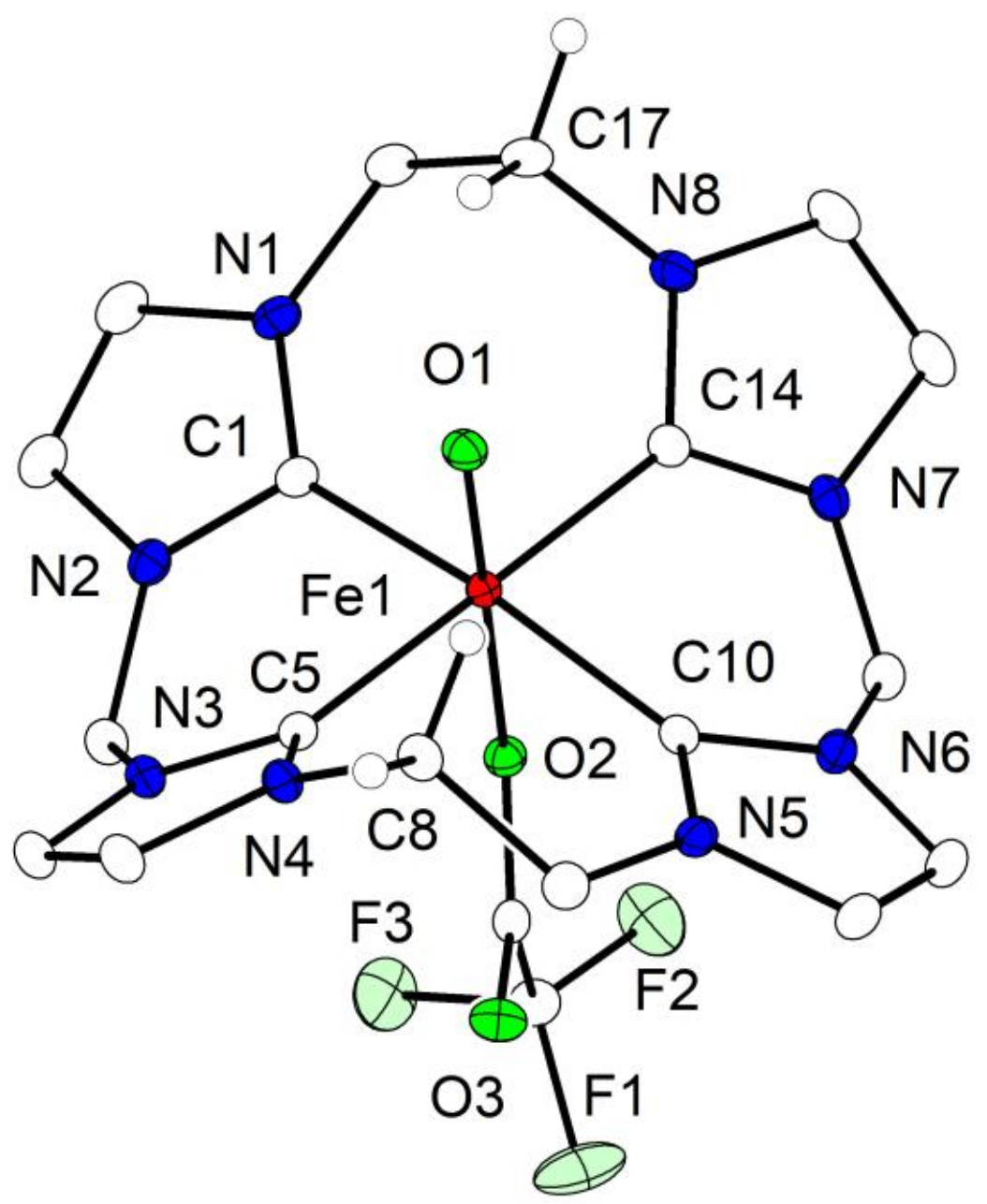

Figure S14. Plot (30% probability thermal ellipsoids) of the molecular structure of the cationic part of **4** (most hydrogen atoms omitted for clarity). Selected bond lengths [Å] and angles [°] for **4**: Fe1-O1 1.6714(10), Fe1-C5 1.9704(15), Fe1-C14 1.9798(15), Fe1-C1 2.0244(15), Fe1-C10 2.0376(15), Fe1-O2 2.0686(10), C8···O1 2.992(2), C17···O1 2.993(2), H8B···O12.2506(13), H17B···O1 2.2443(1); O1-Fe1-C5 89.20(6), O1-Fe1-C14 91.02(6), C5-Fe1-C14 179.40(6), O1-Fe1-C1 94.95(6), C5-Fe1-C1 86.79(6), C14-Fe1-C1 92.64(6), O1-Fe1-C10 97.11(5), C5-Fe1-C10 94.28(6), C14-Fe1-C10 86.24(6), C1-Fe1-C10 167.90(6), O1-Fe1-O2 178.50(5), C5-Fe1-O2 91.34(5), C14-Fe1-O2 88.43(5), C1-Fe1-O2 83.69(5), C10-Fe1-O2 84.24(5), C8-H8B···O1 130.7(1), C17-H17B···O1 131.4(1).

Table S1. Crystal data and refinement details for **4**

**compound**	**4 (mm22)**
empirical formula	$C_{23}H_{23}F_6FeN_9O_6S$
formula weight	723.41
T [K]	133(2)
crystal size [mm³]	0.50 x 0.49 x 0.34
crystal system	monoclinic
space group	*C*2/*c* (No. 15)
a [Å]	26.8979(8)
b [Å]	11.7811(4)
c [Å]	19.4343(5)
β [°]	115.136(2)
V [Å³]	5575.3(3)
Z	8
ρ [g·cm⁻³]	1.724
F(000)	2944
μ [mm⁻¹]	0.715
T_{min} / T_{max}	0.6860 / 0.8077
θ-range [°]	1.673 - 26.793
hkl-range	±34, ±14, –24 to 21
measured refl.	38833
unique refl. [R_{int}]	5915 [0.0266]
observed refl. ($I > 2\sigma(I)$)	5240
data / restr. / param.	5915 / 0 / 416
goodness-of-fit (F^2)	1.039
*R*1, *wR*2 ($I > 2\sigma(I)$)	0.0276 / 0.0722
*R*1, *wR*2 (all data)	0.0327 / 0.0751
res. el. dens. [e·Å⁻³]	–0.327 / 0.539

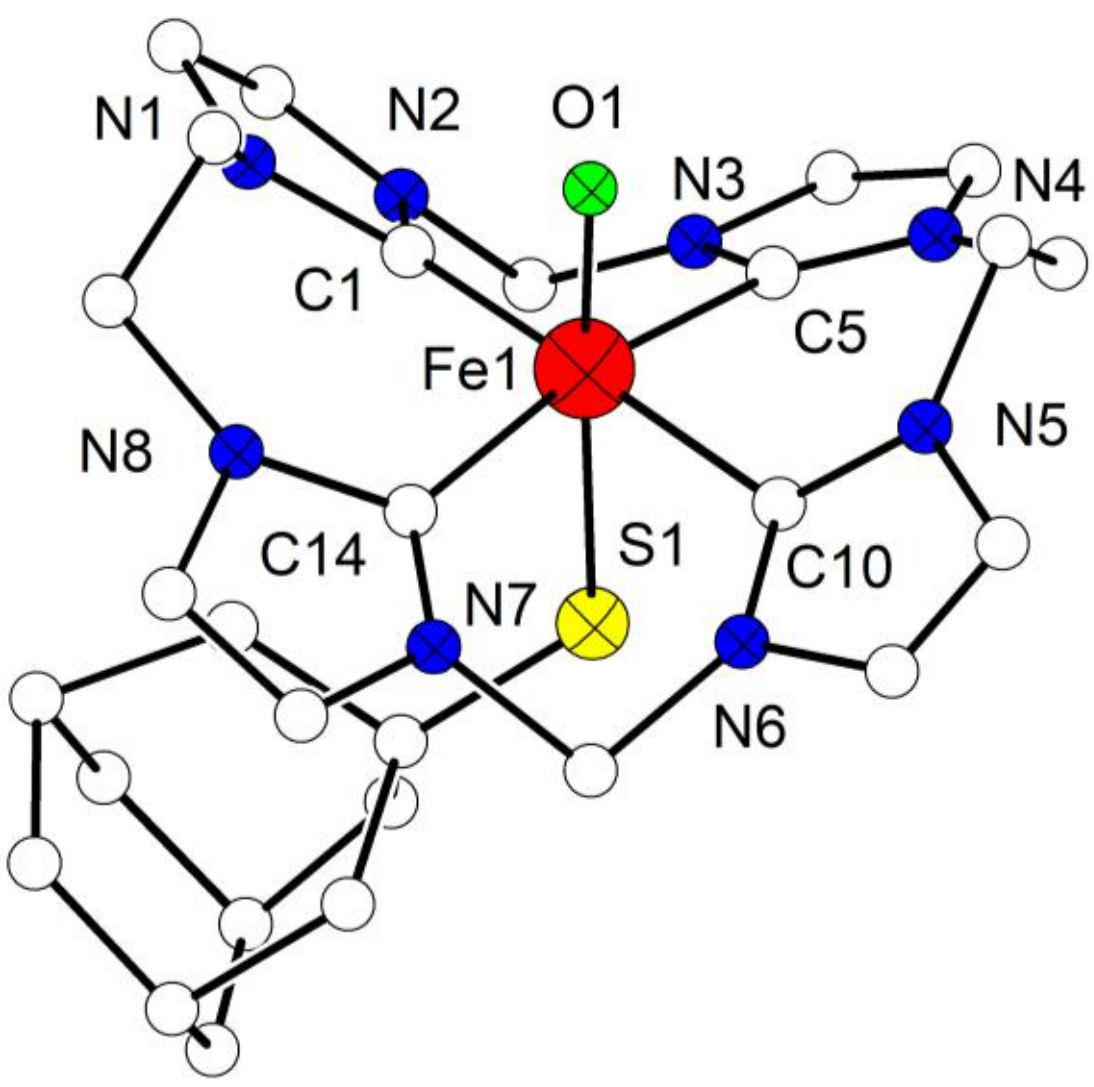

Figure S15. Plot of the molecular structure of the cationic part of **5b**.

6 Further Kinetic Data and Product Quantification

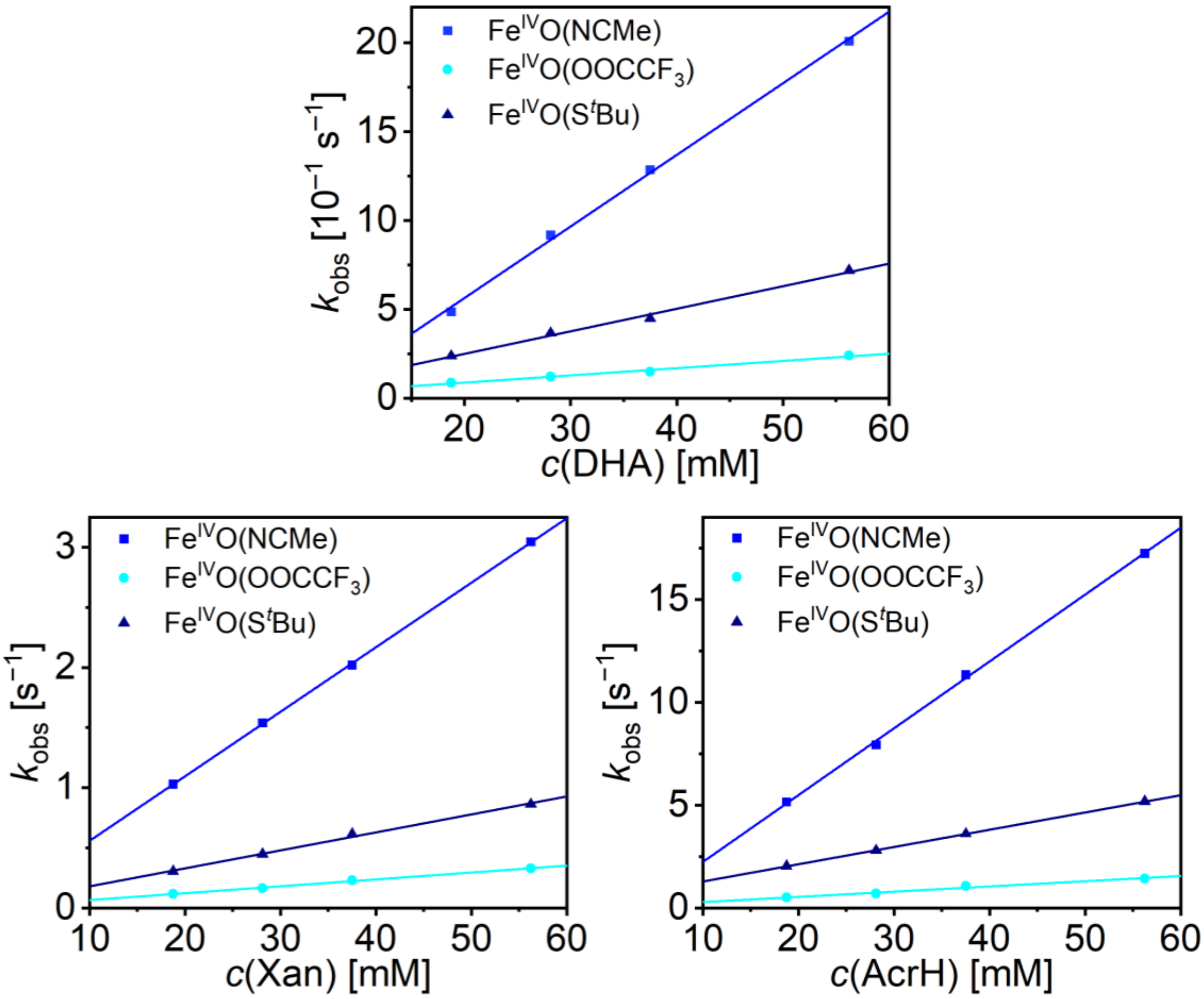

Figure S16. Kinetic data of the reaction of complexes **3**, **4** and **5a** with the different substrates DHA (top), Xan (bottom, left) and AcrH (bottom, right) at 25 °C in MeCN/DCM mixtures.

Table S2. Kinetic rates k_2' of the reaction of complexes **3**, **4** and **5a** with the different substrates CHD, DHA, Xan and AcrH at 25 °C in MeCN/DCM mixtures.

	**3**	**4**	**5a**
k_2'(CHD) [M^{-1} s^{-1}]	6.3	0.6	0.9
k_2'(DHA) [M^{-1} s^{-1}]	10.0	1.0	3.2
k_2'(Xan) [M^{-1} s^{-1}]	26.8	2.9	7.5
k_2'(AcrH) [M^{-1} s^{-1}]	162.5	12.7	42.0

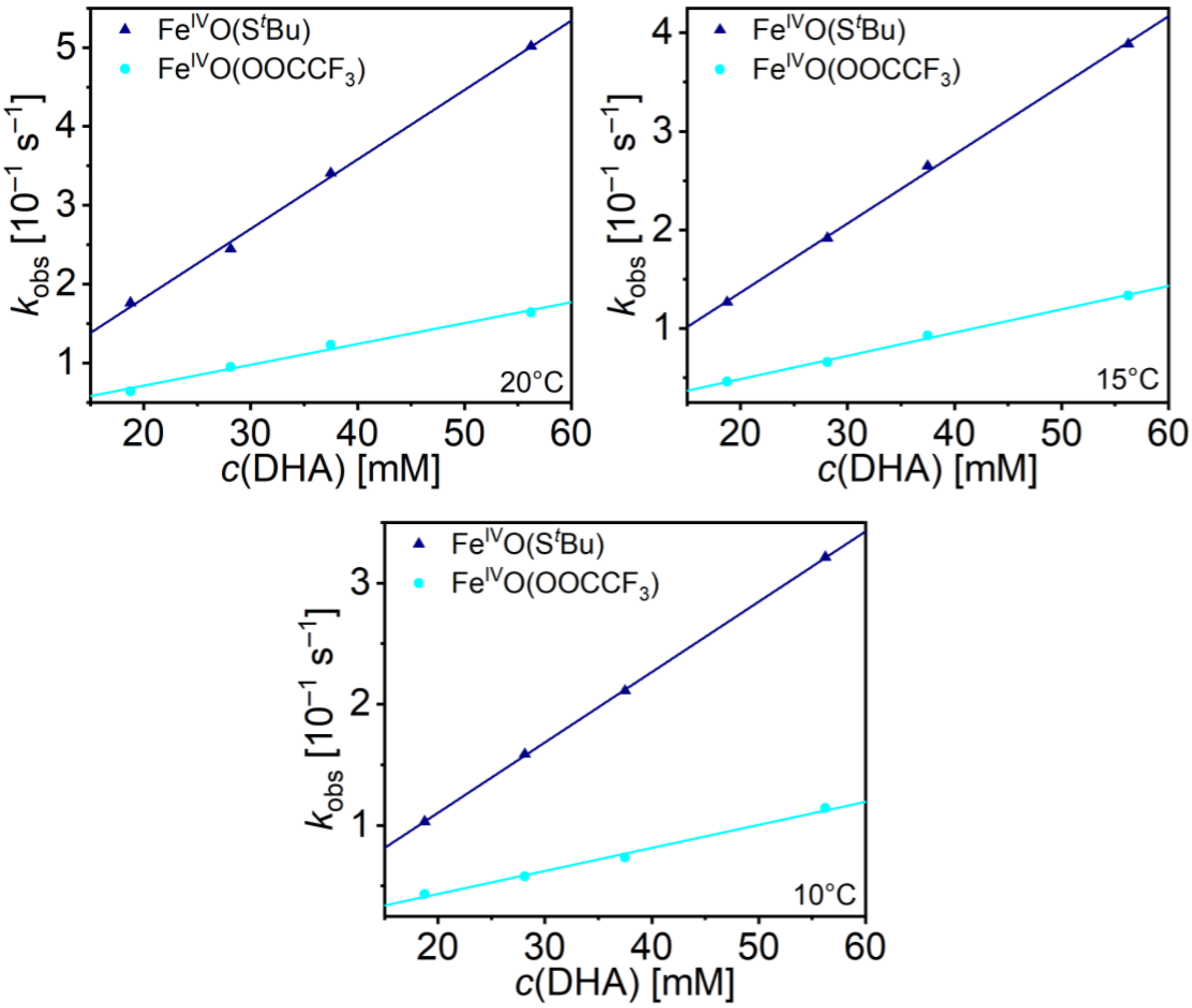

Figure S17. Kinetic data of the reaction of complexes **4** and **5a** with DHA in MeCN/DCM mixtures at different temperatures: 20 °C (top, left), 15 °C (top, right) and 10 °C bottom).

Table S3. Kinetic rates k_2' of the reaction of complexes **4**, and **5a** with DHA at different temperatures in MeCN/DCM mixtures.

	**4**	**5a**
k_2'(DHA) [M^{-1} s^{-1}], 20 °C	0.7	2.2
k_2'(DHA) [M^{-1} s^{-1}],15 °C	0.6	1.8
k_2'(DHA) [M^{-1} s^{-1}], 10 °C	0.5	1.5

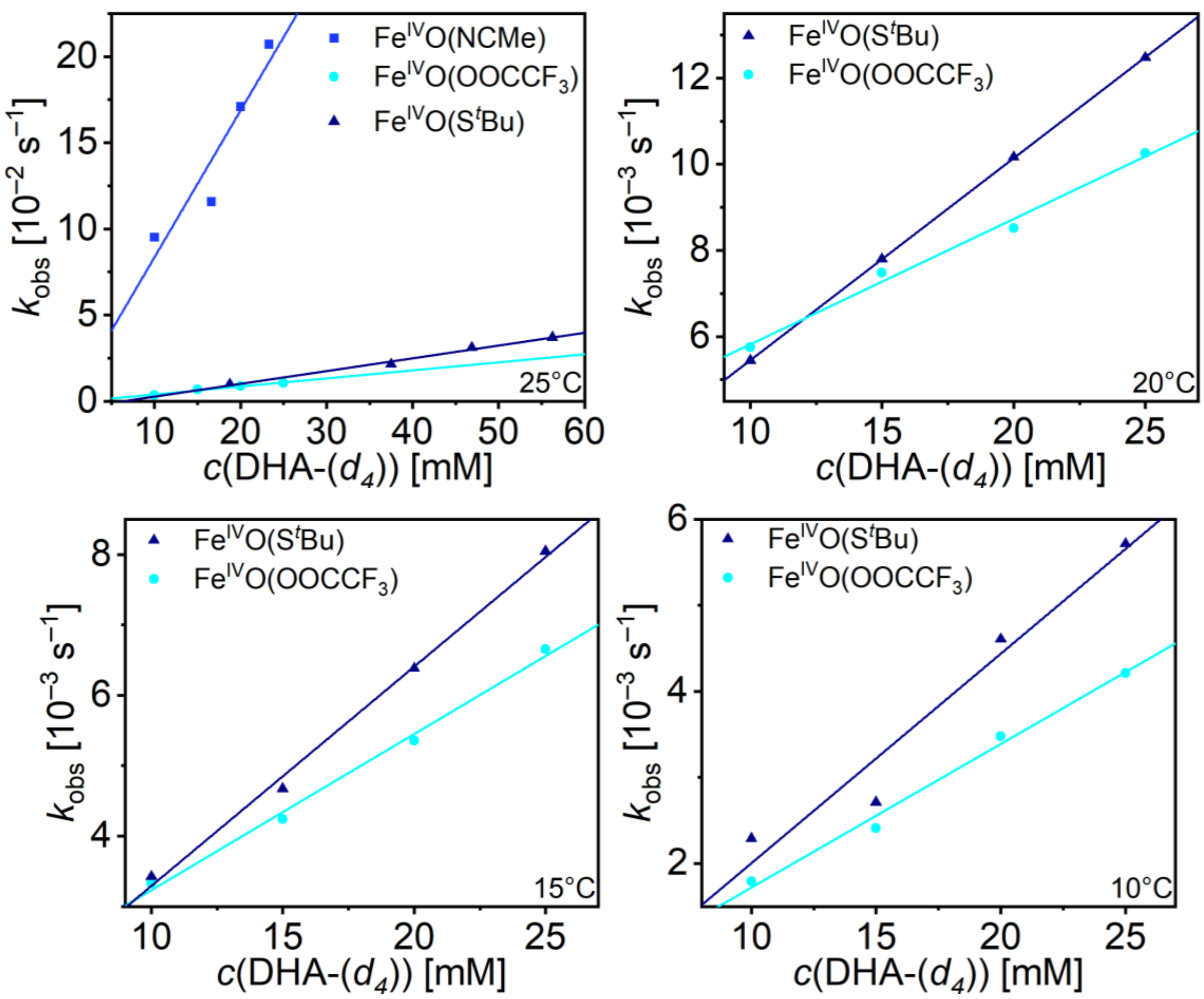

Figure S18. Kinetic data of the reaction of complexes **4** and **5a** with DHA-d_4 in MeCN/DCM mixtures at different temperatures: 25 °C (top, left), 20 °C (top, right), 15 °C (bottom, left) and 10 °C (bottom, right).

Table S4. Kinetic rates k_2' of the reaction of axially ligated complexes **3**, **4** and **5a** with DHA-d_4 at different temperatures in MeCN/DCM mixtures.

	**3**	**4**	**5a**
k_2'(DHA-d_4) [M^{-1} s^{-1}], 25 °C	2.13	0.12	0.19
k_2'(DHA-d_4) [M^{-1} s^{-1}], 20 °C	-	0.07	0.11
k_2'(DHA-d_4) [M^{-1} s^{-1}],15 °C	-	0.06	0.08
k_2'(DHA-d_4) [M^{-1} s^{-1}], 10 °C	-	0.04	0.06

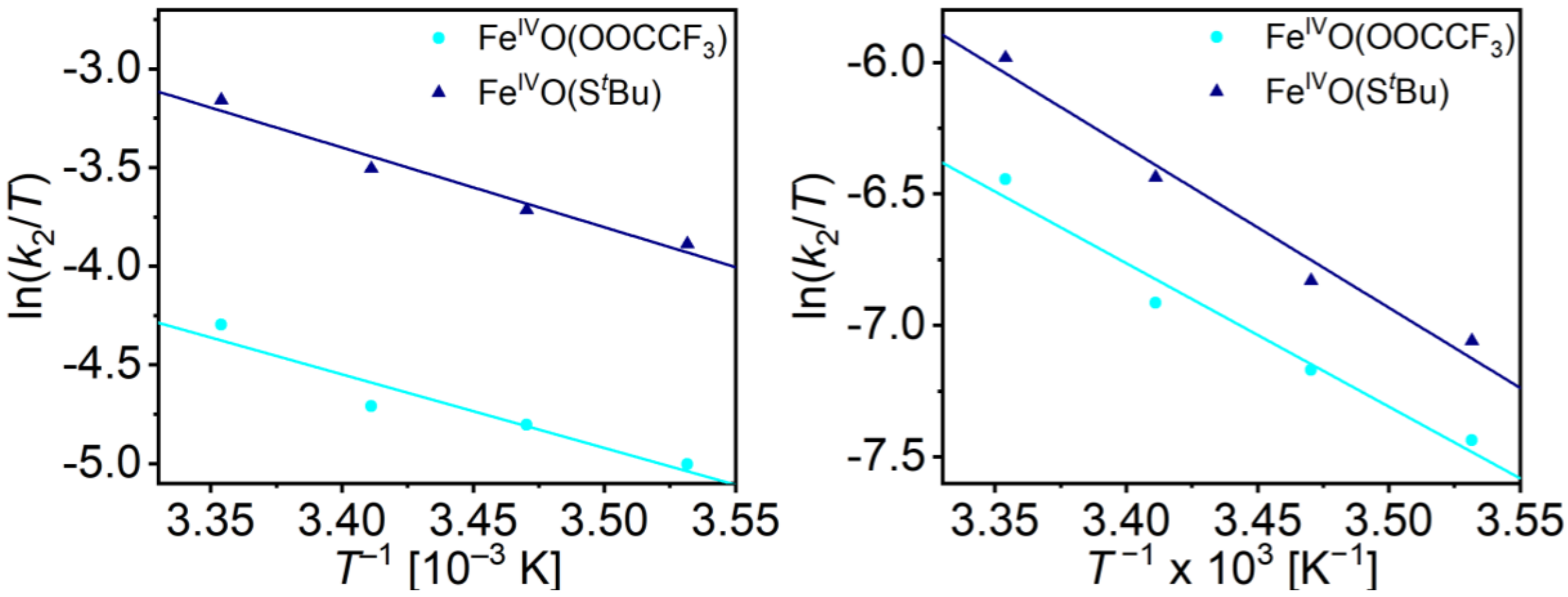

Figure S19. Eyring plot of the reaction of complexes **4** and **5a** with DHA (left) and DHA-d_4 (right).

Table S5. Eyring parameter of **4** and **5a**.

	**5a**, non-deuterated	**5a**, deuterated	**4**, non-deuterated	**4**, deuterated
$\Delta H^{‡}$ [kcal mol^{-1}]	8.0	12.4	7.4	11.8
$\Delta S^{‡}$ [cal K mol^{-1}]	–26.7	–17.7	–31.0	–23.8
$\Delta G^{‡}_{20°C}$ [kcal mol^{-1}]	15.8	17.6	16.5	17.8

For the extrapolation of the KIE values of **4** and **5a** to −40°C and calculation of the uncertainties, ordinary least squares (OLS) linear regressions were performed using a Python script with the statsmodels.api module (version 0.14.6).[11]

The linear regression yielded:

4: ln(KIE) = −3.31(±0.80) + 3.23(±0.46) · $(RT)^{-1}$; R^2 = 0.961

5a: ln(KIE) = −3.78(±0.96) + 3.92(±0.55) · $(RT)^{-1}$; R^2 = 0.962

Extrapolation to −40°C yielded:

4: ln(KIE) = 3.671 (±0.196)

5a: ln(KIE) = 4.688 (±0.237)

which corresponds to KIE = 39 (± 9) for **4** and KIE = 109 (± 29) for **5a**; the error represents the approximate standard error. Since only four data points measured over a narrow temperature range (10 to 25°C) are extrapolated over 65°C to −40°C, these standard errors probably underestimate the true uncertainty. The 95% prediction interval for KIE is therefore rather high, 17 - 92 for **4** and 39 - 304 for **5a** (75% PI for comparison: 29 – 54 for **4** and 74 - 160 for **5a**).

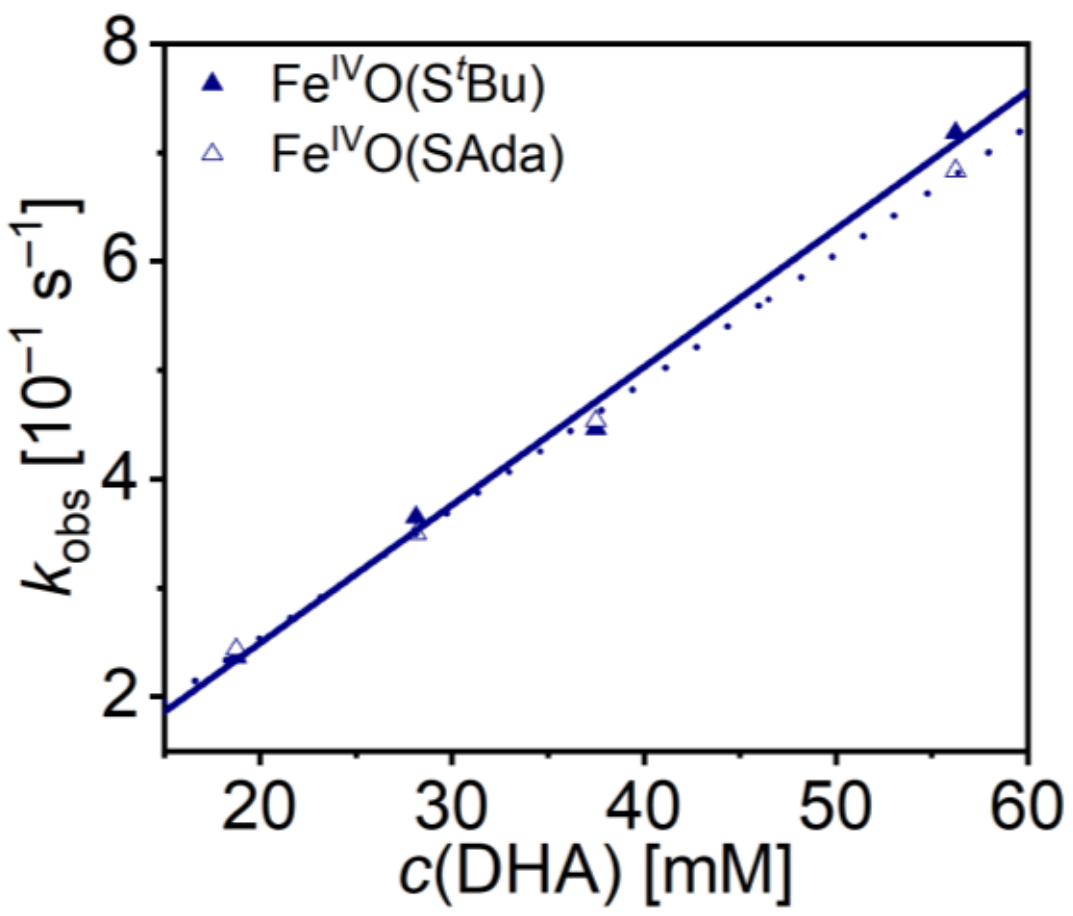

Figure S20. Comparison of the kinetic data of the reaction of complexes **5a** and **5b** with DHA at 25 °C in MeCN/DCM mixtures.

Table S6. Kinetic rates k_2’ of the reaction of axially ligated complexes **5a** and **5b** with DHA.

	**5a**	**5b**
k_2’(DHA) [M^{-1} s^{-1}], 25 °C	3.2	2.9

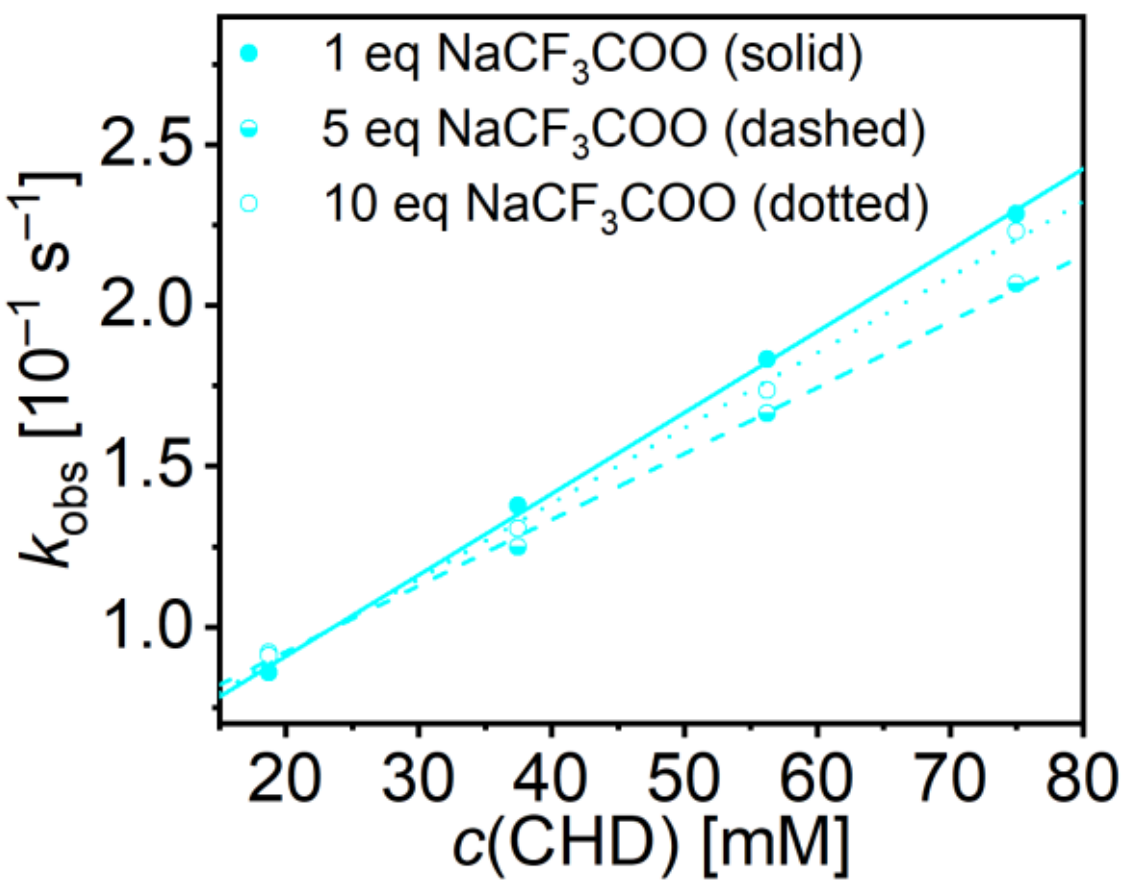

Figure S21. Comparison of the kinetic data of the reaction of complex **4** with different equivalents of added $NaCF_3COO$ with CHD at 25 °C in MeCN.

Table S7. Kinetic rates k_2’ of the reaction of complex **4** with CHD with different amounts of $NaCF_3COO$ added.

	**4** + 1 eq $NaCF_3COO$	**4** + 5 eq $NaCF_3COO$	**4** + 10 eq $NaCF_3COO$
k_2’(CHD) [M^{-1} s^{-1}], 25 °C	0.6	0.5	0.6

Product quantification:

Product analysis for the oxidation of DHA and CHD was performed by gas chromatography. Therefore, crystalline material of the different complexes (except for **5a**, which was formed *in situ* to avoid decomposition; the other oxidoiron(IV) complexes were isolated to separate from excess of sPhIO) ($5.12 \cdot 10^{-6}$ mol) was dissolved in acetonitrile (0.5 mL) and was reacted with 5 eq. of DHA or CHD ($2.56 \cdot 10^{-5}$ mol) in 0.5 mL solvent (1:1 mixture of MeCN and DCM). The yield was determined after 10 min of reaction time at −40 °C. Prior to injection, cyclododecane (CDD, 350 µL of a 51.2 mM solution in 1:1 MeCN/DCM) was added to the solution was filtered through silica and washed with acetonitrile (2 mL) and DCM (3.65 mL). Anthracene and benzene, respectively, were the only observed products and the yields were quantified by GC-FID using a calibration curve obtained with standard samples (linear fitting: DHA: y = 1.27207x, R^2 = 0.99929; anthracene: y = 1.36731x, R^2 = 0.99933; CHD: y = 0.45818x, R^2 = 0.9998; benzene: y = 0.52595x, R^2 = 0.99995).

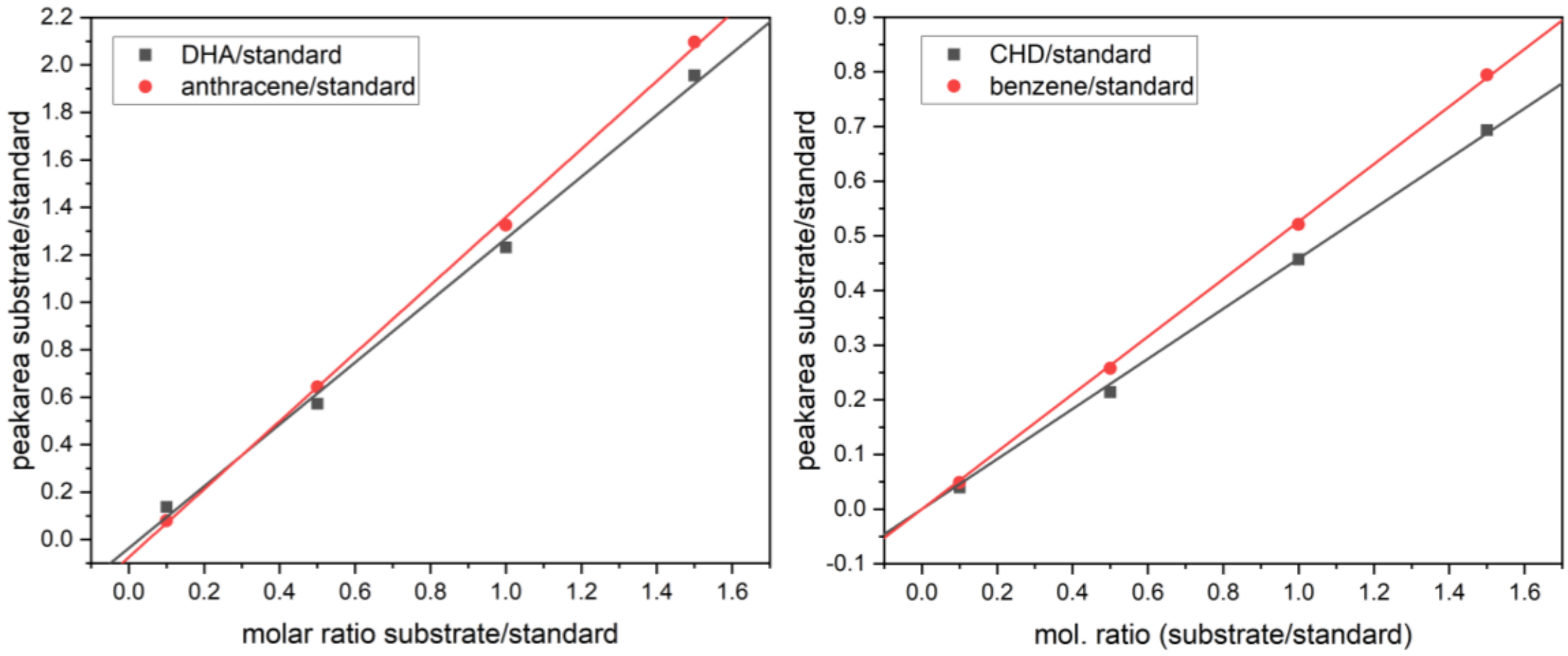

Figure S22. Calibration curves for GC-FID measurements with cyclododecane as standard.

Table S8. Determination of the yield of anthracene from the oxidation of DHA with different complexes.

	Peak area DHA/CDD	Peak area anthracene/CDD	*n*(DHA) [mol]	*n*(anthracene) [mol]	Yield [%]
**3**	1.41016427	0.14389117	$2.8379 \cdot 10^{-6}$	$2.6941 \cdot 10^{-7}$	86.7
	1.567618841	0.144127053	$3.15478 \cdot 10^{-6}$	$2.69848 \cdot 10^{-7}$	78.8
	1.406829023	0.141350741	$2.8312 \cdot 10^{-6}$	$2.64649 \cdot 10^{-7}$	85.5
**4**	1.53544914	0.1794276	$3.09 \cdot 10^{-6}$	$3.3594 \cdot 10^{-7}$	98.1
	1.656148341	0.120527001	$3.33295 \cdot 10^{-6}$	$2.25661 \cdot 10^{-7}$	63.4
	1.524041657	0.175880789	$3.06708 \cdot 10^{-6}$	$3.293 \cdot 10^{-7}$	97.0
**5a**	1.42675464	0.13882829	$2.8713 \cdot 10^{-6}$	$2.5993 \cdot 10^{-7}$	83.0
	1.396144578	0.129060241	$2.8097 \cdot 10^{-6}$	$2.41638 \cdot 10^{-7}$	79.1
	1.403404924	0.134625458	$2.82431 \cdot 10^{-6}$	$2.52058 \cdot 10^{-7}$	81.2

Table S9. Determination of the yield of benzene from the oxidation of CHD with different complexes.

	Peak area CHD/CDD	Peak area benzene/CDD	*n*(CHD) [mol]	*n*(benzene) [mol]	Yield [%]
**3**	0.50579879	0.064979746	$2.82606 \cdot 10^{-6}$	$3.16281 \cdot 10^{-7}$	100.7
	0.490250244	0.062452605	$2.73919 \cdot 10^{-6}$	$3.03981 \cdot 10^{-7}$	99.9
	0.492960809	0.062890689	$2.75433 \cdot 10^{-6}$	$3.06113 \cdot 10^{-7}$	100.0
**4**	0.574956217	0.065674256	$3.21247 \cdot 10^{-6}$	$3.19662 \cdot 10^{-7}$	90.4
	0.574271873	0.065429289	$3.20864 \cdot 10^{-6}$	$3.18469 \cdot 10^{-7}$	90.3
	0.560383715	0.063895392	$3.13105 \cdot 10^{-6}$	$3.11003 \cdot 10^{-7}$	90.4
**5a**	0.569976722	0.062851303	$3.18464 \cdot 10^{-6}$	$3.05921 \cdot 10^{-7}$	87.5
	0.557324132	0.061219947	$3.11395 \cdot 10^{-6}$	$2.97981 \cdot 10^{-7}$	87.3
	0.558487198	0.061694651	$3.12045 \cdot 10^{-6}$	$3.00291 \cdot 10^{-7}$	87.8

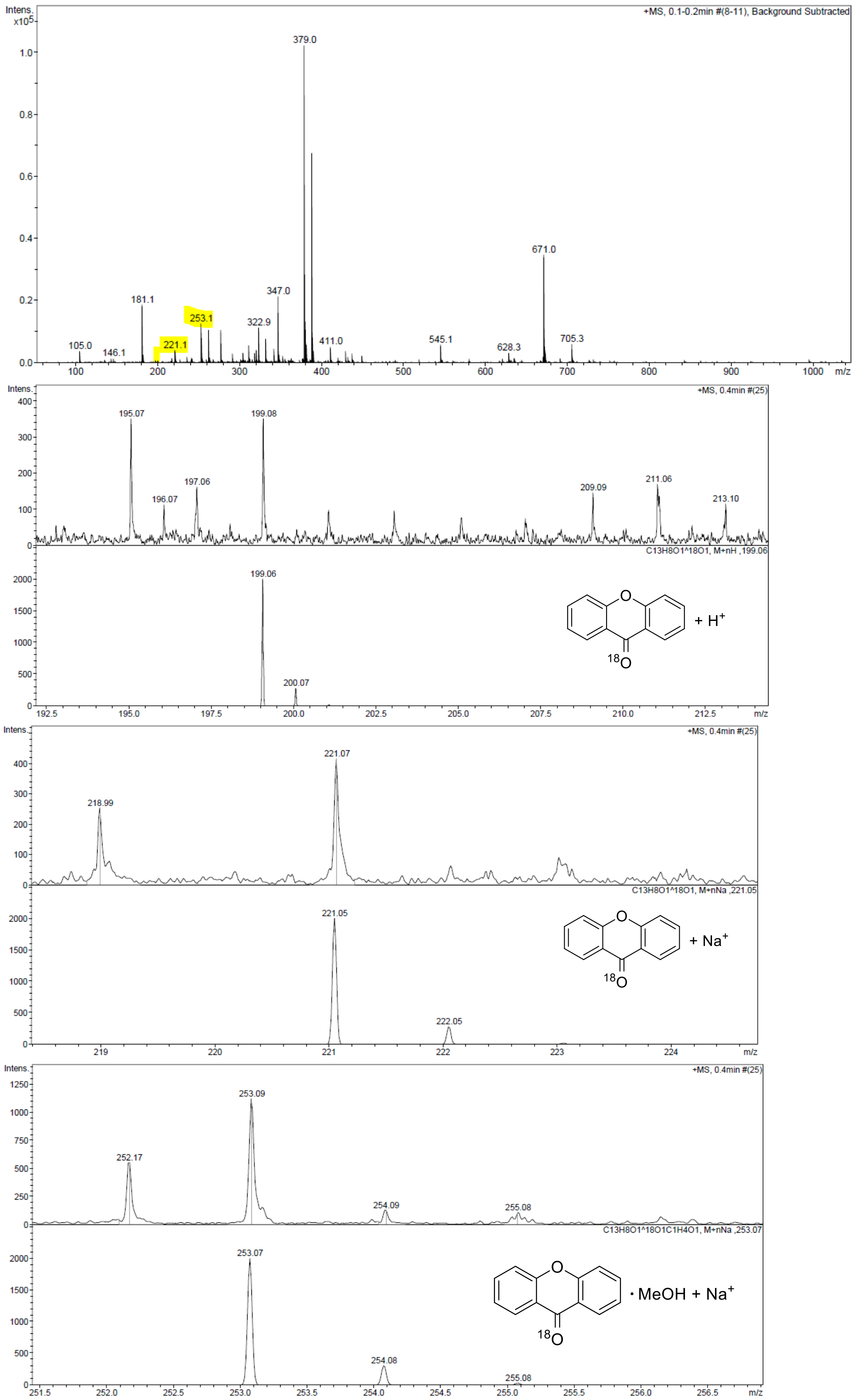

Figure S23. ESI-MS spectra of the reaction of ^{18}O-labelled complex **3** with XanH. The marked peaks are enlarged and simulated and confirm some substrate oxygenation. Complexes **4** and **5a** show similar behavior with XanH.

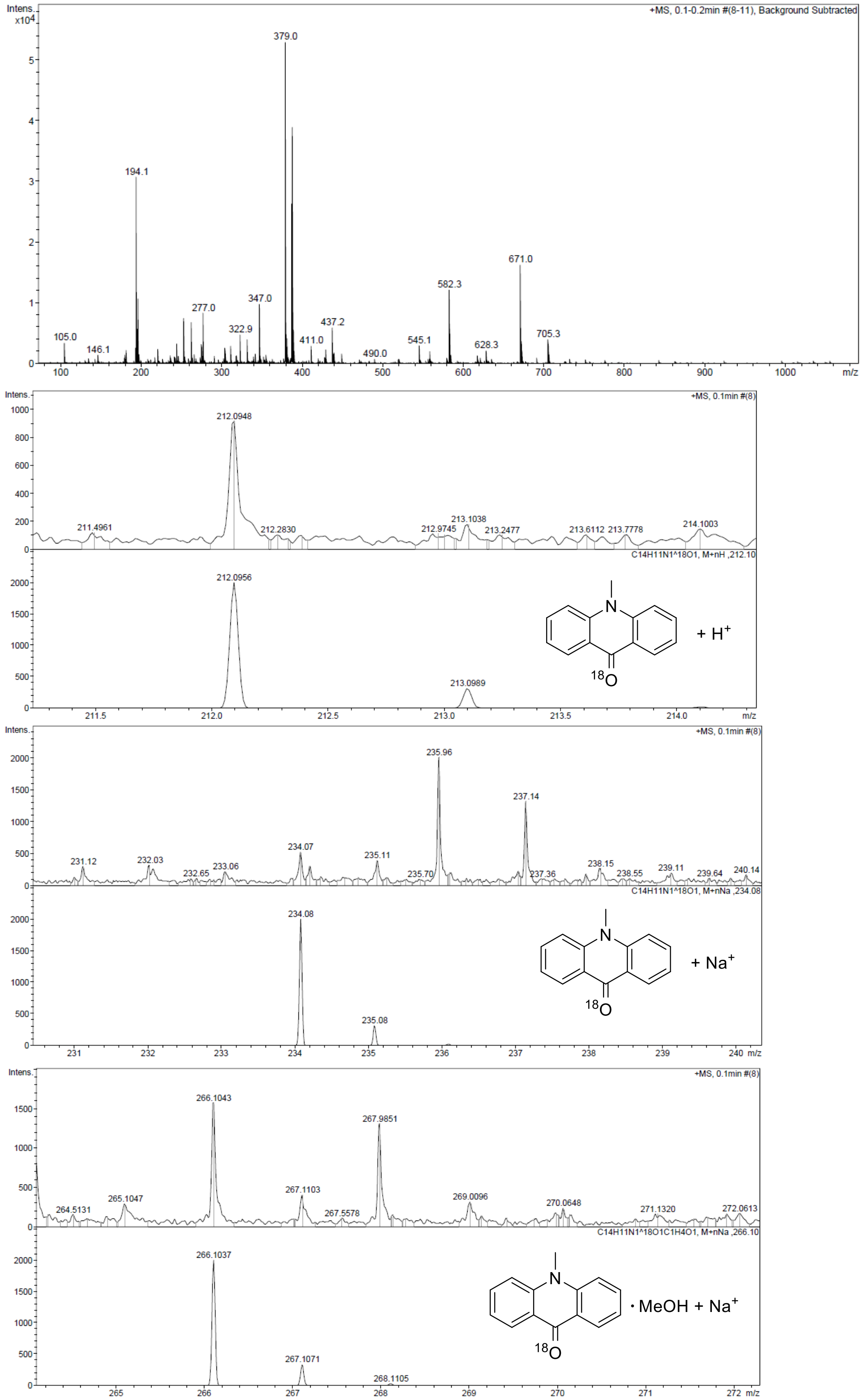

Figure S24. ESI-MS spectra of the reaction of ^{18}O-labelled complex **3** with AcrH. The peaks showing the oxygenated products are enlarged and simulated. Complexes **4** and 5**a** show similar behavior with AcrH.

7 DFT Calculations

All calculations were carried out using the ORCA 4.2.1 suite of quantum chemical programs.[12,13] Geometry optimizations were performed employing the hybrid-GGA B3LYP density[14,15] functional in the gas phase unless explicitly stated otherwise. Ahlrichs' triple-ζ quality basis set, def2-TZVP,[16] was applied to Fe and atoms of the first coordination sphere, while the double-ζ def2-SVP[17] basis set was used for the remaining atoms during the optimization. The scalar relativistic zeroth-order regular approximation (ZORA)[18,19] was applied in geometry optimizations as well as single-point energy calculations. The hybrid DFT calculations were accelerated using the resolution of the identity for the Coulomb part (J) and a chain-of-spheres algorithm for the Hartree−Fock exchange part (X) (RIJCOSX) approximation,[20] combined with a fine integration grid (GridX5) and the auxiliary Coulomb-fitting basis set def2/J. Grimme's atom-pairwise dispersion correction with Becke−Johnson (D3BJ) damping[21] was employed to account for non-covalent interactions (NCIs).

For solvent-phase geometry optimizations, implicit solvation effects were incorporated using a conductor-like polarizable continuum model with acetonitrile (ε = 36.6) as the solvent.[22] The nature of all optimized stationary points was confirmed via harmonic vibrational frequency calculations, which yielded either zero (for local minima) or one (for first-order saddle points) imaginary frequency. The zero-point vibrational energies, thermal corrections, and entropy terms were obtained from the frequency calculations. Gibbs free energies were calculated from the electronic energies of the stationary points, corrected for larger basis sets using the Ahlrichs' triple-ζ basis set with single polarization (def2-TZVP) for all atoms, along with free-energy corrections from the optimization level of theory.

The σ- and π-pathways are characterized by distinct electronic structures. In the σ-pathway (triplet surface), the Fe centre exhibits a local $S = 3/2$ spin configuration, while the substrate C centre carries a down-spin unpaired electron with local $S = 1/2$. This gives rise to an antiferromagnetically coupled state with $M_S = 1$, which can be described within the broken-symmetry DFT framework as a BS(3,1) solution. Accordingly, the transition state (TS_σ) and intermediate (Int_σ) for the σ-pathway were optimized using the BS-DFT formalism. In contrast, for the π-pathway, the Fe centre has a local $S = 1/2$ spin configuration, and the substrate C centre carries an up-spin unpaired electron with local $S = 1/2$. This leads to a ferromagnetically coupled pure triplet ($S = 1$) state. Therefore, the transition state (TS_π) and intermediate (Int_π) associated with the π-pathway were optimized on the pure triplet surface. To further validate the assignments, we analysed the relevant molecular orbitals and geometries of the transition states and intermediates.

To verify spin-state energetics and obtain reliable electronic structures and d−d transition energies, ab initio multiconfigurational CASSCF(12,12)[23,24] and CASSCF(12,9) calculations followed by NEVPT2[25] were employed, respectively. A balanced active space was constructed, including equatorial bonding (σ_{eq}), three Fe−O bonding orbitals (σ_z, π_x, π_y), the corresponding antibonding orbitals ($^*\sigma_z$, $^*\pi_x$, $^*\pi_y$), two nonbonding Fe-d_{x2-y2} and Fe-d_{xy} orbitals, and three 4d shell orbitals ($4d_{xy}$, $4d_{xz}$ and $4d_{yz}$) to account for the missing correlation. This resulted in an active space of 12 electrons in 12 orbitals, corresponding to CASSCF(12,12) calculations. For the d-d transition energies, a rather simpler version of the active space CASSCF(12,9) with 30 roots was utilized by removing equatorial bonding (σ_{eq}) and three 4d shell orbitals ($4d_{xy}$, $4d_{xz,}$ and $4d_{yz}$). This active space construction approach has been widely applied in literature for

analyzing the electronic structures and spectroscopic properties of oxidoiron(IV) complexes.[26] The def2-TZVPP basis set, def2-TZVPP/C correlation-fitting basis sets, and B3LYP-optimized geometries were used for the CASSCF/NEVPT2 calculations.

Intrinsic reaction coordinate calculations were performed with Gaussian 16 programs package[27] for the lowest energy transition states for complexes **3**, **4**, and **5a**. Geometry optimization for the transition states was calculated by employing LANL2DZ[28-30] basis set and effective core potential (ECP) on Fe, and the rest of the atoms were treated with 6-31+G(d)[31] basis set in conjunction with B3LYP functional. The estimated widths (in bohr·amu$^{1/2}$), and full width at half maxima (FWHM), were obtained from the resulting IRC plots.

Table S10. Comparison of the key *d*-*d* transition energies (cm^{-1}) obtained at the CASSCF(12,9)/NEVPT2 level of theory.

**Transitions**	Complex **3**	Complex **4**	Complex **5a**
$d_{xz,yz} \rightarrow d_{z2}$	18312, 18273	17192, 16853	19370, 19153
$d_{xz,yz} \rightarrow d_{x2-y2}$	24217, 24655	24051, 24599	24185, 23711

Table S11. Spin-state energetics between triplet and quintet state at the DFT and CASSCF/NEVPT2 levels.

**Complex**	**Spin State**	**Δ*E*, kcal/mol**	
		DFT-B3LYP	CASSCF(12,12)/NEVPT2
**3′**	*S = 1*	0.0	0.0
	S = 2	10.8	16.8
**3**	*S = 1*	0.0	0.0
	S = 2	28.4	26.4
**4**	*S = 1*	0.0	0.0
	S = 2	17.4	33.4
**5a**	*S = 1*	0.0	0.0
	S = 2	24.3	33.5

Table S12. Key geometric parameters and calculated stretching frequencies ν(Fe–O) obtained from DFT-B3LYP/zora-Def2-TZVP(CPCM, acetonitrile) optimized structures in (*S* = 1) ground-state.

Complex	*d*(Fe–O) [Å] (DFT)	*d*(Fe–O) [Å] (exp.)	*d*(Fe–ax) [Å] (DFT)	ν_{Fe-O} [cm^{-1}] (DFT)
**3′**	1.618		--	945
**3**	1.645	1.661(3)	2.074	881
**4**	1.644	1.671(1)	2.106	885
**5a**	1.672		2.486	820

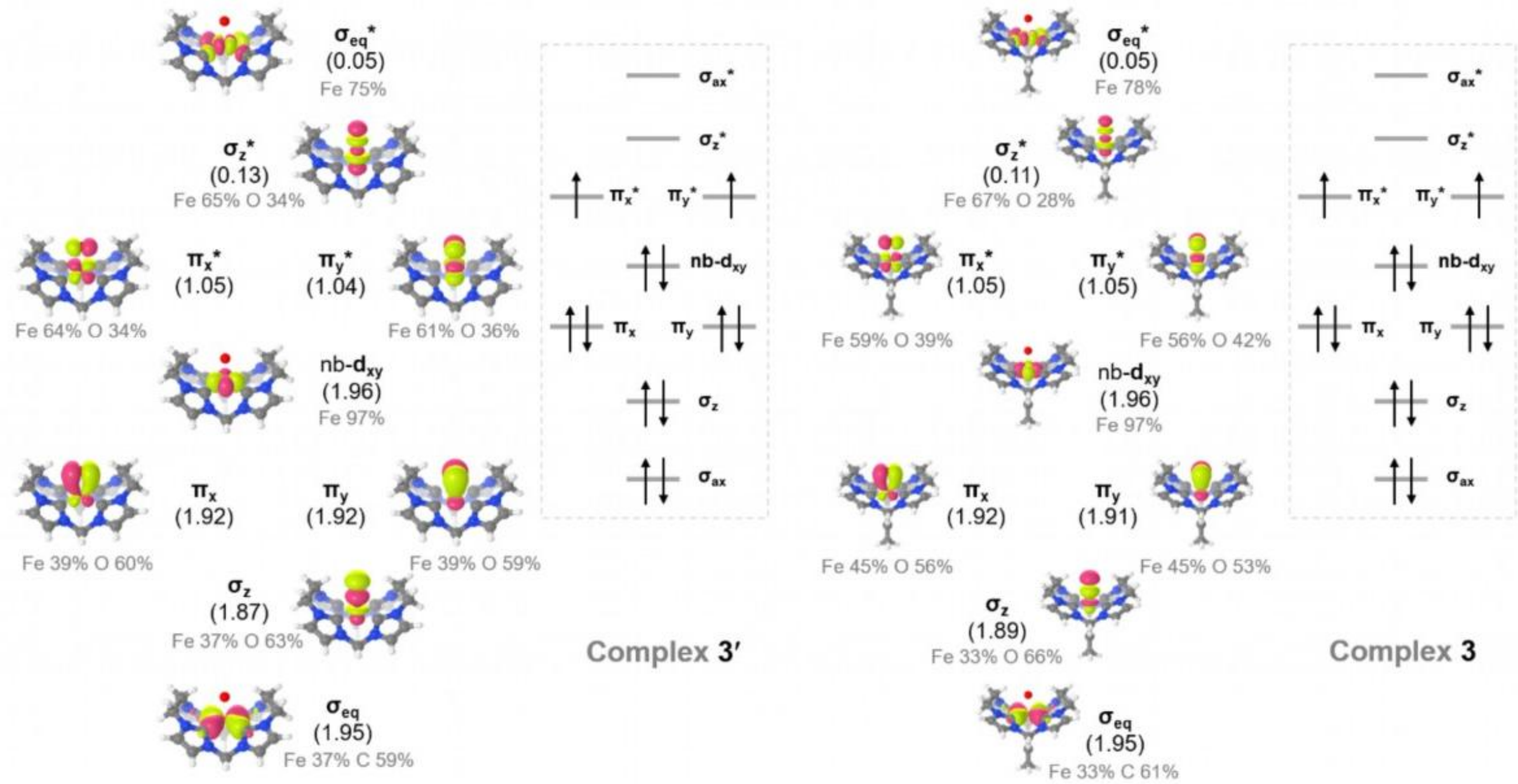

Figure S25. Natural orbitals along with their atomic orbital contributions and major ground state electronic configuration for complexes **3'** and **3** obtained at the CASSCF(12,12) level of theory.

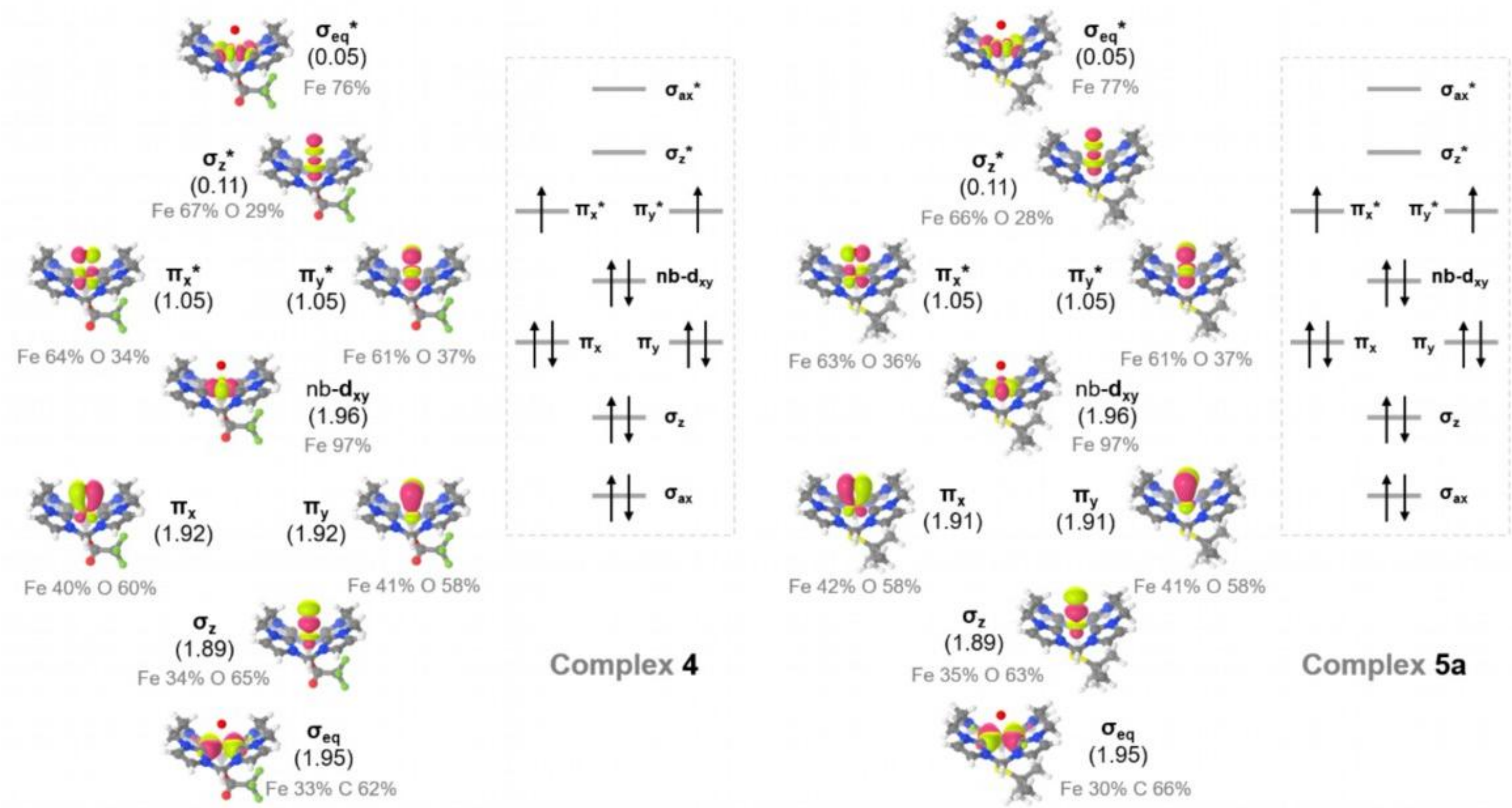

Figure S26. Natural orbitals along with their atomic orbital contributions and major ground state electronic configuration for complexes **4** and **5a** obtained at the CASSCF(12,12) level of theory.

Table S13. Calculated enthalpy (Δ$H^{‡}$, kcal/mol) and Gibbs free energy (Δ$G^{‡}$, kcal/mol) barrier for the HAT reactions exhibited by complexes **3′** and **3-5**.

	**3′**		**3**		**4**		**5a**
TS	$^{3}TS_{\sigma}$	$^{3}TS_{\pi}$	$^{3}TS_{\sigma}$	$^{3}TS_{\pi}$	$^{3}TS_{\sigma}$	$^{3}TS_{\pi}$	$^{3}TS_{\pi}$
Δ$H^{‡}$ [kcal/mol]	5.7	10.1	12.2	10.4	14.9	11.7	12.1
Δ$G^{‡}_{25°C}$ [kcal/mol]	11.0	15.7	15.3	15.1	19.3	17.3	18.4

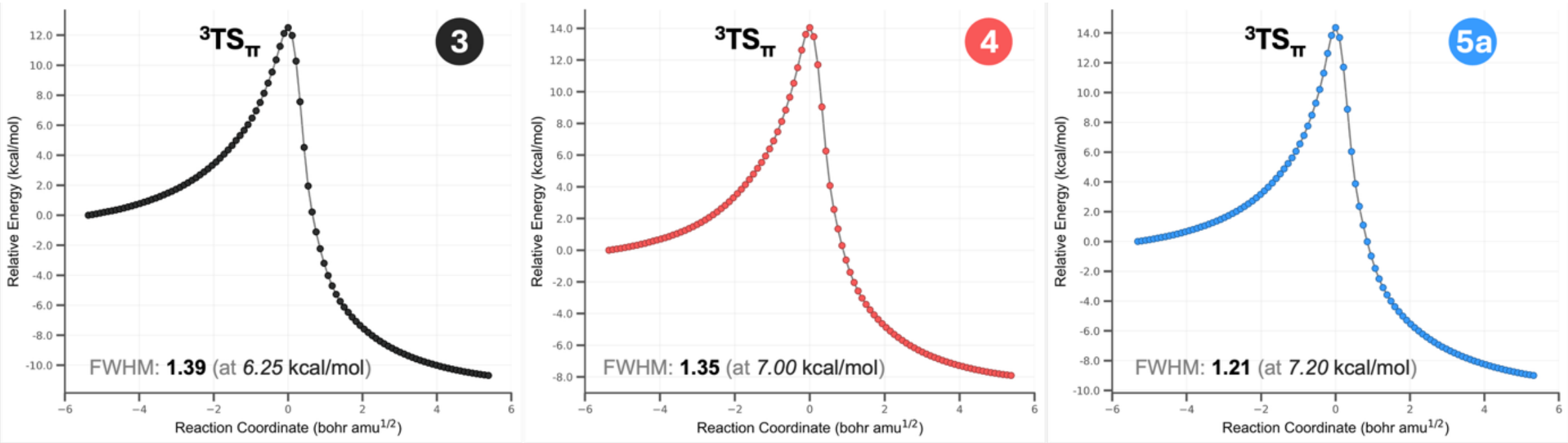

Figure S27. Intrinsic reaction coordinates (IRCs) calculated for the lowest energy transition states for complexes **3**, **4**, and **5a**. The estimated widths (in bohr·amu$^{1/2}$), full width at half maxima (FWHM), are shown along with the IRCs.

Table S14. Bond dissociation free energy (BDFE) of the Fe(III)-OH intermediate involved in the lowest energy HAA pathway calculated at the DFT-B3LYP/zora-Def2-TZVP(CPCM, Acetonitrile) level of theory.

$BDFE_{O–H}$	**3′**	**3**	**4**	**5**
Int	$^{3}Int_{\sigma}$	$^{3}Int_{\pi}$	$^{3}Int_{\pi}$	$^{3}Int_{\pi}$
Δ$G_{25°C}$ [kcal/mol]	88.2	80.0	75.6	77.8

8 References

(1) Meyer, S.; Klawitter, I.; Demeshko, S.; Bill, E.; Meyer, F. A Tetracarbene–Oxoiron(IV) Complex. *Angew. Chem. Int. Ed.* **2013**, *52* (3), 901–905. DOI: 10.1002/anie.201208044.

(2) Becker, I.; Morganti, M.; Gross, S. J.; Demeshko, S.; Dechert, S.; John, M.; Meyer, F. Axial Ligand Lability and Coordination Induced Spin State Variations of Tetracarbene Iron(II) Thiolato Complexes. *Eur. J. Inorg. Chem.* **2025**, *28*, e202400823. DOI: 10.1002/ejic.202400823.

(3) Goldsmith, C. R.; Jonas, R. T.; Stack, T. D. P. C−H Bond Activation by a Ferric Methoxide Complex: Modeling the Rate-Determining Step in the Mechanism of Lipoxygenase. *J. Am. Chem. Soc.* **2002**, *124* (1), 83–96. DOI: 10.1021/ja016451g.

(4) Mondal, P. K.; Tiwari, S. K.; Singh, P.; Pandey, G. Direct Arylation of Distal and Proximal C(sp3)–H Bonds of t-Amines with Aryl Diazonium Tetrafluoroborates via Photoredox Catalysis. *J. Org. Chem.* **2021**, *86* (23), 17184–17196. DOI: 10.1021/acs.joc.1c02286.

(5) Stoll, S.; Schweiger, A. *J. Magn. Res.* **2006**, *178* (1), 42–55. DOI: 10.1016/j.jmr.2005.08.013.

(6) Jašík, J.; Žabka, J.; Roithová, J.; Gerlich, D. Infrared spectroscopy of trapped molecular dications below 4 K. *Int. J. Mass Spectrom.* **2013**, *354-355*, 204-210. DOI: 10.1016/j.ijms.2013.06.007.

(7) Roithová, J.; Gray, A.; Andris. E.; Jašík, J.; Gerlich, D. Helium Tagging Infrared Photodissociation Spectroscopy of Reactive Ions. *Acc. Chem. Res.* **2016**, *49* (2), 223–230. DOI: 10.1021/acs.accounts.5b00489.

(8) Sheldrick, G. M. SHELXT - integrated space-group and crystal-structure determination. *Acta Cryst. A* **2015**, *71* (Pt 1), 3–8. DOI: 10.1107/S2053273314026370. Published Online: Jan. 1, 2015.

(9) Sheldrick, G. M. Crystal structure refinement with SHELXL. *Acta Cryst C* **2015**, *71* (Pt 1), 3–8. DOI: 10.1107/S2053229614024218. Published Online: Jan. 1, 2015.

(10) X-RED; STOE & CIE GmbH, Darmstadt, Germany **2002**.

(11) Seabold, S.; Perktold, J. Statsmodels: Econometric and Statistical Modeling with Python. *Proc. of the 9th Python in Science Conf.* **2010**.

(12) Neese, F. The ORCA Program System. *WIREs Comput. Mol. Sci.* **2012**, *2*, 73–78. https://doi.org/10.1002/wcms.81.

(13) Neese, F. Software Update: The ORCA Program System, Version 4.0. *WIREs Comput. Mol. Sci.* **2018**, *8*, 1–6. https://doi.org/10.1002/wcms.1327.

(14) Becke, A. D. Density-Functional Exchange-Energy Approximation with Correct Asymptotic Behavior. *Phys. Rev. A* **1988**, *38*, 3098–3100. https://doi.org/10.1103/PhysRevA.38.3098.

(15) Lee, C.; Yang, W.; Parr, R. G. Development of the Colle-Salvetti Correlation-Energy Formula into a Functional of the Electron Density. *Phys. Rev. B* **1988**, *37*, 785–789. https://doi.org/10.1103/PhysRevB.37.785.

(16) Schäfer, A.; Huber, C.; Ahlrichs, R. Fully Optimized Contracted Gaussian Basis Sets of Triple Zeta Valence Quality for Atoms Li to Kr. *J. Chem. Phys.* **1994**, *100*, 5829–5835. https://doi.org/10.1063/1.467146.

(17) Schäfer, A.; Horn, H.; Ahlrichs, R. Fully Optimized Contracted Gaussian Basis Sets for Atoms Li to Kr. *J. Chem. Phys.* **1992**, *97*, 2571–2577. https://doi.org/10.1063/1.463096.

(18) Van Lenthe, E.; Baerends, E. J.; Snijders, J. G. Relativistic Total Energy Using Regular Approximations. *J. Chem. Phys.* **1994**, *101*, 9783–9792. https://doi.org/10.1063/1.467943.

(19) Van Lenthe, E.; Baerends, E. J.; Snijders, J. G. Relativistic Regular Two-component Hamiltonians. *J. Chem. Phys.* **1993**, *99*, 4597–4610. https://doi.org/10.1063/1.466059.

(20) Neese, F.; Wennmohs, F.; Hansen, A.; Becker, U. Efficient, Approximate and Parallel Hartree–Fock and Hybrid DFT Calculations. A 'Chain-of-Spheres' Algorithm for the Hartree–Fock Exchange. *Chem. Phys.* **2009**, *356*, 98–109. https://doi.org/10.1016/J.CHEMPHYS.2008.10.036.

(21) Grimme, S.; Ehrlich, S.; Goerigk, L. Effect of the Damping Function in Dispersion Corrected Density Functional Theory. *J. Comput. Chem.* **2011**, *32*, 1456–1465. https://doi.org/10.1002/JCC.21759.

(22) Takano, Y.; Houk, K. N. Benchmarking the Conductor-like Polarizable Continuum Model (CPCM) for Aqueous Solvation Free Energies of Neutral and Ionic Organic Molecules. *J. Chem. Theory Comput.* **2005**, *1*, 70–77.

(23) Roos, B. O. The Complete Active Space Self-Consistent Field Method and Its Applications in Electronic Structure Calculations. *Ab Initio Methods in Quantum Chemistry - II* **2007**, *69*, 399–445. https://doi.org/10.1002/9780470142943.CH7.

(24) Ruedenberg, K.; Cheung, L. M.; Elbert, S. T. MCSCF Optimization through Combined Use of Natural Orbitals and the Brillouin–Levy–Berthier Theorem. *Int. J. Quantum Chem.* **1979**, *16* (5), 1069–1101. https://doi.org/10.1002/QUA.560160511.

(25) Angeli, C.; Cimiraglia, R.; Evangelisti, S.; Leininger, T.; Malrieu, J. P. Introduction of N-Electron Valence States for Multireference Perturbation Theory. *J. Chem. Phys.* **2001**, *114*, 10252–10264. https://doi.org/10.1063/1.1361246.

(26) Kupper, C.; Mondal, B.; Serrano-Plana, J.; Klawitter, I.; Neese, F.; Costas, M.; Ye, S.; Meyer, F. Nonclassical Single-State Reactivity of an Oxo-Iron(IV) Complex Confined to Triplet Pathways. *J. Am. Chem. Soc.* **2017**, *139*, 8939–8949. https://doi.org/10.1021/jacs.7b03255.

(27) Frisch, M. J.; Trucks, G. W.; Schlegel, H. B.; Scuseria, G. E.; Robb, M. A.; Cheeseman, J. R.; Scalmani, G.; Barone, V.; Petersson, G. A.; Nakatsuji, H.; Li, X.; Caricato, M.; Marenich, A. V; Bloino, J.; Janesko, B. G.; Gomperts, R.; Mennucci, B.; Hratchian, H. P.; Ortiz, J. V; Izmaylov, A. F.; Sonnenberg, J. L.; Williams-Young, D.; Ding, F.; Lipparini, F.; Egidi, F.; Goings, J.; Peng, B.; Petrone, A.; Henderson, T.; Ranasinghe, D.; Zakrzewski, V. G.; Gao, J.; Rega, N.; Zheng, G.; Liang, W.; Hada, M.; Ehara, M.; Toyota, K.; Fukuda, R.; Hasegawa, J.; Ishida, M.; Nakajima, T.; Honda, Y.; Kitao, O.; Nakai, H.; Vreven, T.; Throssell, K.; Montgomery Jr., J. A.; Peralta, J. E.; Ogliaro, F.; Bearpark, M. J.; Heyd, J. J.; Brothers, E. N.; Kudin, K. N.; Staroverov, V. N.; Keith, T. A.; Kobayashi, R.; Normand, J.; Raghavachari, K.; Rendell, A. P.; Burant, J. C.; Iyengar, S. S.; Tomasi, J.; Cossi, M.; Millam, J. M.; Klene, M.; Adamo, C.; Cammi, R.; Ochterski, J. W.; Martin, R. L.; Morokuma, K.; Farkas, O.; Foresman, J. B.; Fox, D. J. Gaussian˜16 {R}evision {B}.01. 2016.

(28) Hay, P. J.; Wadt, W. R. Ab Initio Effective Core Potentials for Molecular Calculations. Potentials for K to Au Including the Outermost Core Orbitals. *J. Chem. Phys.* **1985**, *82*, 299–310. https://doi.org/10.1063/1.448975.

(29) Wadt, W. R.; Hay, ; P Jeffrey; Wadt, W. A.; Hay, P. J. Ab Initio Effective Core Potentials for Molecular Calculations. Potentials for Main Group Elements Na to Bi. *J. Chem. Phys.* **1985**, *82*, 284–298. https://doi.org/10.1063/1.448800.

(30) Hay, P. J.; Wadt, W. R. Ab Initio Effective Core Potentials for Molecular Calculations. Potentials for the Transition Metal Atoms Sc to Hg. *J. Chem. Phys.* **1985**, *82*, 270–283. https://doi.org/10.1063/1.448799.

(31) Francl, M. M.; Pietro, W. J.; Hehre, W. J.; Binkley, J. S.; Gordon, M. S.; DeFrees, D. J.; Pople, J. A. Self-Consistent Molecular Orbital Methods. XXIII. A Polarization-Type Basis Set for Second-Row Elements. *J. Chem. Phys.* **1982**, *77*, 3654–3665. https://doi.org/10.1063/1.444267.

9 Exemplary Kinetic Traces and Fits

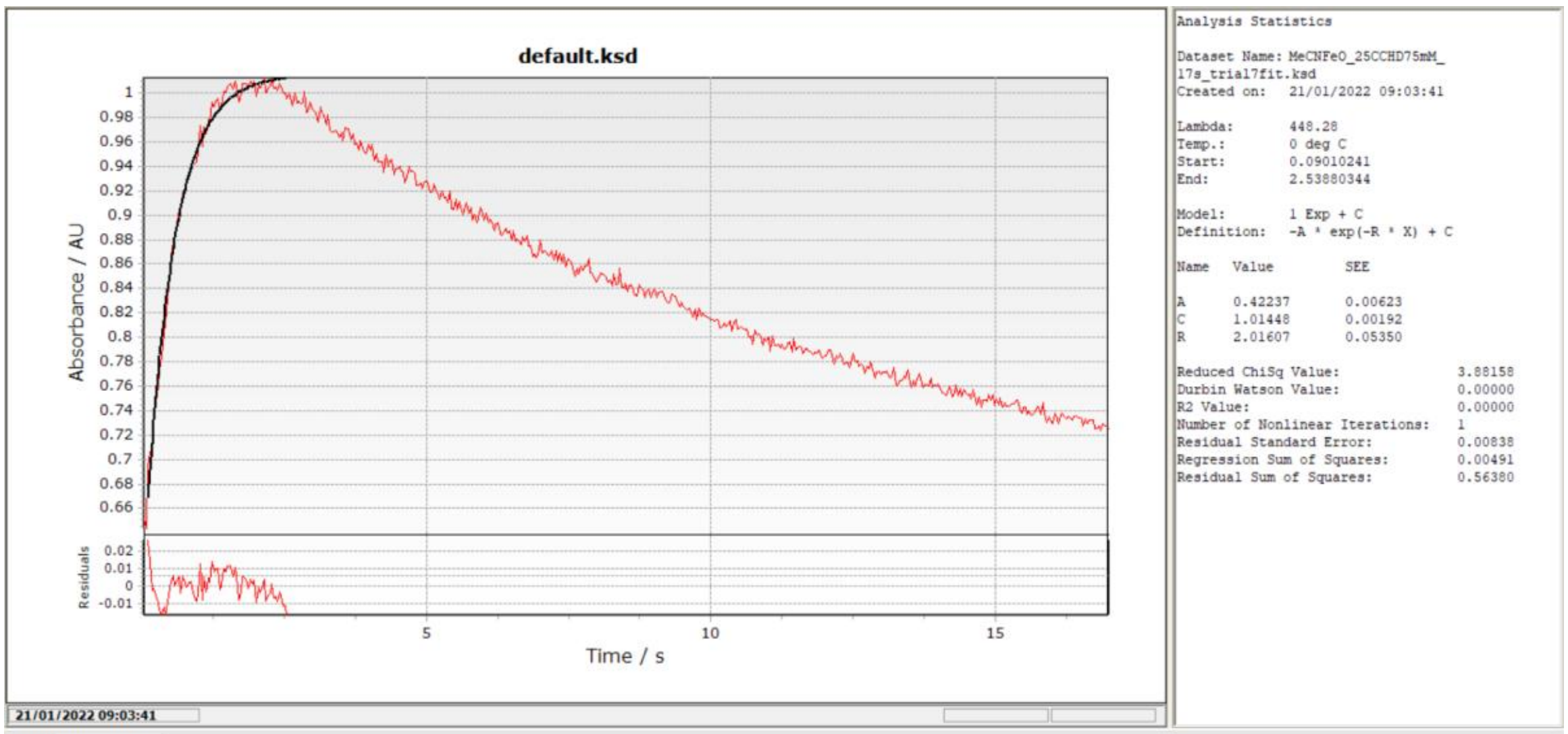

Figure S28. Exemplary trace (448 nm) and fit for the UV-Vis stopped-flow measurement of the reaction of **3** (1 mM) with 75 mM CHD at 25 °C in MeCN.

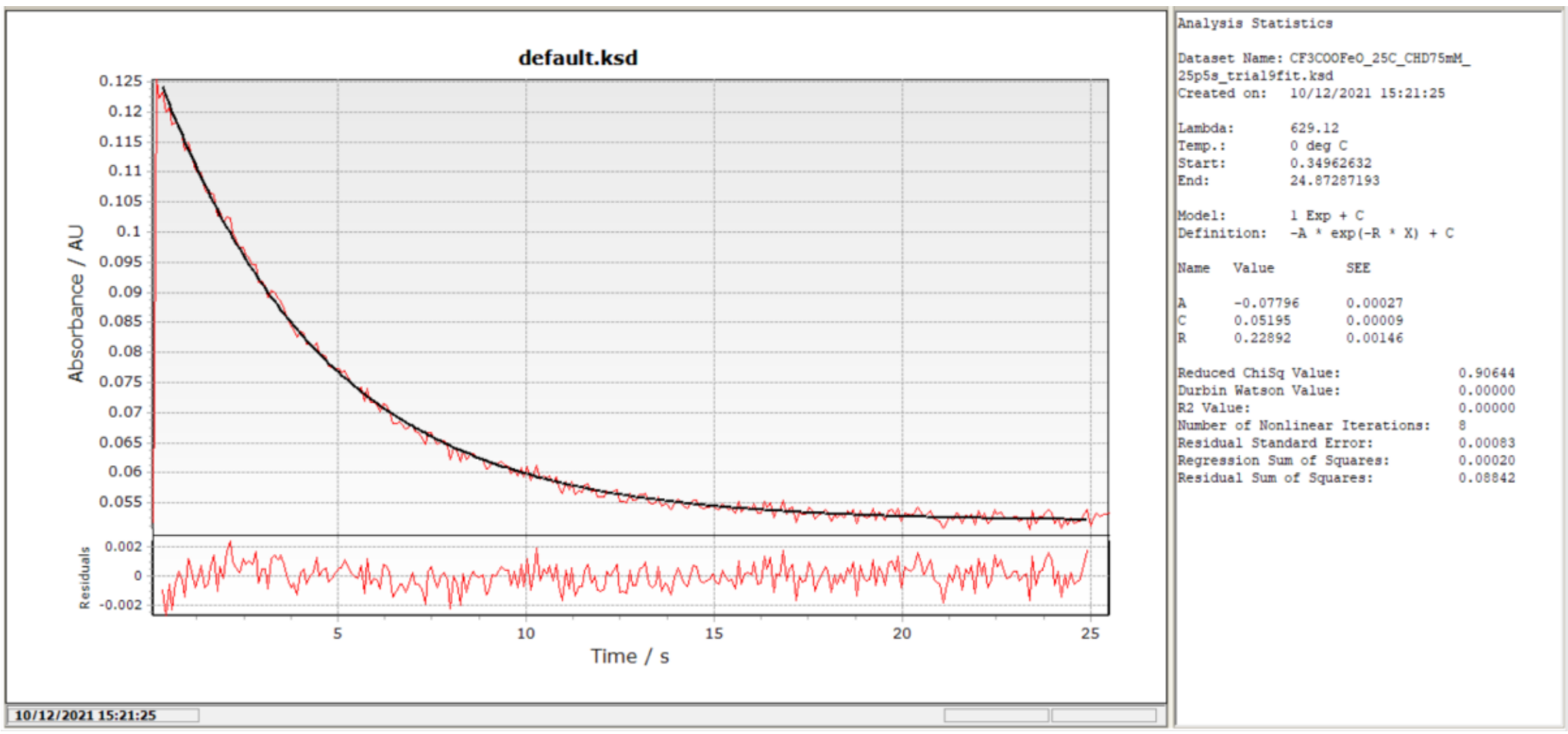

Figure S29. Exemplary trace (629 nm) and fit for the UV-Vis stopped-flow measurement of the reaction of **4** (1 mM) with 75 mM CHD at 25 °C in MeCN.

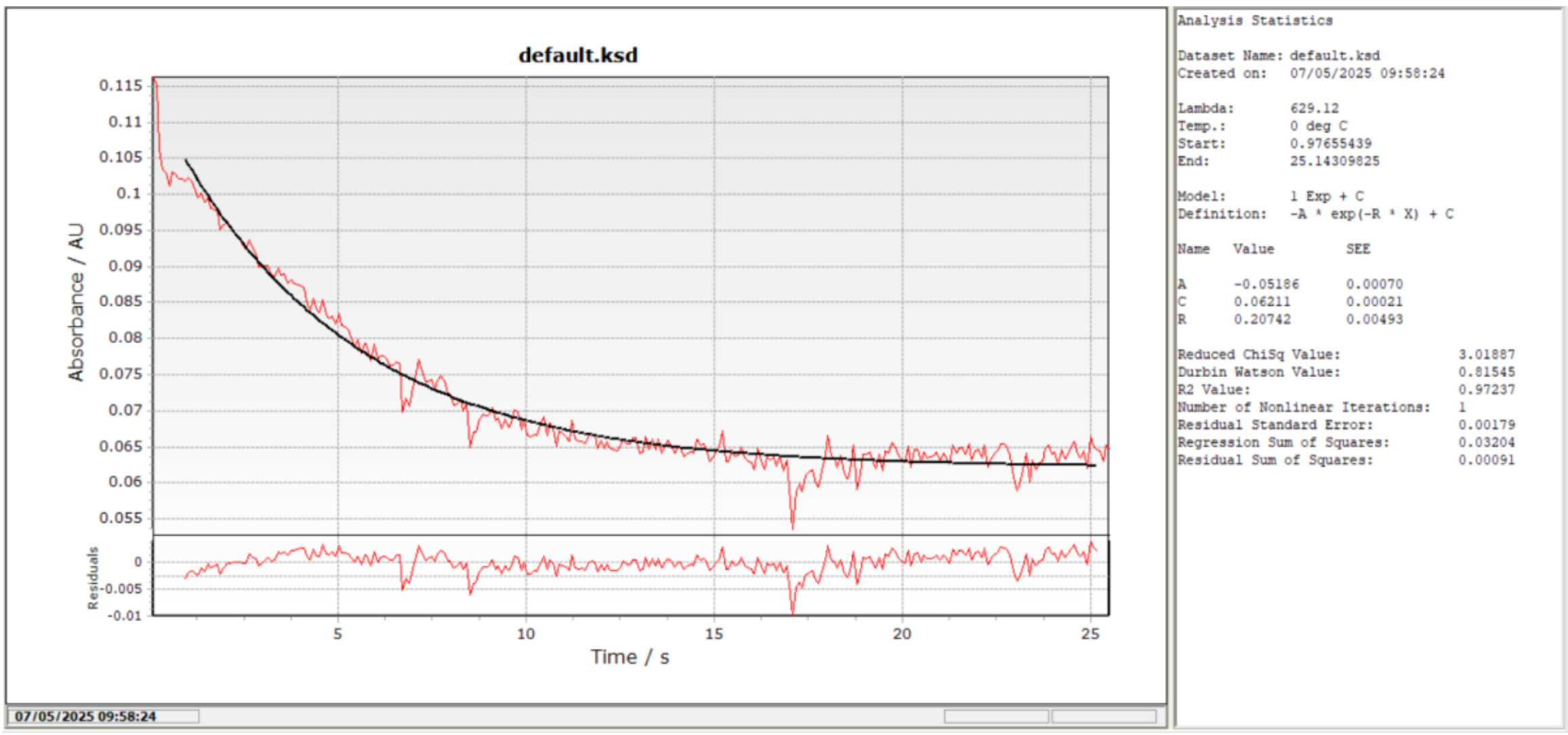

Figure S30. Exemplary trace (629 nm) and fit for the UV-Vis stopped-flow measurement of the reaction of **4** (1 mM) with additional 5 equivalents of NaTFA and 75 mM CHD at 25 °C in MeCN.

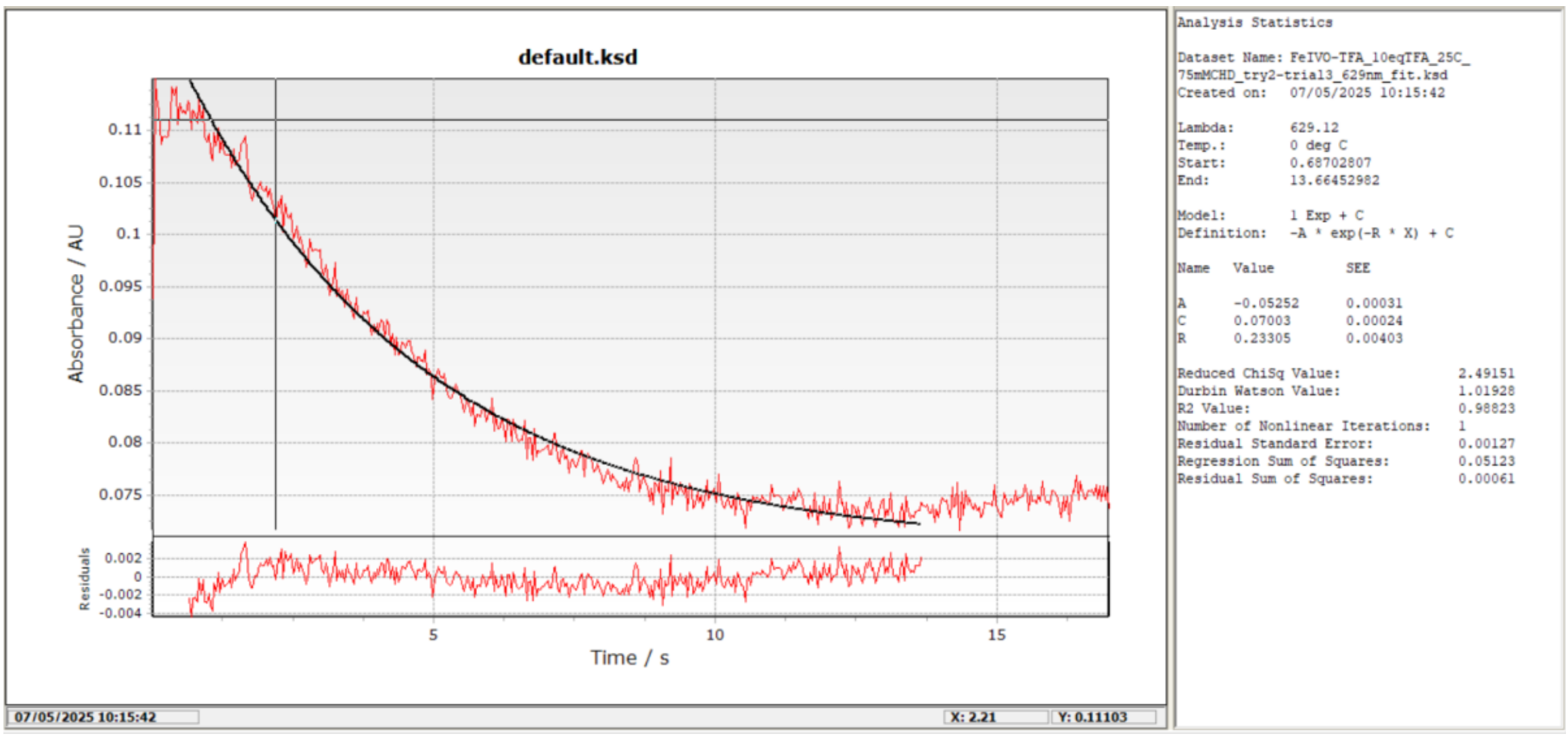

Figure S31. Exemplary trace (629 nm) and fit for the UV-Vis stopped-flow measurement of the reaction of **4** (1 mM) with additional 10 equivalents of NaTFA and 75 mM CHD at 25 °C in MeCN.

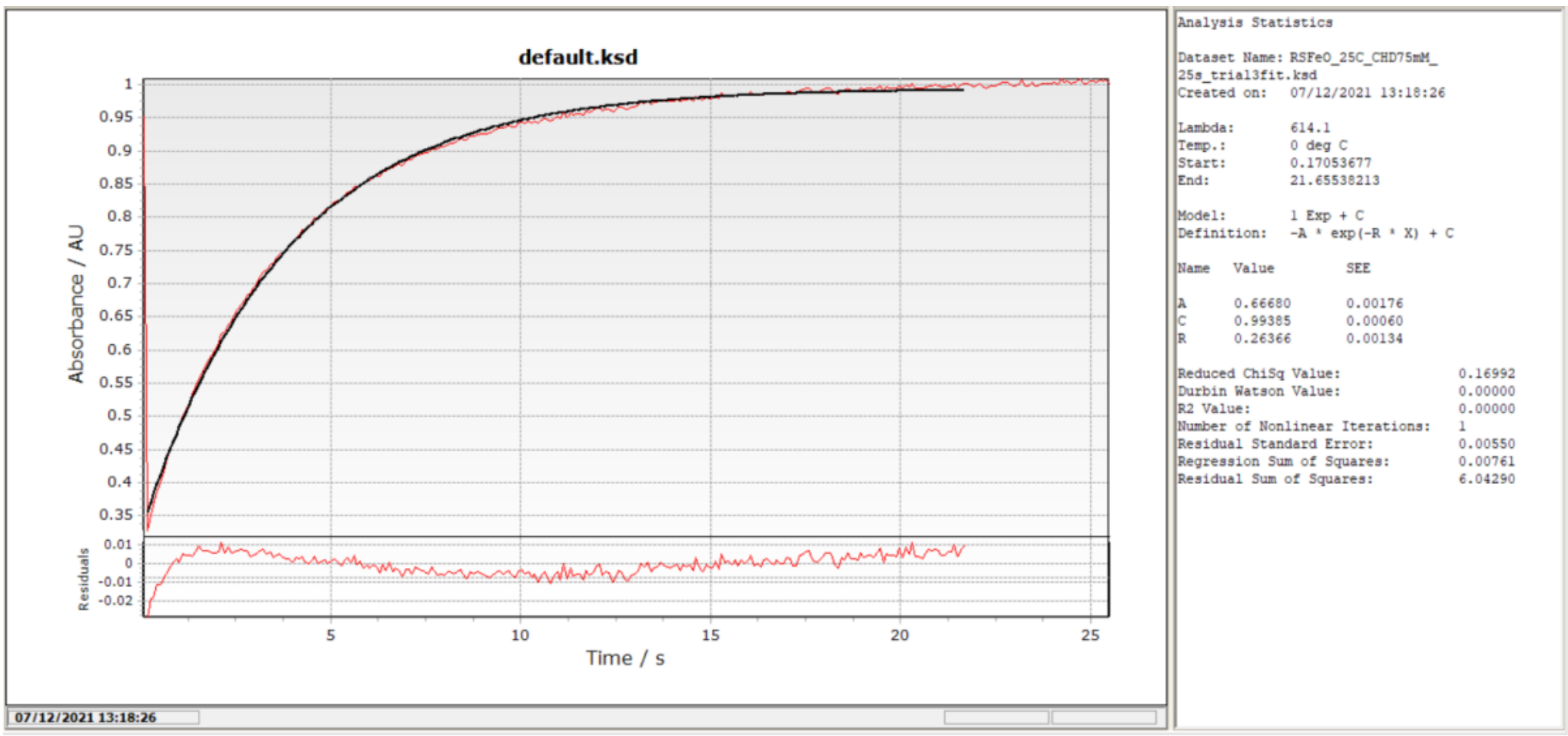

Figure 32. Exemplary trace (614 nm) and fit for the UV-Vis stopped-flow measurement of the reaction of **5a** (1 mM) with 75 mM CHD at 25 °C in MeCN.

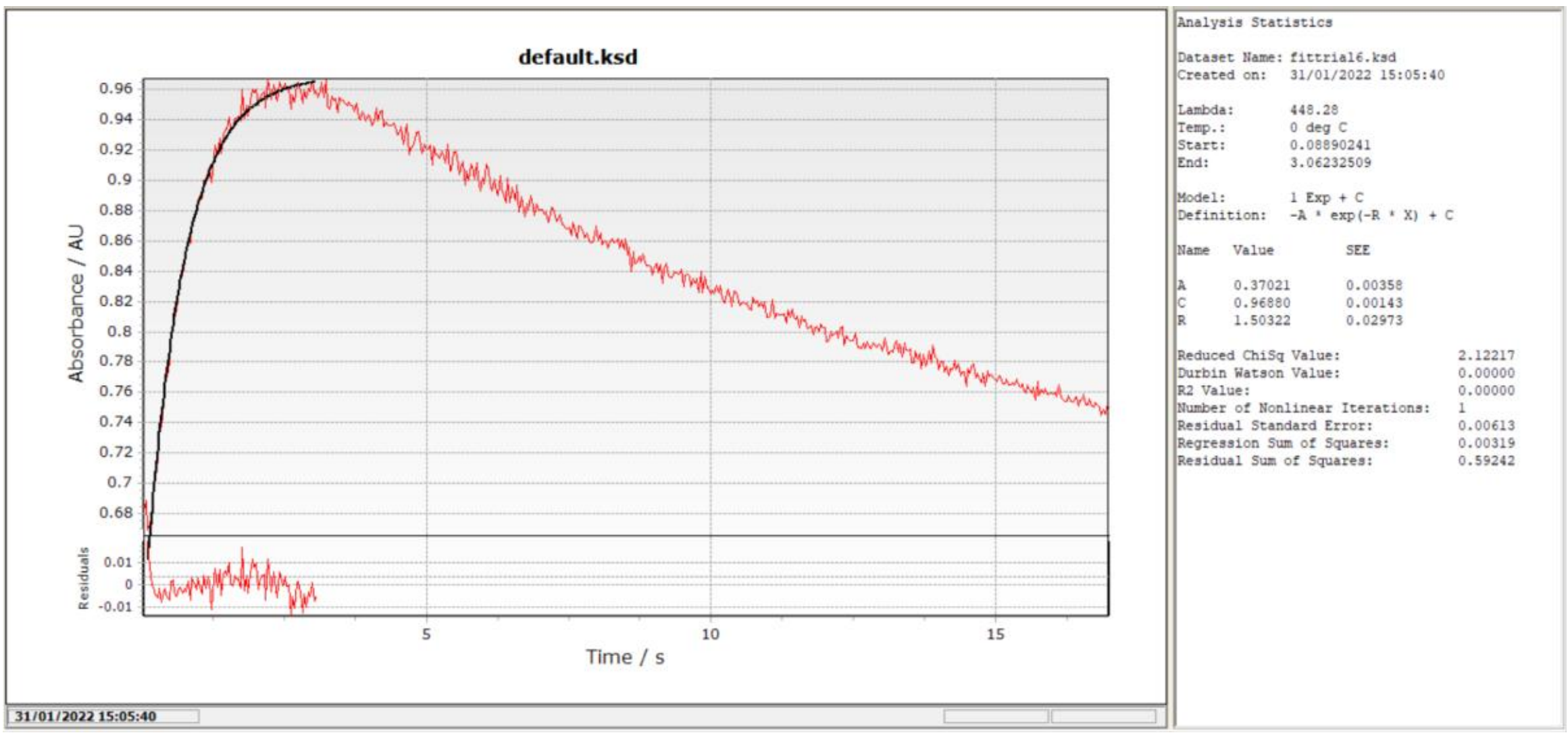

Figure S33. Exemplary trace (448 nm) and fit for the UV-Vis stopped-flow measurement of the reaction of **3** (1 mM) with 56.25 mM CHD at 25 °C in MeCN.

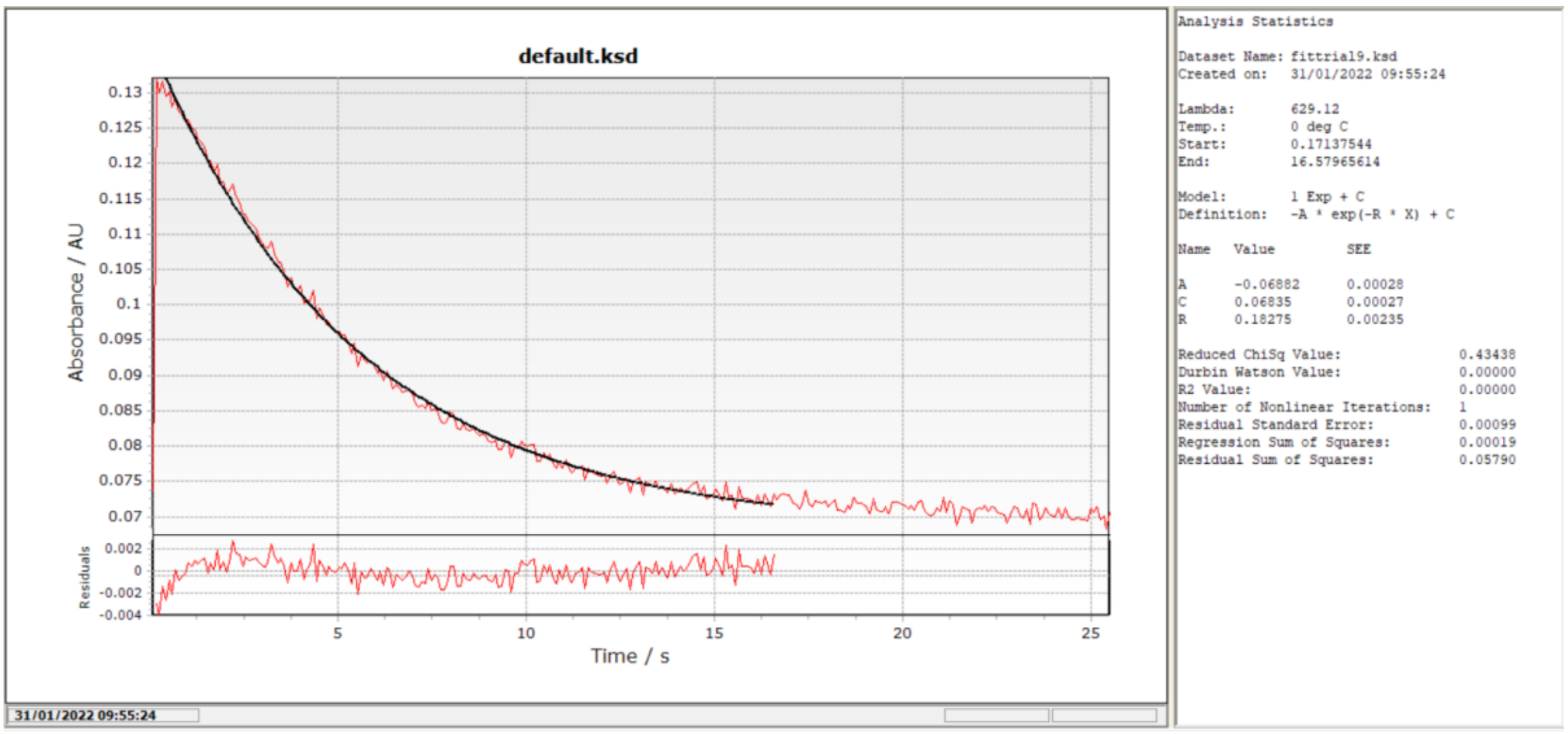

Figure S34. Exemplary trace (629 nm) and fit for the UV-Vis stopped-flow measurement of the reaction of **4** (1 mM) with 56.25 mM CHD at 25 °C in MeCN.

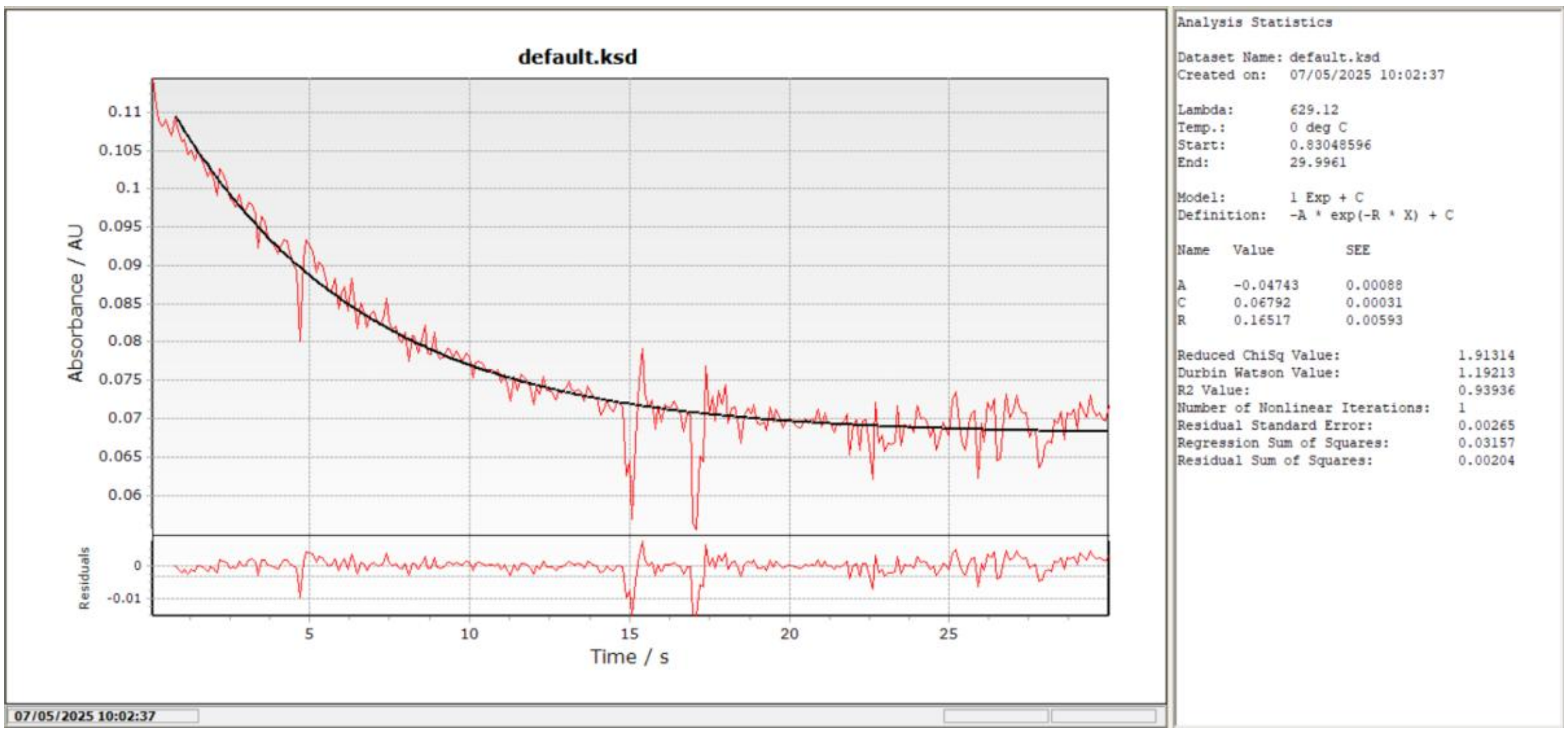

Figure S35. Exemplary trace (629 nm) and fit for the UV-Vis stopped-flow measurement of the reaction of **4** (1 mM) with additional 5 equivalents of NaTFA and 56.25 mM CHD at 25 °C in MeCN.

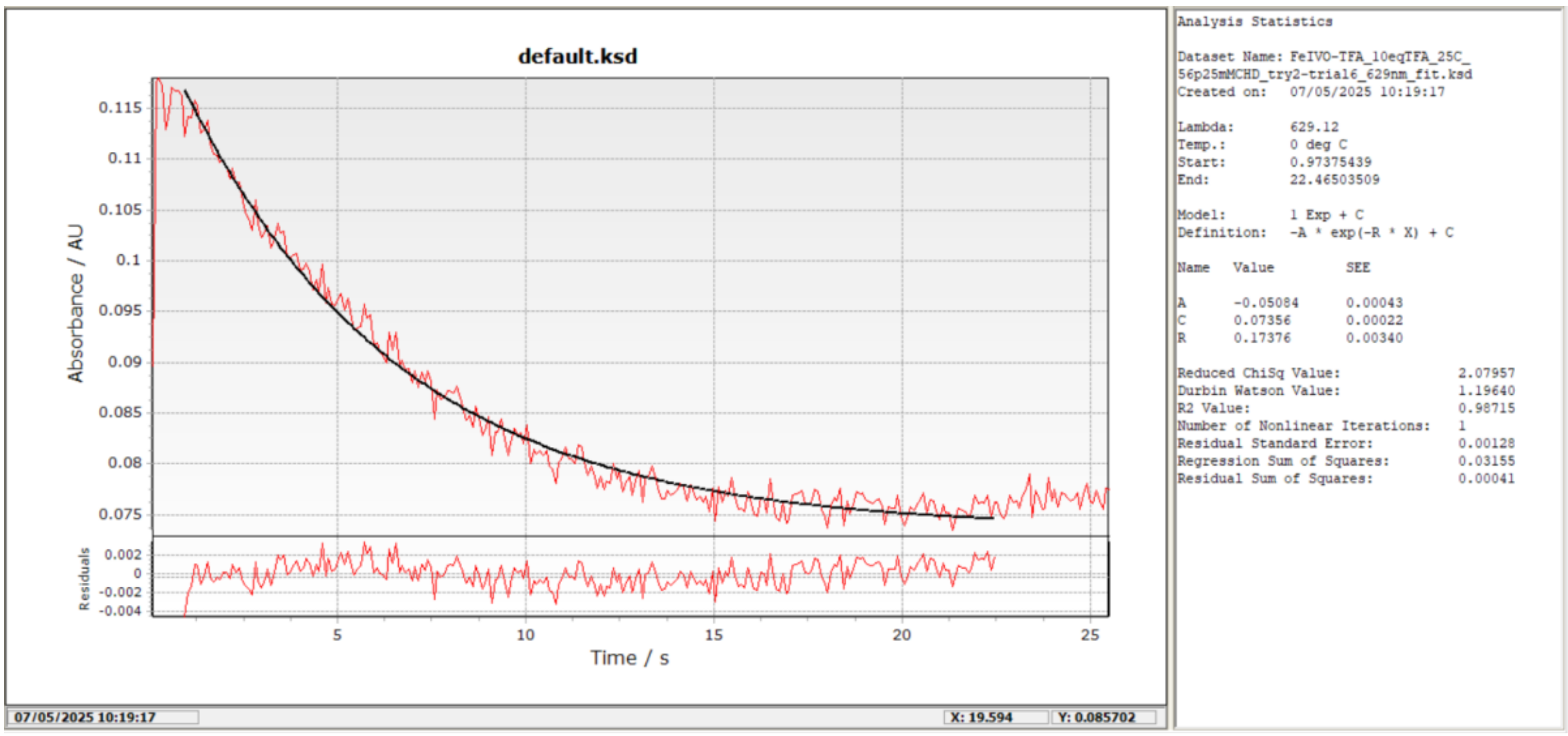

Figure S36. Exemplary trace (629 nm) and fit for the UV-Vis stopped-flow measurement of the reaction of **4** (1 mM) with additional 10 equivalents of NaTFA and 56.25 mM CHD at 25 °C in MeCN.

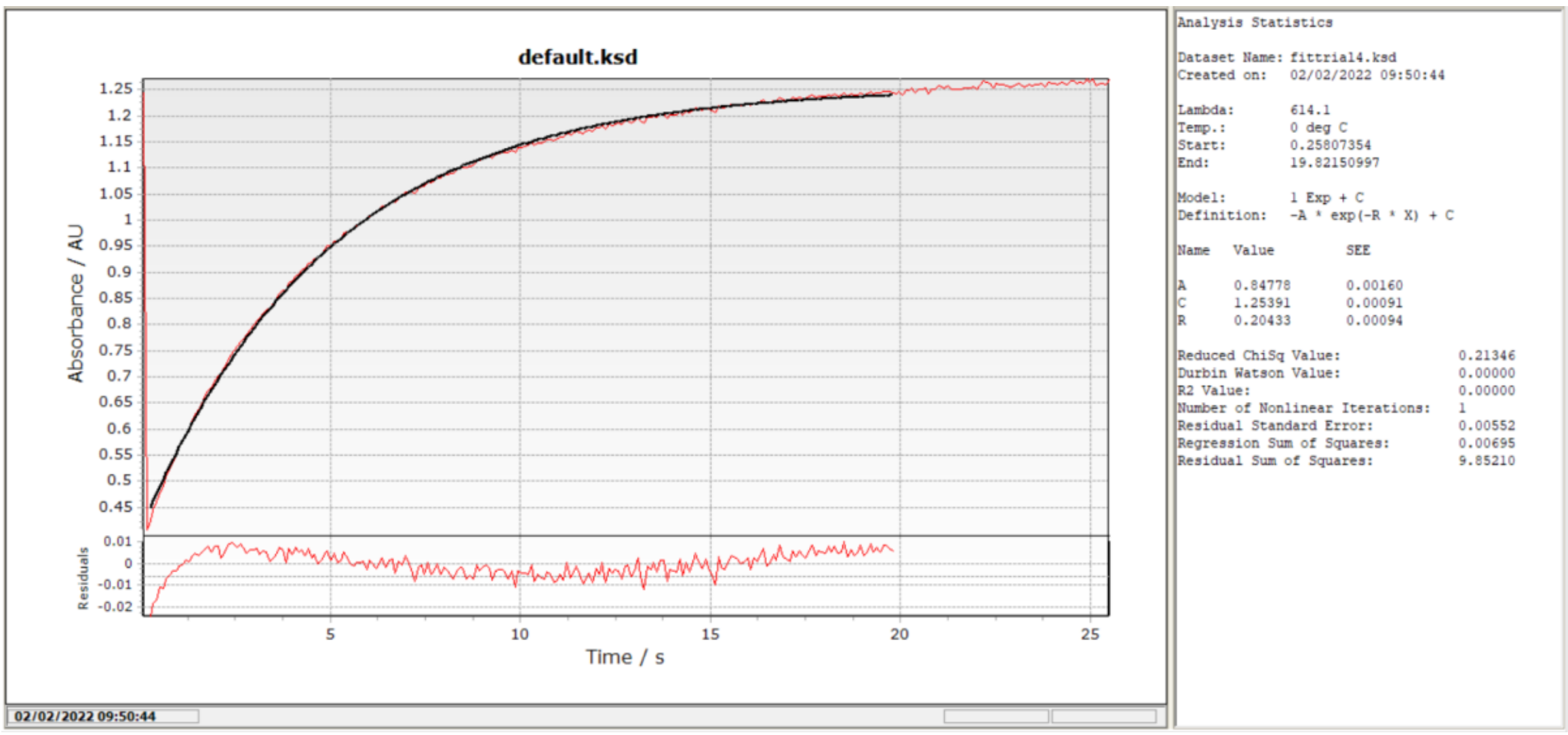

Figure S37. Exemplary trace (614 nm) and fit for the UV-Vis stopped-flow measurement of the reaction of **5a** (1 mM) with 56.25 mM CHD at 25 °C in MeCN.

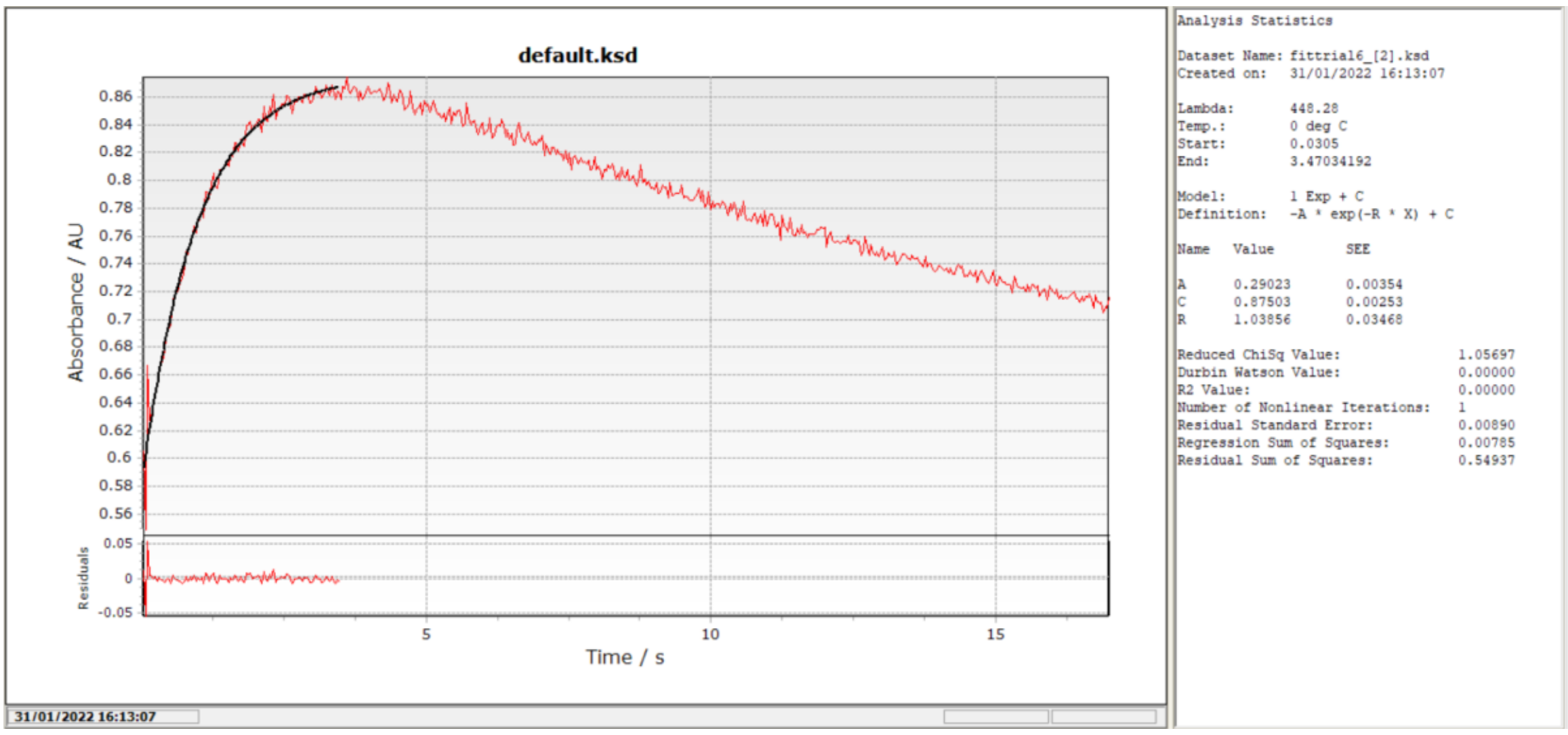

Figure S38. Exemplary trace (448 nm) and fit for the UV-Vis stopped-flow measurement of the reaction of **3** (1 mM) with 37.5 mM CHD at 25 °C in MeCN.

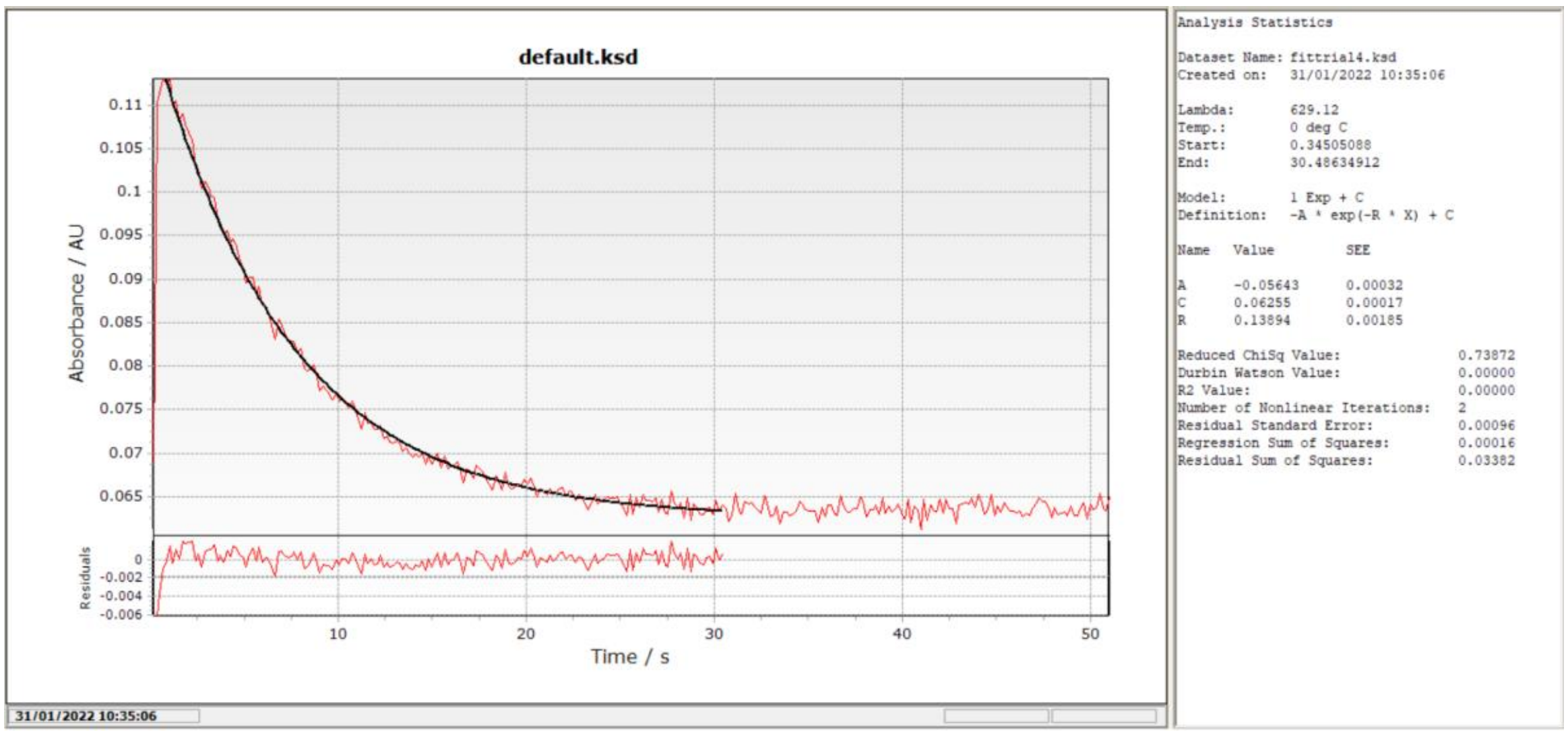

Figure S39. Exemplary trace (629 nm) and fit for the UV-Vis stopped-flow measurement of the reaction of **4** (1 mM) with 37.5 mM CHD at 25 °C in MeCN.

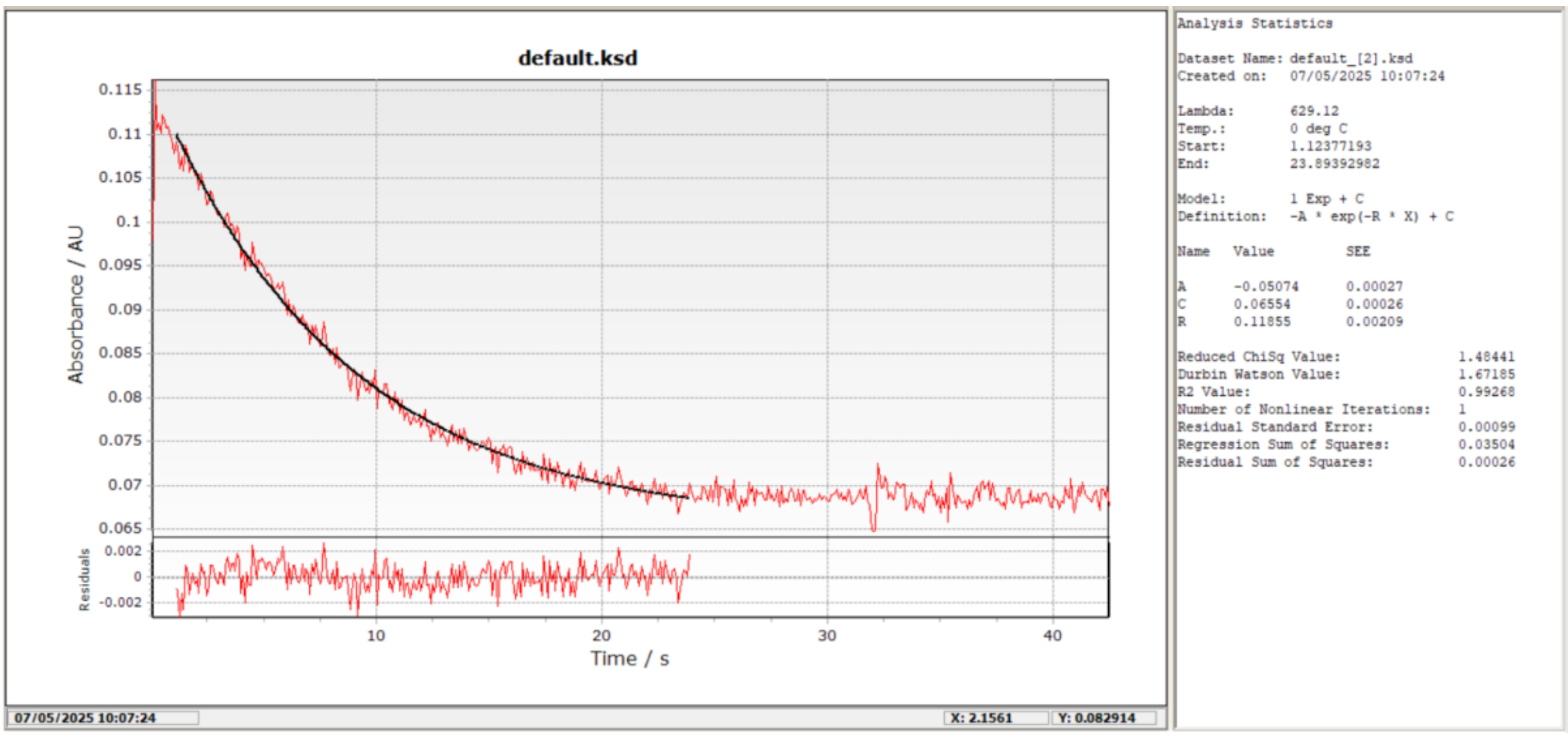

Figure S40. Exemplary trace (629 nm) and fit for the UV-Vis stopped-flow measurement of the reaction of **4** (1 mM) with additional 5 equivalents of NaTFA and 37.5 mM CHD at 25 °C in MeCN.

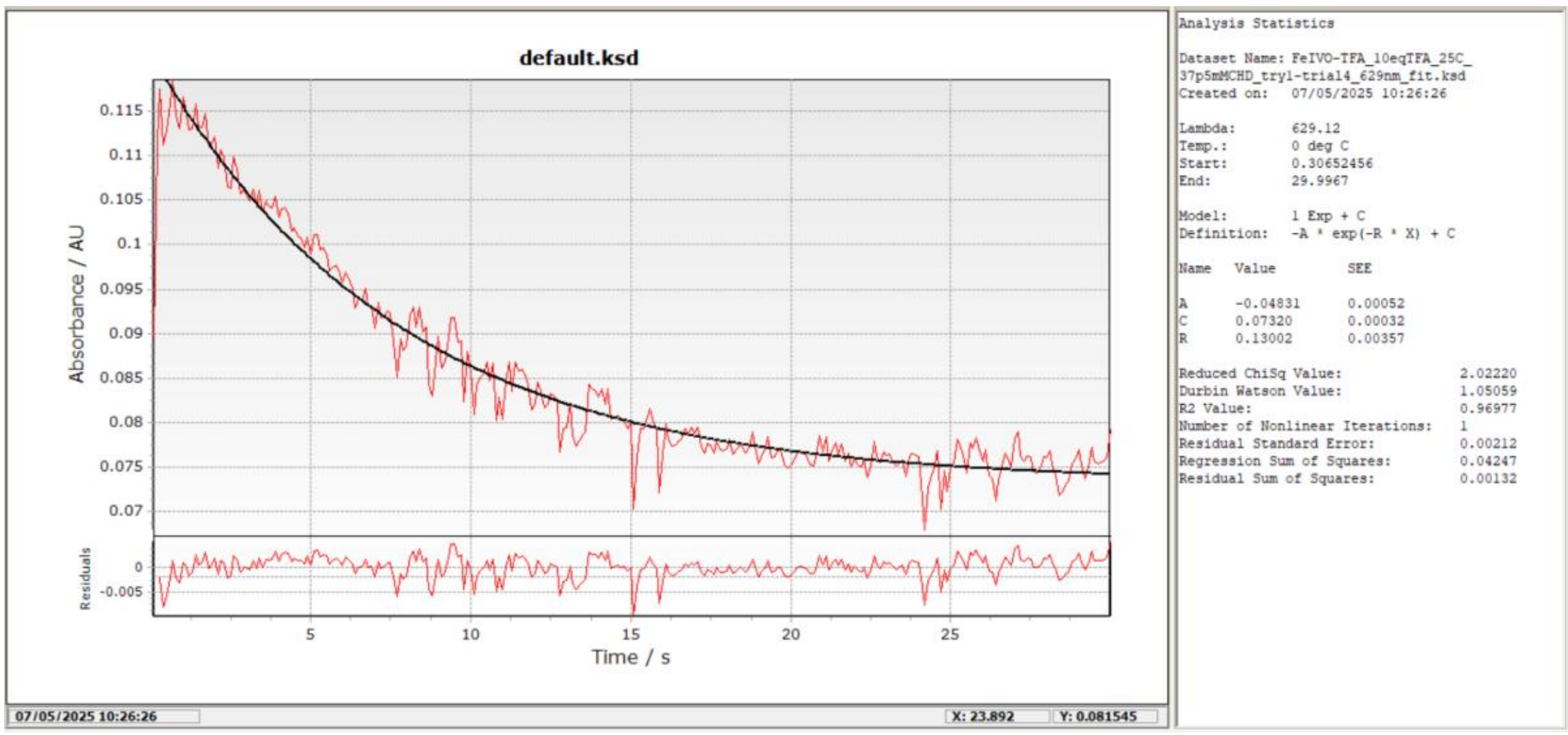

Figure S41. Exemplary trace (629 nm) and fit for the UV-Vis stopped-flow measurement of the reaction of **4** (1 mM) with additional 10 equivalents of NaTFA and 37.5 mM CHD at 25 °C in MeCN.

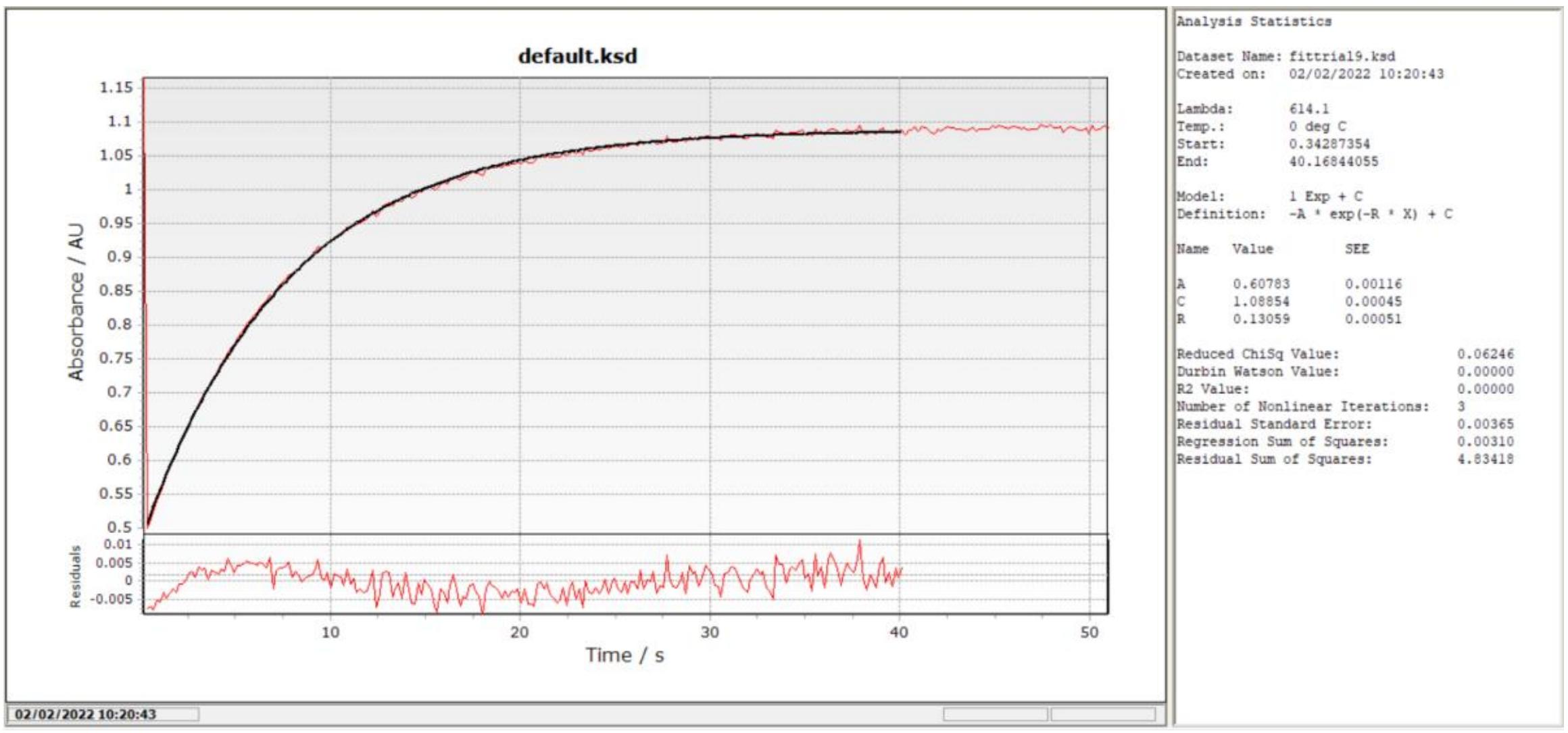

Figure S42. Exemplary trace (614 nm) and fit for the UV-Vis stopped-flow measurement of the reaction of **5a** (1 mM) with 37.5 mM CHD at 25 °C in MeCN.

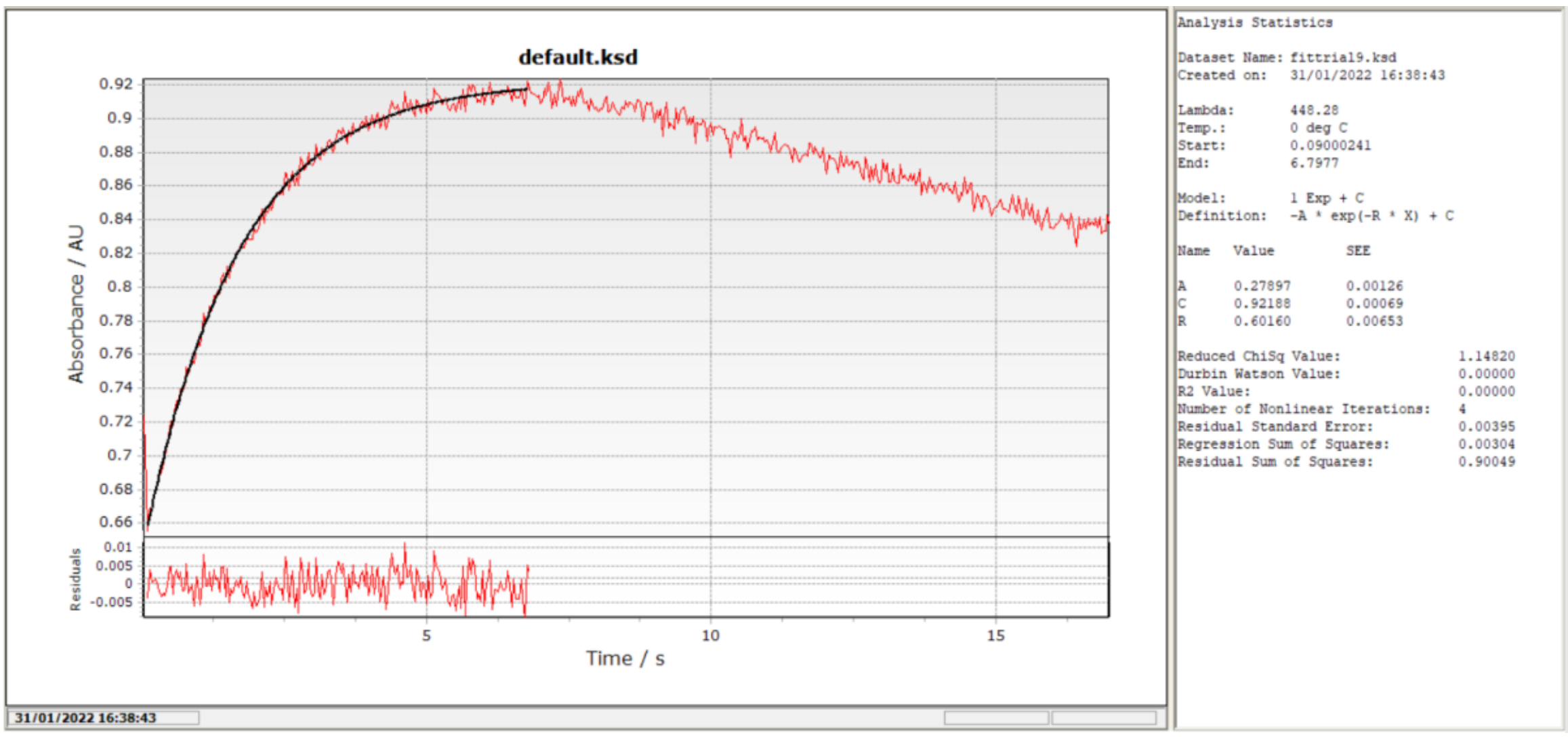

Figure S43. Exemplary trace (448 nm) and fit for the UV-Vis stopped-flow measurement of the reaction of **3** (1 mM) with 18.75 mM CHD at 25 °C in MeCN.

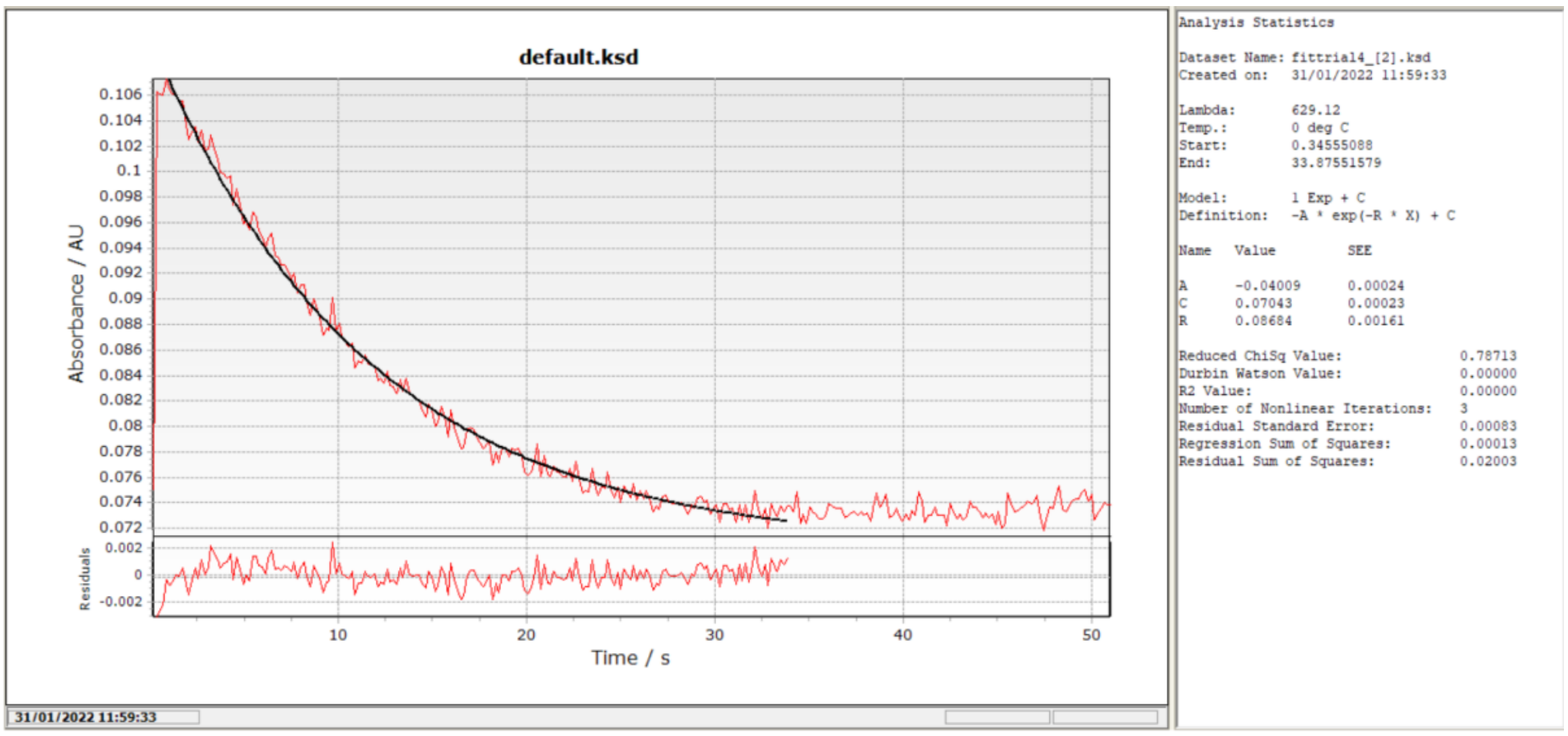

Figure S44. Exemplary trace (629 nm) and fit for the UV-Vis stopped-flow measurement of the reaction of **4** (1 mM) with 18.75 mM CHD at 25 °C in MeCN.

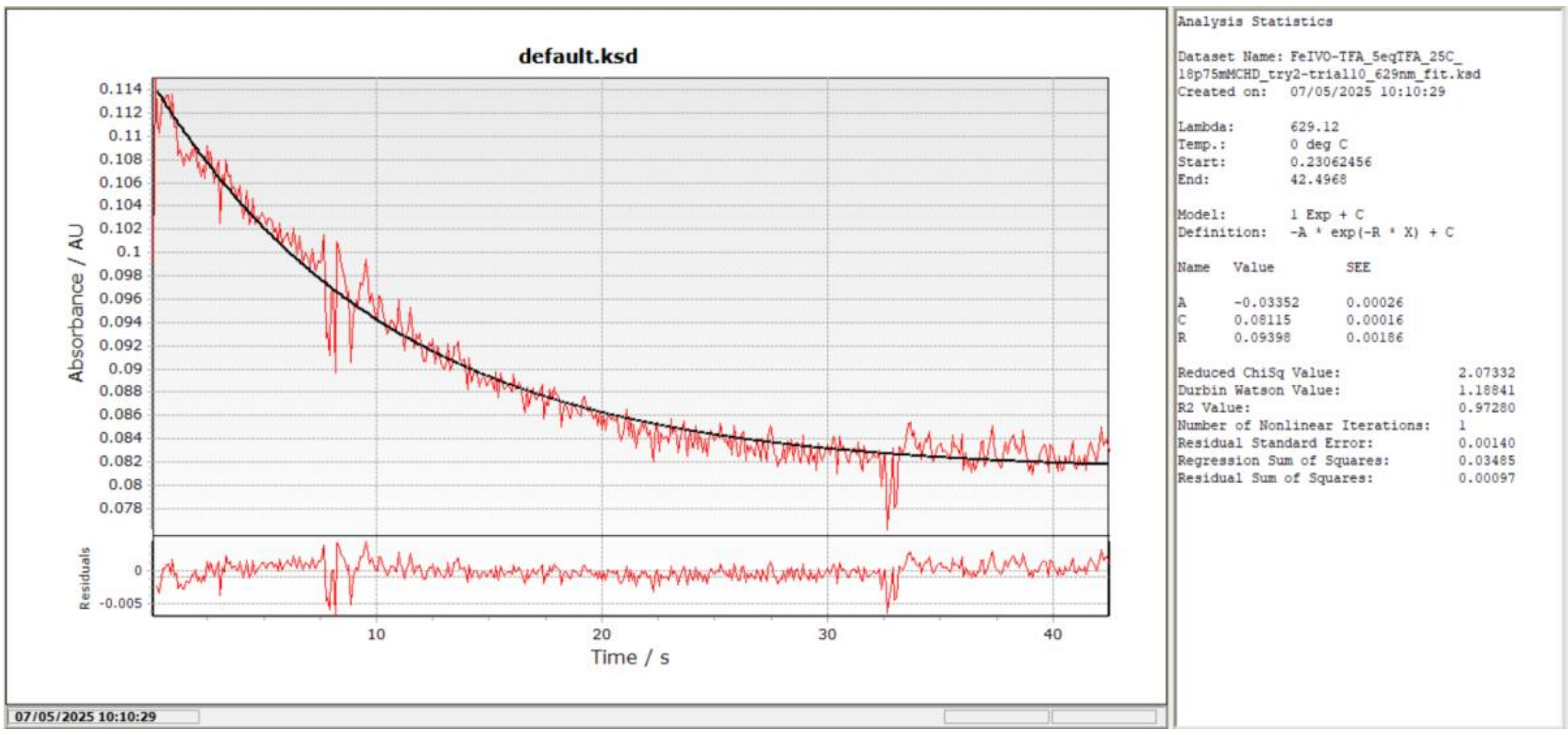

Figure S45. Exemplary trace (629 nm) and fit for the UV-Vis stopped-flow measurement of the reaction of **4** (1 mM) with additional 5 equivalents of NaTFA and 18.75 mM CHD at 25 °C in MeCN.

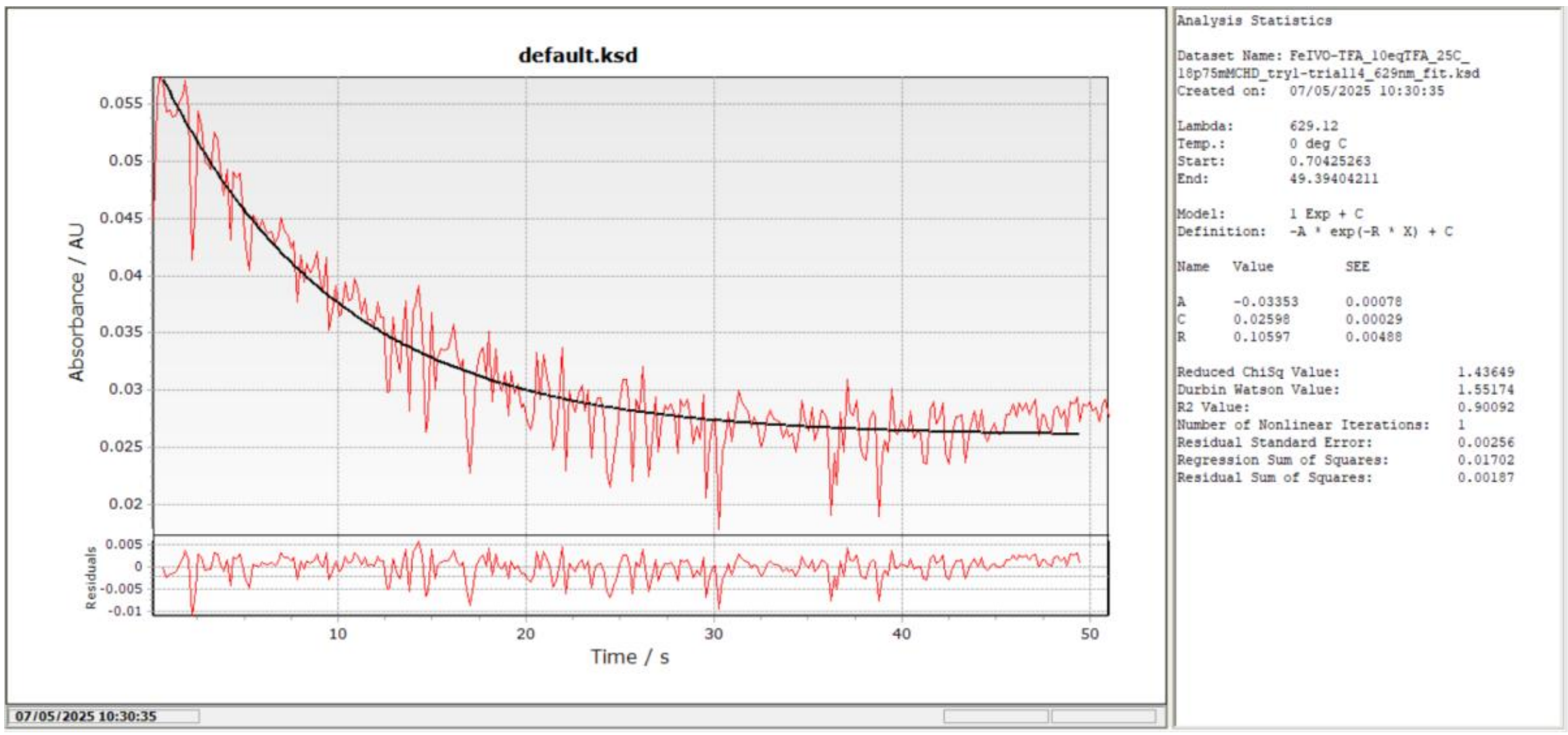

Figure S46. Exemplary trace (629 nm) and fit for the UV-Vis stopped-flow measurement of the reaction of **4** (1 mM) with additional 10 equivalents of NaTFA and 18.75 mM CHD at 25 °C in MeCN.

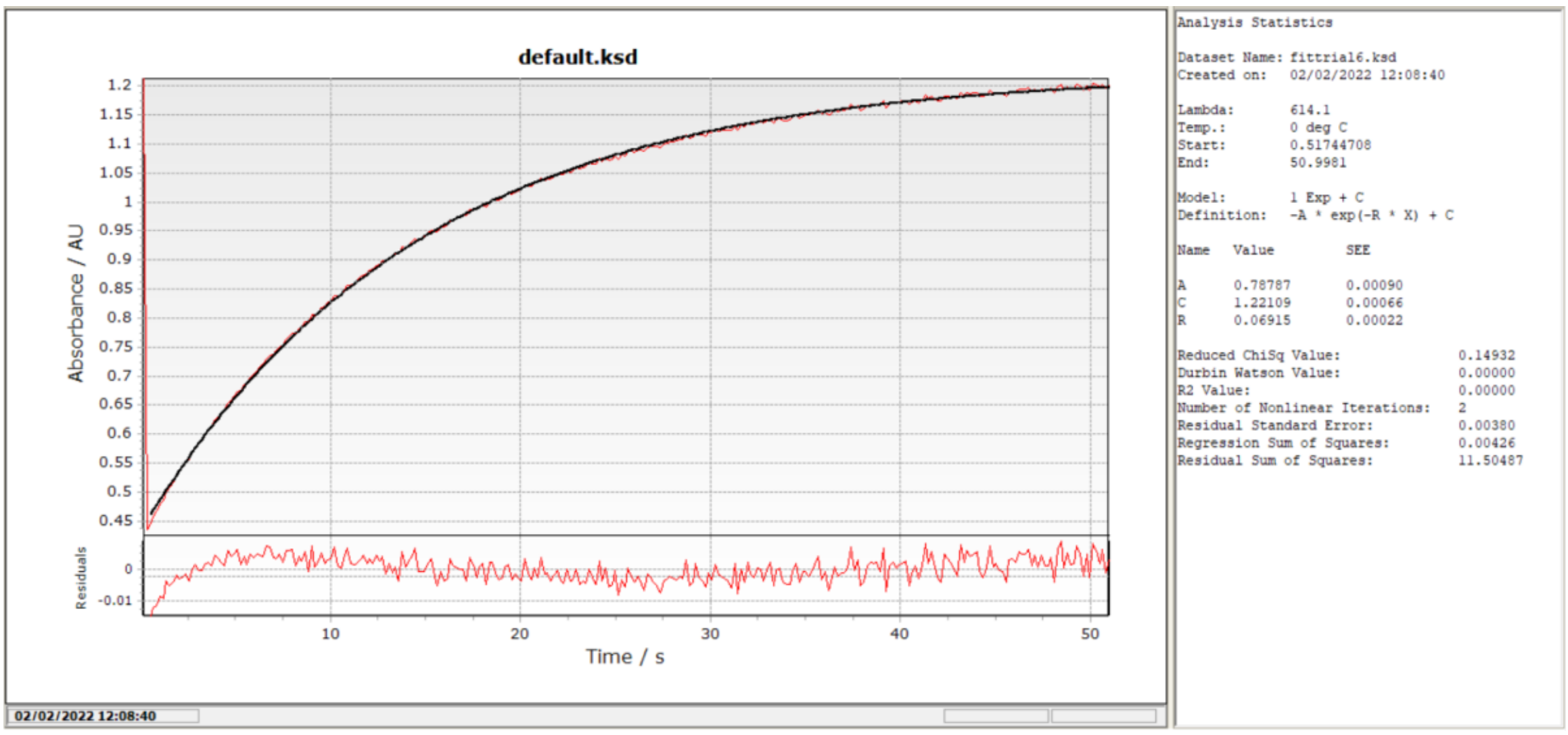

Figure S47. Exemplary trace (614 nm) and fit for the UV-Vis stopped-flow measurement of the reaction of **5a** (1 mM) with 18.75 mM CHD at 25 °C in MeCN.

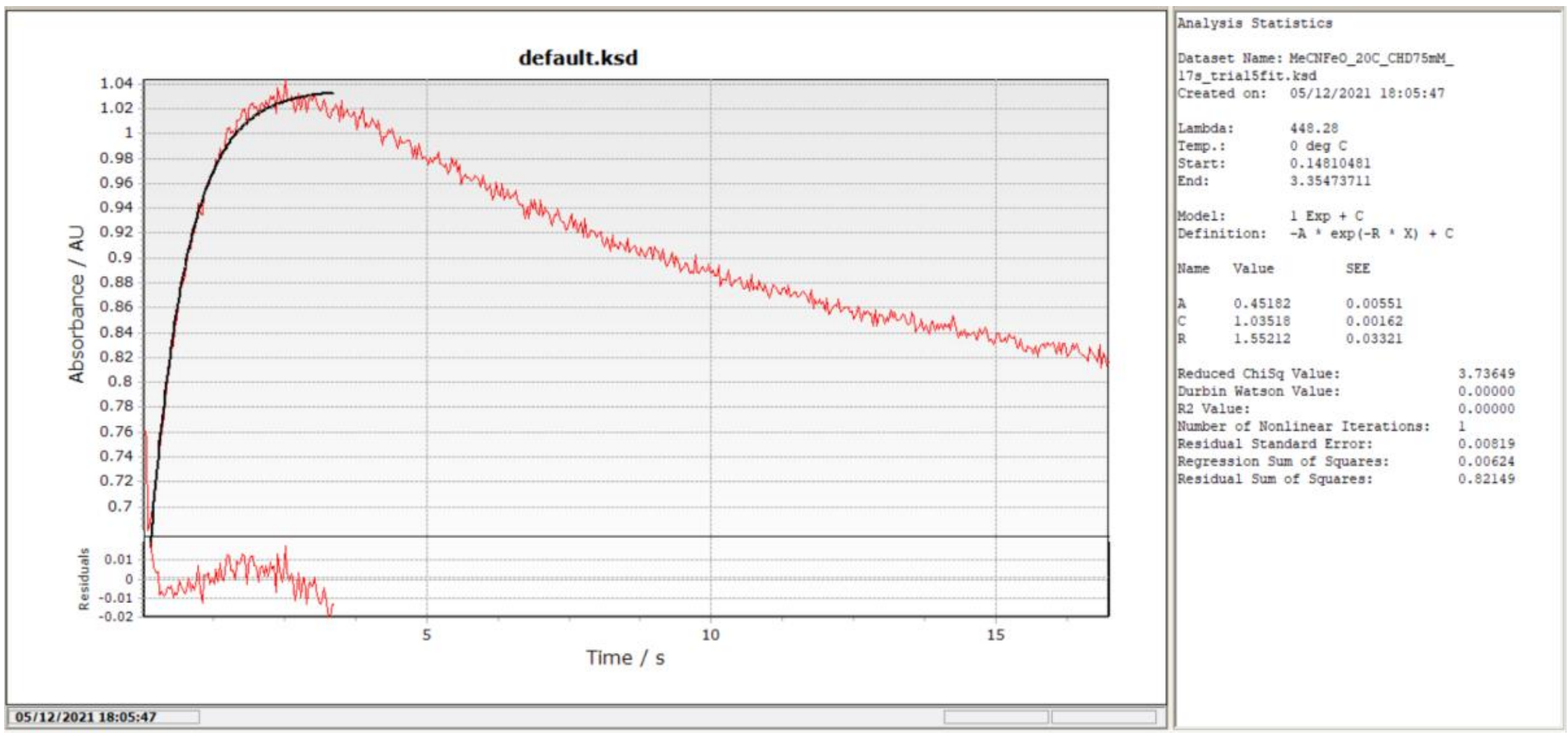

Figure S48. Exemplary trace (448 nm) and fit for the UV-Vis stopped-flow measurement of the reaction of **3** (1 mM) with 75 mM CHD at 20 °C in MeCN.

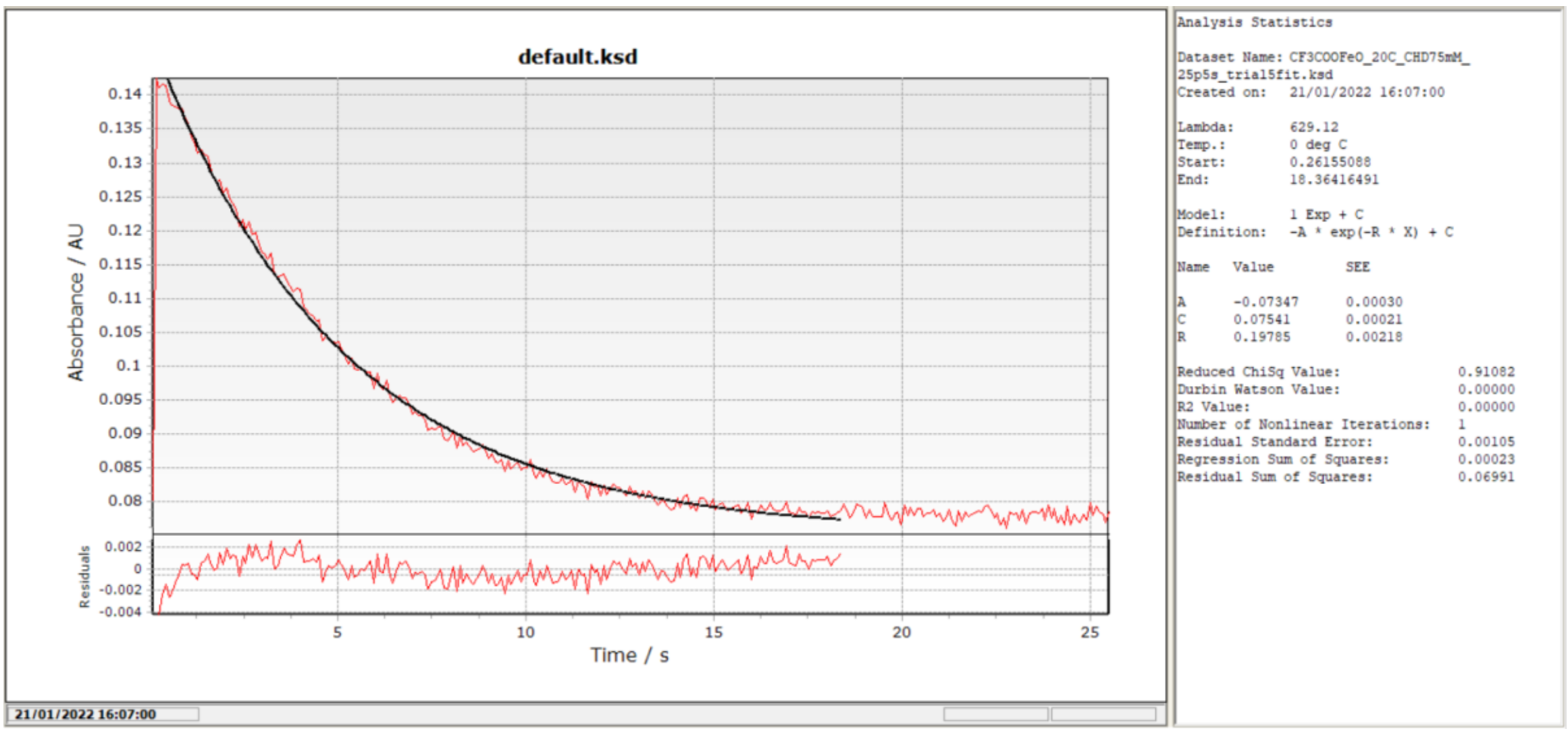

Figure S49. Exemplary trace (629 nm) and fit for the UV-Vis stopped-flow measurement of the reaction of **4** (1 mM) with 75 mM CHD at 20 °C in MeCN.

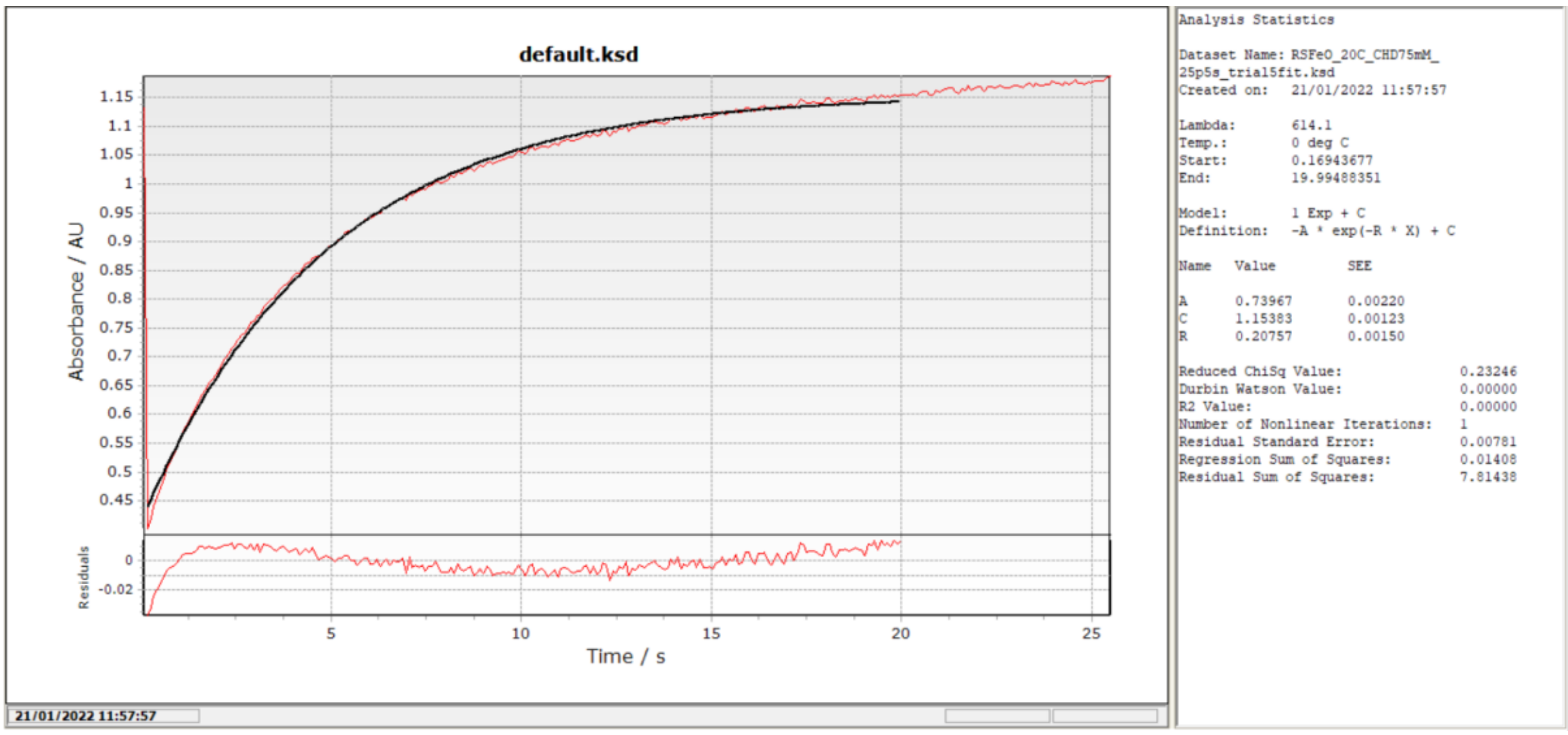

Figure S50. Exemplary trace (614 nm) and fit for the UV-Vis stopped-flow measurement of the reaction of **5a** (1 mM) with 75 mM CHD at 20 °C in MeCN.

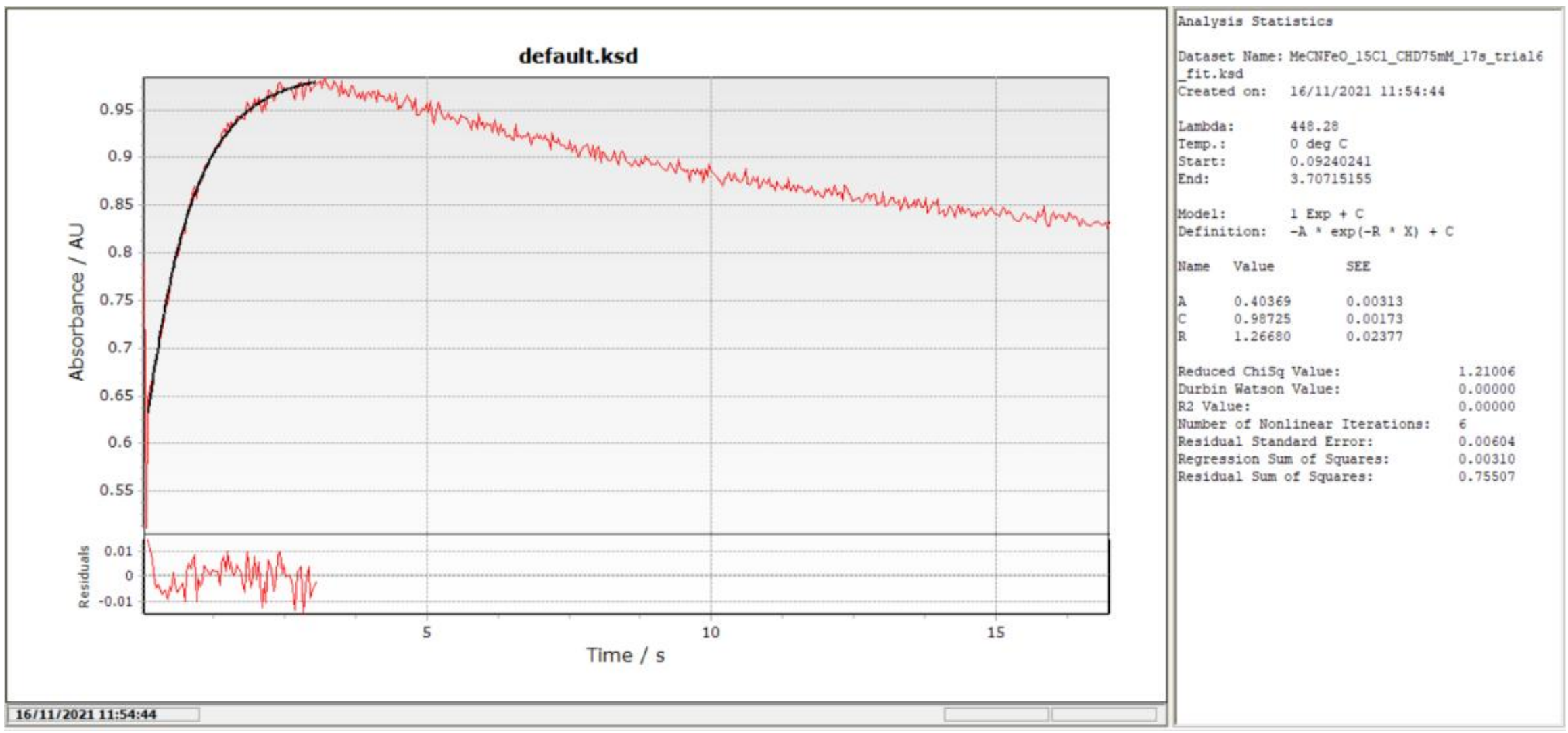

Figure S51. Exemplary trace (448 nm) and fit for the UV-Vis stopped-flow measurement of the reaction of **3** (1 mM) with 75 mM CHD at 15 °C in MeCN.

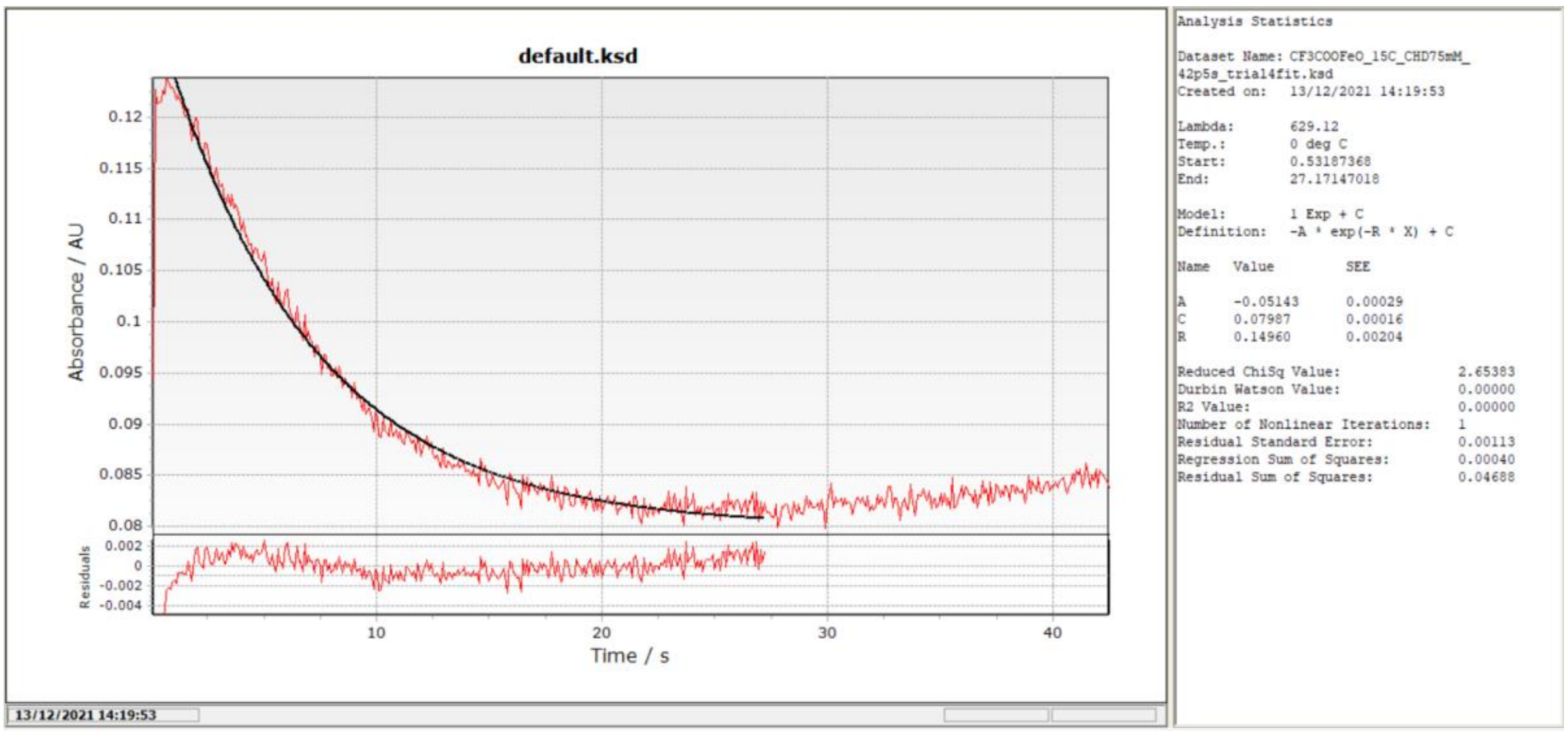

Figure S52. Exemplary trace (629 nm) and fit for the UV-Vis stopped-flow measurement of the reaction of **4** (1 mM) with 75 mM CHD at 15 °C in MeCN.

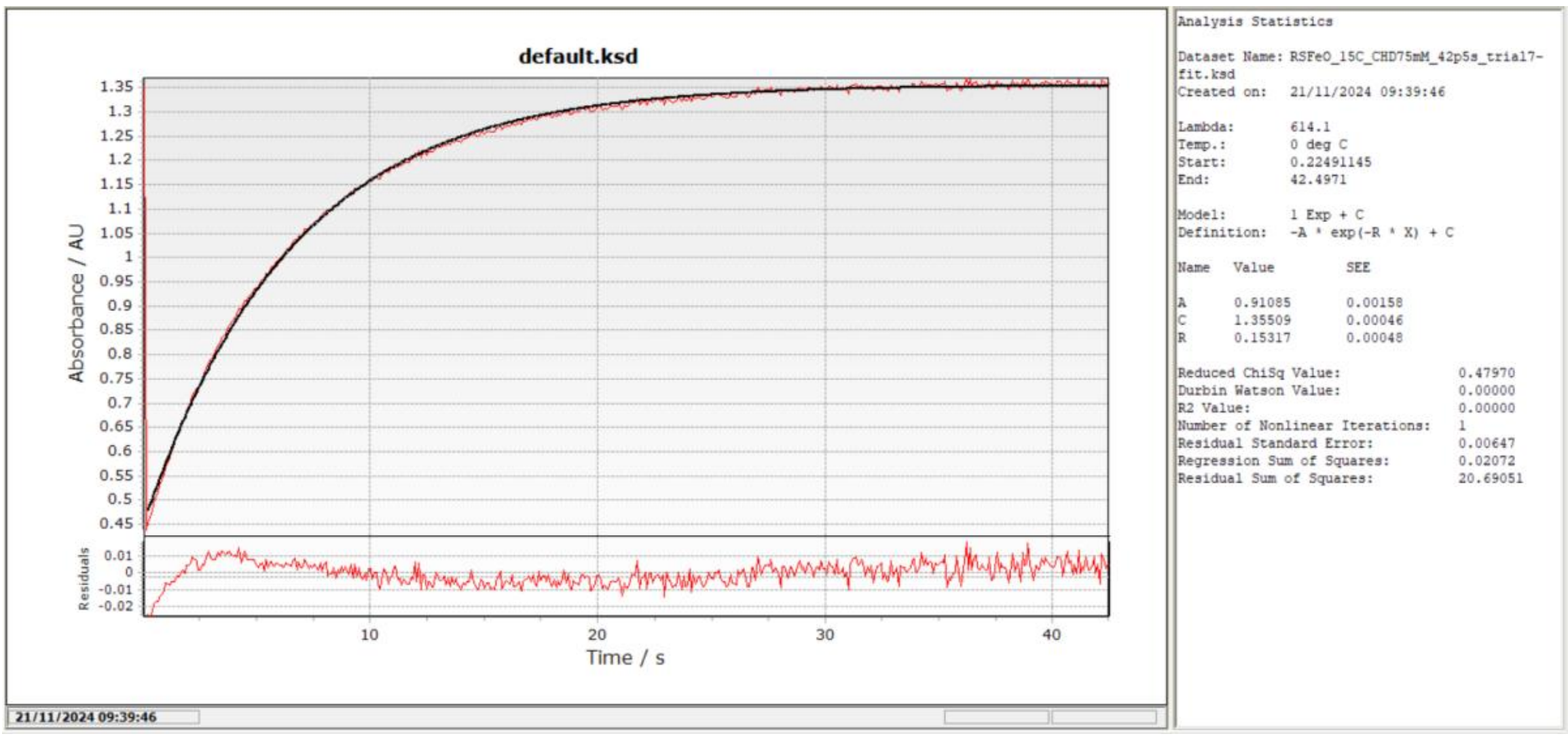

Figure S53. Exemplary trace (614 nm) and fit for the UV-Vis stopped-flow measurement of the reaction of **5a** (1 mM) with 75 mM CHD at 15 °C in MeCN.

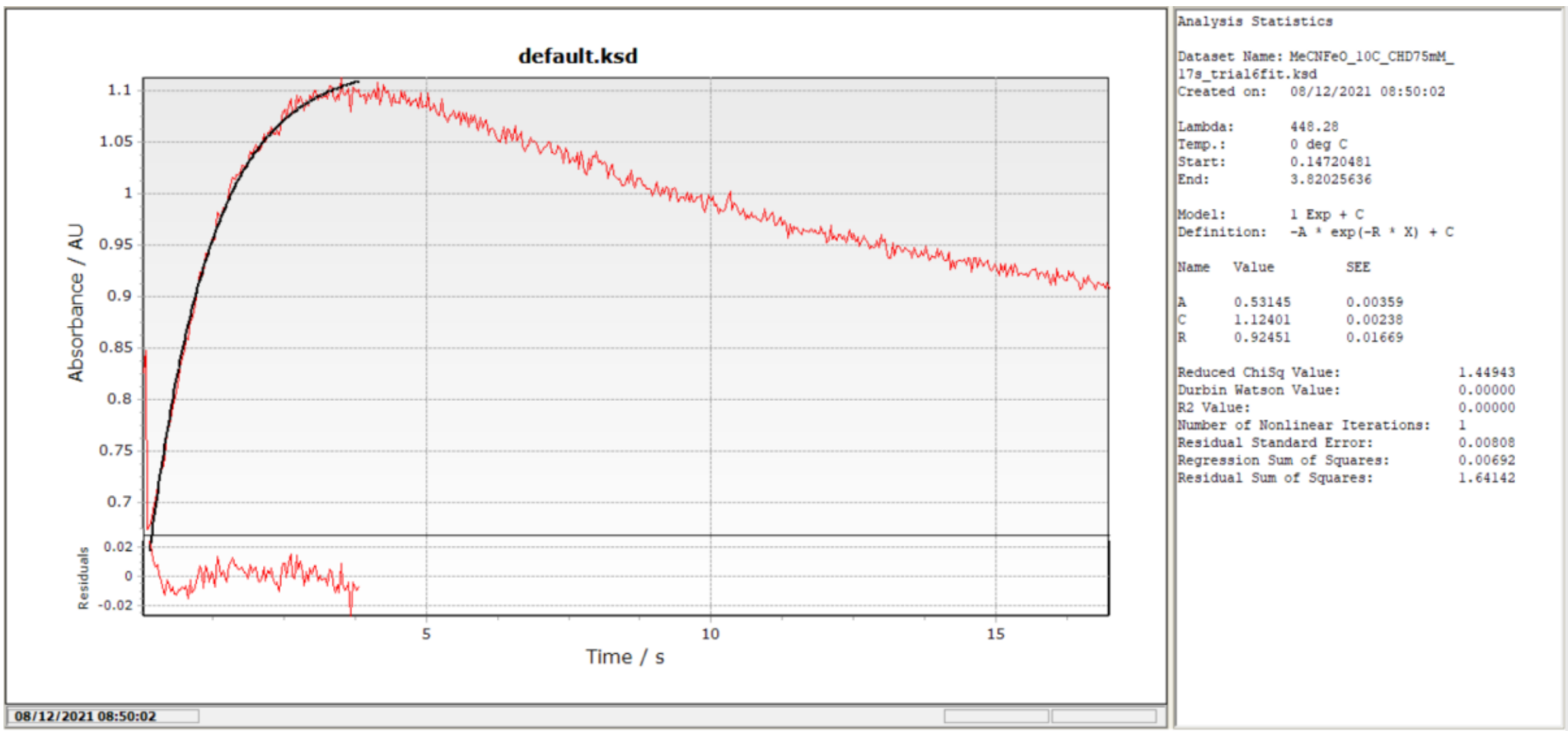

Figure S54. Exemplary trace (448 nm) and fit for the UV-Vis stopped-flow measurement of the reaction of **3** (1 mM) with 75 mM CHD at 10 °C in MeCN.

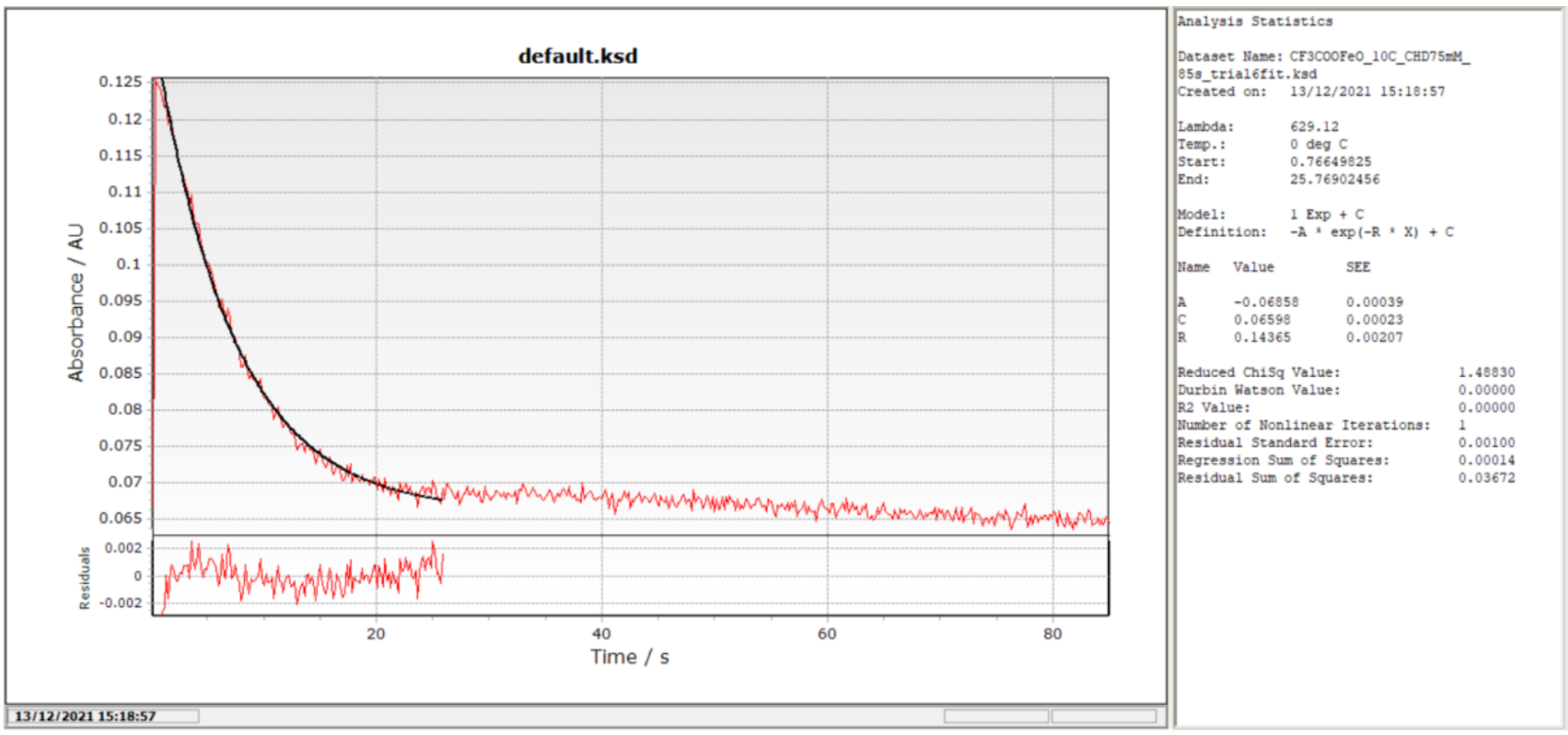

Figure S55. Exemplary trace (629 nm) and fit for the UV-Vis stopped-flow measurement of the reaction of **4** (1 mM) with 75 mM CHD at 10 °C in MeCN.

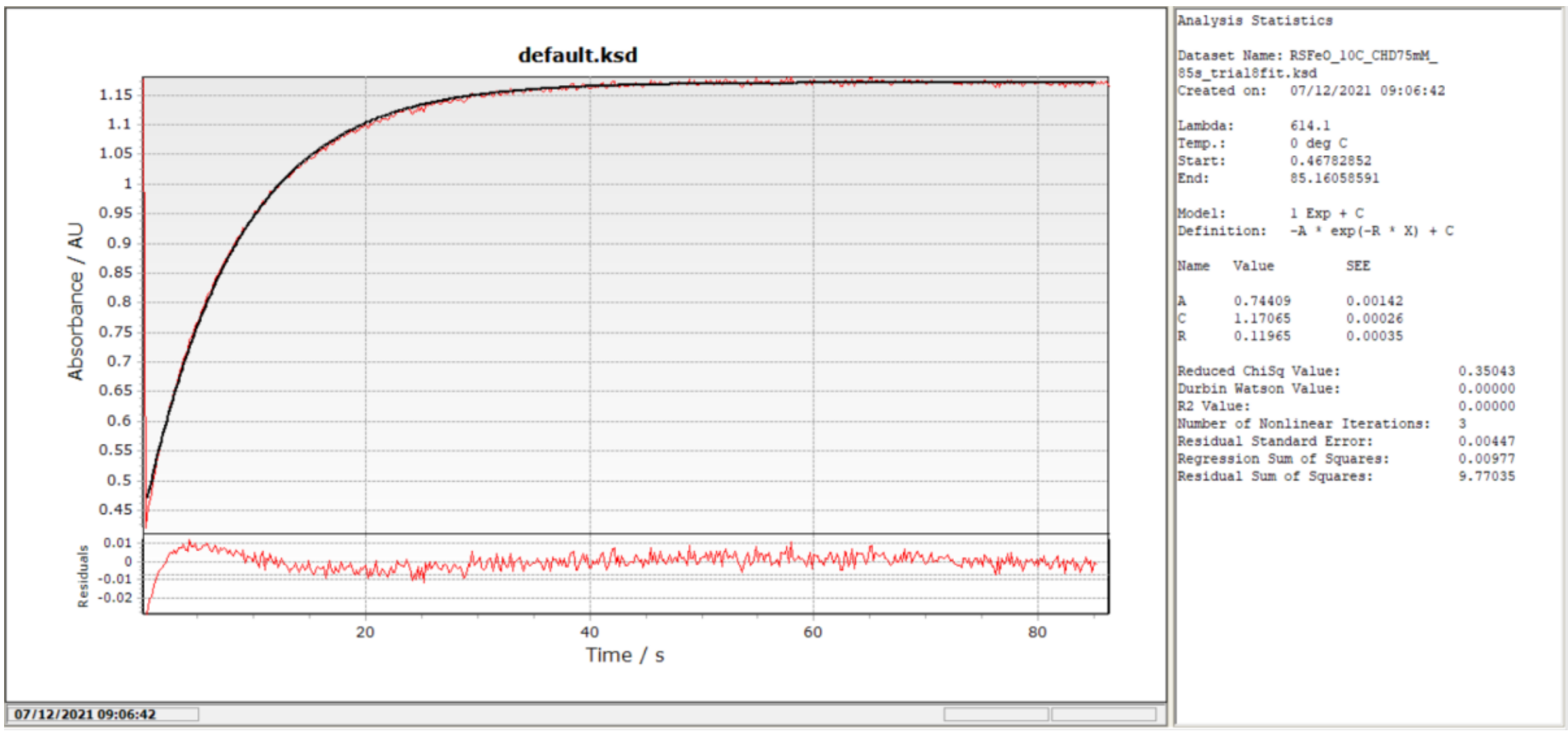

Figure S56. Exemplary trace (614 nm) and fit for the UV-Vis stopped-flow measurement of the reaction of **5a** (1 mM) with 75 mM CHD at 10 °C in MeCN.

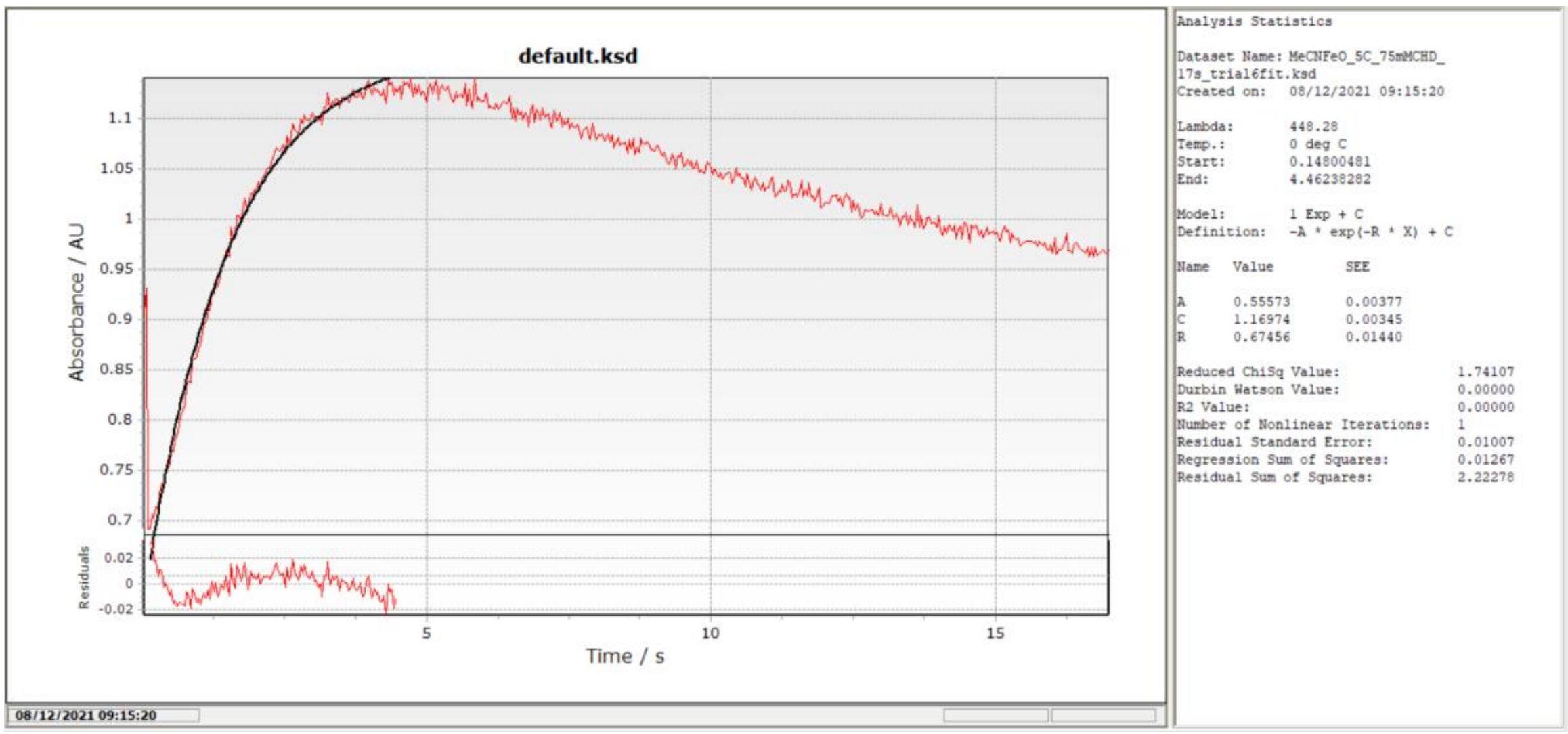

Figure S57. Exemplary trace (448 nm) and fit for the UV-Vis stopped-flow measurement of the reaction of **3** (1 mM) with 75 mM CHD at 5 °C in MeCN.

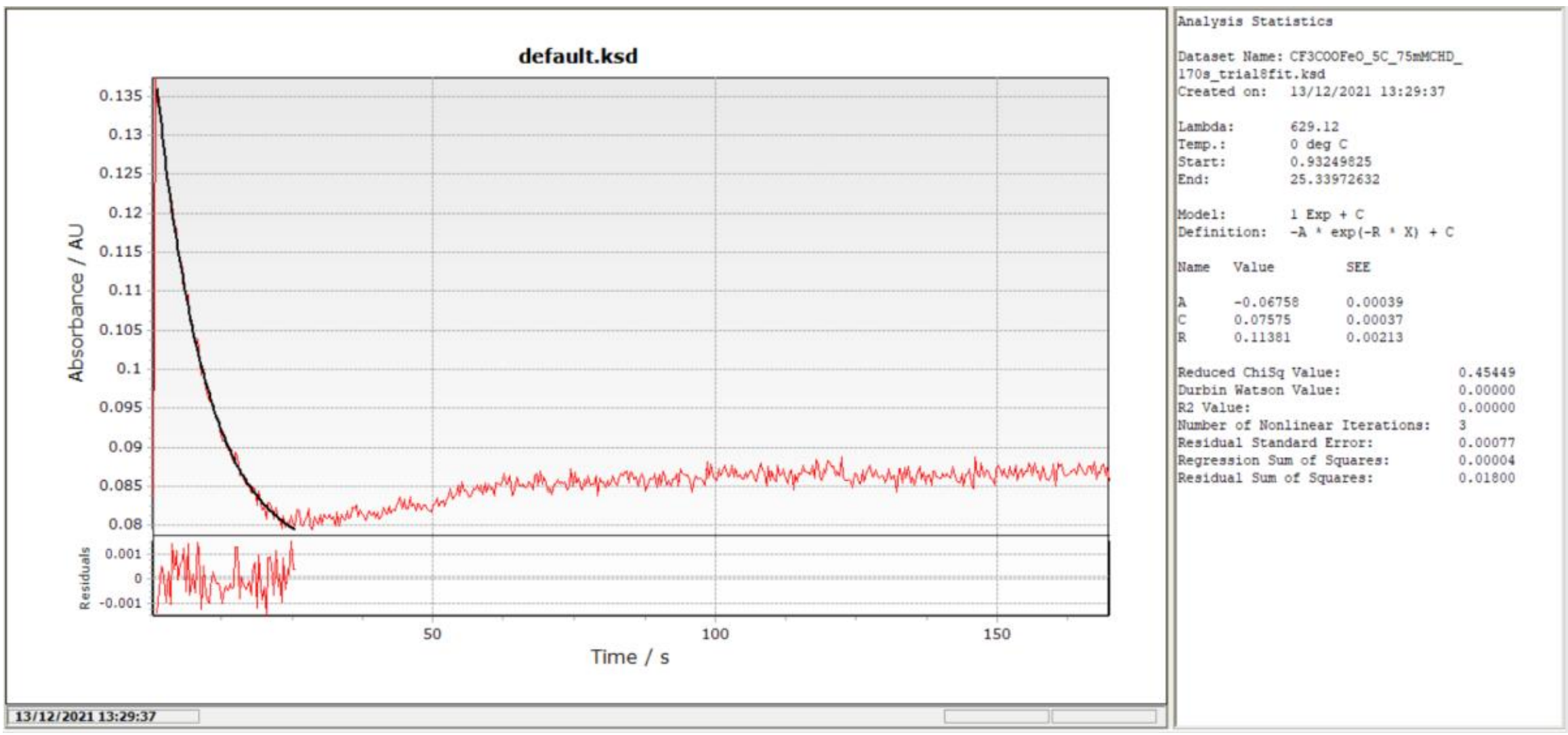

Figure S58. Exemplary trace (629 nm) and fit for the UV-Vis stopped-flow measurement of the reaction of **4** (1 mM) with 75 mM CHD at 5 °C in MeCN.

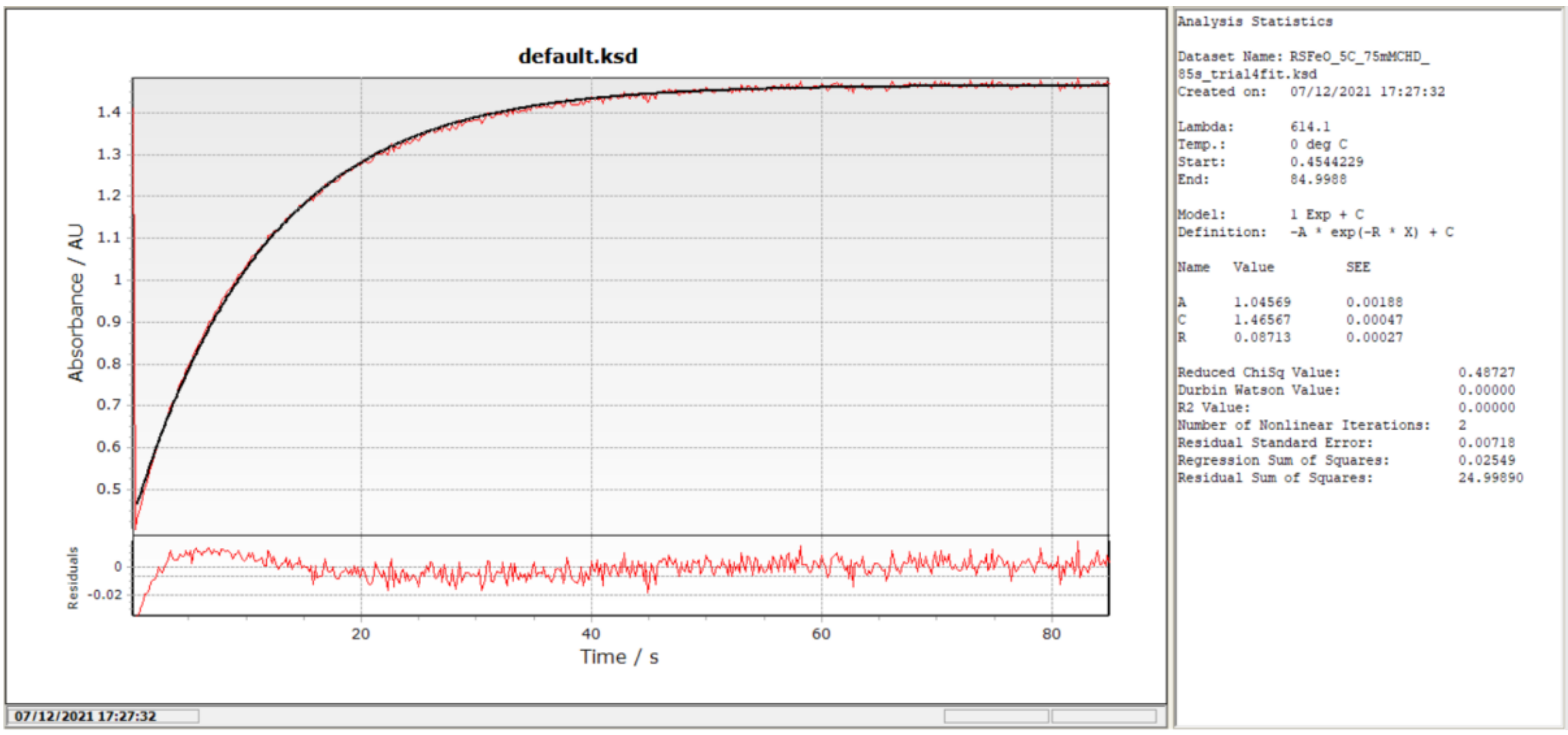

Figure S59. Exemplary trace (614 nm) and fit for the UV-Vis stopped-flow measurement of the reaction of **5a** (1 mM) with 75 mM CHD at 5 °C in MeCN.

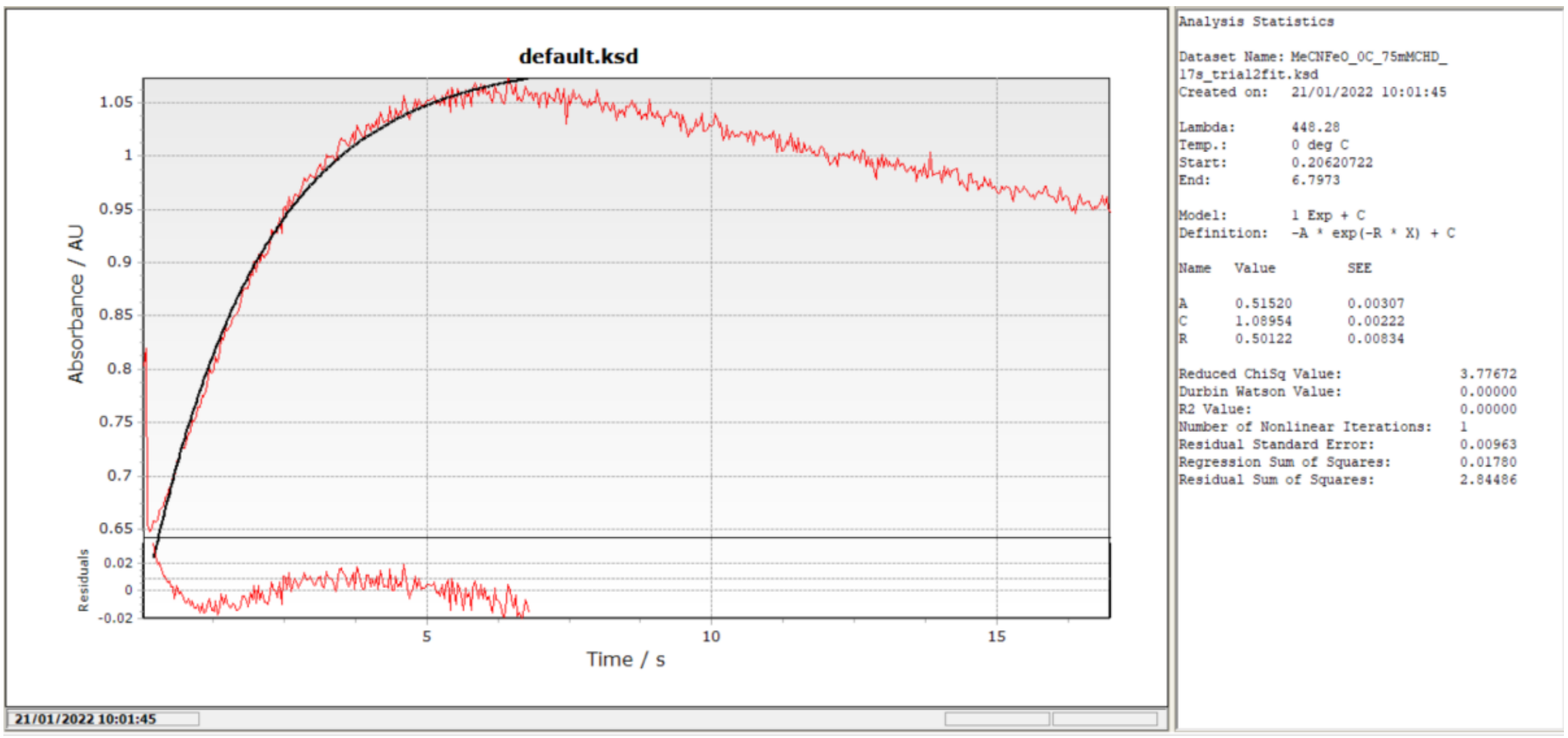

Figure S60. Exemplary trace (448 nm) and fit for the UV-Vis stopped-flow measurement of the reaction of **3** (1 mM) with 75 mM CHD at 0 °C in MeCN.

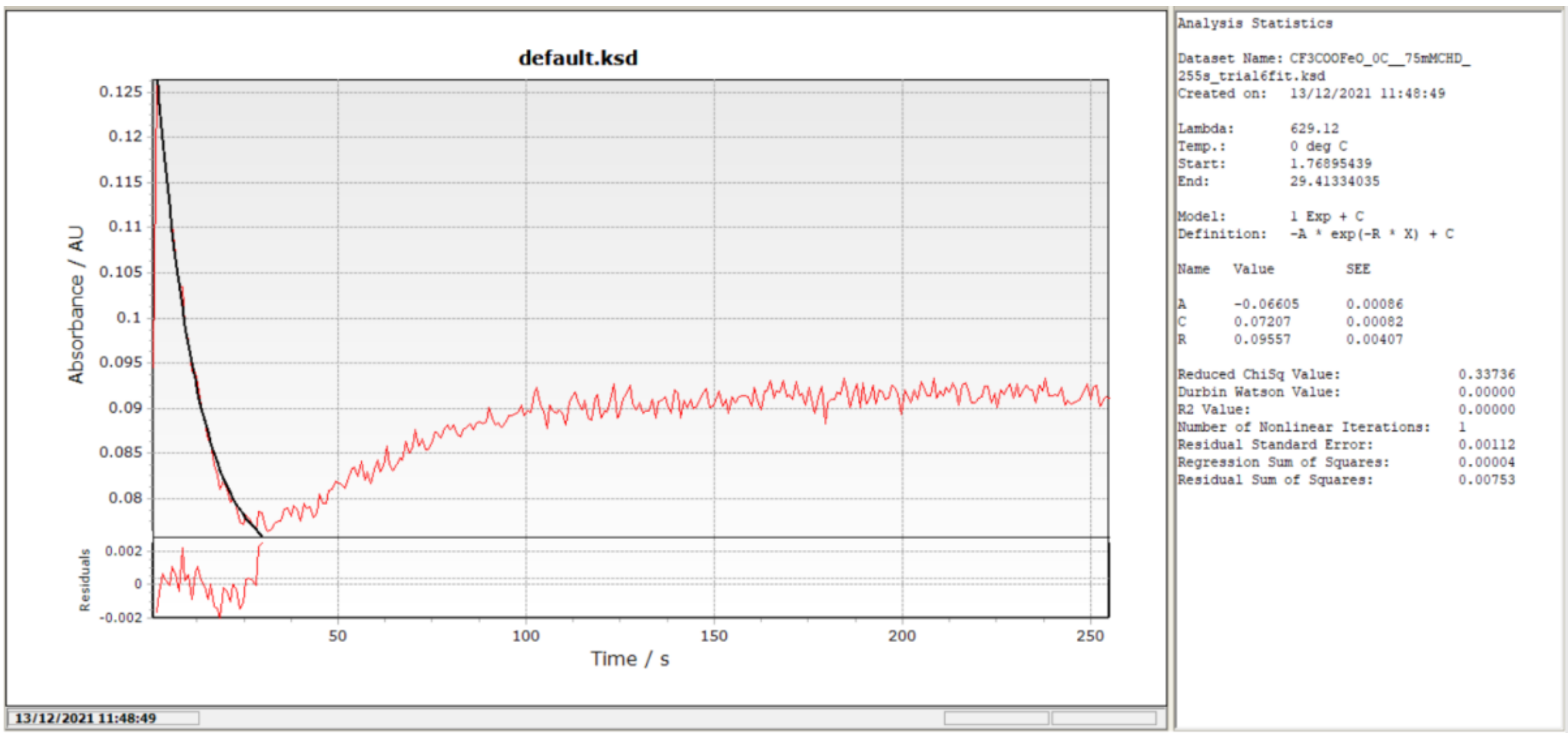

Figure S61. Exemplary trace (629 nm) and fit for the UV-Vis stopped-flow measurement of the reaction of **4** (1 mM) with 75 mM CHD at 0 °C in MeCN.

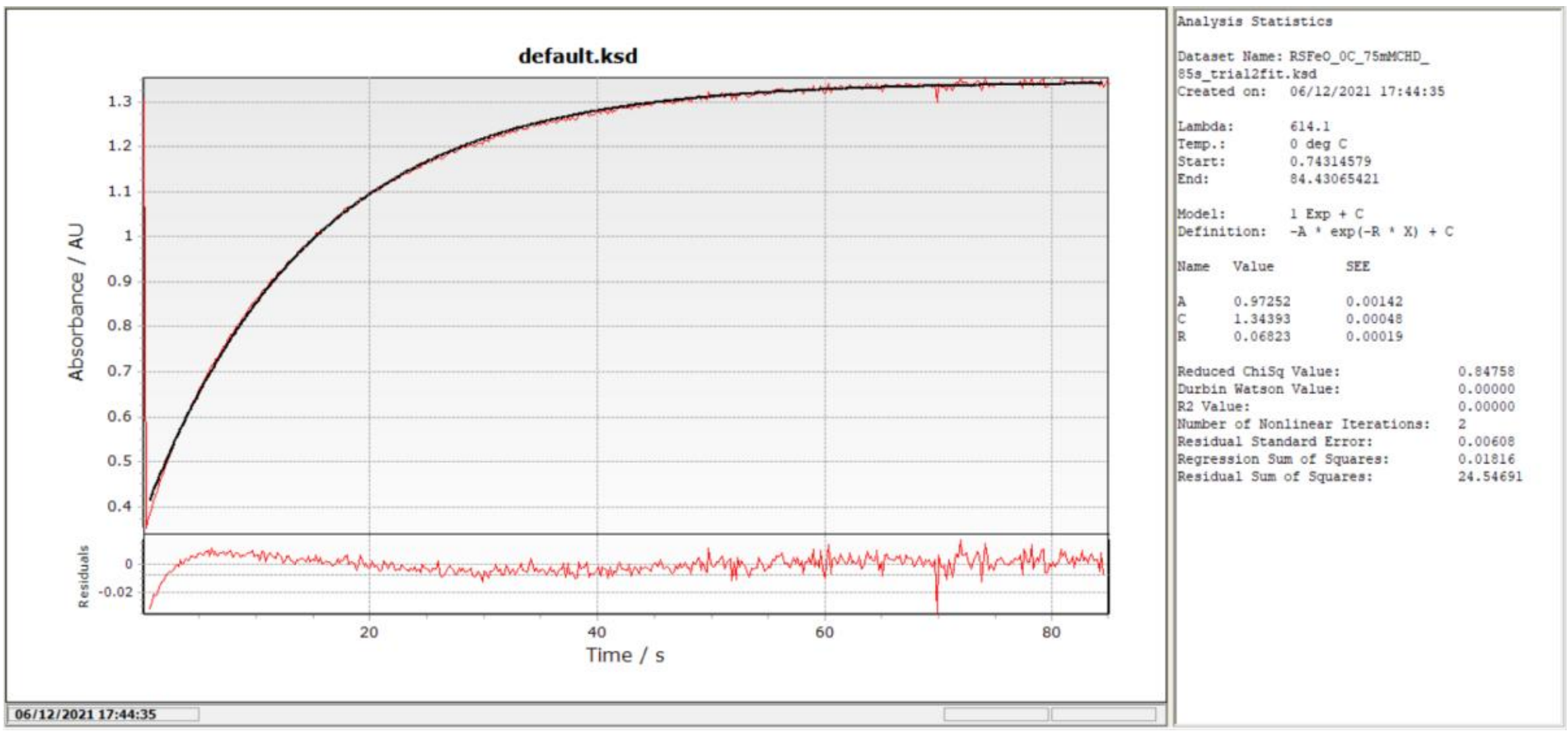

Figure S62. Exemplary trace (614 nm) and fit for the UV-Vis stopped-flow measurement of the reaction of **5a** (1 mM) with 75 mM CHD at 0 °C in MeCN.

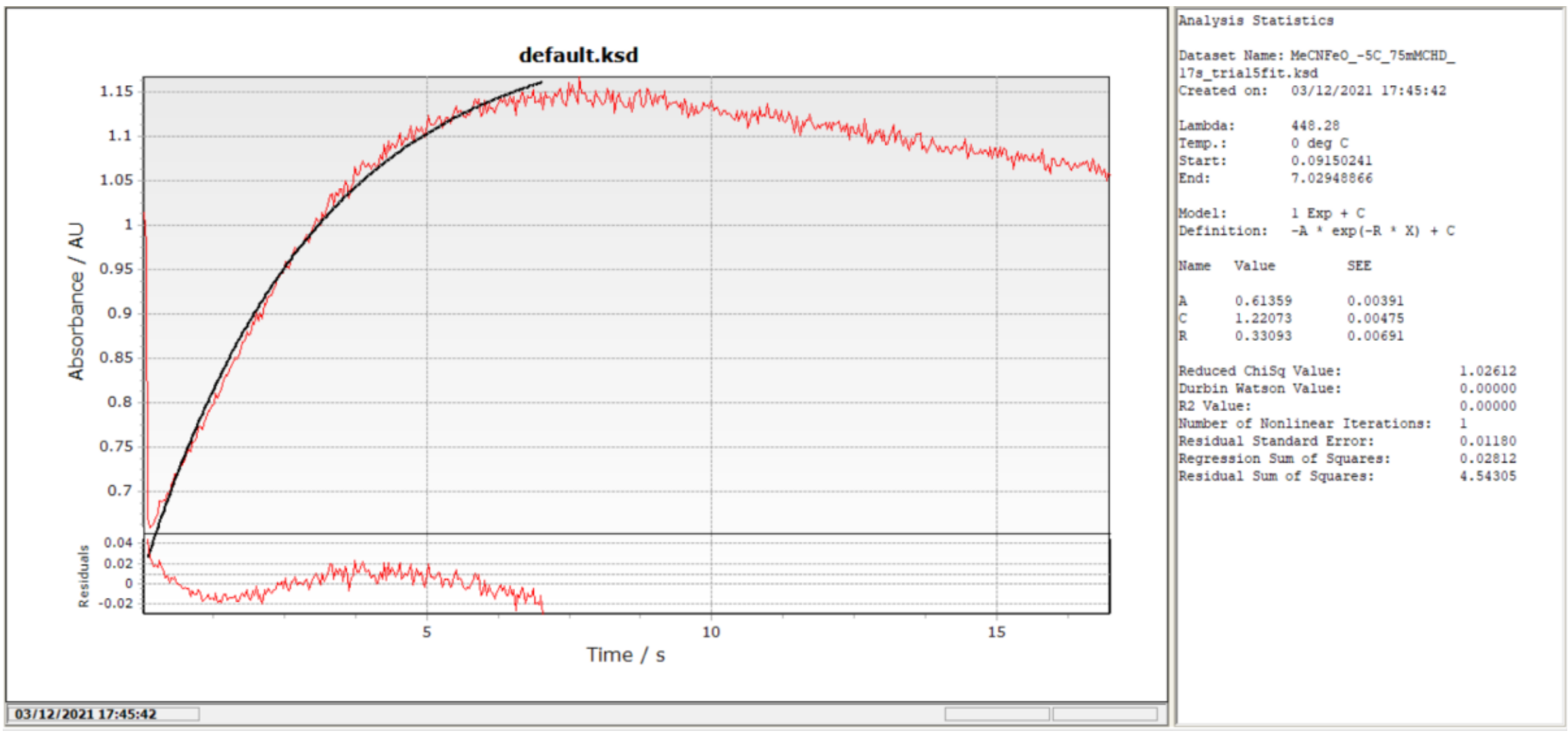

Figure S63. Exemplary trace (448 nm) and fit for the UV-Vis stopped-flow measurement of the reaction of **3** (1 mM) with 75 mM CHD at −5 °C in MeCN.

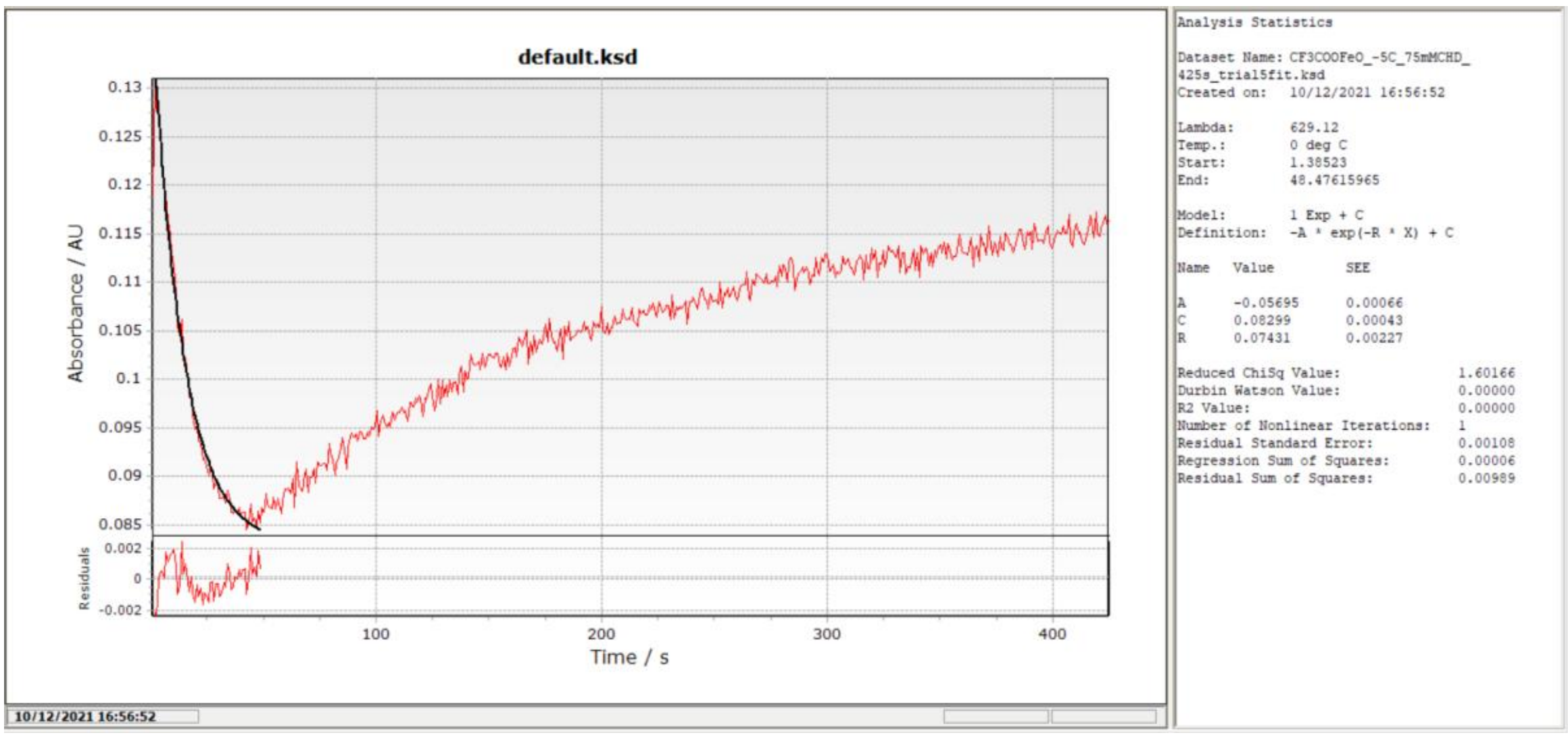

Figure S64. Exemplary trace (629 nm) and fit for the UV-Vis stopped-flow measurement of the reaction of **4** (1 mM) with 75 mM CHD at −5 °C in MeCN.

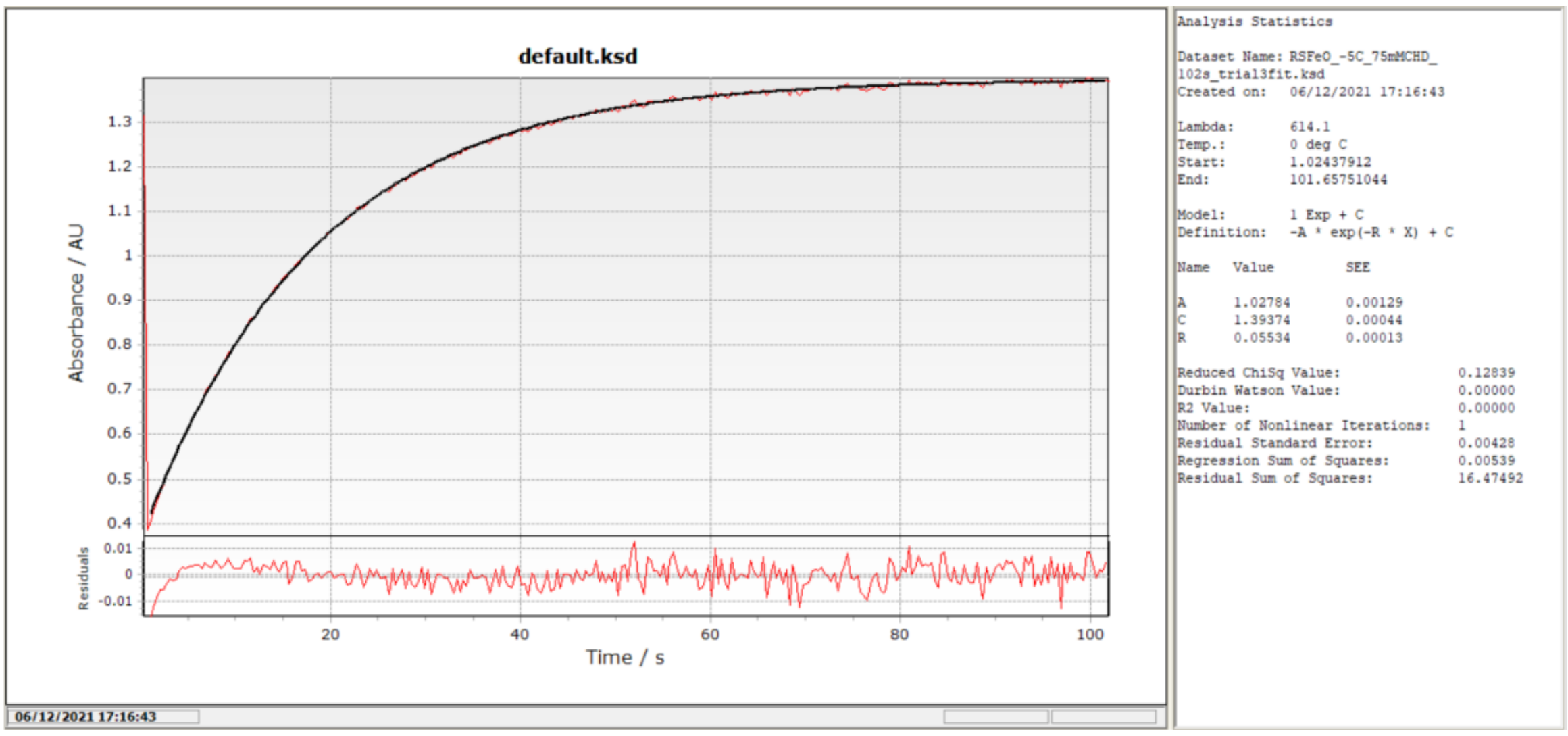

Figure S65. Exemplary trace (614 nm) and fit for the UV-Vis stopped-flow measurement of the reaction of **5a** (1 mM) with 75 mM CHD at −5 °C in MeCN.

Kinetic stopped-flow measurements with DHA:

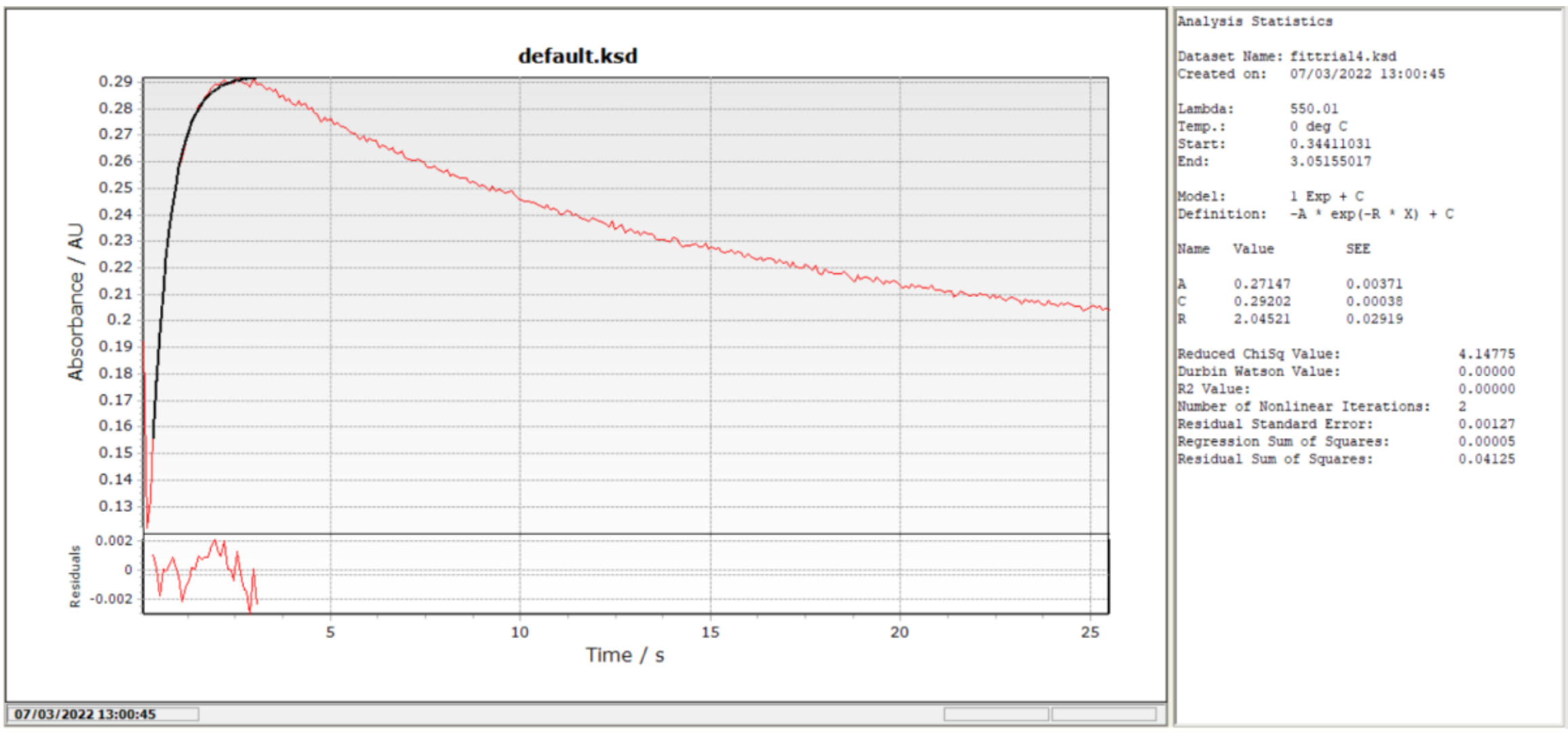

Figure S66. Exemplary trace (550 nm) and fit for the UV-Vis stopped-flow measurement of the reaction of **3** (1 mM) with 56.25 mM DHA at 25 °C in a mixture of MeCN and DCM.

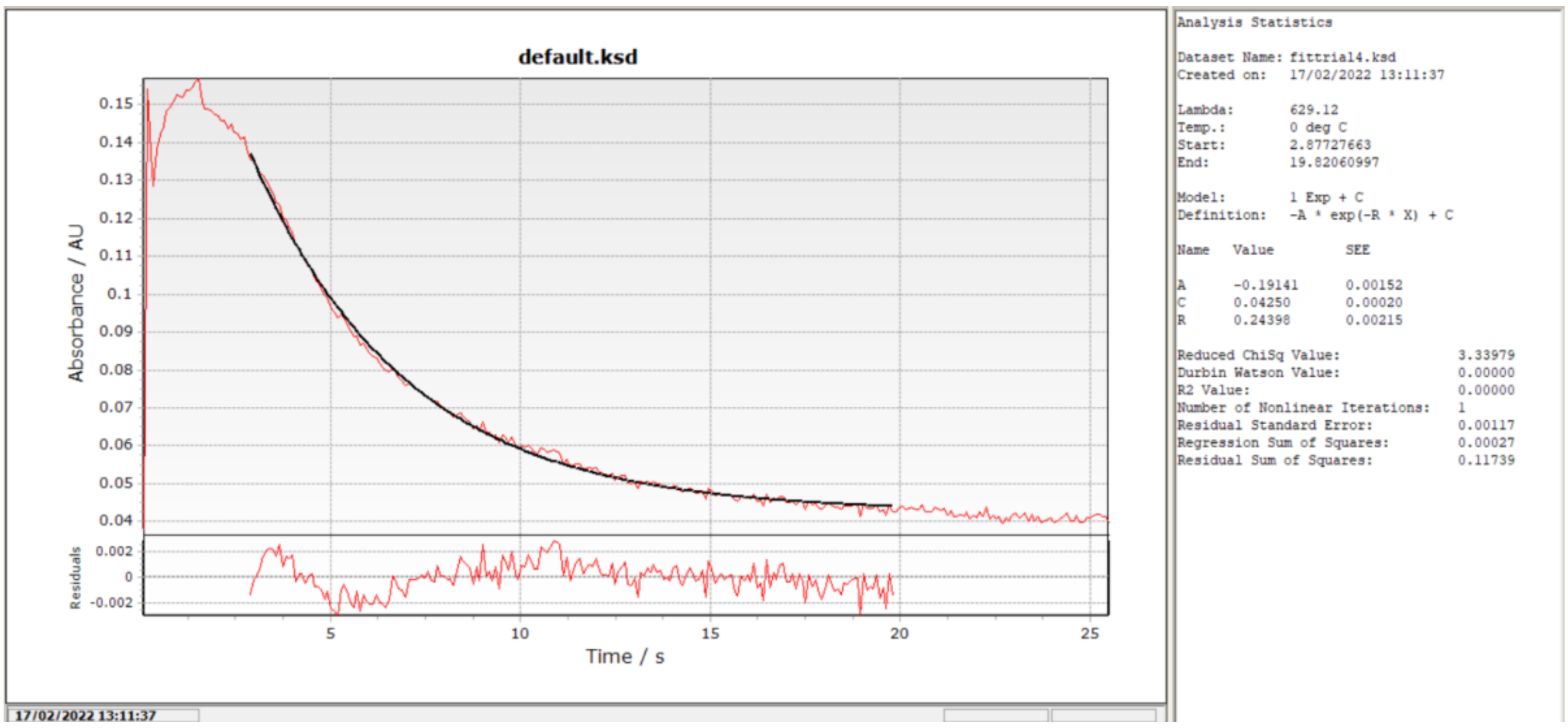

Figure S67. Exemplary trace (629 nm) and fit for the UV-Vis stopped-flow measurement of the reaction of **4** (1 mM) with 56.25 mM DHA at 25 °C in a mixture of MeCN and DCM.

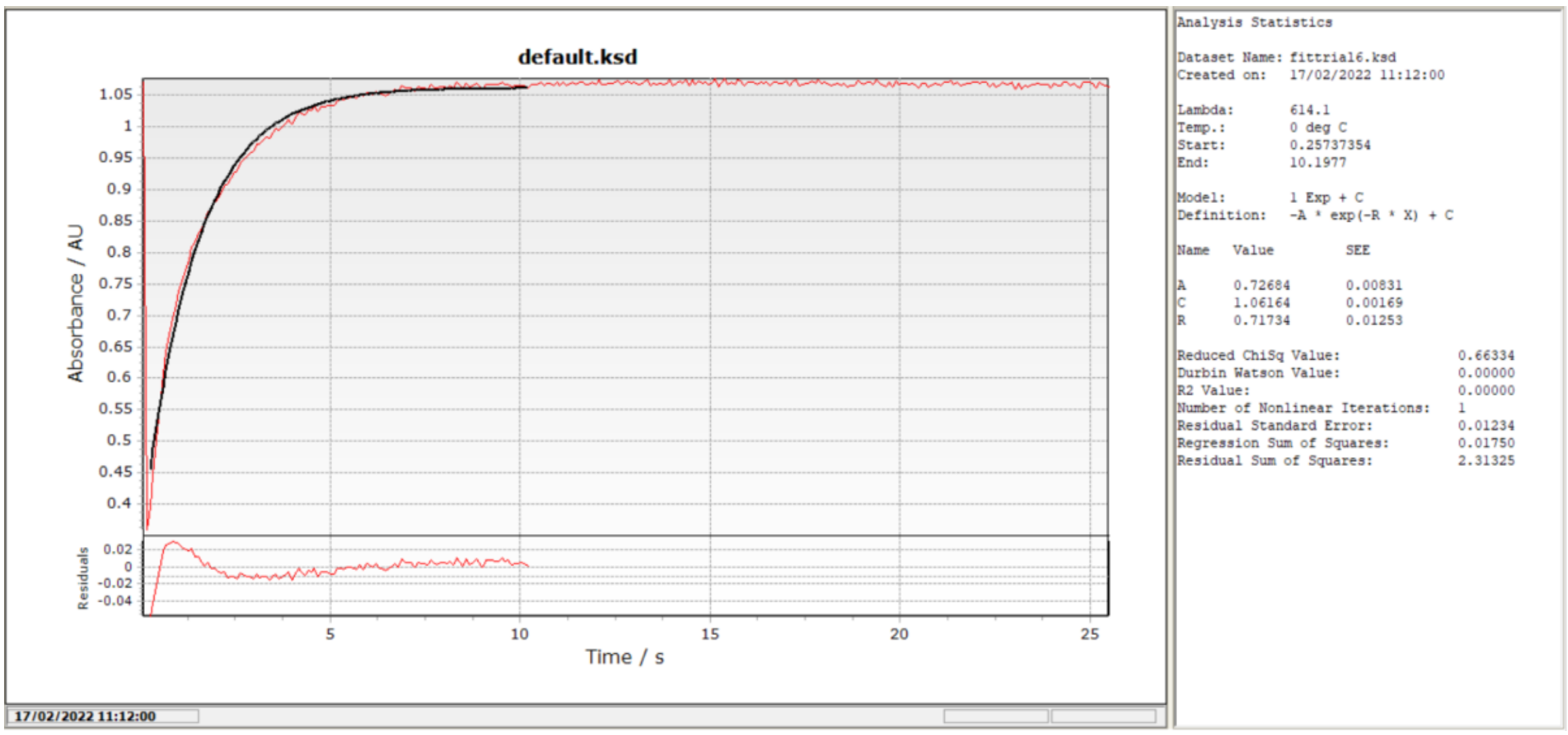

Figure S68. Exemplary trace (614 nm) and fit for the UV-Vis stopped-flow measurement of the reaction of **5a** (1 mM) with 56.25 mM DHA at 25 °C in a mixture of MeCN and DCM.

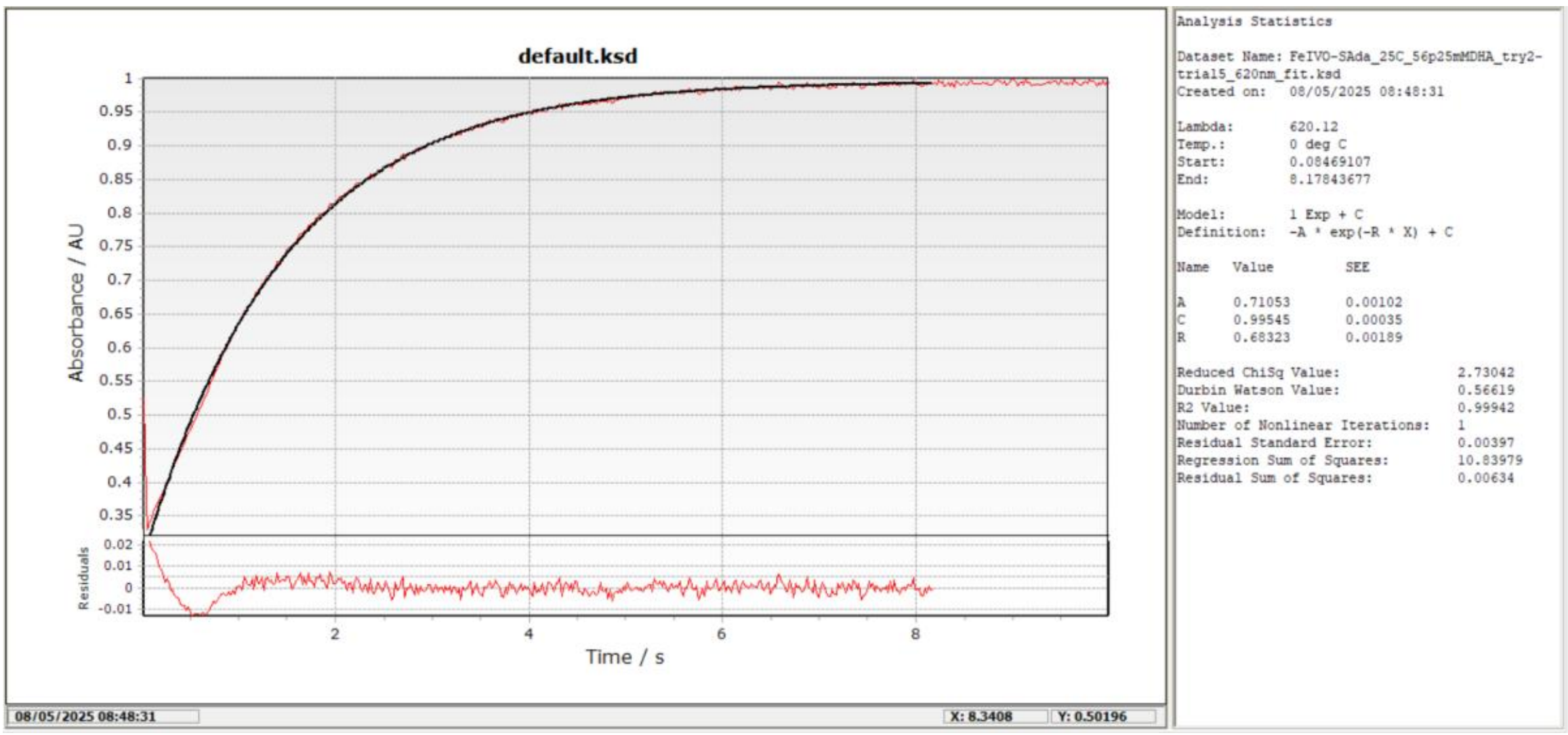

Figure S69. Exemplary trace (620 nm) and fit for the UV-Vis stopped-flow measurement of the reaction of **5b** (1 mM) with 56.25 mM DHA at 25 °C in a mixture of MeCN and DCM.

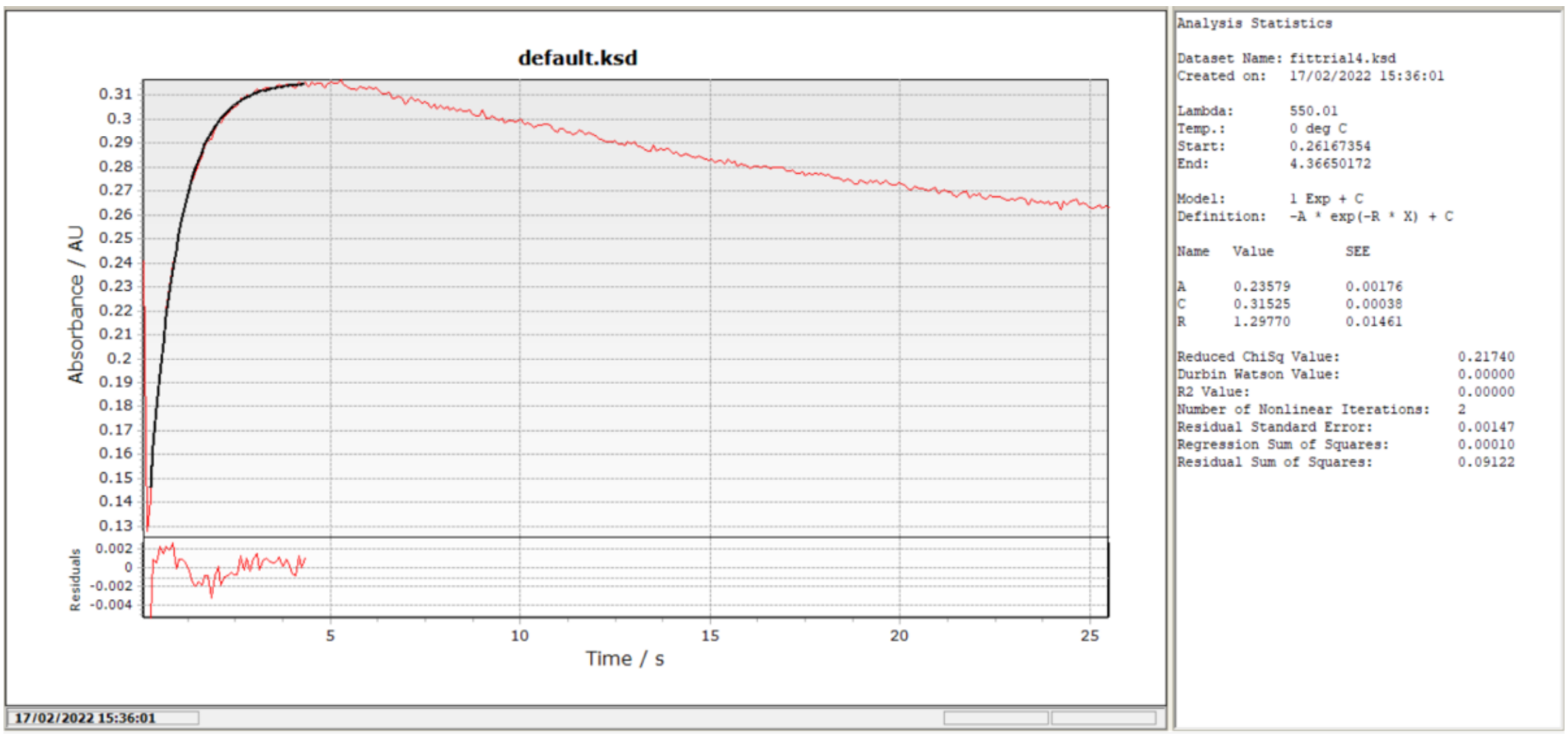

Figure S70. Exemplary trace (550 nm) and fit for the UV-Vis stopped-flow measurement of the reaction of **3** (1 mM) with 37.5 mM DHA at 25 °C in a mixture of MeCN and DCM.

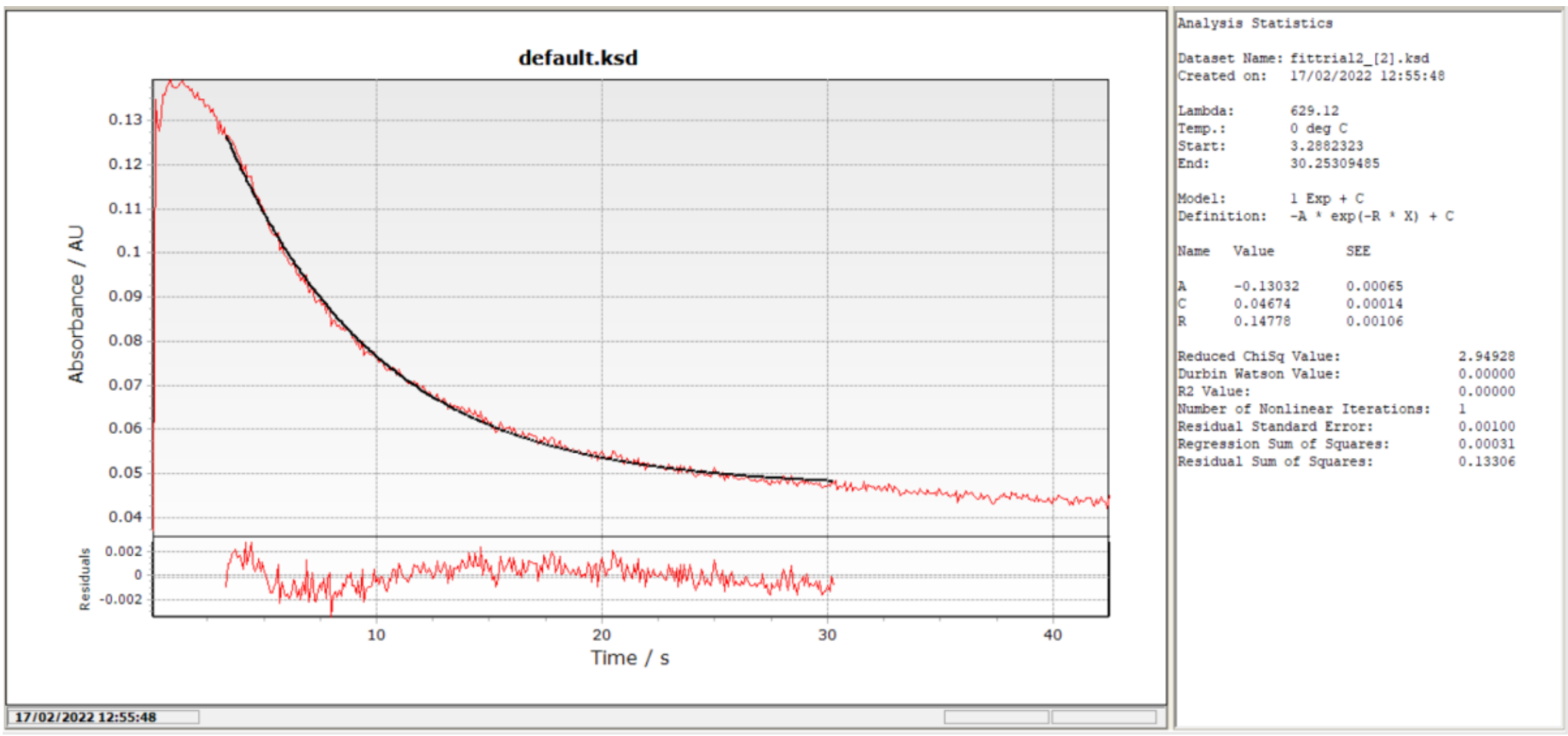

Figure S71. Exemplary trace (629 nm) and fit for the UV-Vis stopped-flow measurement of the reaction of **4** (1 mM) with 37.5 mM DHA at 25 °C in a mixture of MeCN and DCM.

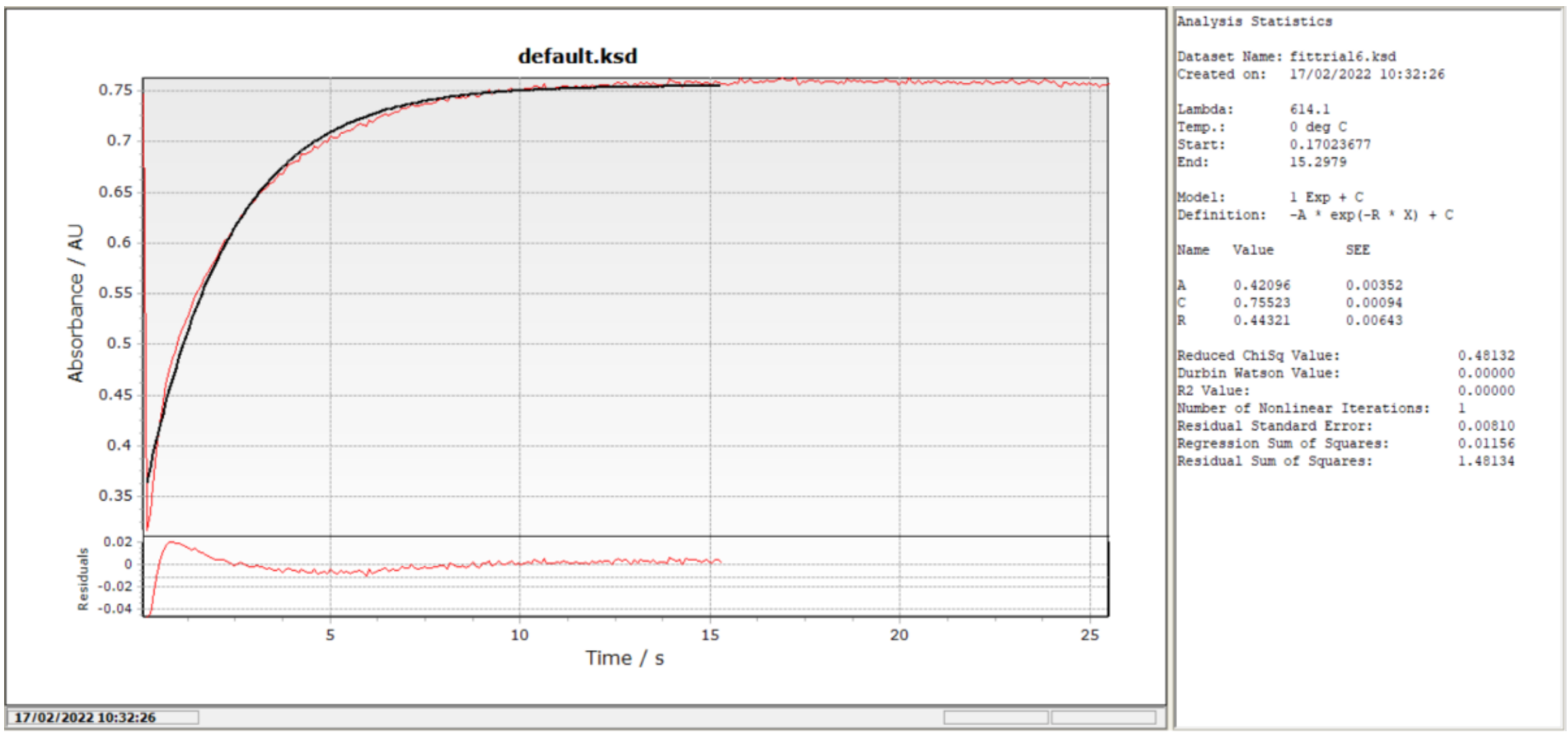

Figure S72. Exemplary trace (614 nm) and fit for the UV-Vis stopped-flow measurement of the reaction of **5a** (1 mM) with 37.5 mM DHA at 25 °C in a mixture of MeCN and DCM.

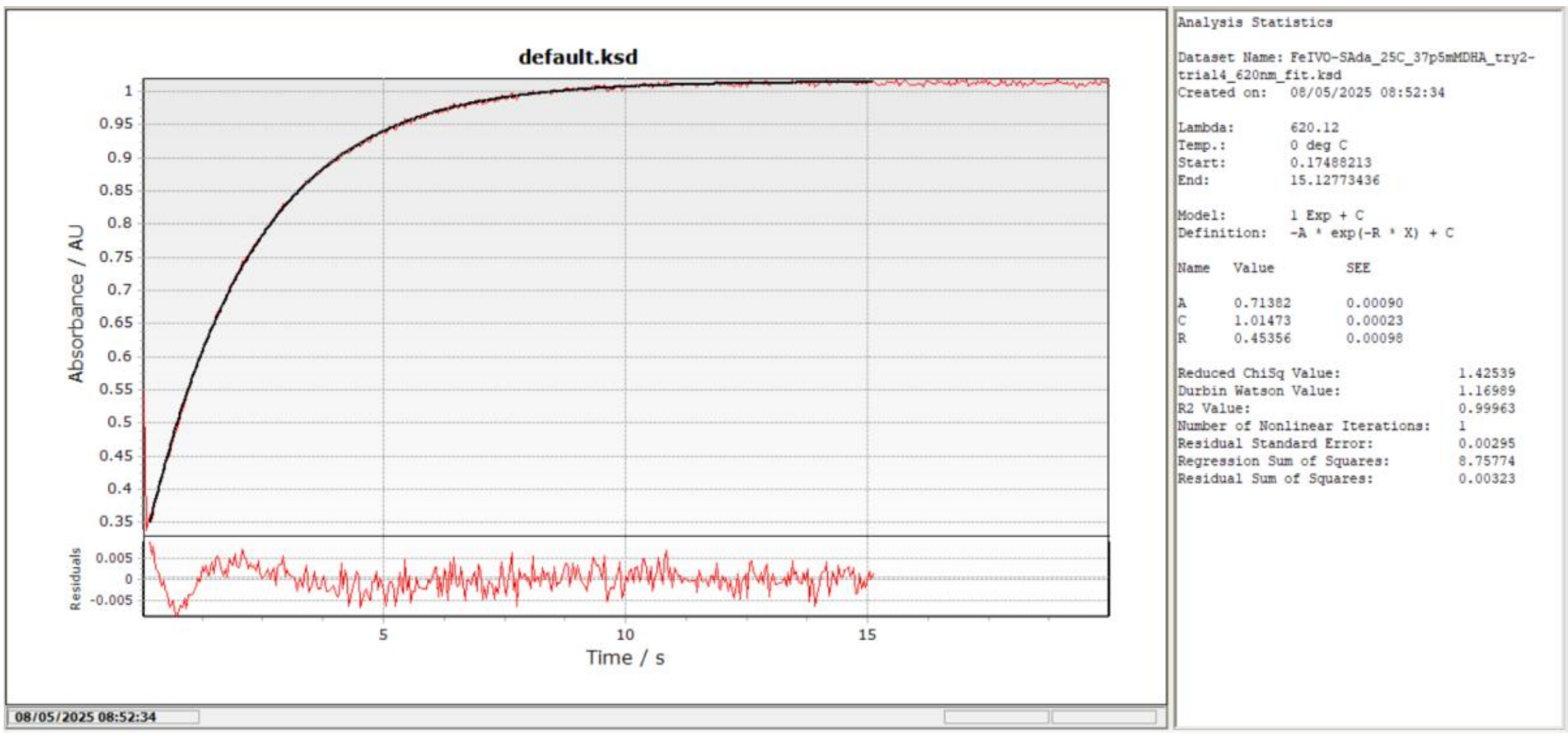

Figure S73. Exemplary trace (620 nm) and fit for the UV-Vis stopped-flow measurement of the reaction of **5b** (1 mM) with 37.5 mM DHA at 25 °C in a mixture of MeCN and DCM.

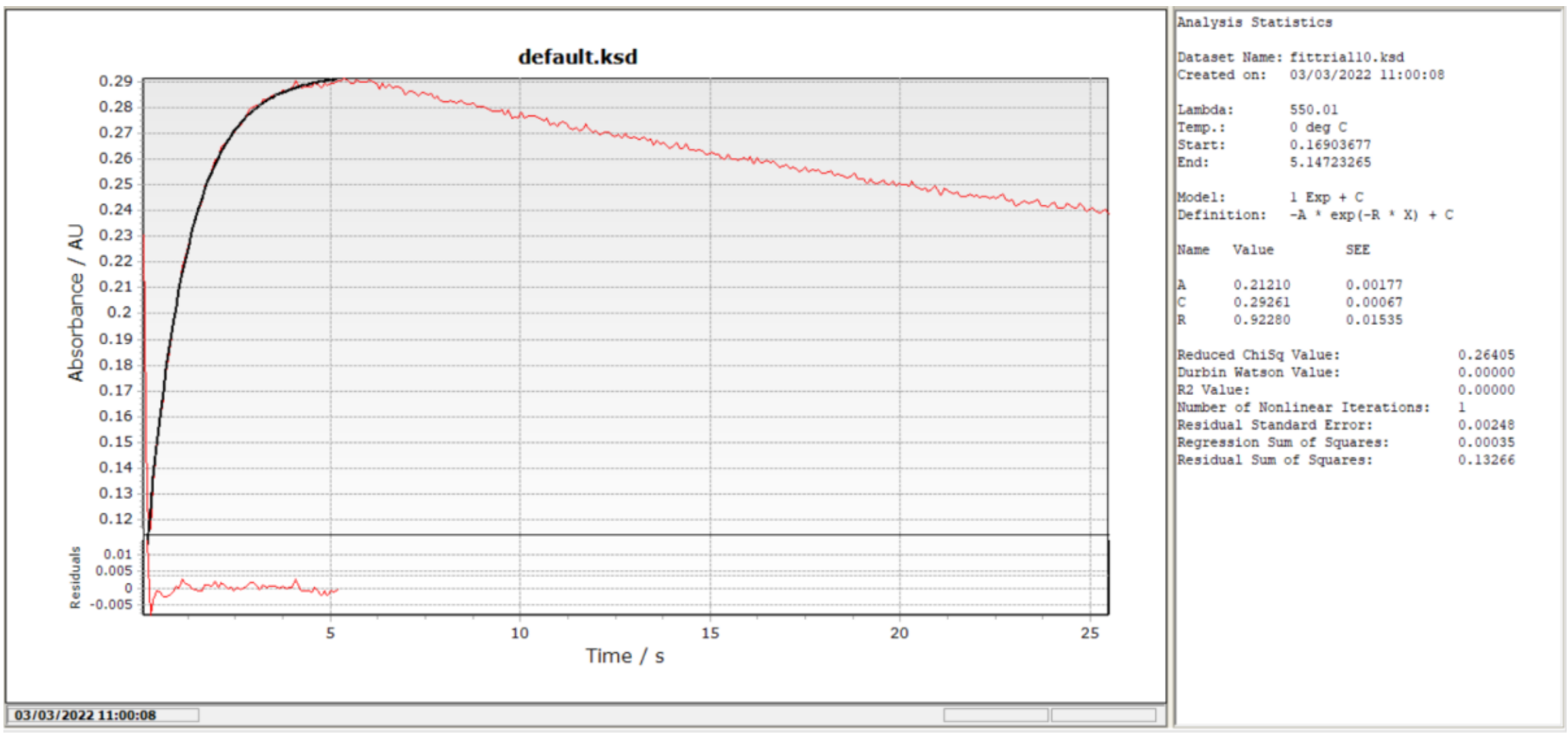

Figure S74. Exemplary trace (550 nm) and fit for the UV-Vis stopped-flow measurement of the reaction of **3** (1 mM) with 28.125 mM DHA at 25 °C in a mixture of MeCN and DCM.

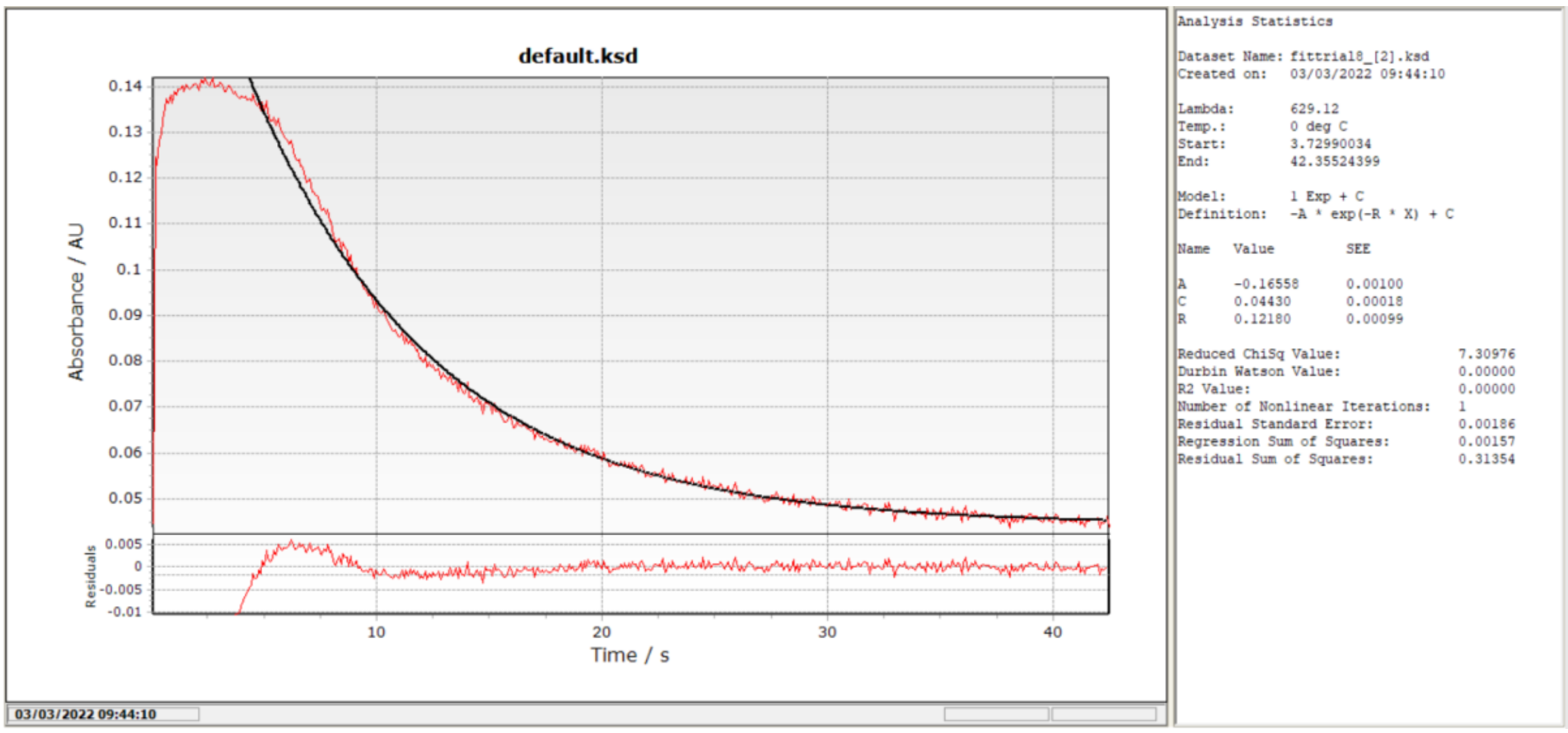

Figure S75. Exemplary trace (629 nm) and fit for the UV-Vis stopped-flow measurement of the reaction of **4** (1 mM) with 28.125 mM DHA at 25 °C in a mixture of MeCN and DCM.

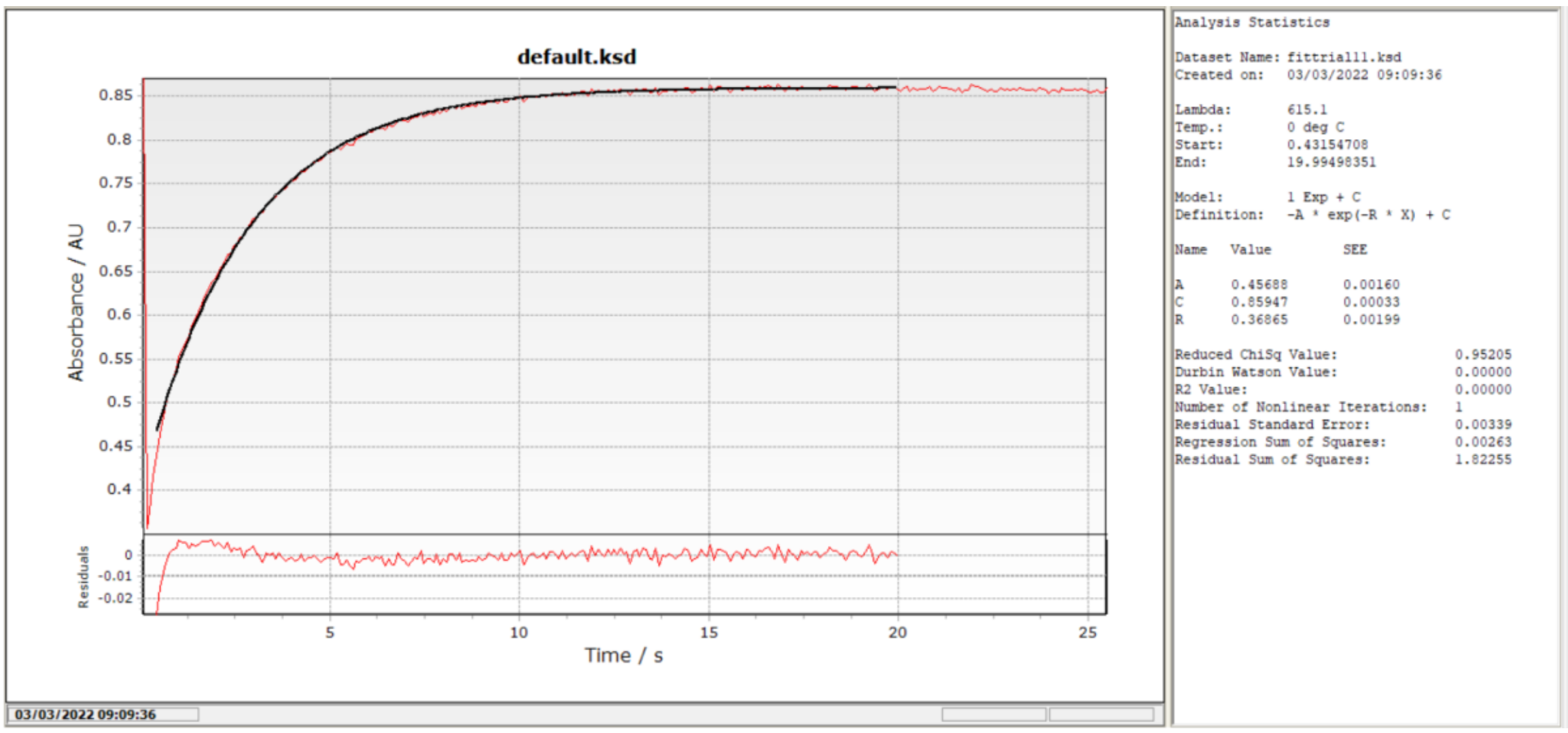

Figure S76. Exemplary trace (615 nm) and fit for the UV-Vis stopped-flow measurement of the reaction of **5a** (1 mM) with 28.125 mM DHA at 25 °C in a mixture of MeCN and DCM.

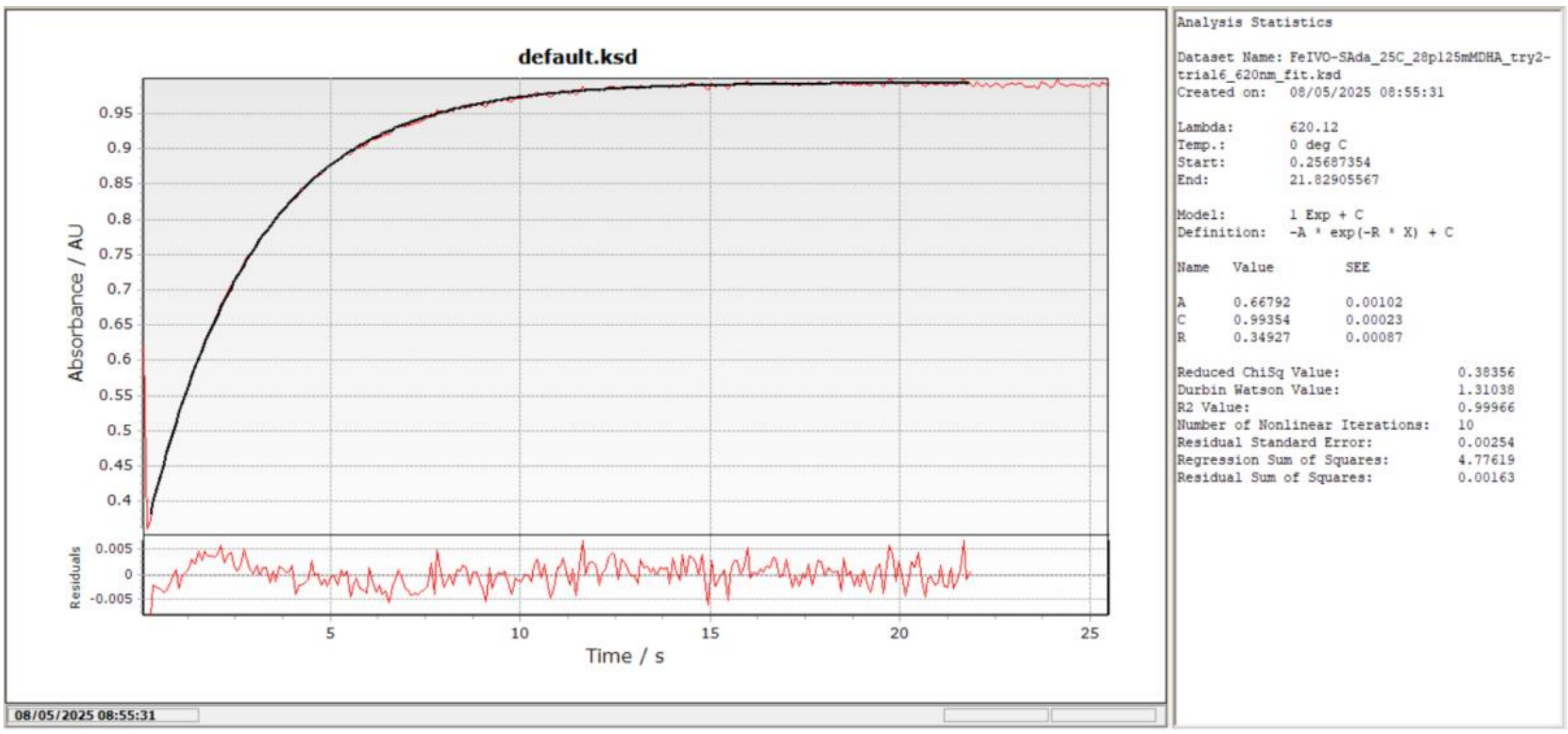

Figure S77. Exemplary trace (620 nm) and fit for the UV-Vis stopped-flow measurement of the reaction of **5b** (1 mM) with 28.125 mM DHA at 25 °C in a mixture of MeCN and DCM.

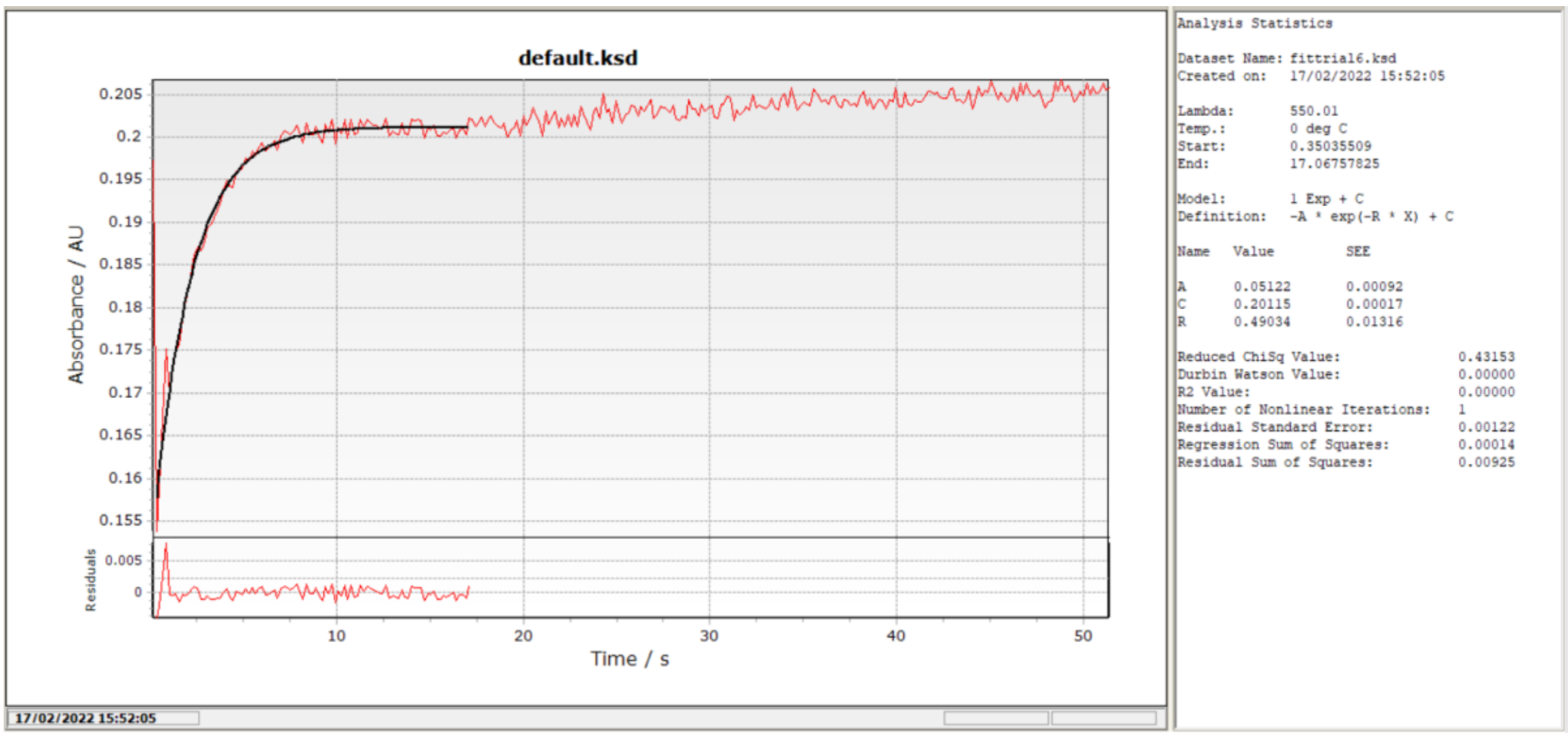

Figure S78. Exemplary trace (550 nm) and fit for the UV-Vis stopped-flow measurement of the reaction of **3** (1 mM) with 18.75 mM DHA at 25 °C in a mixture of MeCN and DCM.

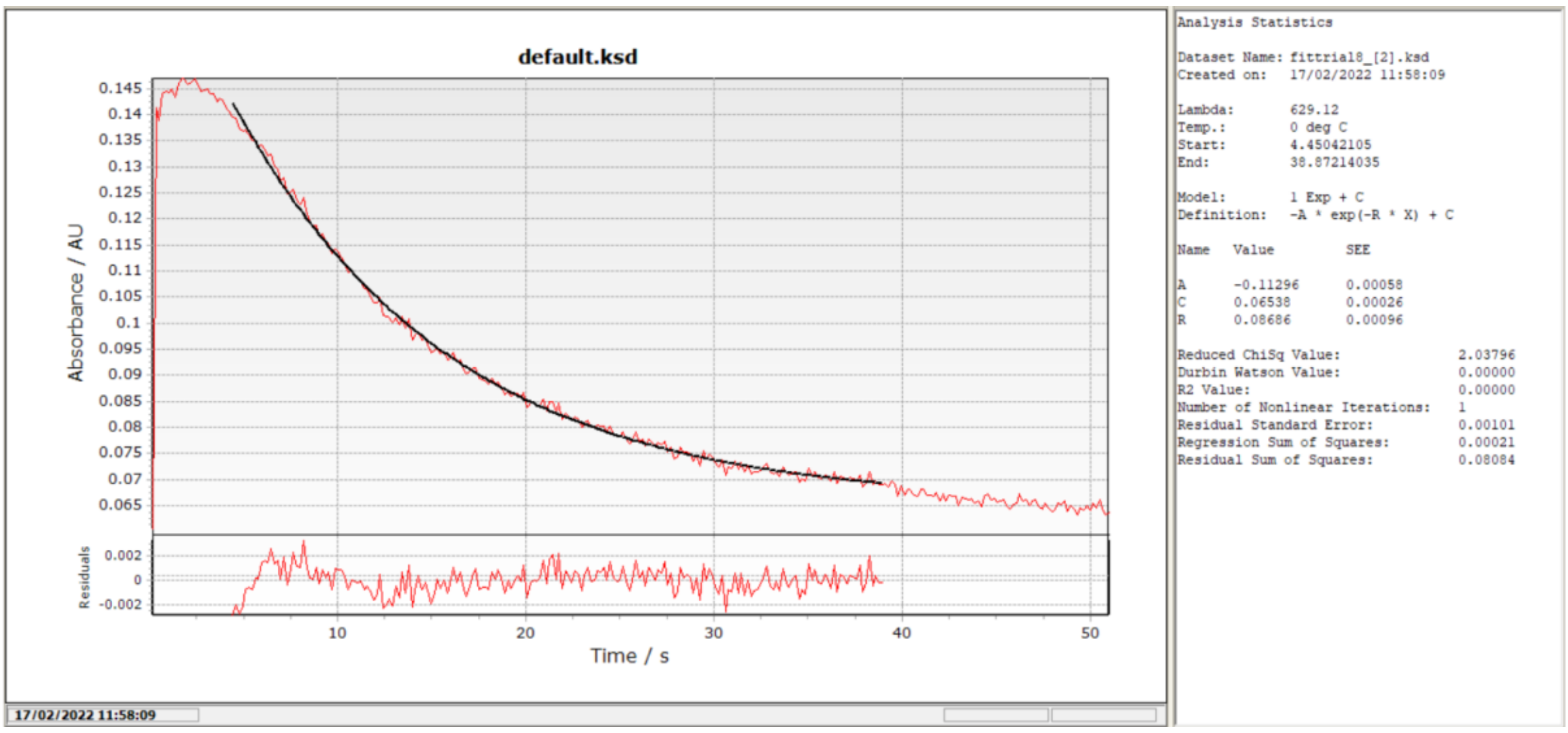

Figure S79. Exemplary trace (629 nm) and fit for the UV-Vis stopped-flow measurement of the reaction of **4** (1 mM) with 18.75 mM DHA at 25 °C in a mixture of MeCN and DCM.

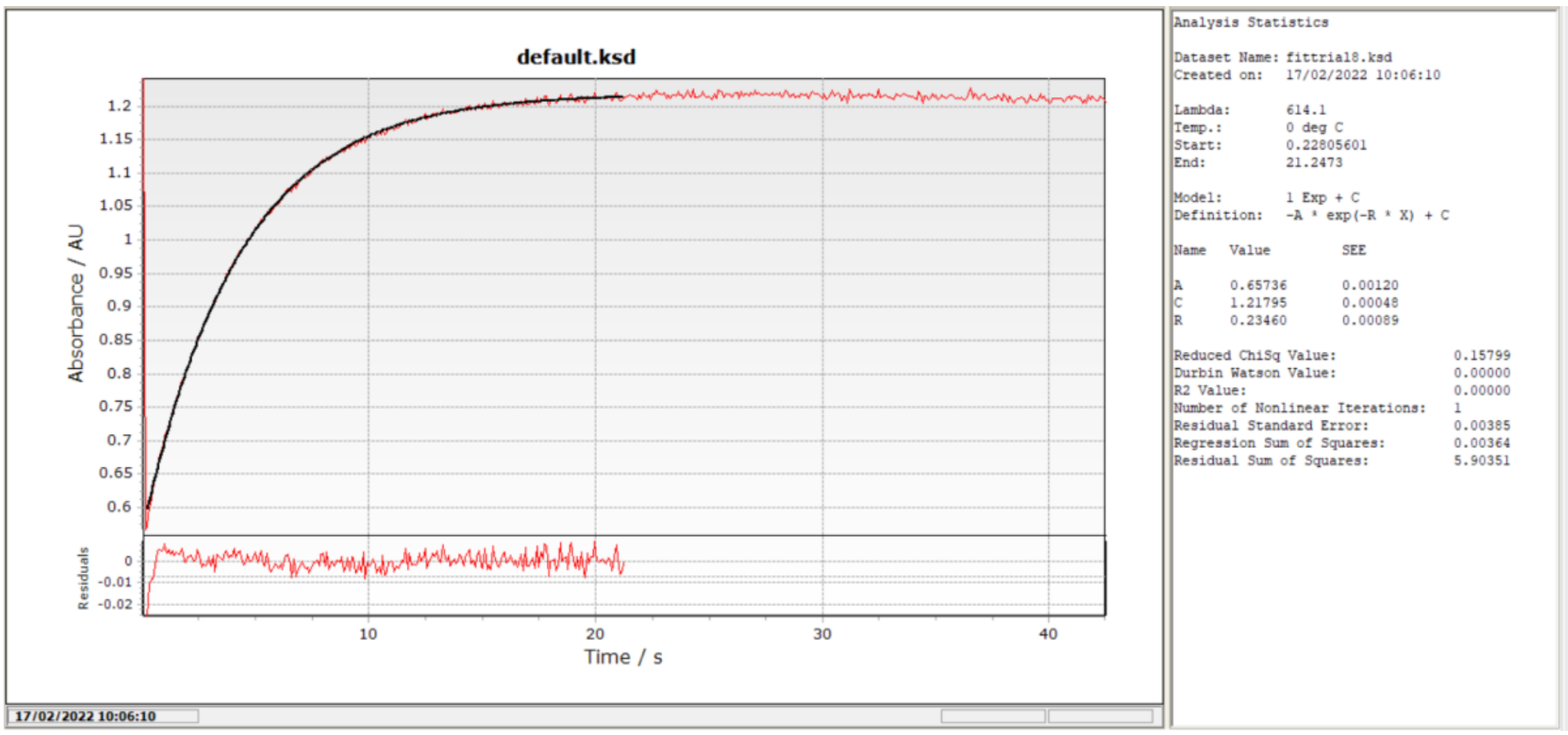

Figure S80. Exemplary trace (614 nm) and fit for the UV-Vis stopped-flow measurement of the reaction of **5a** (1 mM) with 18.75 mM DHA at 25 °C in a mixture of MeCN and DCM.

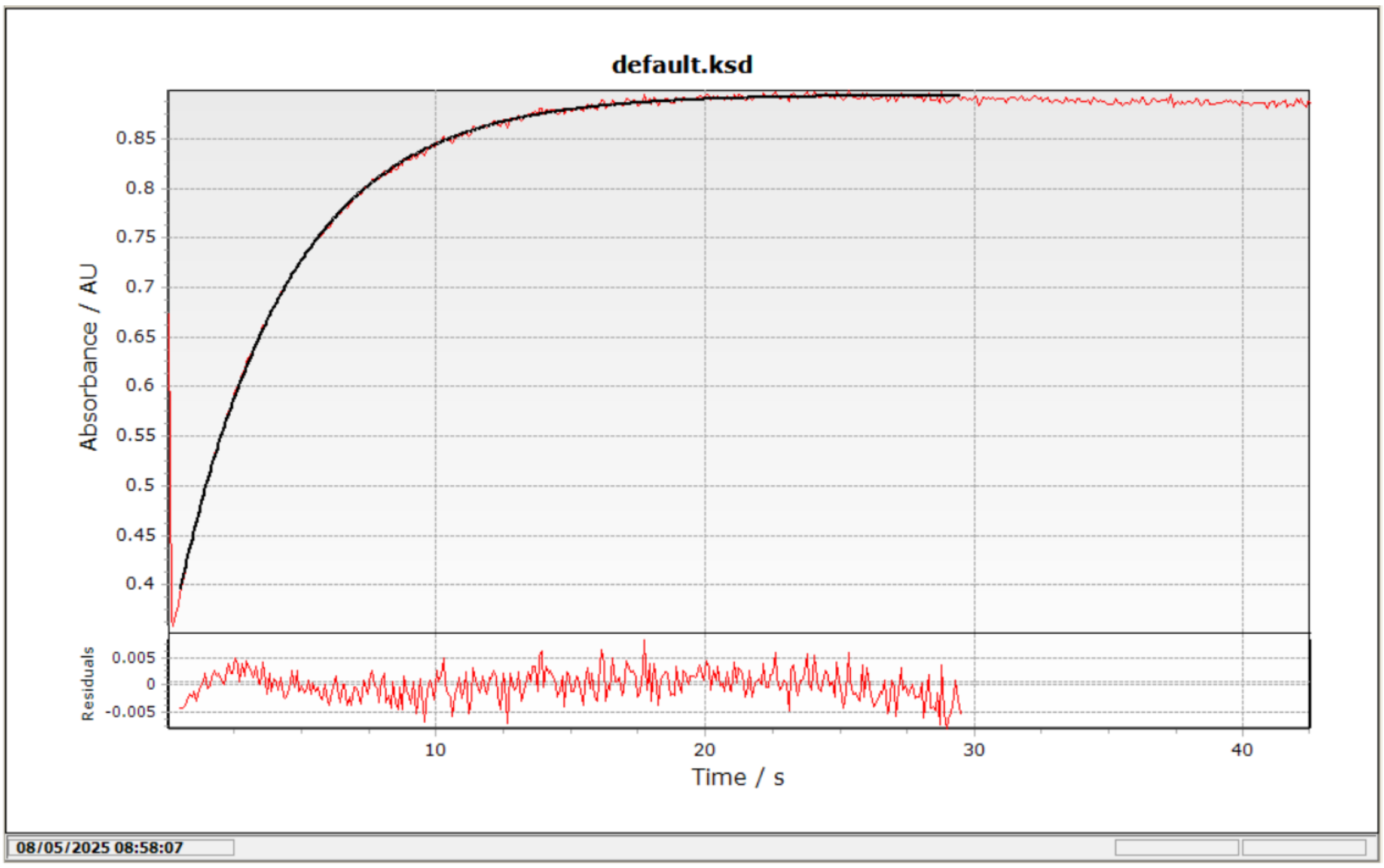

Figure S81. Exemplary trace (620 nm) and fit for the UV-Vis stopped-flow measurement of the reaction of **5b** (1 mM) with 18.75 mM DHA at 25 °C in a mixture of MeCN and DCM.

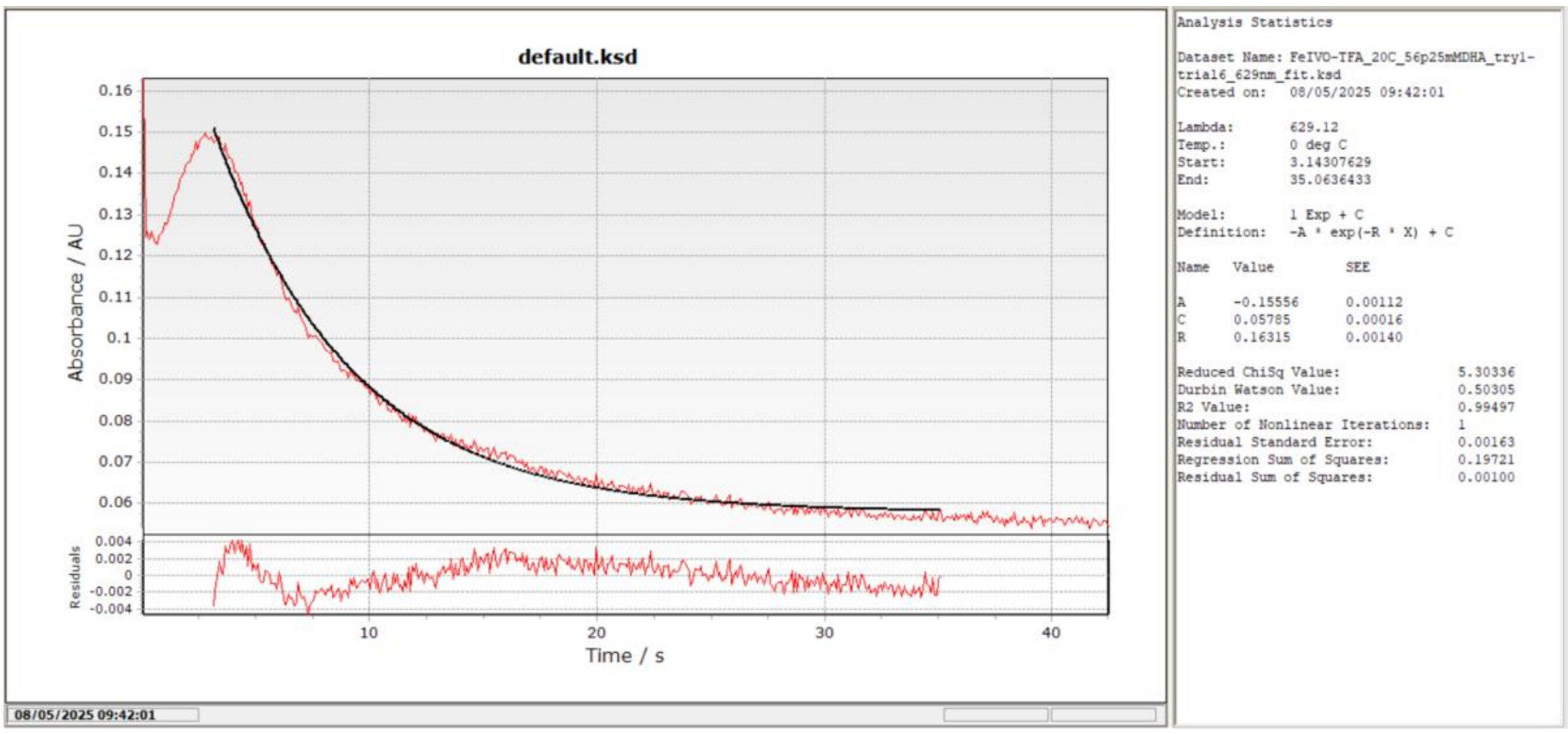

Figure S82. Exemplary trace (629 nm) and fit for the UV-Vis stopped-flow measurement of the reaction of **4** (1 mM) with 56.25 mM DHA at 20 °C in a mixture of MeCN and DCM.

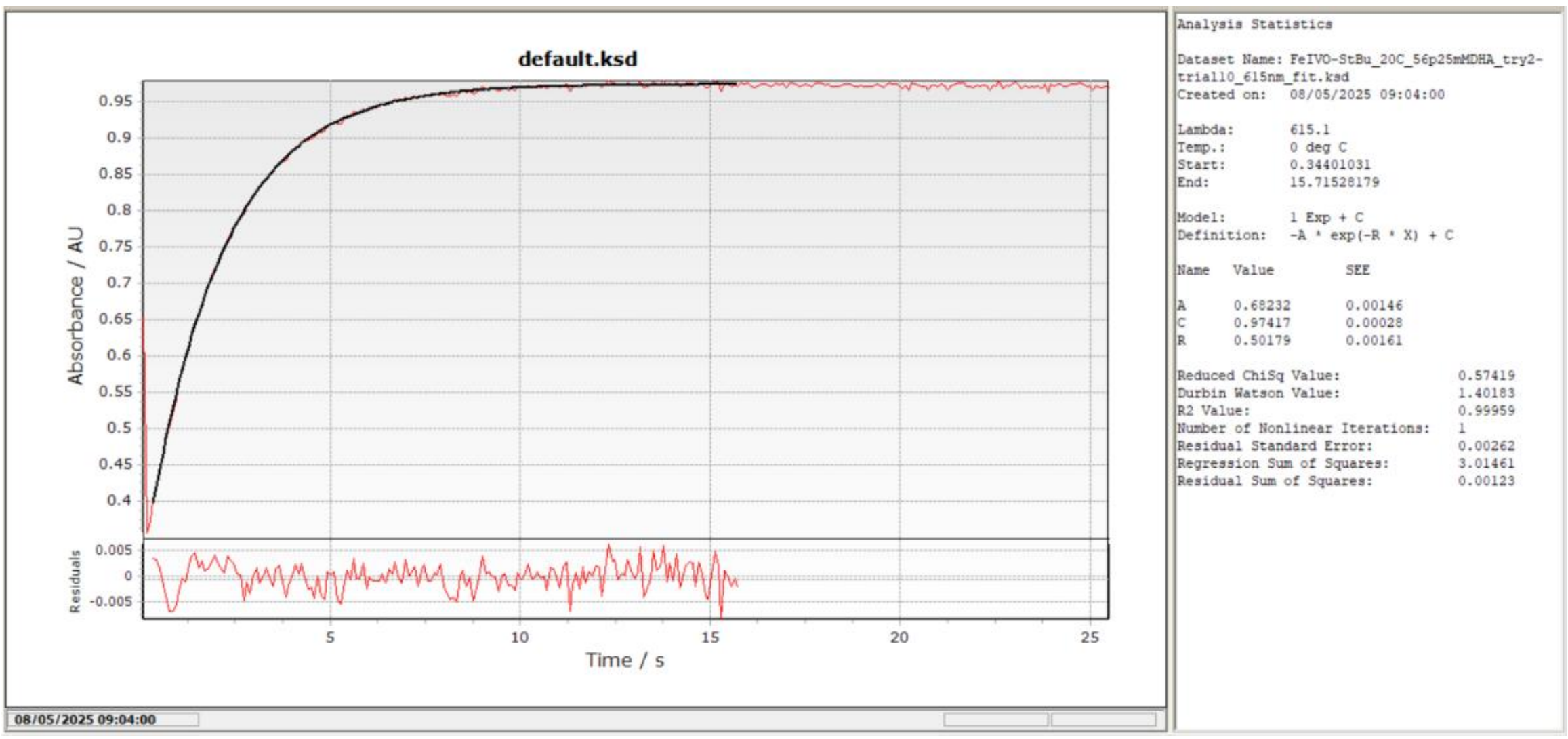

Figure S83. Exemplary trace (615 nm) and fit for the UV-Vis stopped-flow measurement of the reaction of **5a** (1 mM) with 56.25 mM DHA at 20 °C in a mixture of MeCN and DCM.

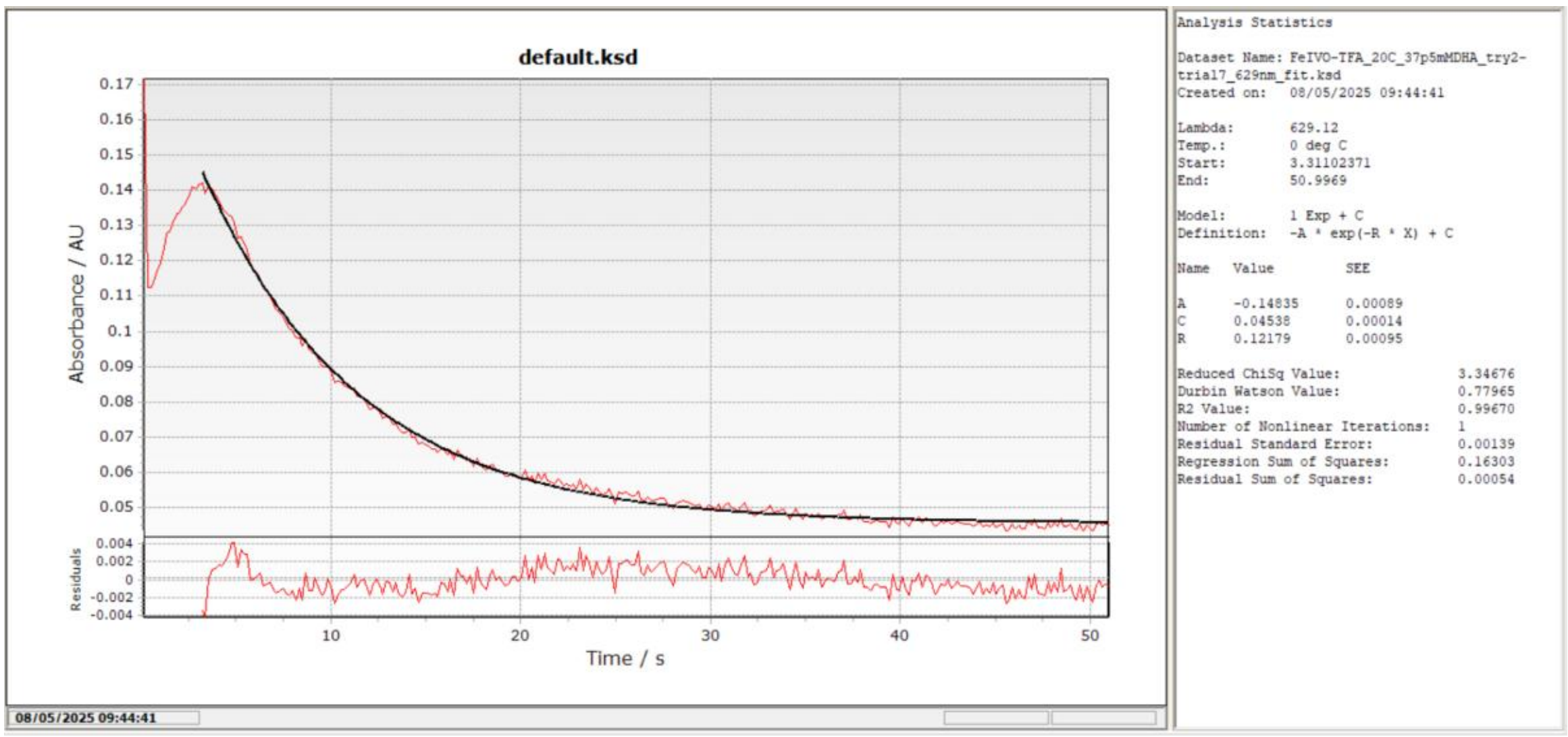

Figure S84. Exemplary trace (629 nm) and fit for the UV-Vis stopped-flow measurement of the reaction of **4** (1 mM) with 37.5 mM DHA at 20 °C in a mixture of MeCN and DCM.

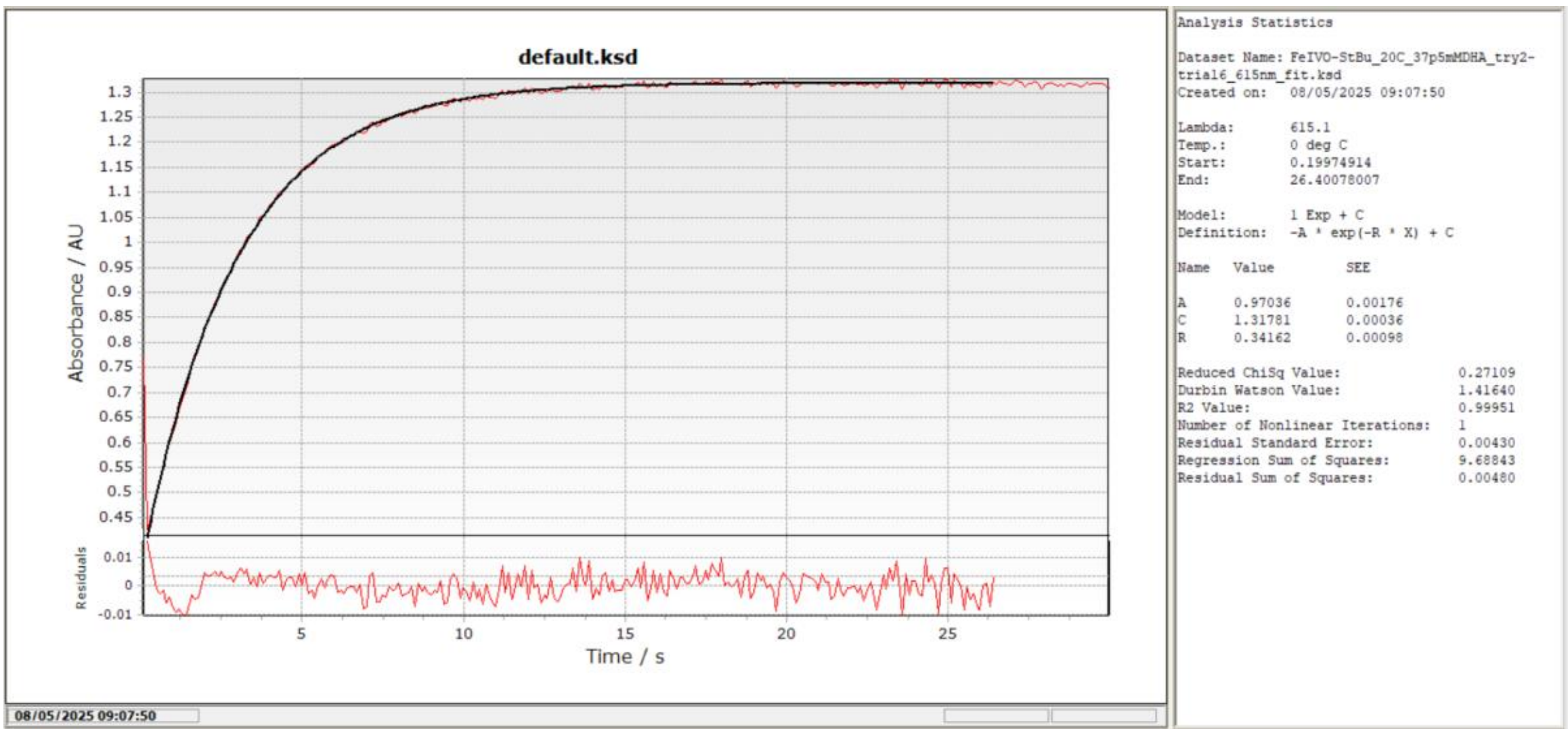

Figure S85. Exemplary trace (615 nm) and fit for the UV-Vis stopped-flow measurement of the reaction of **5a** (1 mM) with 37.5 mM DHA at 20 °C in a mixture of MeCN and DCM.

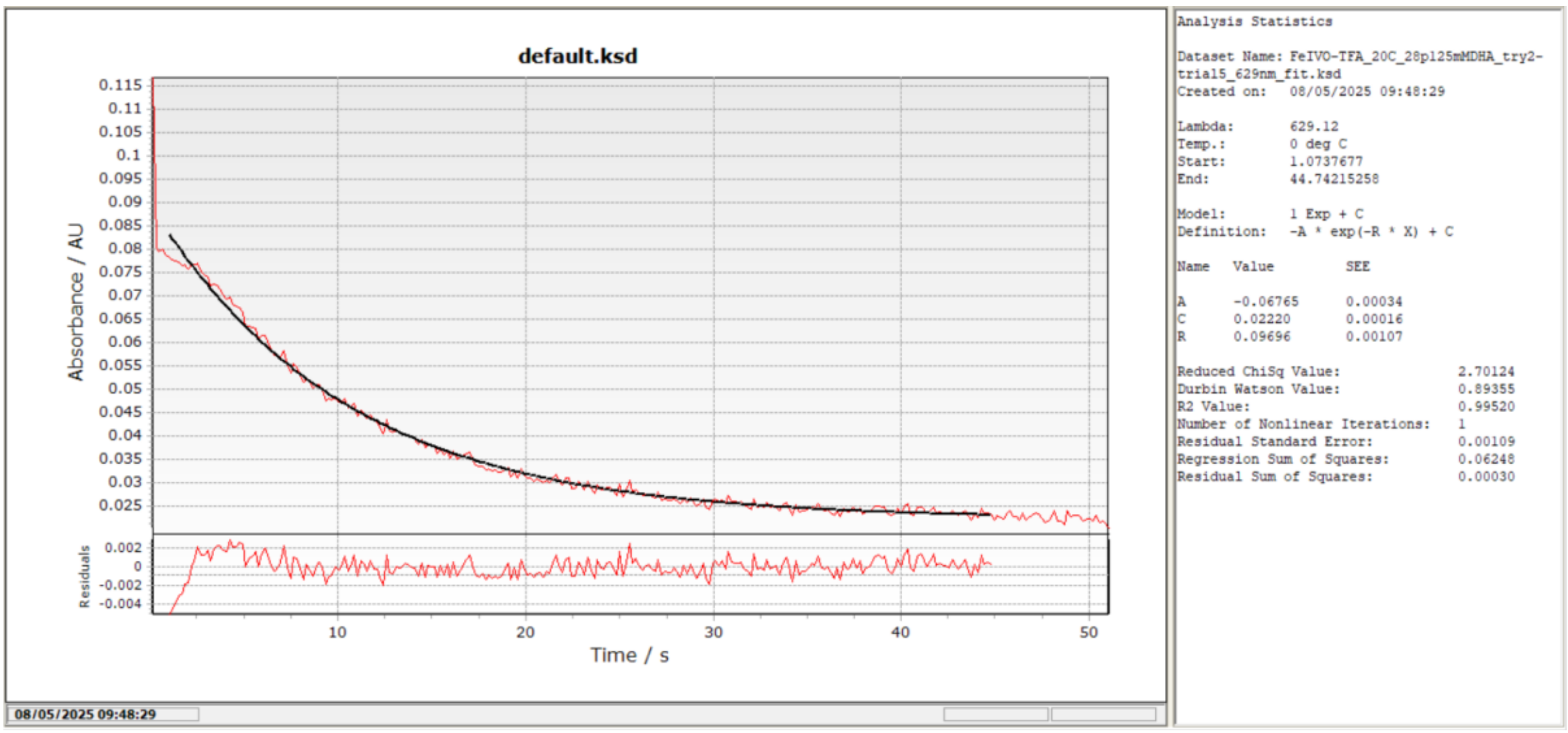

Figure S86. Exemplary trace (629 nm) and fit for the UV-Vis stopped-flow measurement of the reaction of **4** (1 mM) with 28.125 mM DHA at 20 °C in a mixture of MeCN and DCM.

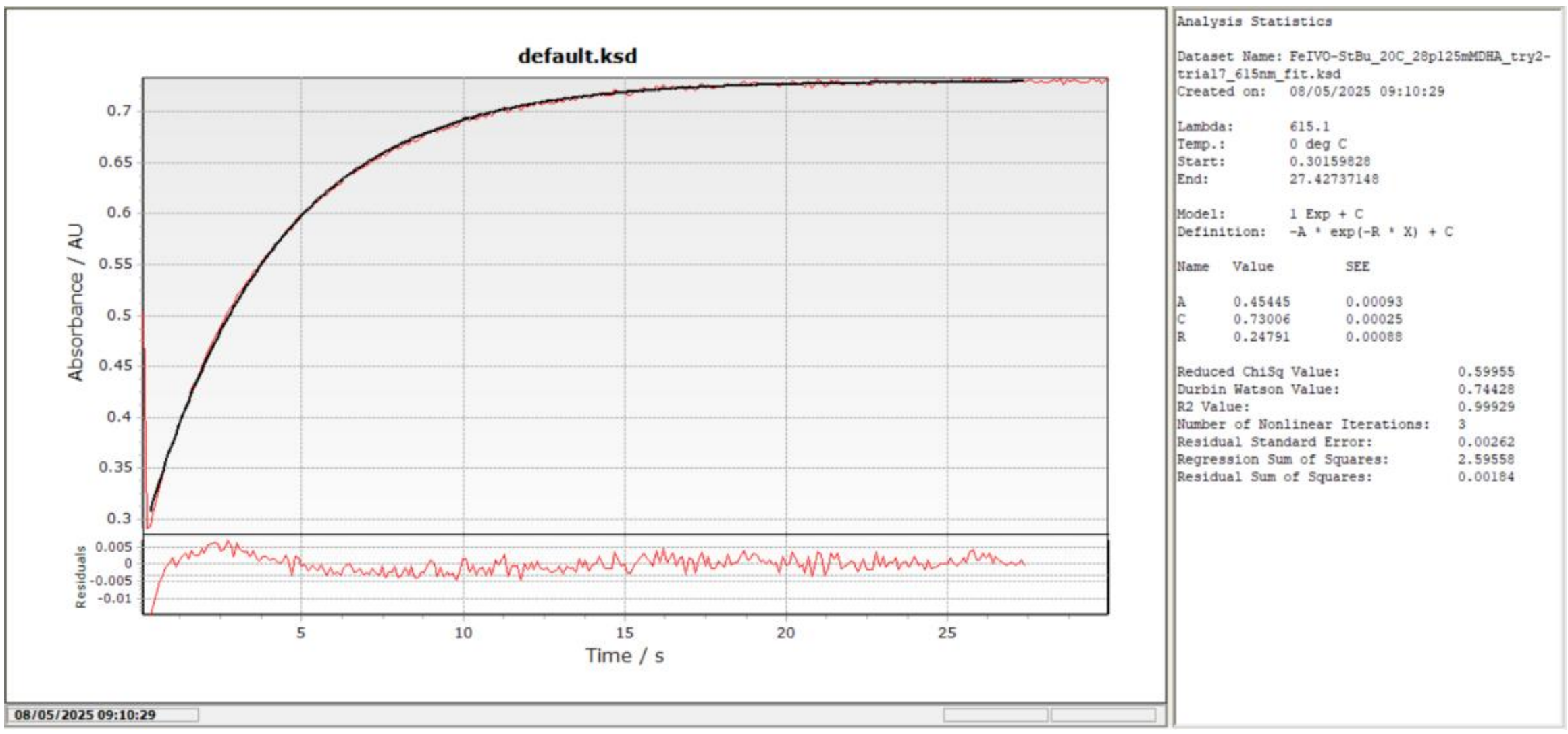

Figure S87. Exemplary trace (615 nm) and fit for the UV-Vis stopped-flow measurement of the reaction of **5a** (1 mM) with 28.125 mM DHA at 20 °C in a mixture of MeCN and DCM.

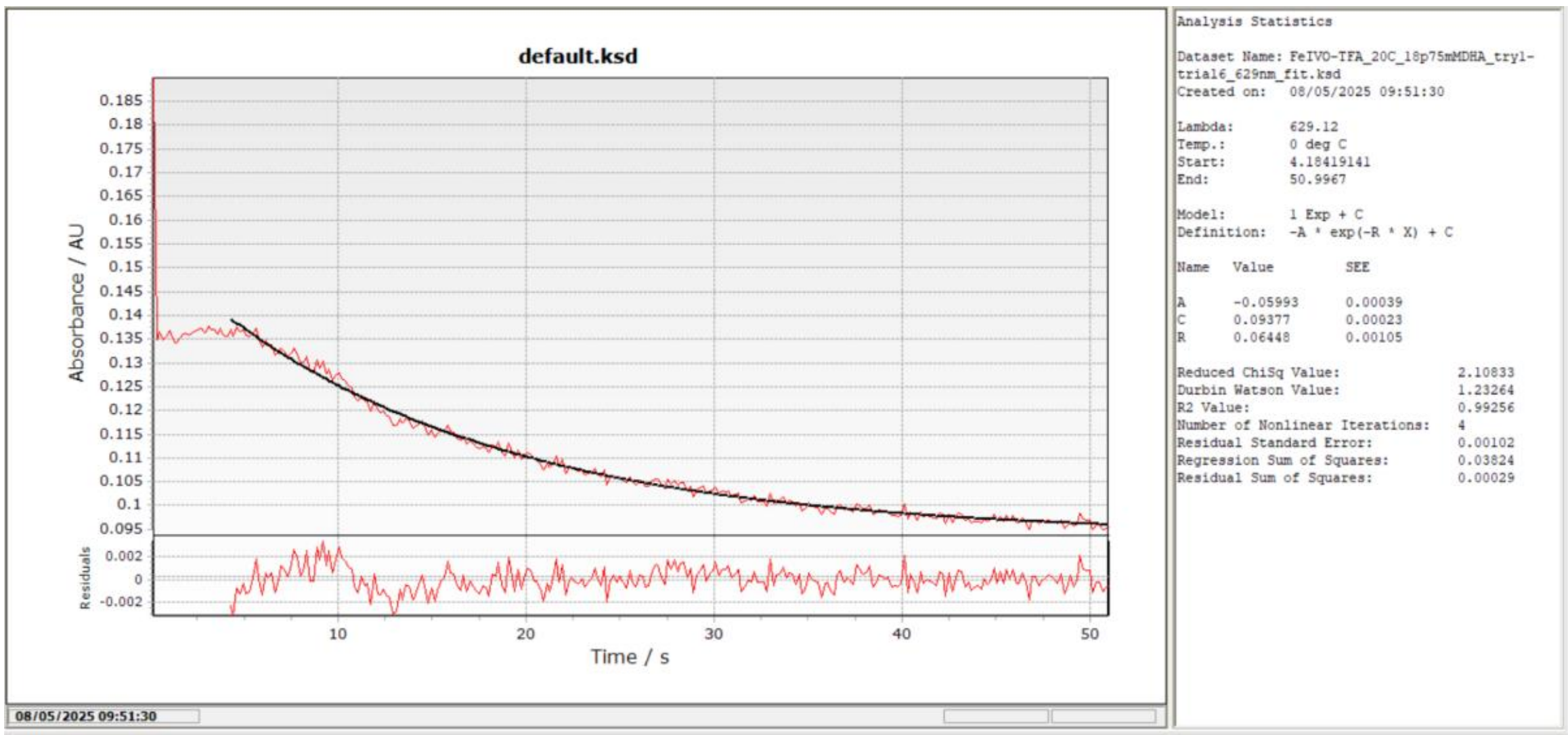

Figure S88. Exemplary trace (629 nm) and fit for the UV-Vis stopped-flow measurement of the reaction of **4** (1 mM) with 18.75 mM DHA at 20 °C in a mixture of MeCN and DCM.

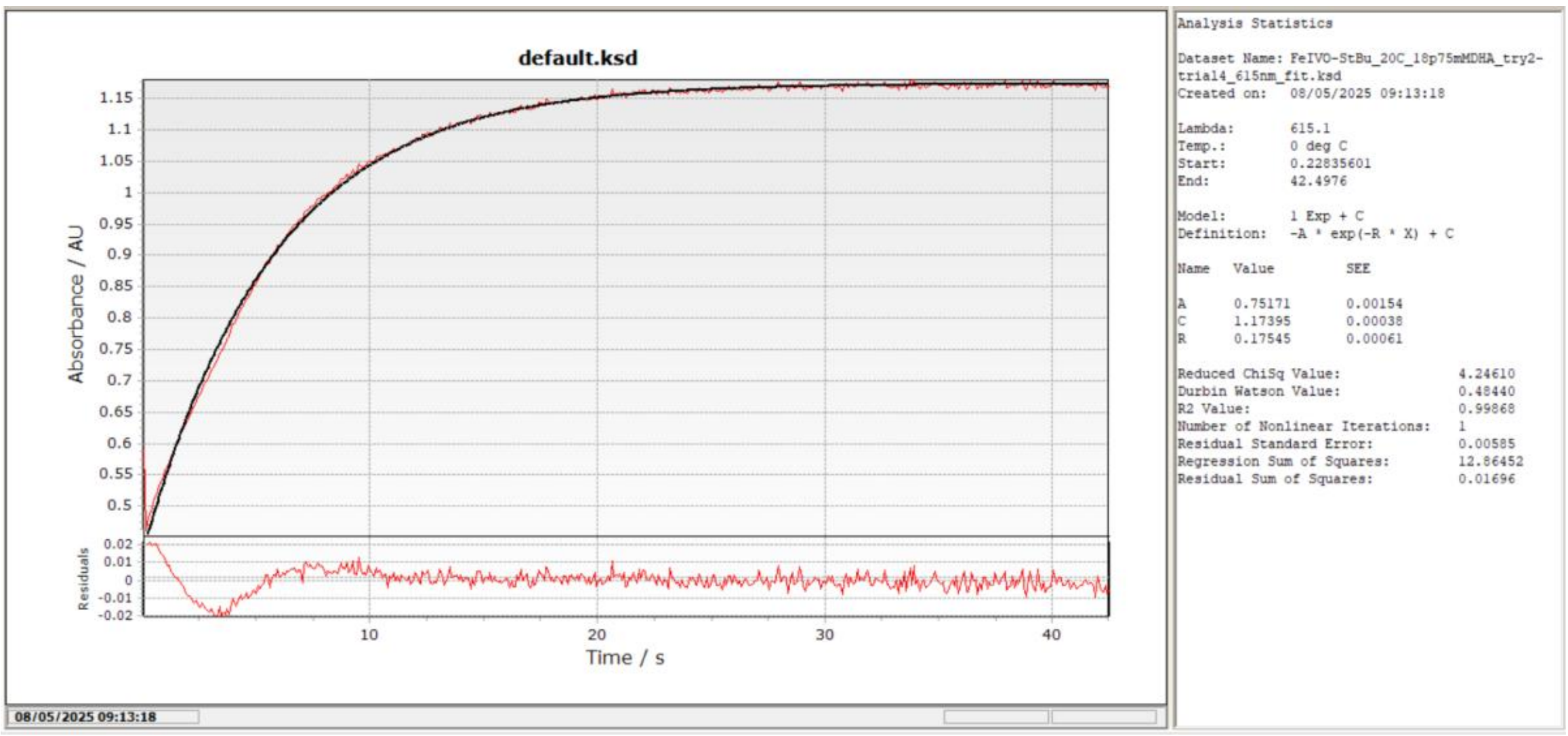

Figure S89. Exemplary trace (615 nm) and fit for the UV-Vis stopped-flow measurement of the reaction of **5a** (1 mM) with 18.75 mM DHA at 20 °C in a mixture of MeCN and DCM.

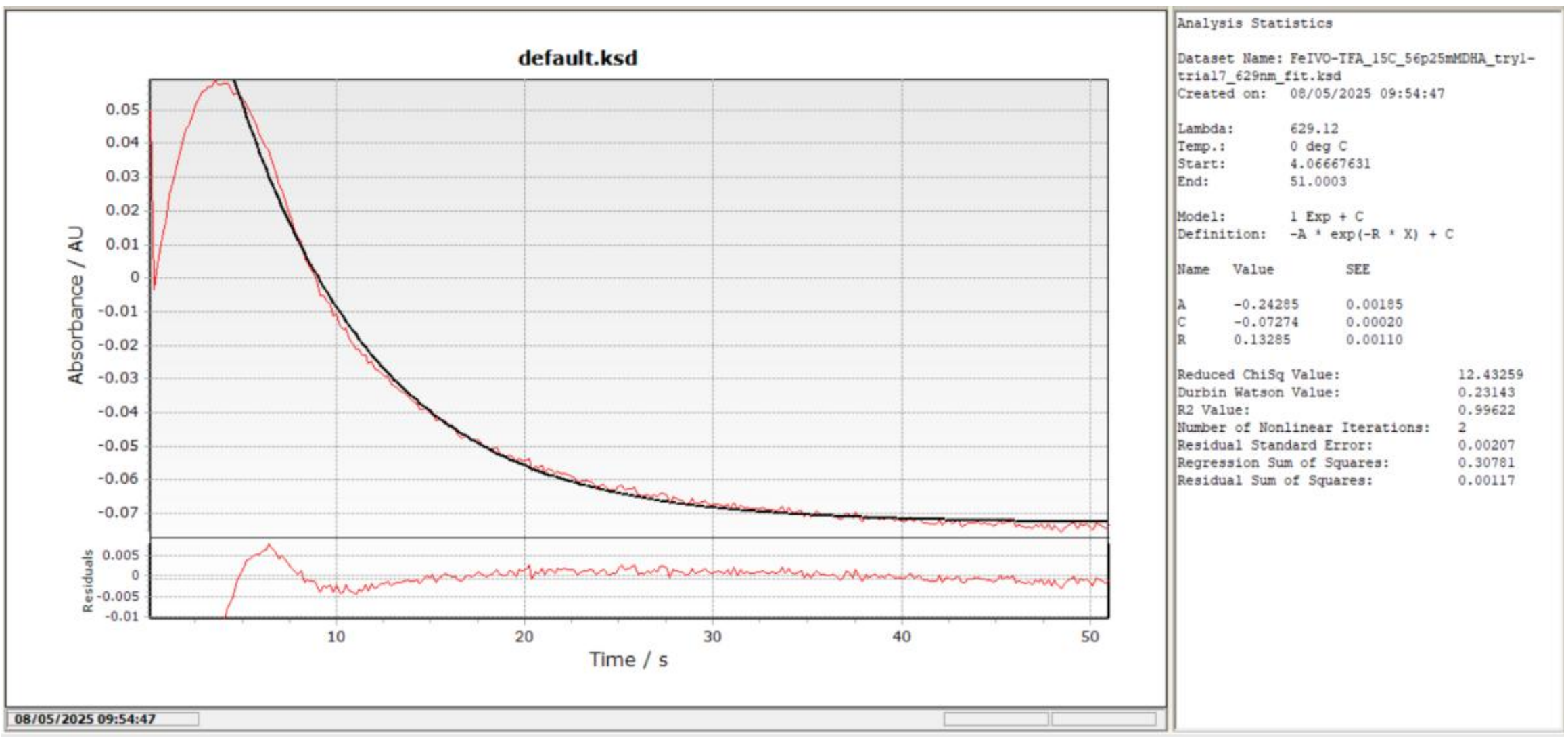

Figure S90. Exemplary trace (629 nm) and fit for the UV-Vis stopped-flow measurement of the reaction of **4** (1 mM) with 56.25 mM DHA at 15 °C in a mixture of MeCN and DCM.

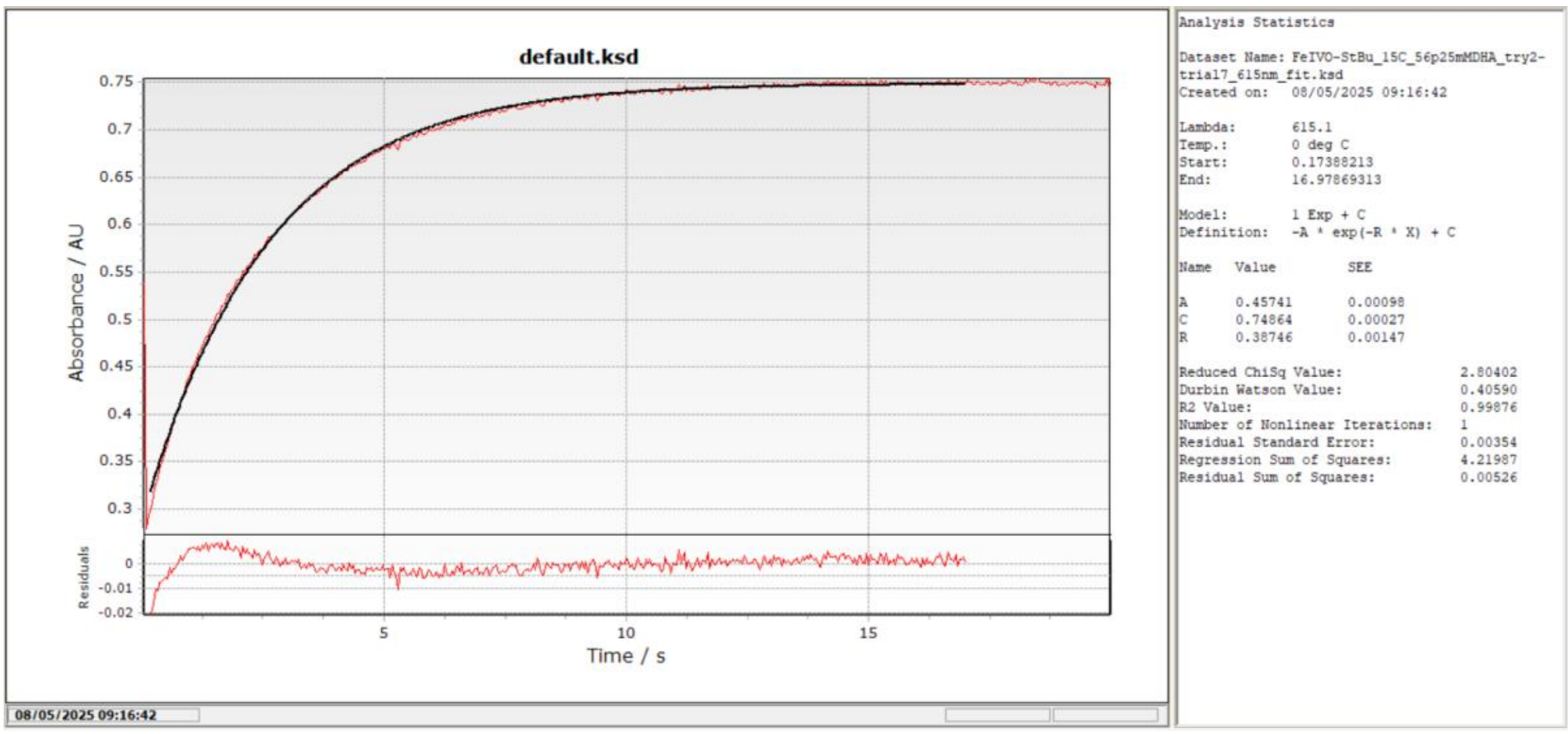

Figure S91. Exemplary trace (615 nm) and fit for the UV-Vis stopped-flow measurement of the reaction of **5a** (1 mM) with 56.25 mM DHA at 15 °C in a mixture of MeCN and DCM.

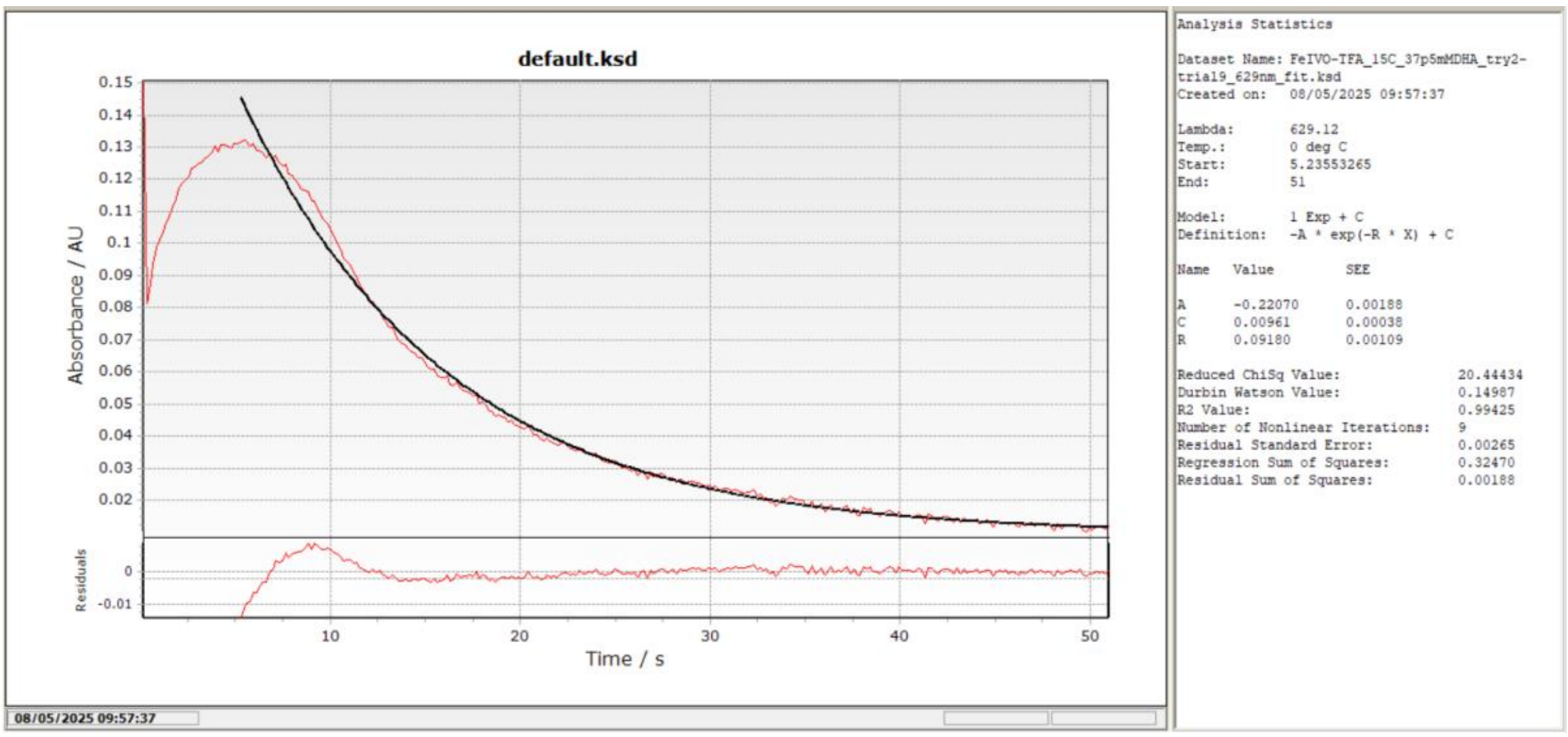

Figure S92. Exemplary trace (629 nm) and fit for the UV-Vis stopped-flow measurement of the reaction of **4** (1 mM) with 37.5 mM DHA at 15 °C in a mixture of MeCN and DCM.

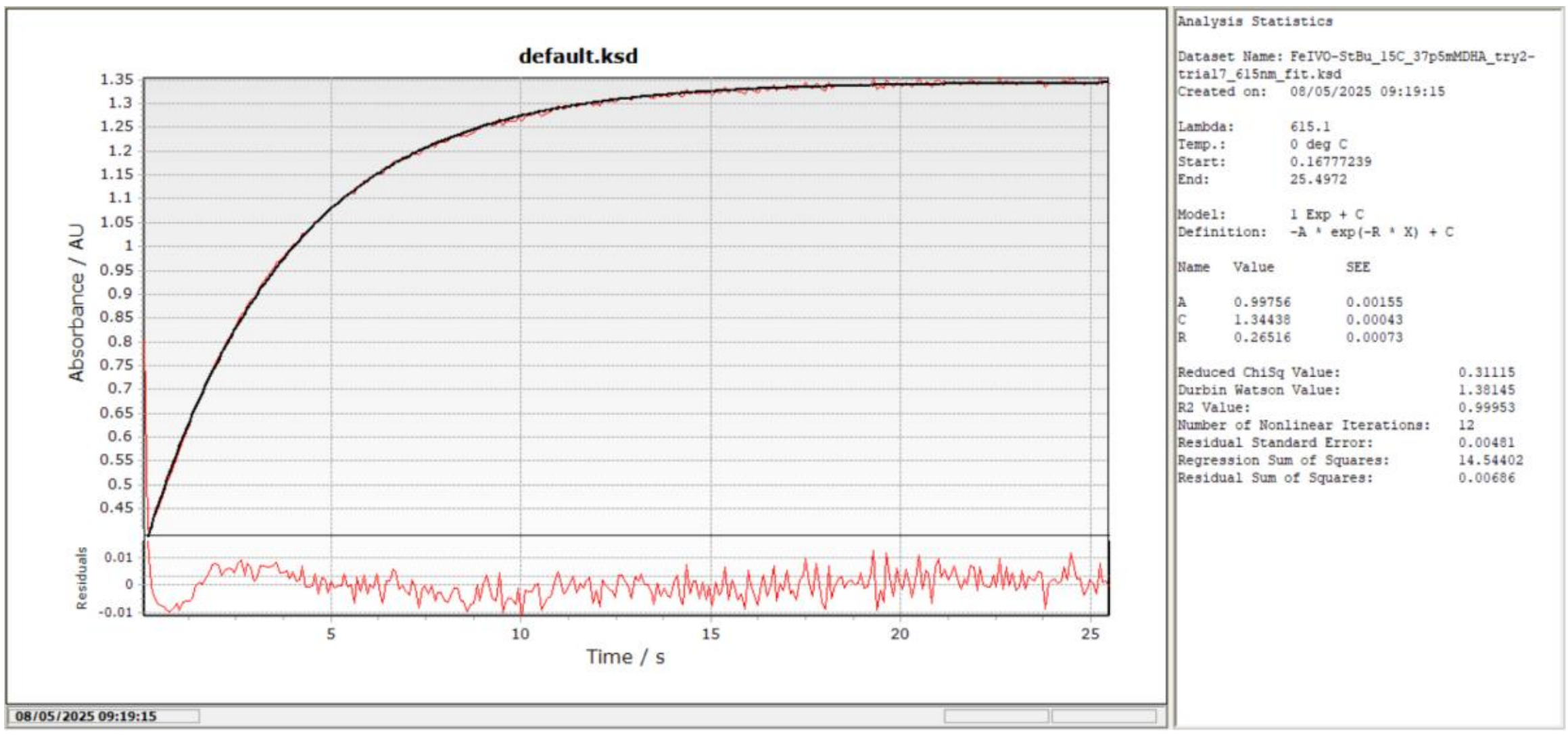

Figure S93. Exemplary trace (615 nm) and fit for the UV-Vis stopped-flow measurement of the reaction of **5a** (1 mM) with 37.5 mM DHA at 15 °C in a mixture of MeCN and DCM.

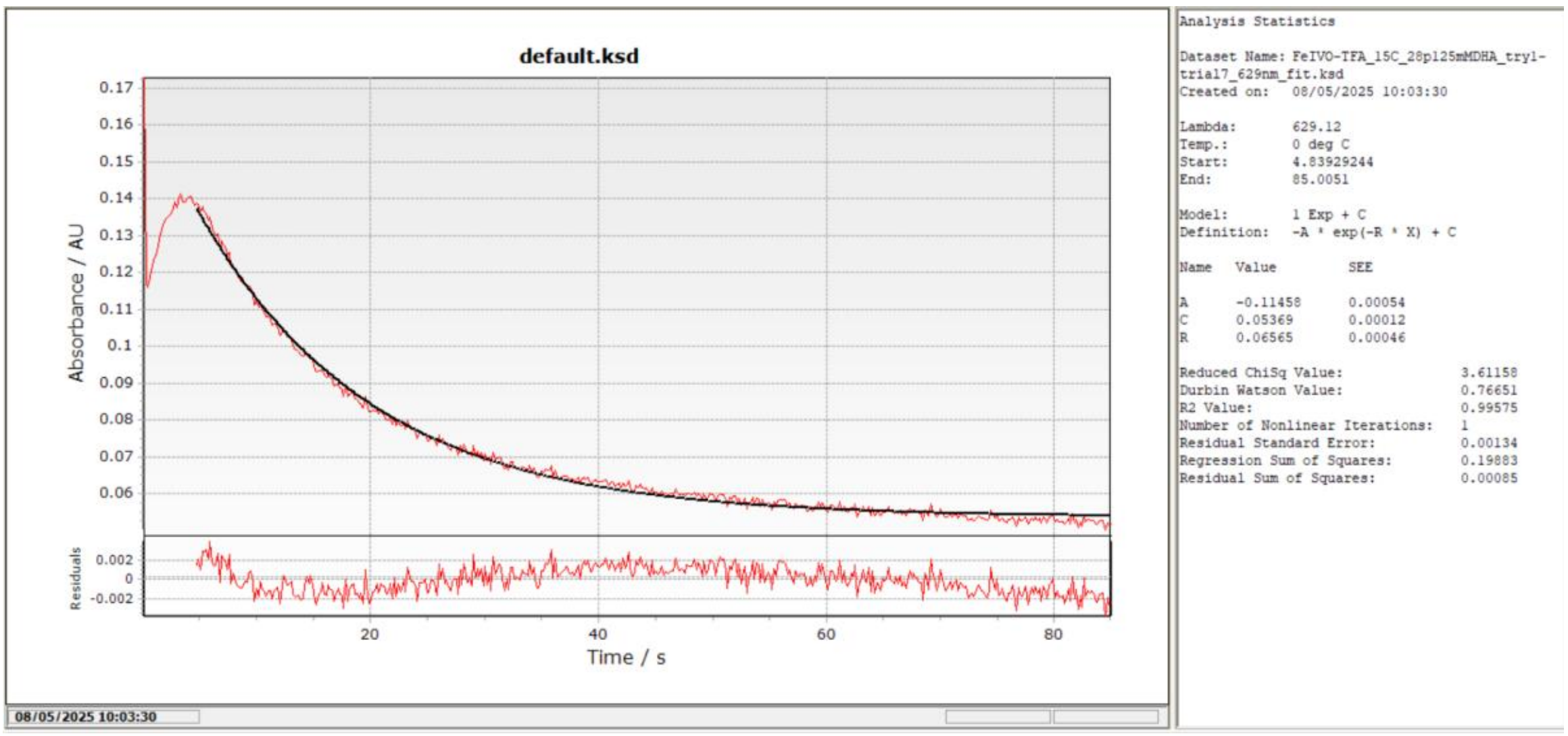

Figure S94. Exemplary trace (629 nm) and fit for the UV-Vis stopped-flow measurement of the reaction of **4** (1 mM) with 28.125 mM DHA at 15 °C in a mixture of MeCN and DCM.

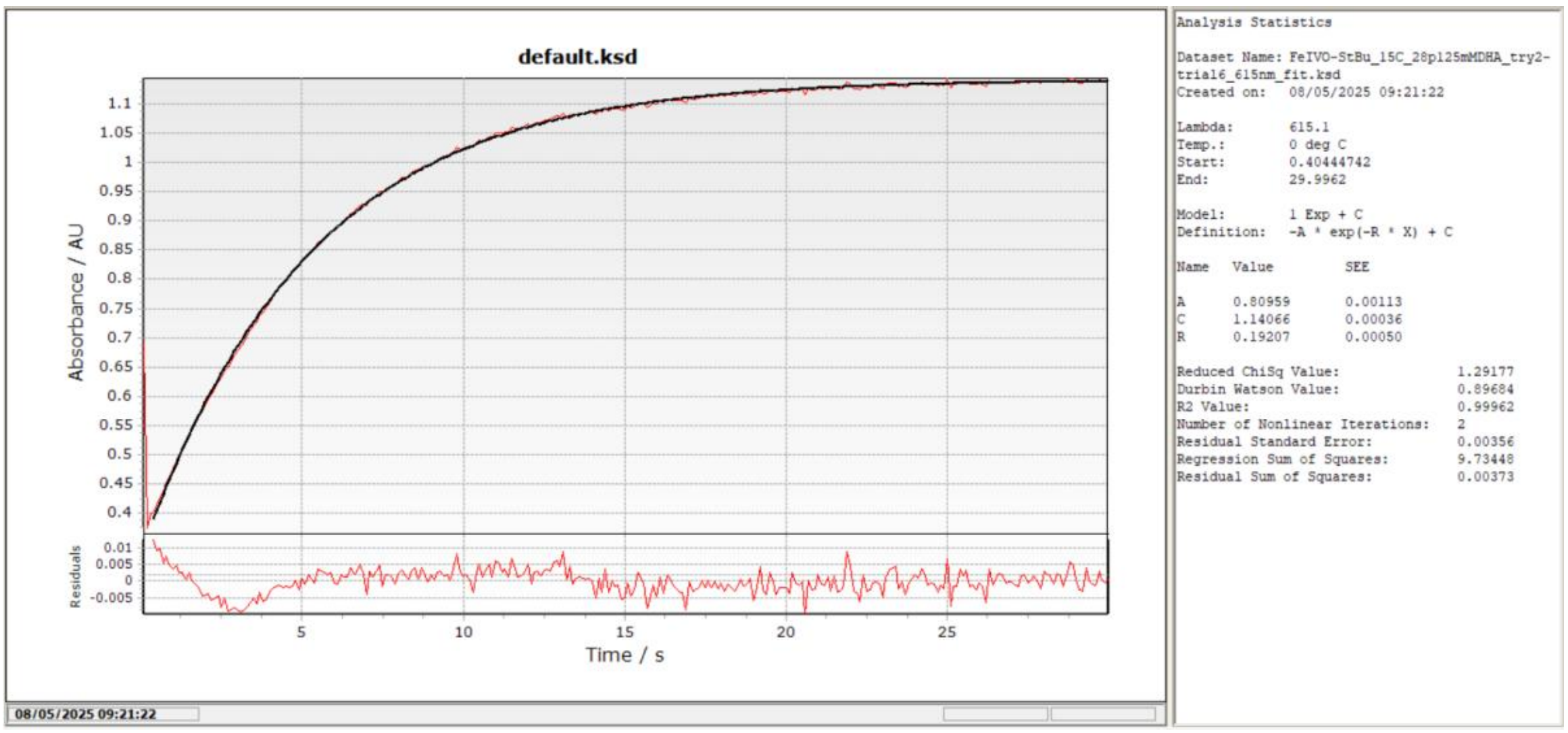

Figure S95. Exemplary trace (615 nm) and fit for the UV-Vis stopped-flow measurement of the reaction of **5a** (1 mM) with 28.125 mM DHA at 15 °C in a mixture of MeCN and DCM.

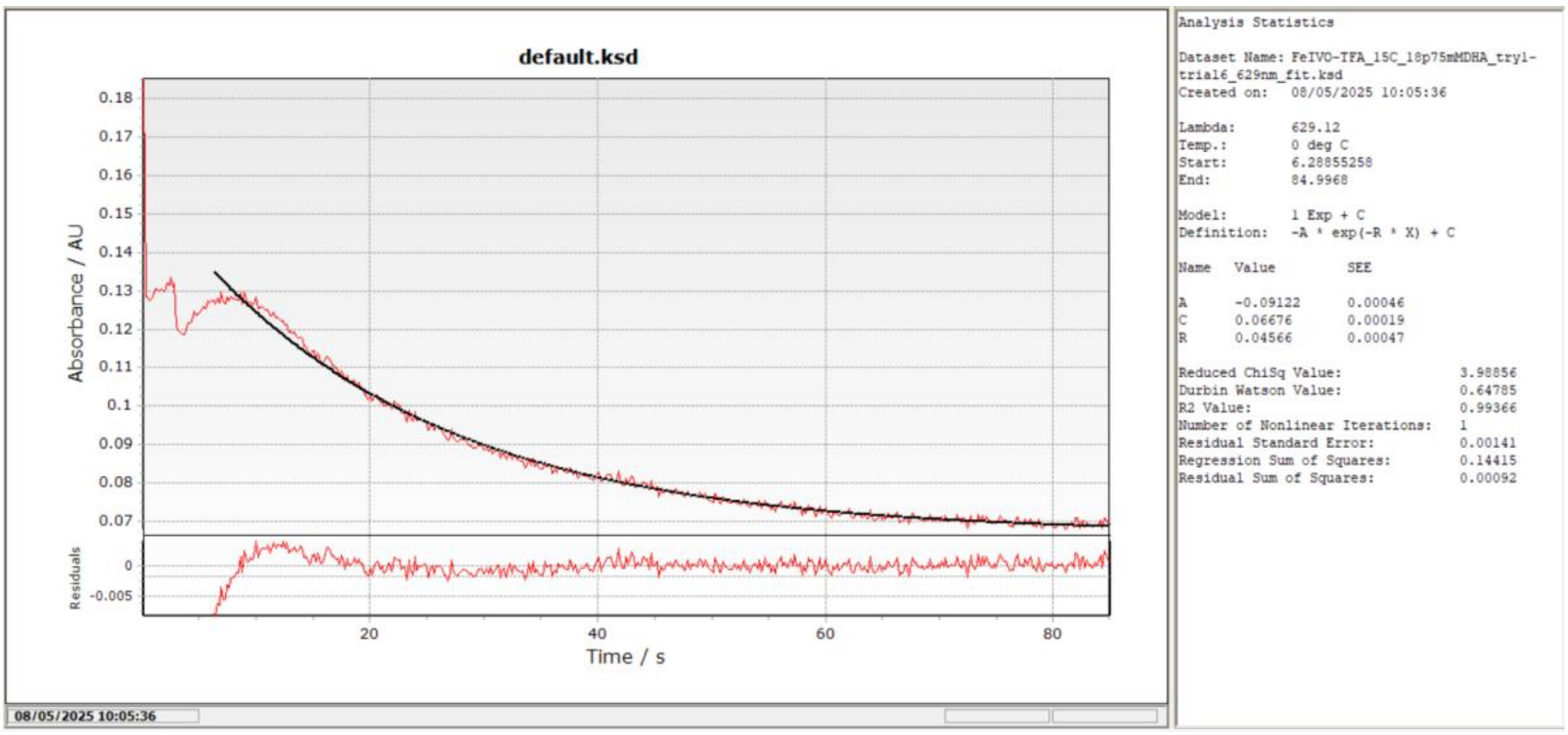

Figure S96. Exemplary trace (629 nm) and fit for the UV-Vis stopped-flow measurement of the reaction of **4** (1 mM) with 18.75 mM DHA at 15 °C in a mixture of MeCN and DCM.

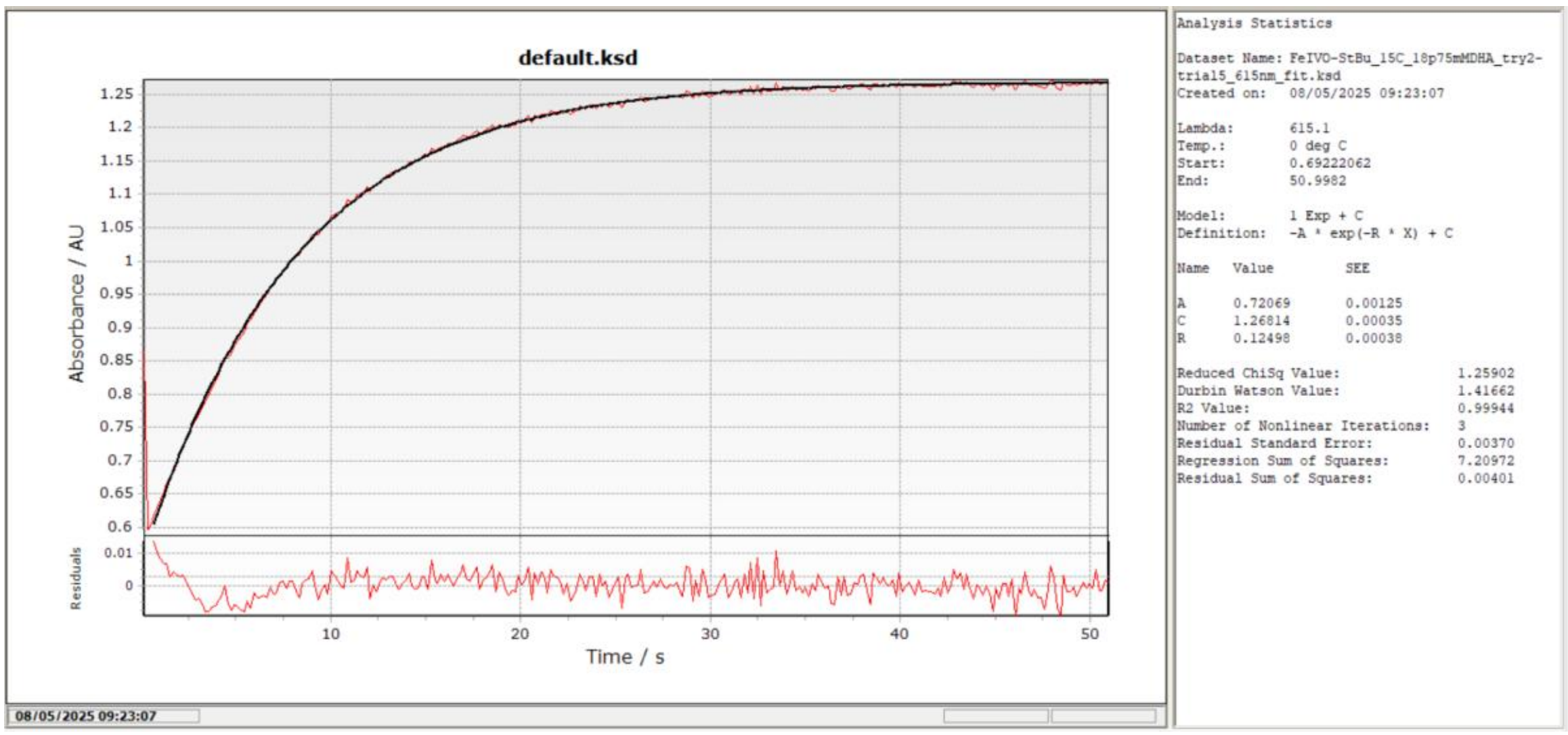

Figure S97. Exemplary trace (615 nm) and fit for the UV-Vis stopped-flow measurement of the reaction of **5a** (1 mM) with 18.75 mM DHA at 15 °C in a mixture of MeCN and DCM.

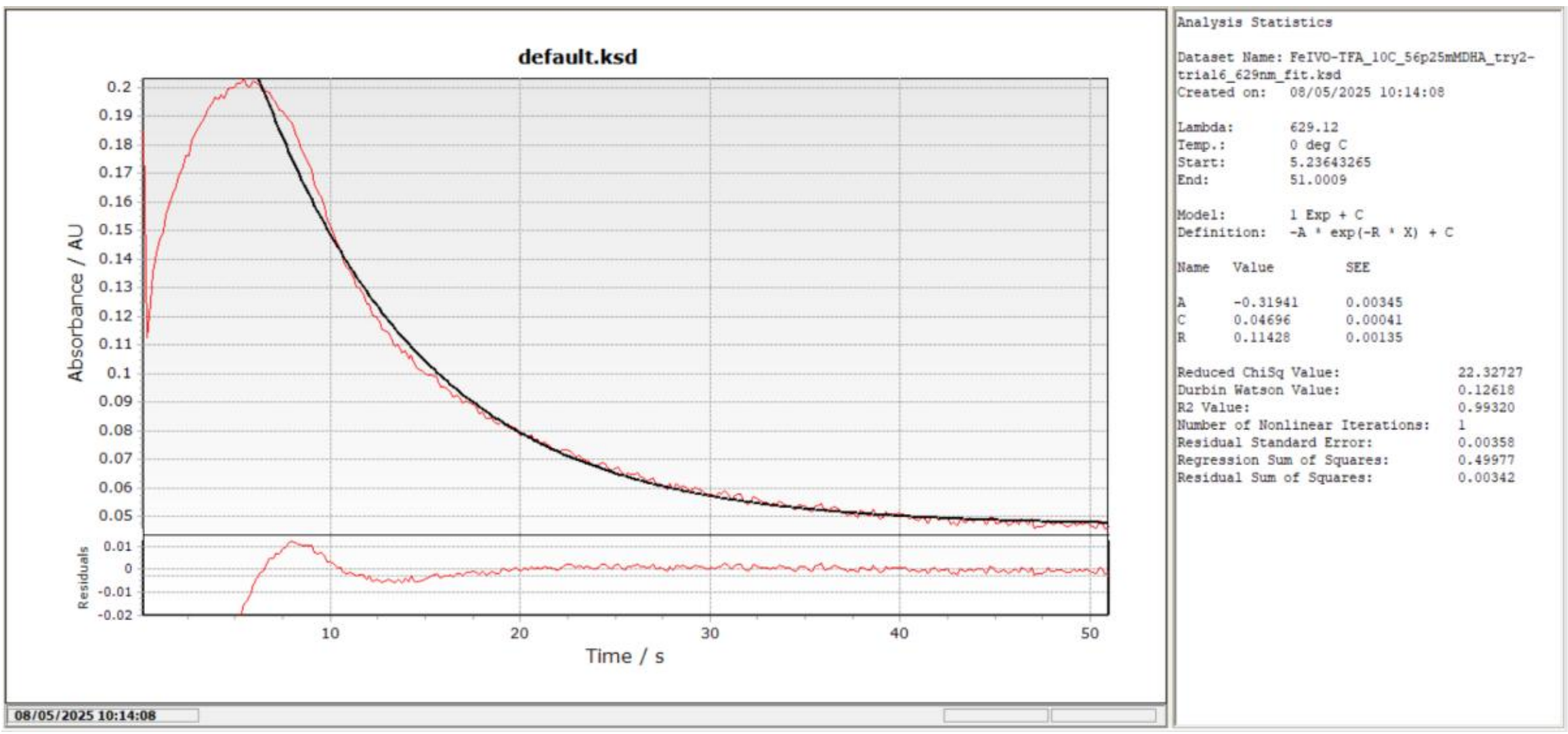

Figure S98. Exemplary trace (629 nm) and fit for the UV-Vis stopped-flow measurement of the reaction of **4** (1 mM) with 56.25 mM DHA at 10 °C in a mixture of MeCN and DCM.

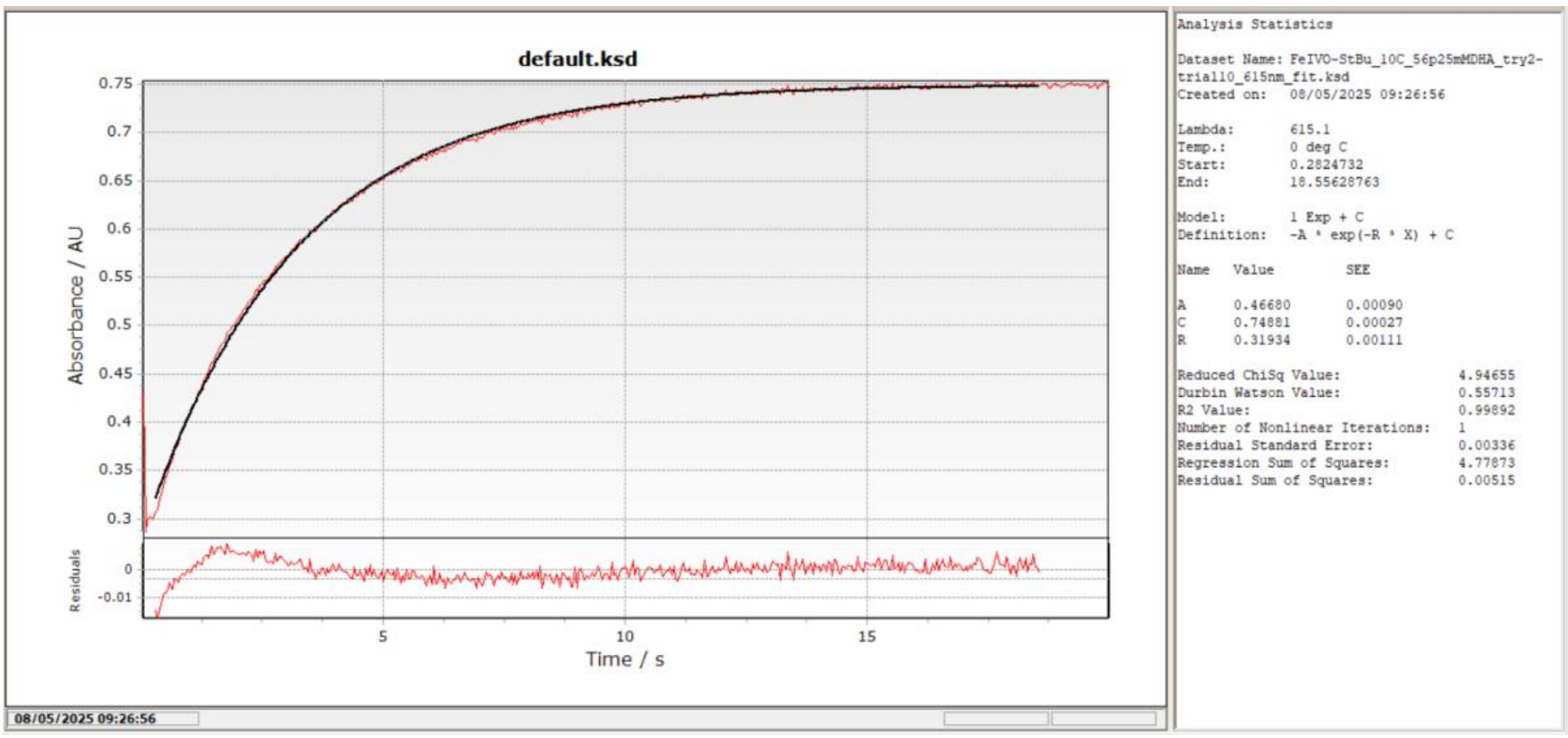

Figure S99. Exemplary trace (615 nm) and fit for the UV-Vis stopped-flow measurement of the reaction of **5a** (1 mM) with 56.25 mM DHA at 10 °C in a mixture of MeCN and DCM.

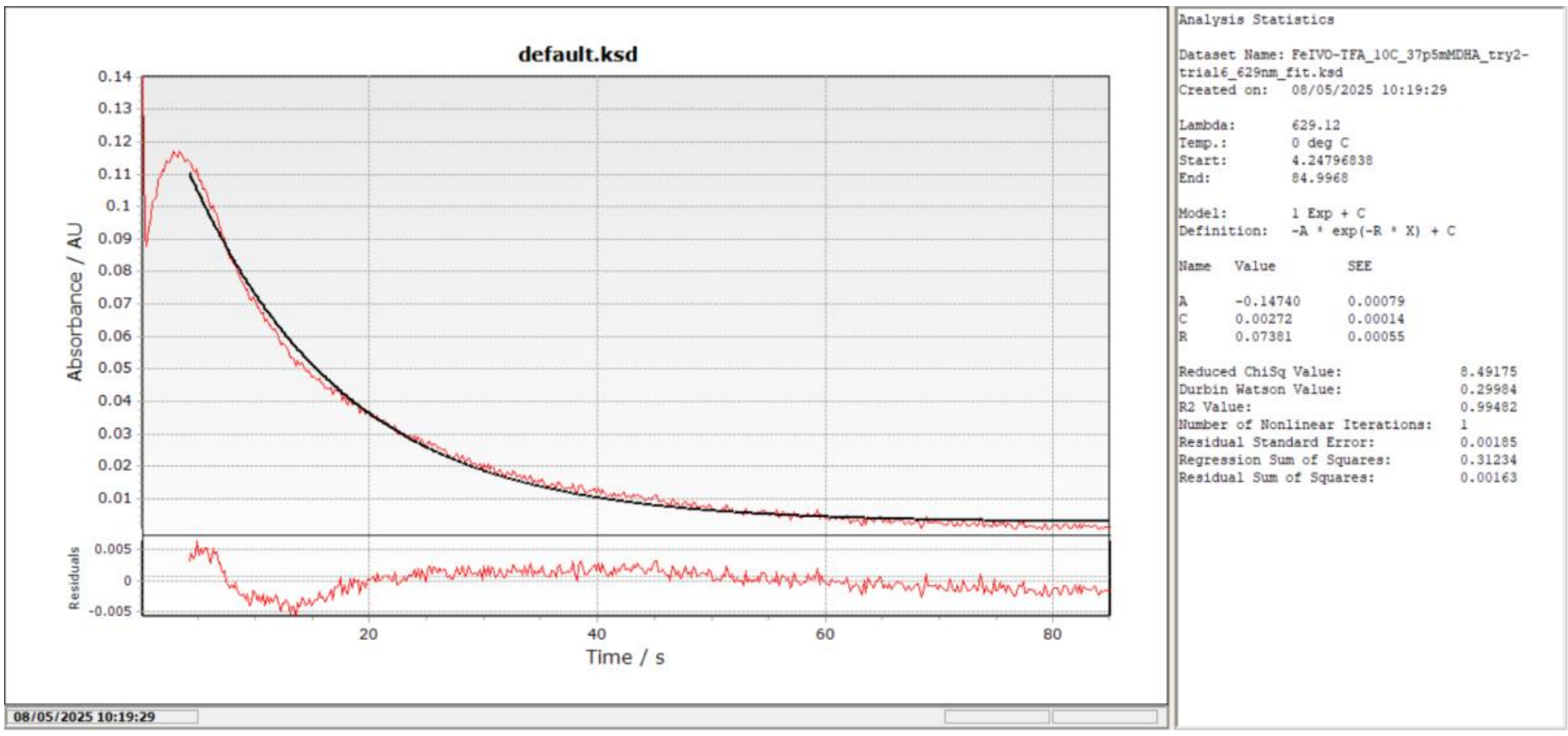

Figure S100. Exemplary trace (629 nm) and fit for the UV-Vis stopped-flow measurement of the reaction of **4** (1 mM) with 37.5 mM DHA at 10 °C in a mixture of MeCN and DCM.

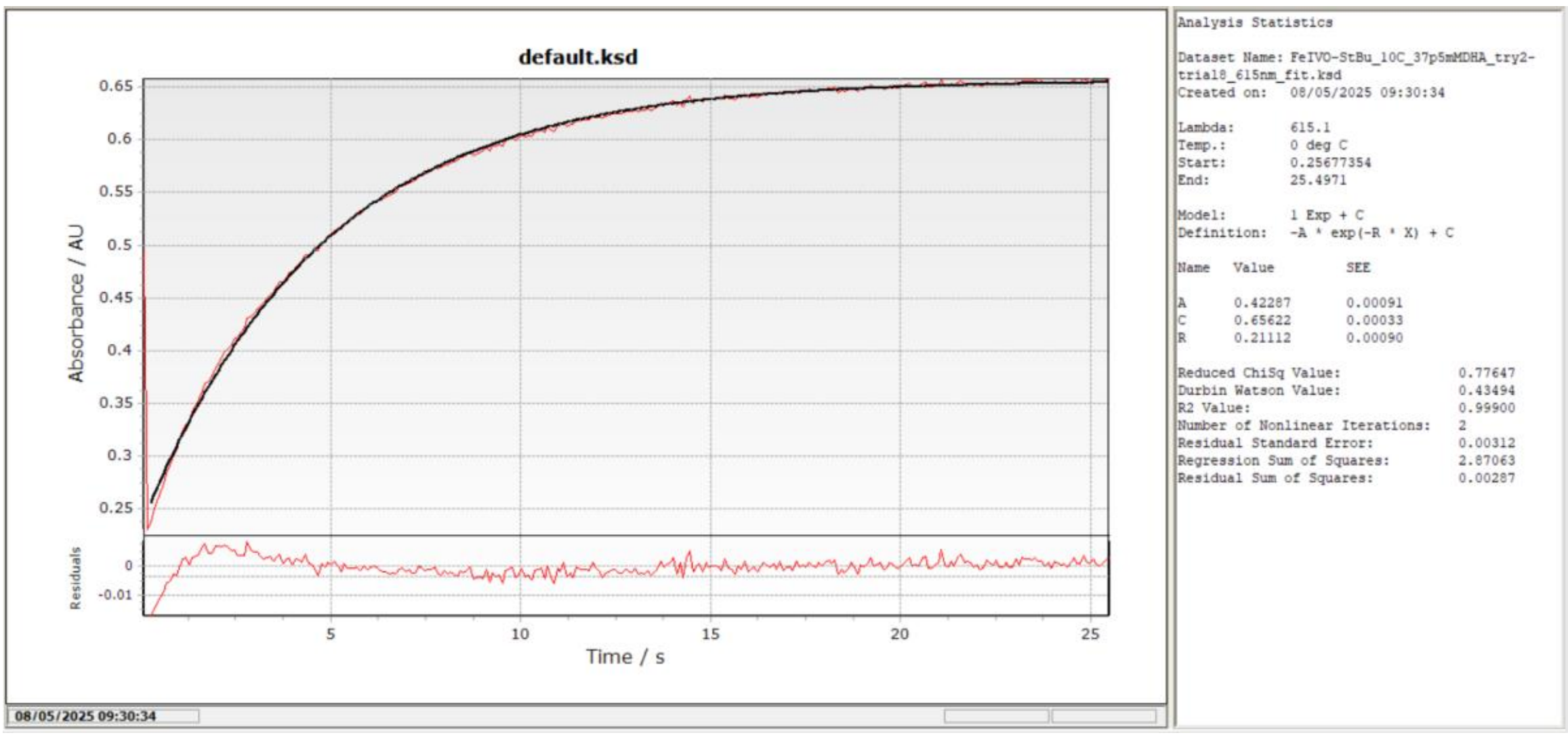

Figure S101. Exemplary trace (615 nm) and fit for the UV-Vis stopped-flow measurement of the reaction of **5a** (1 mM) with 37.5 mM DHA at 10 °C in a mixture of MeCN and DCM.

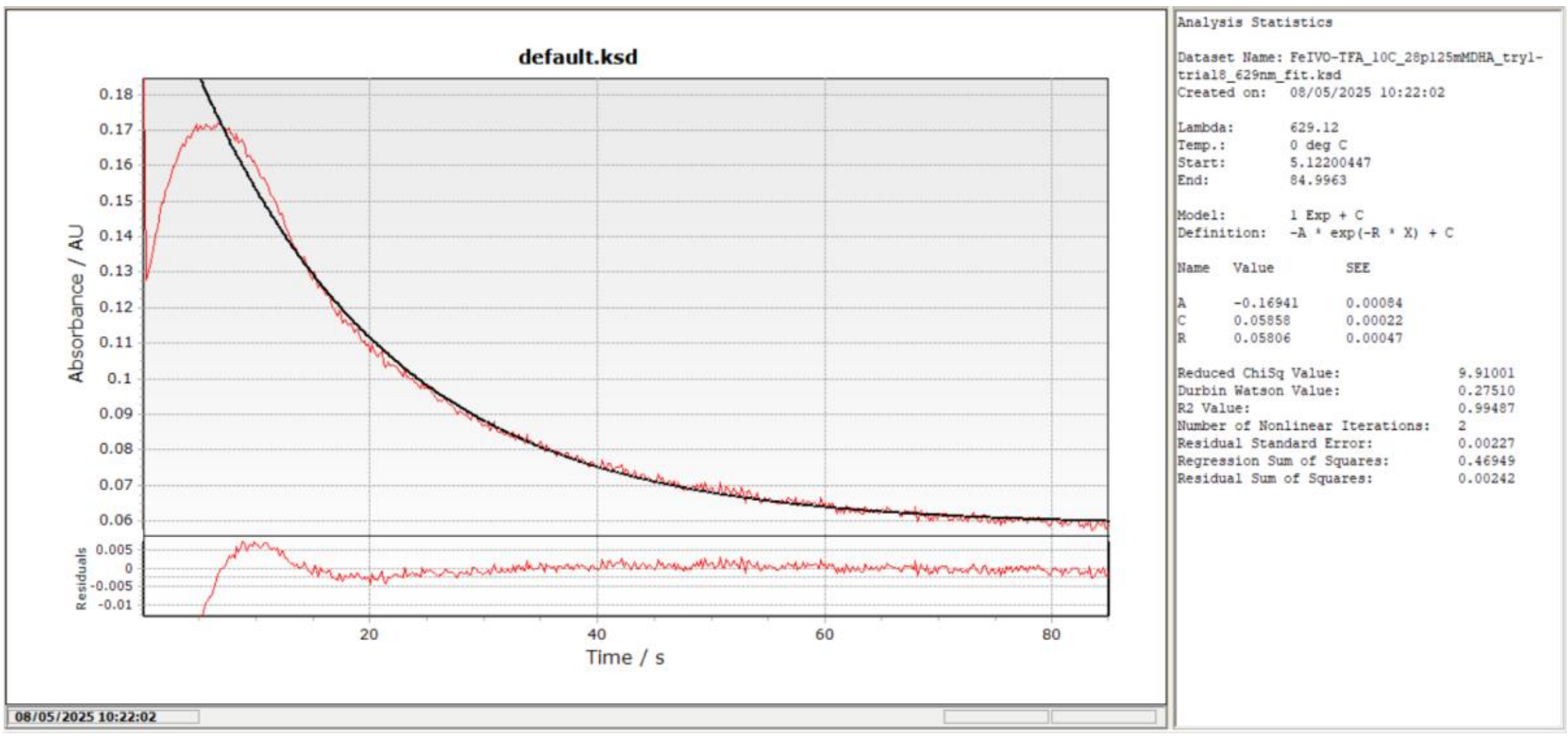

Figure S102. Exemplary trace (629 nm) and fit for the UV-Vis stopped-flow measurement of the reaction of **4** (1 mM) with 28.125 mM DHA at 10 °C in a mixture of MeCN and DCM.

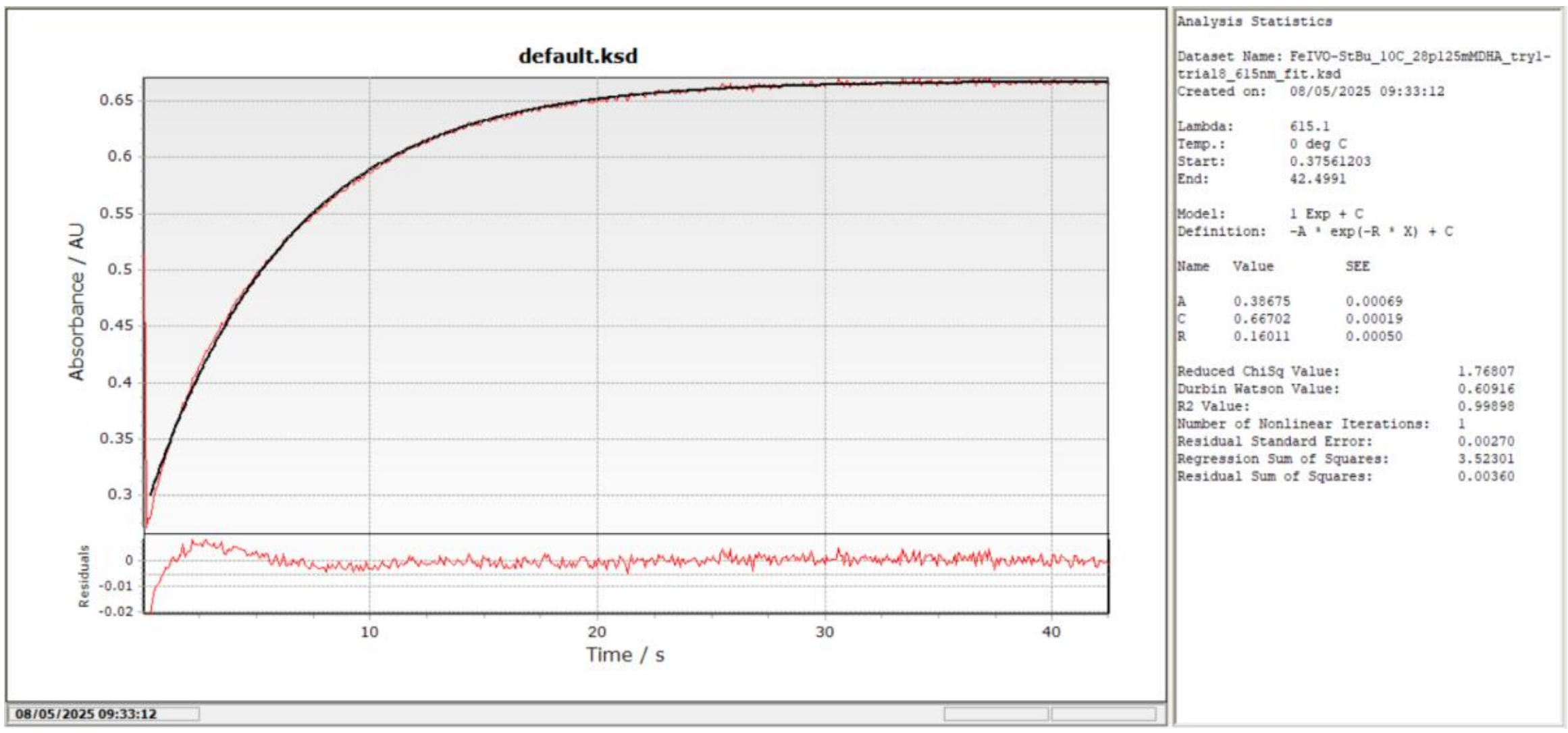

Figure S103. Exemplary trace (615 nm) and fit for the UV-Vis stopped-flow measurement of the reaction of **5a** (1 mM) with 28.125 mM DHA at 10 °C in a mixture of MeCN and DCM.

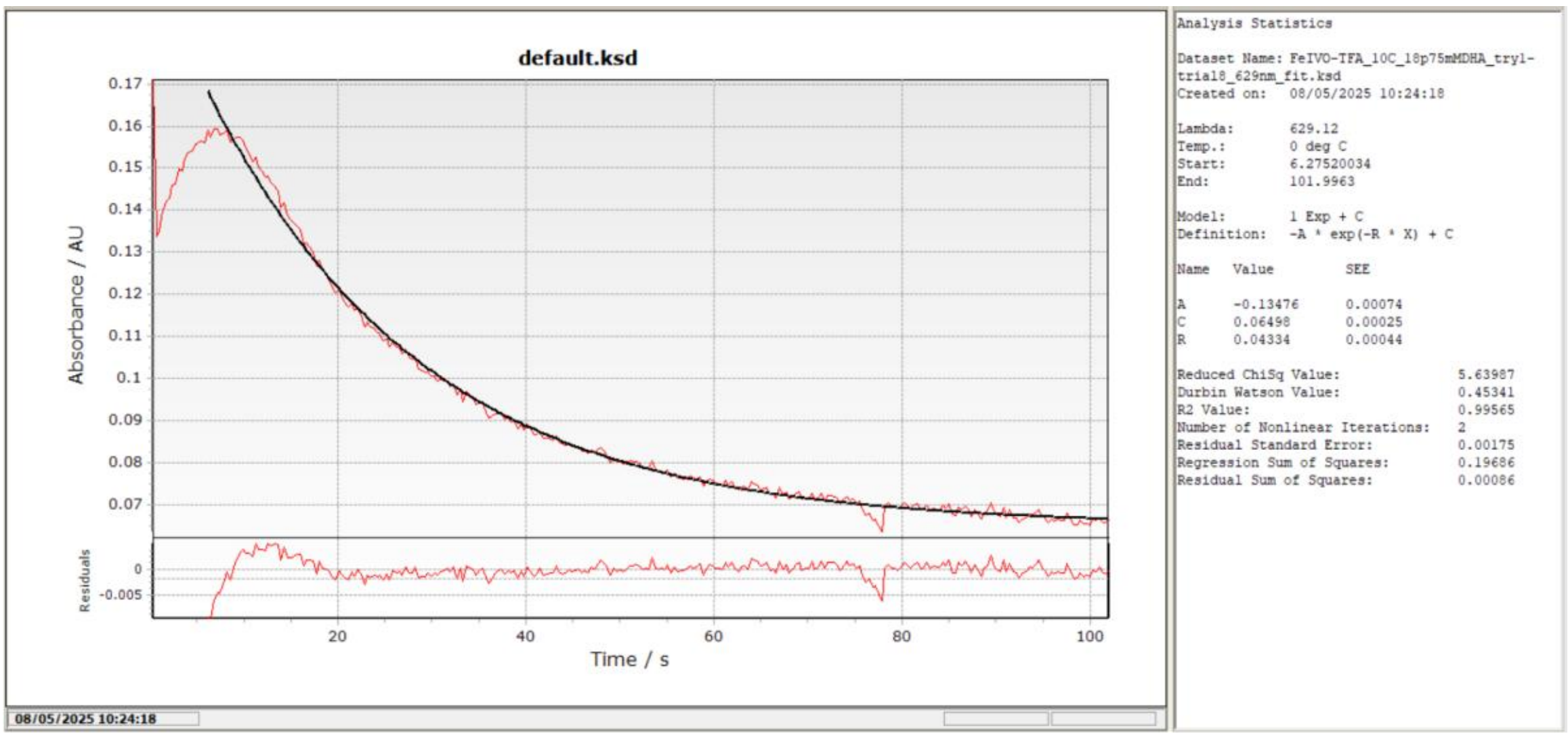

Figure S104. Exemplary trace (629 nm) and fit for the UV-Vis stopped-flow measurement of the reaction of **4** (1 mM) with 18.75 mM DHA at 10 °C in a mixture of MeCN and DCM.

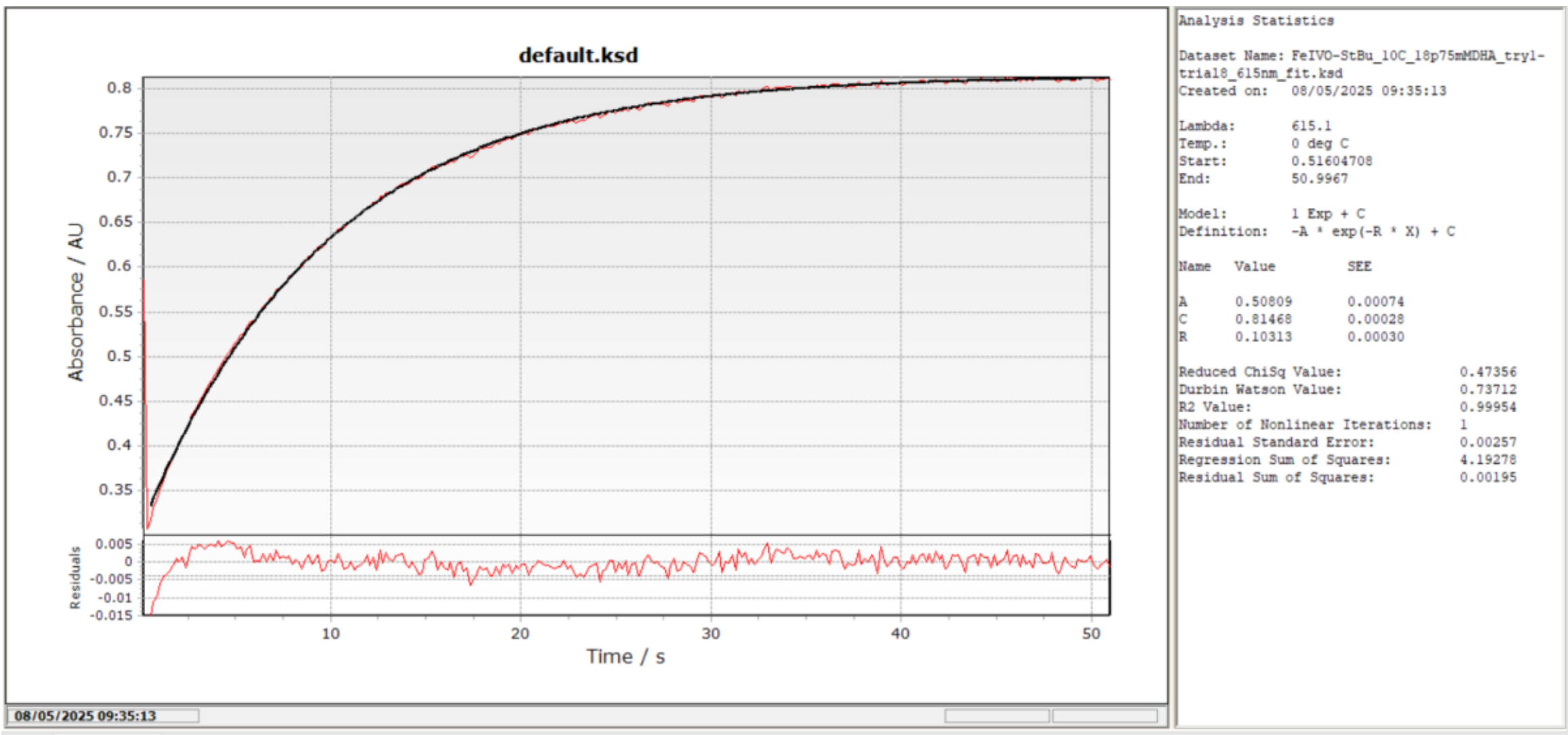

Figure S105. Exemplary trace (615 nm) and fit for the UV-Vis stopped-flow measurement of the reaction of **5a** (1 mM) with 18.75 mM DHA at 10 °C in a mixture of MeCN and DCM.

Kinetic (stopped-flow) UV-Vis measurements with DHA-d_4:

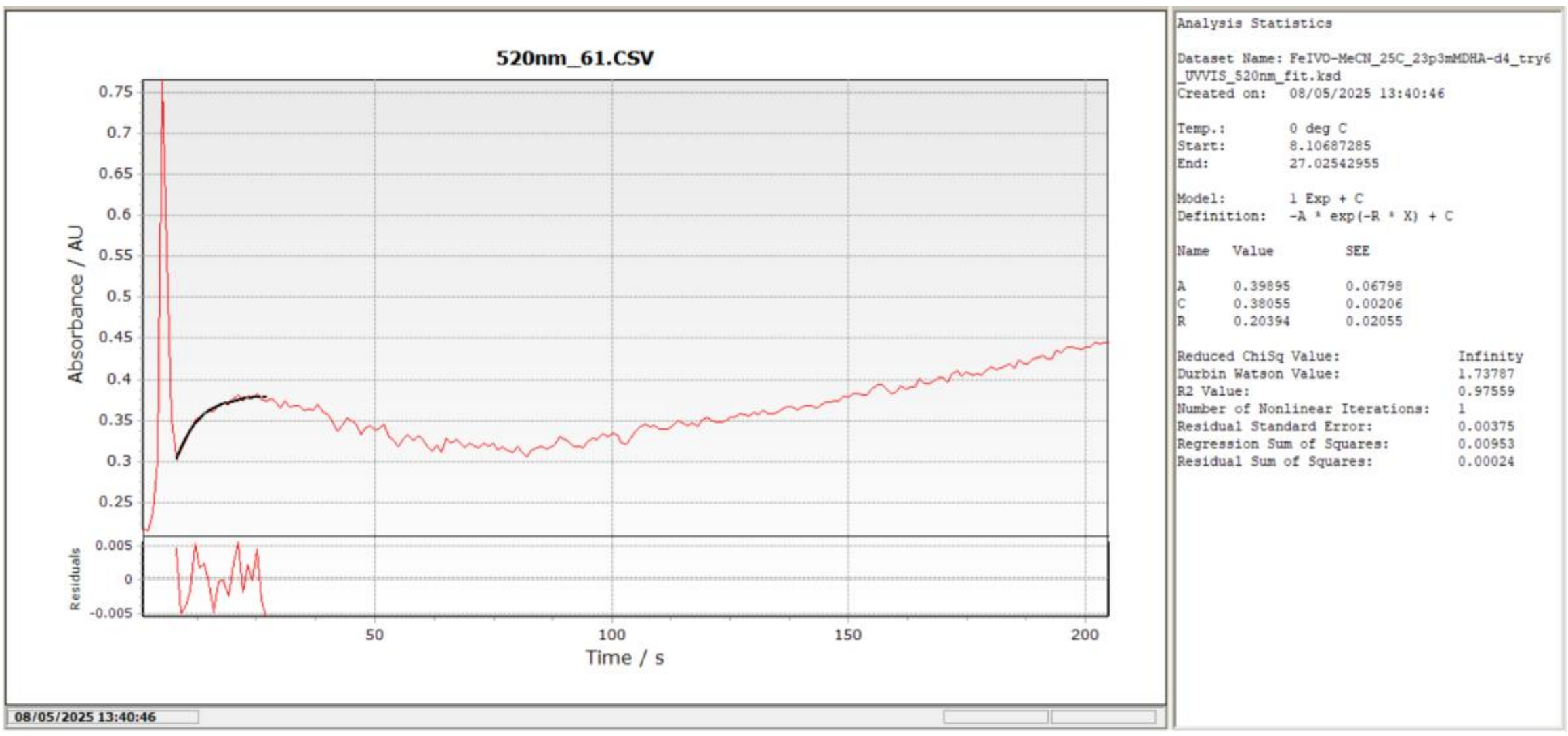

Figure S106. Exemplary trace (520 nm) and fit for the UV-Vis measurement of the reaction of **3** (1 mM) with 23.3 mM DHA-d_4 at 25 °C in a mixture of MeCN and DCM.

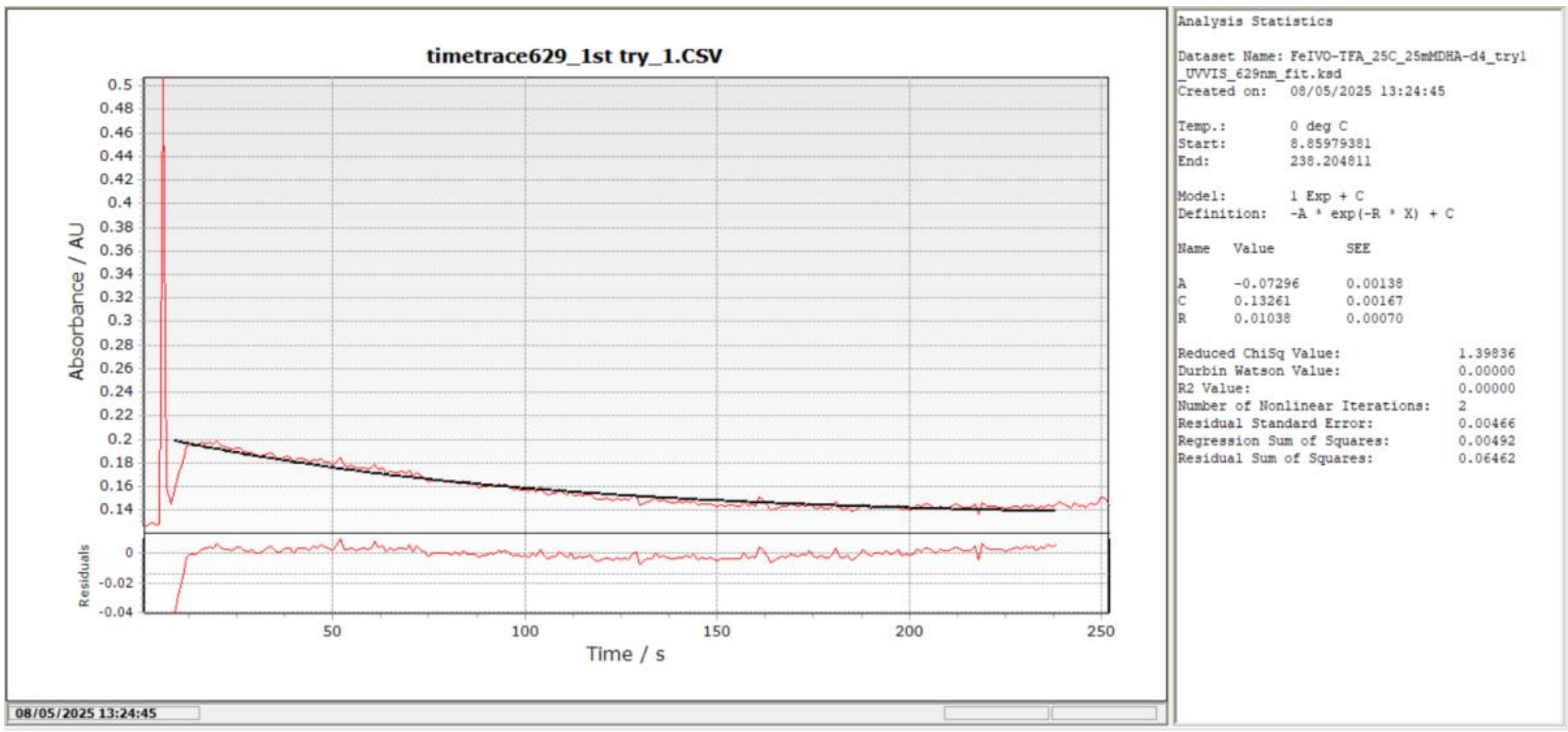

Figure S107. Exemplary trace (629 nm) and fit for the UV-Vis measurement of the reaction of **4** (1 mM) with 25 mM DHA-d_4 at 25 °C in a mixture of MeCN and DCM.

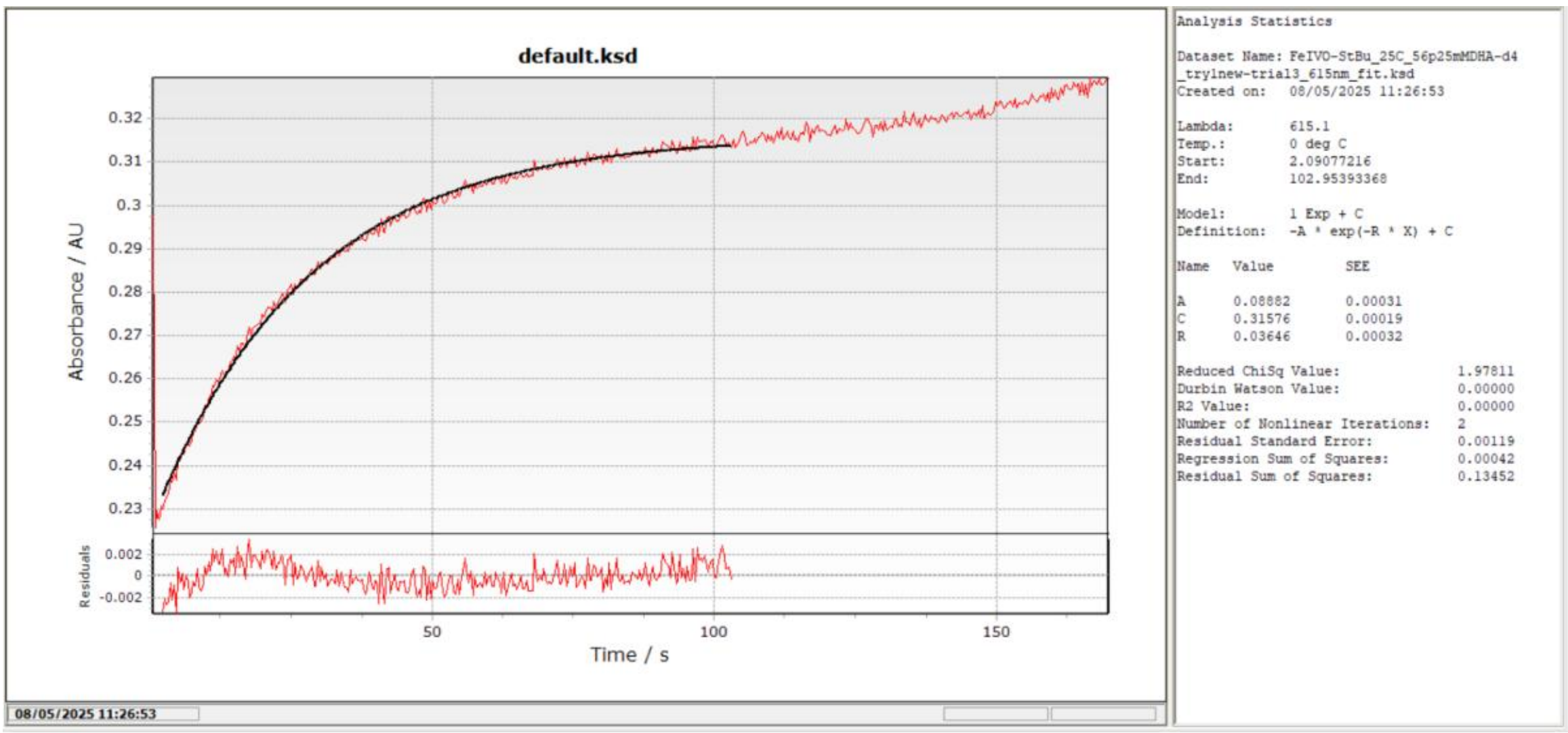

Figure S108. Exemplary trace (615 nm) and fit for the UV-Vis stopped-flow measurement of the reaction of **5a** (1 mM) with 56.25 mM DHA-d_4 at 25 °C in a mixture of MeCN and DCM.

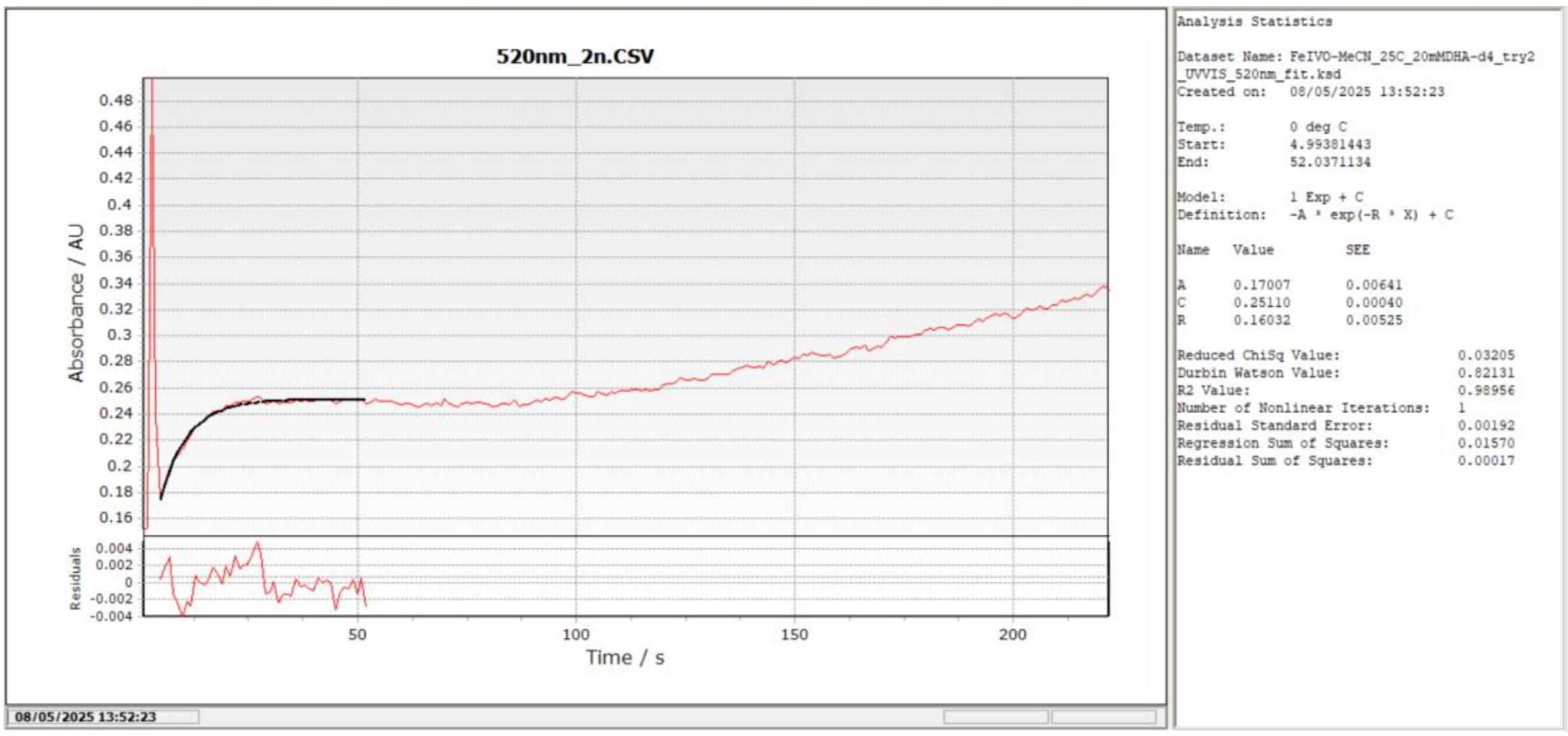

Figure S109. Exemplary trace (520 nm) and fit for the UV-Vis measurement of the reaction of **3** (1 mM) with 20 mM DHA-d_4 at 25 °C in a mixture of MeCN and DCM.

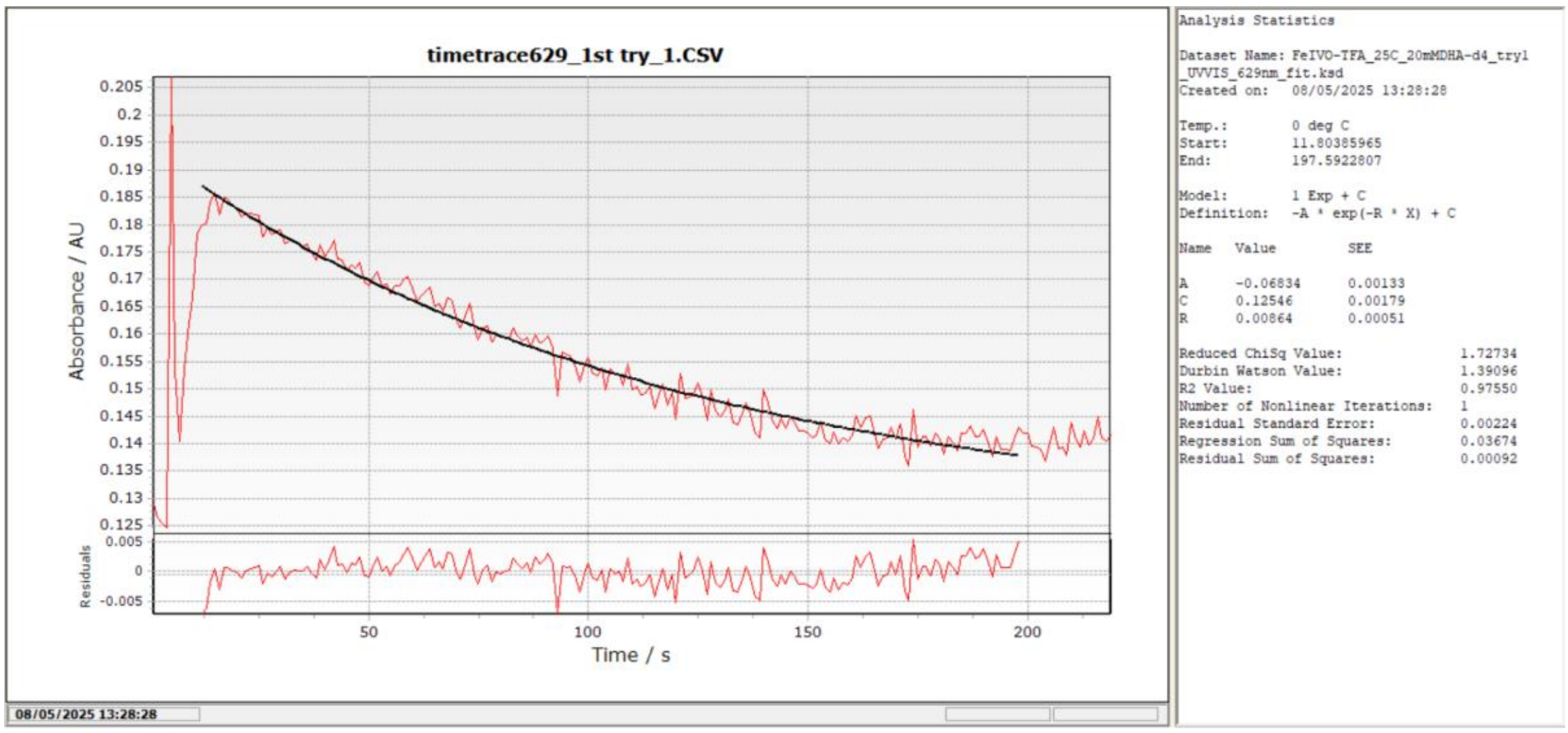

Figure S110. Exemplary trace (629 nm) and fit for the UV-Vis measurement of the reaction of **4** (1 mM) with 20 mM DHA-d_4 at 25 °C in a mixture of MeCN and DCM.

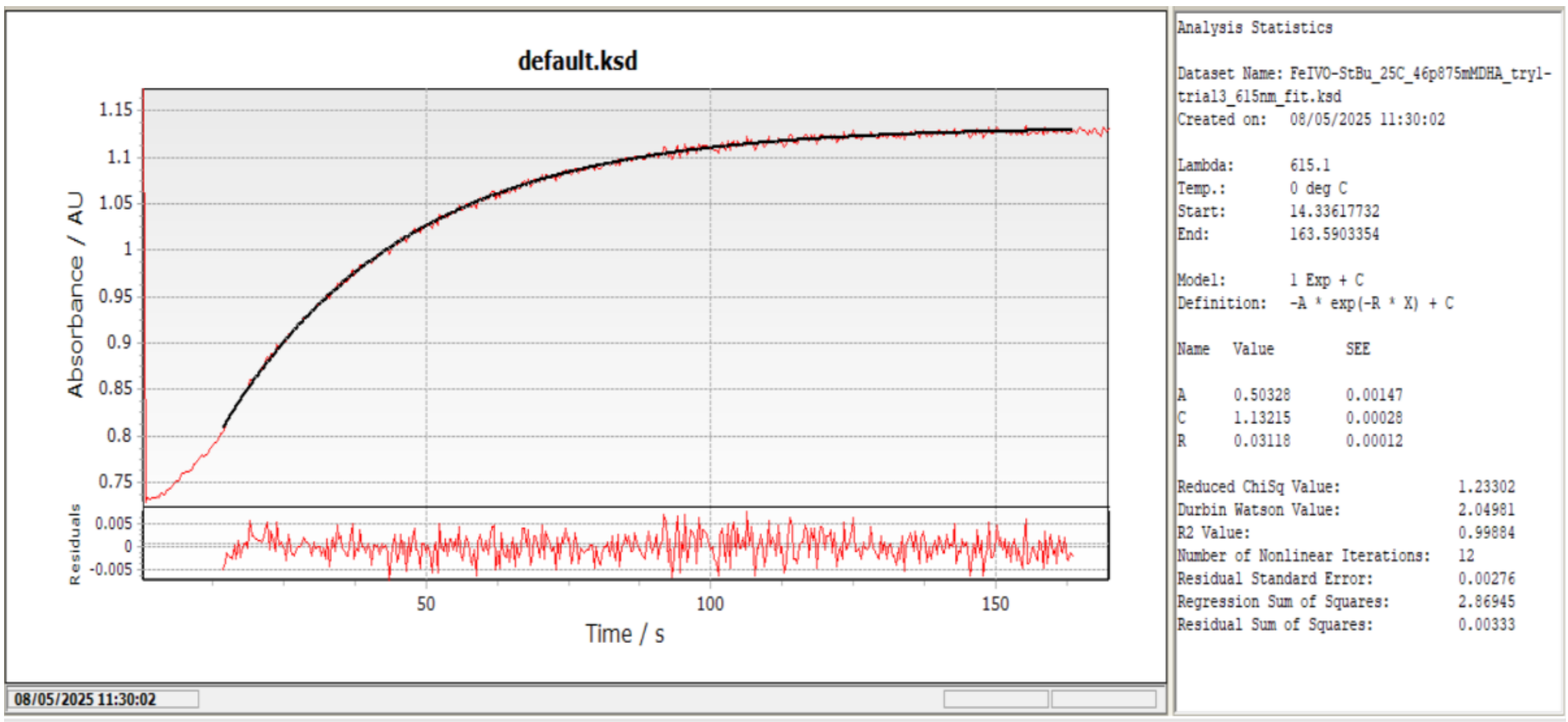

Figure S111. Exemplary trace (615 nm) and fit for the UV-Vis stopped-flow measurement of the reaction of **5a** (1 mM) with 46.875 mM DHA-d_4 at 25 °C in a mixture of MeCN and DCM.

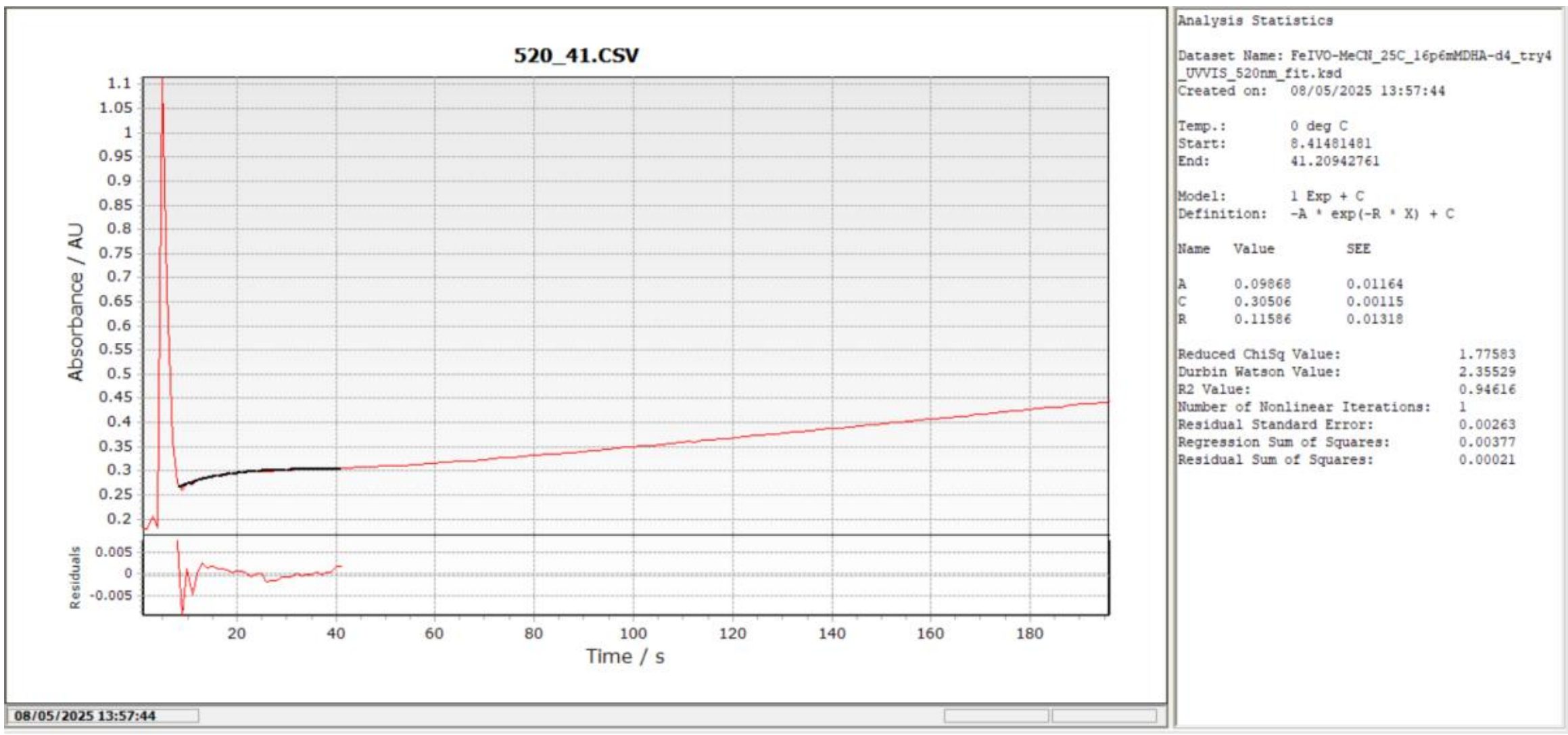

Figure S112. Exemplary trace (520 nm) and fit for the UV-Vis measurement of the reaction of **3** (1 mM) with 16.6 mM DHA-d_4 at 25 °C in a mixture of MeCN and DCM.

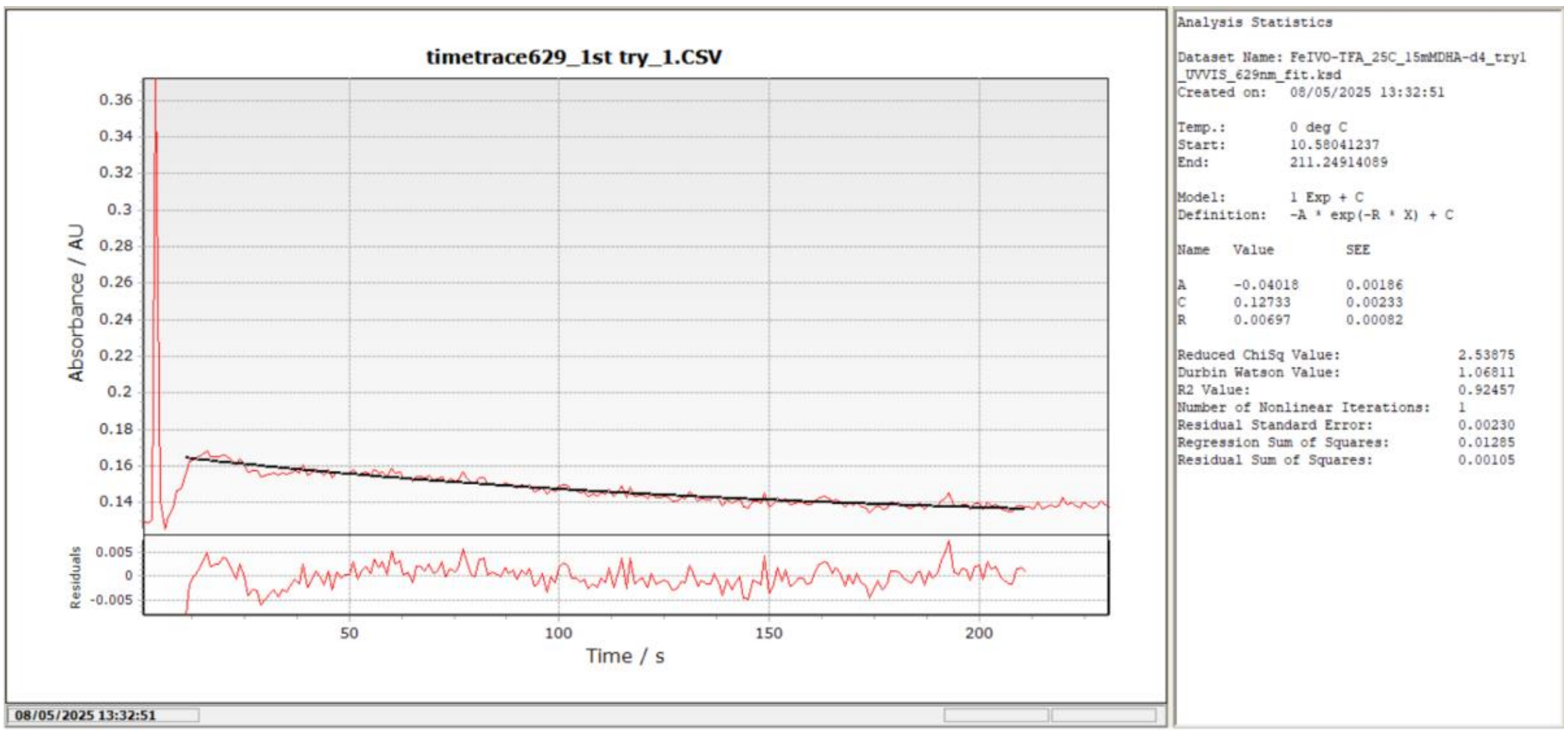

Figure S113. Exemplary trace (629 nm) and fit for the UV-Vis measurement of the reaction of **4** (1 mM) with 15 mM DHA-d_4 at 25 °C in a mixture of MeCN and DCM.

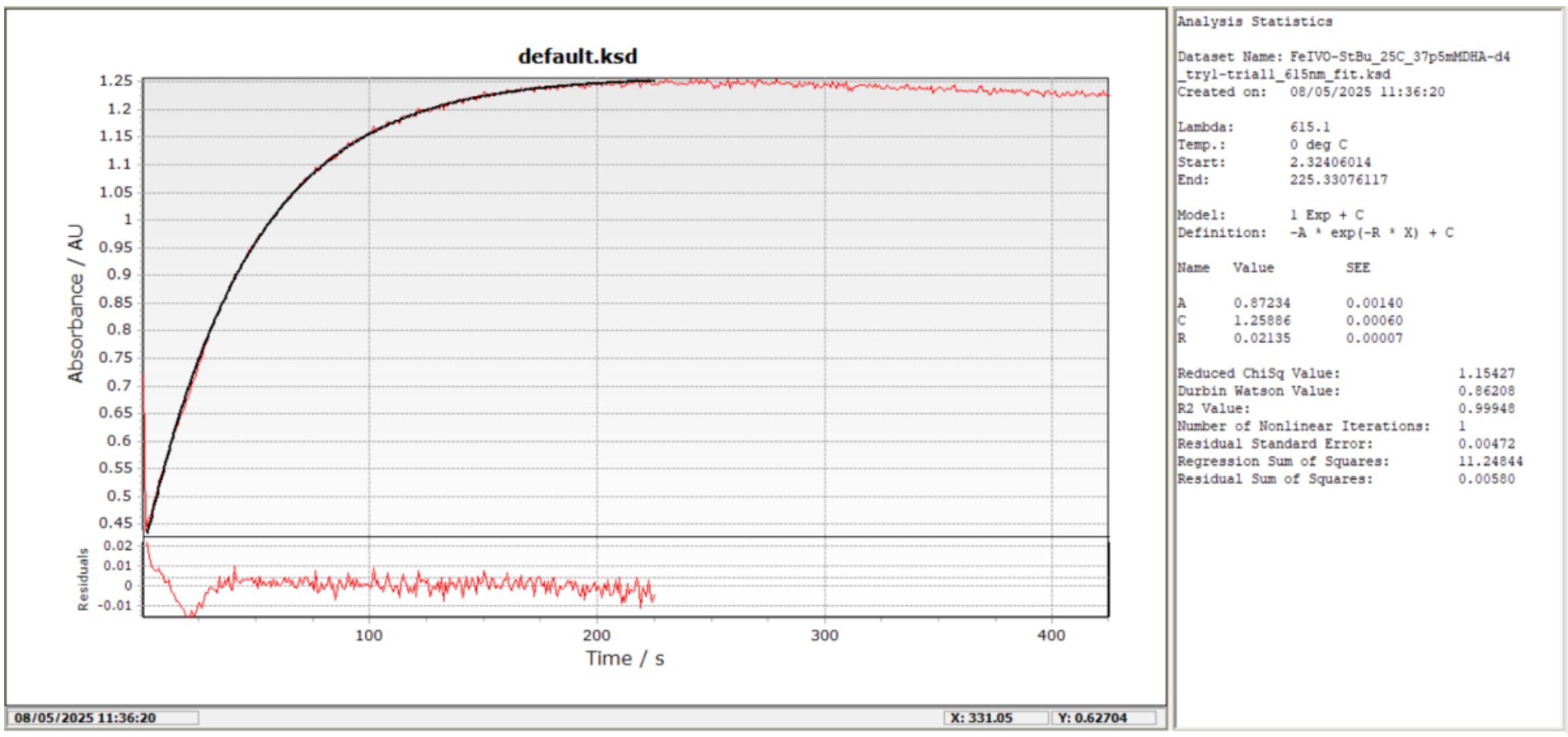

Figure S114. Exemplary trace (615 nm) and fit for the UV-Vis stopped-flow measurement of the reaction of **5a** (1 mM) with 37.5 mM DHA-d_4 at 25 °C in a mixture of MeCN and DCM.

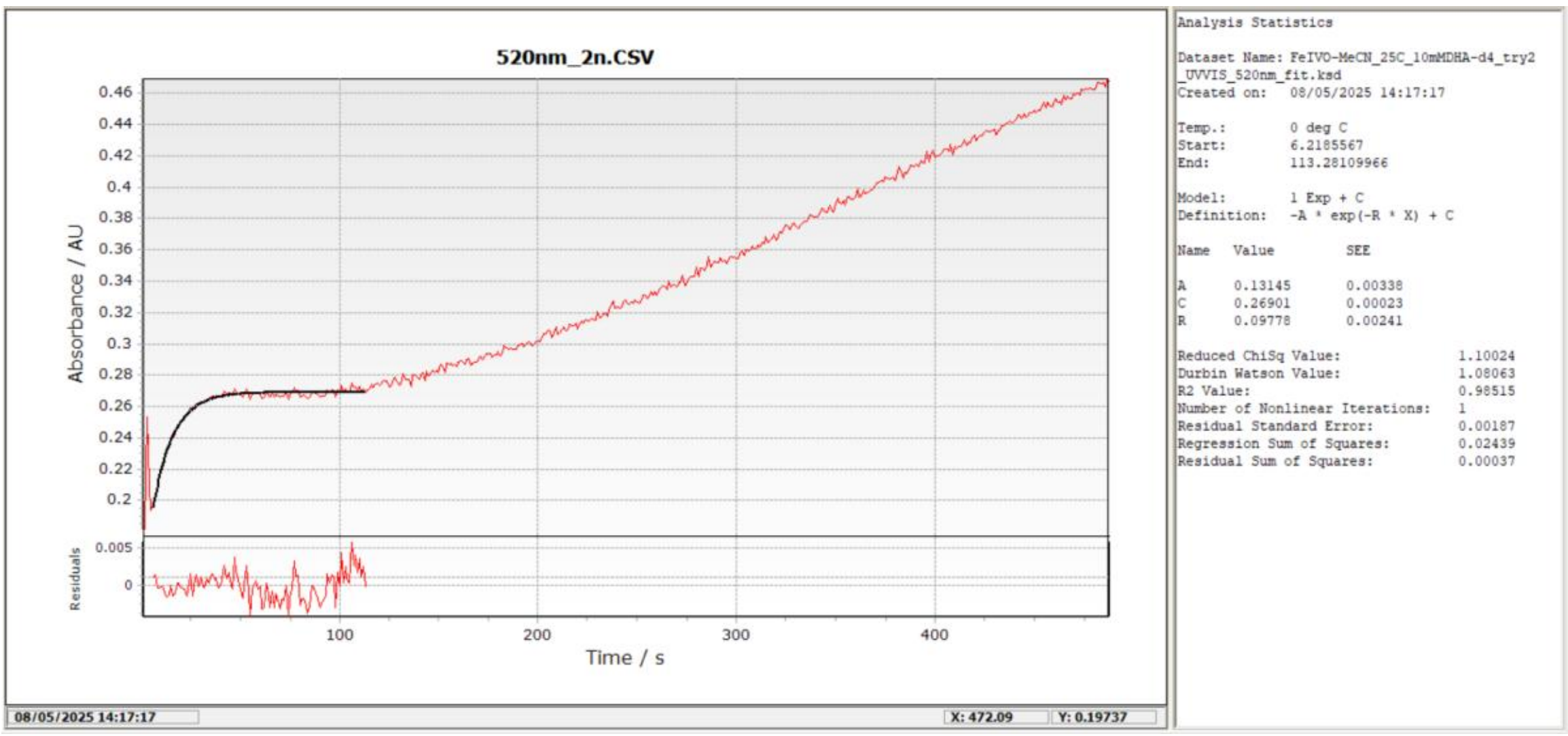

Figure S115. Exemplary trace (520 nm) and fit for the UV-Vis measurement of the reaction of **3** (1 mM) with 10 mM DHA-d_4 at 25 °C in a mixture of MeCN and DCM.

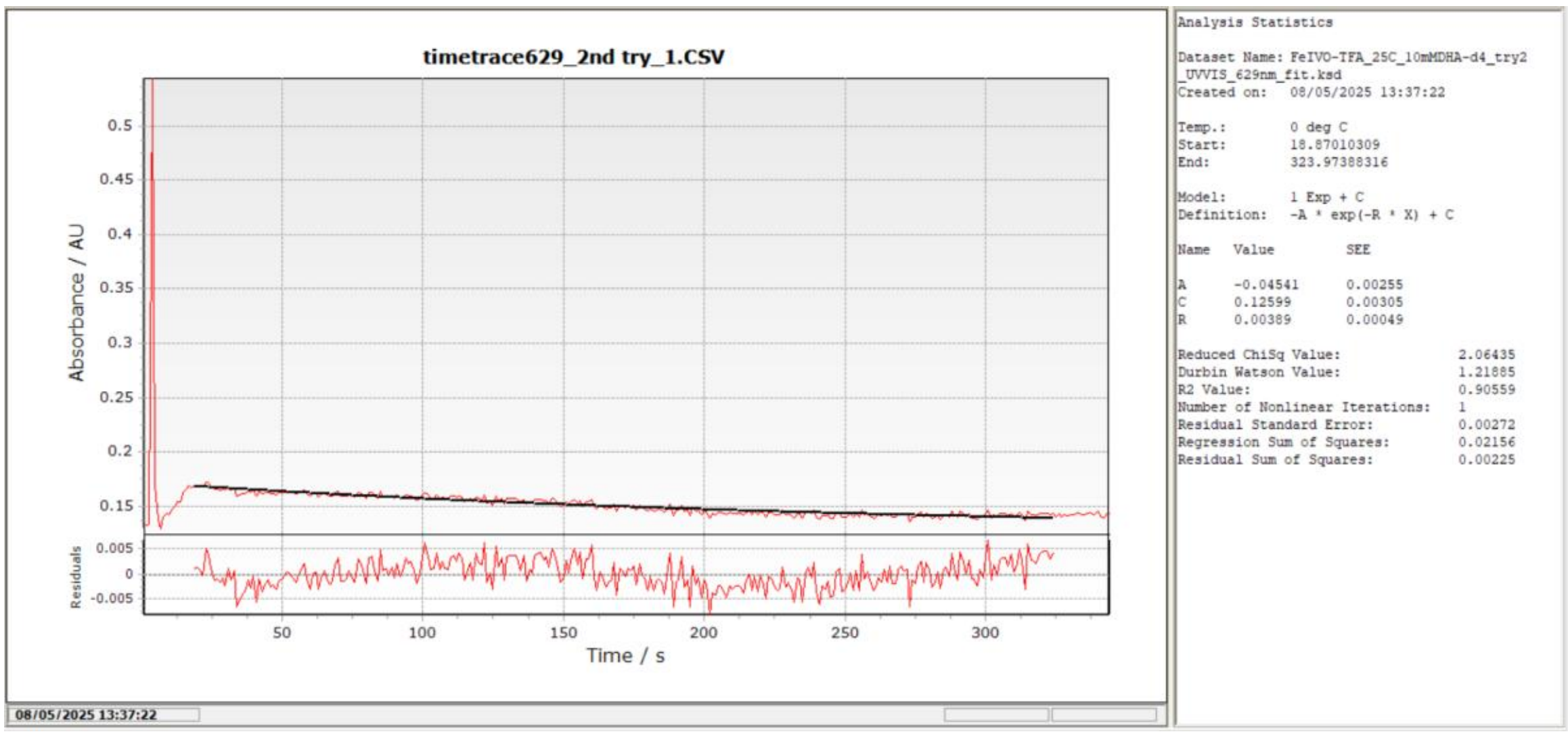

Figure S116. Exemplary trace (629 nm) and fit for the UV-Vis measurement of the reaction of **4** (1 mM) with 10 mM DHA-d_4 at 25 °C in a mixture of MeCN and DCM.

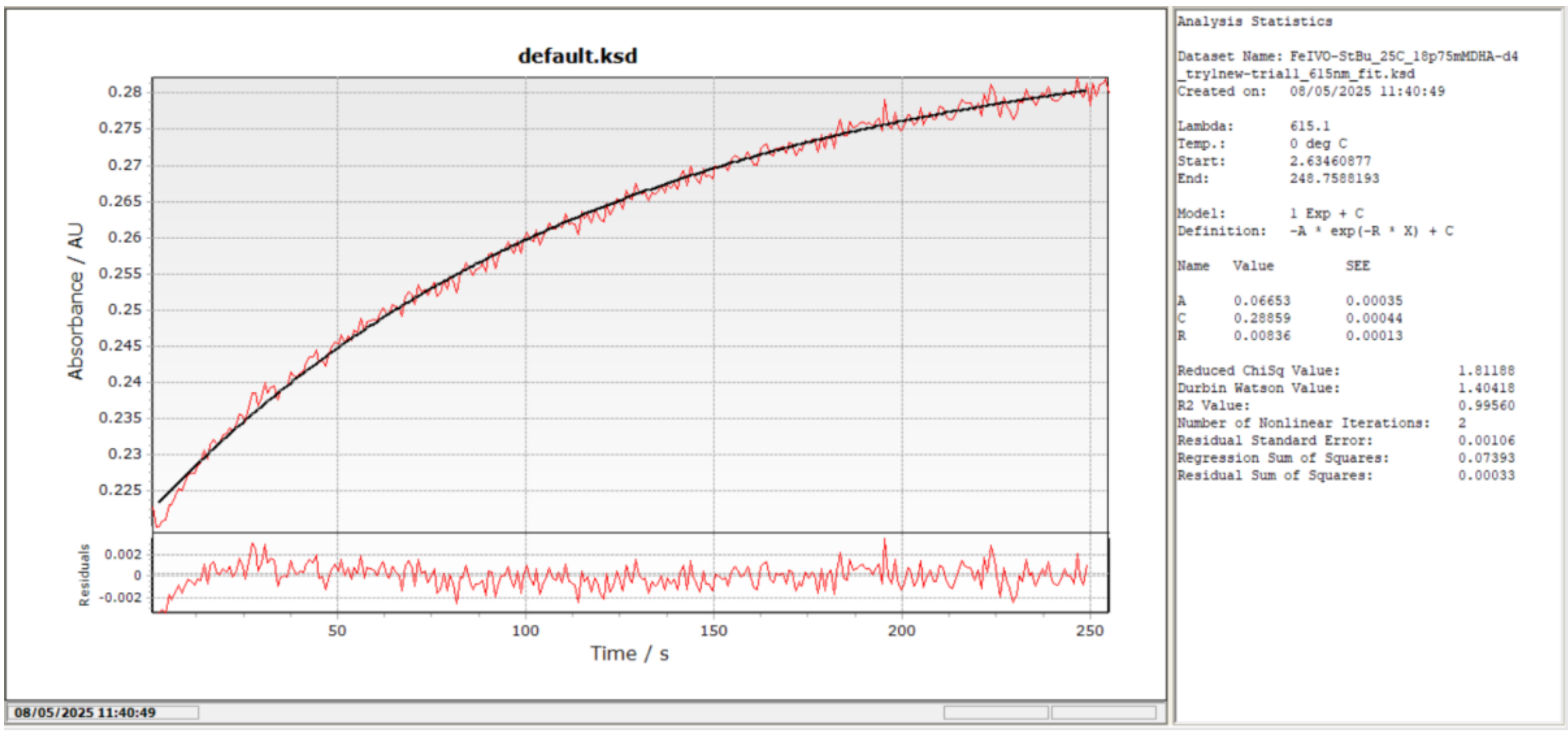

Figure S117. Exemplary trace (615 nm) and fit for the UV-Vis stopped-flow measurement of the reaction of **5a** (1 mM) with 18.75 mM DHA-d_4 at 25 °C in a mixture of MeCN and DCM.

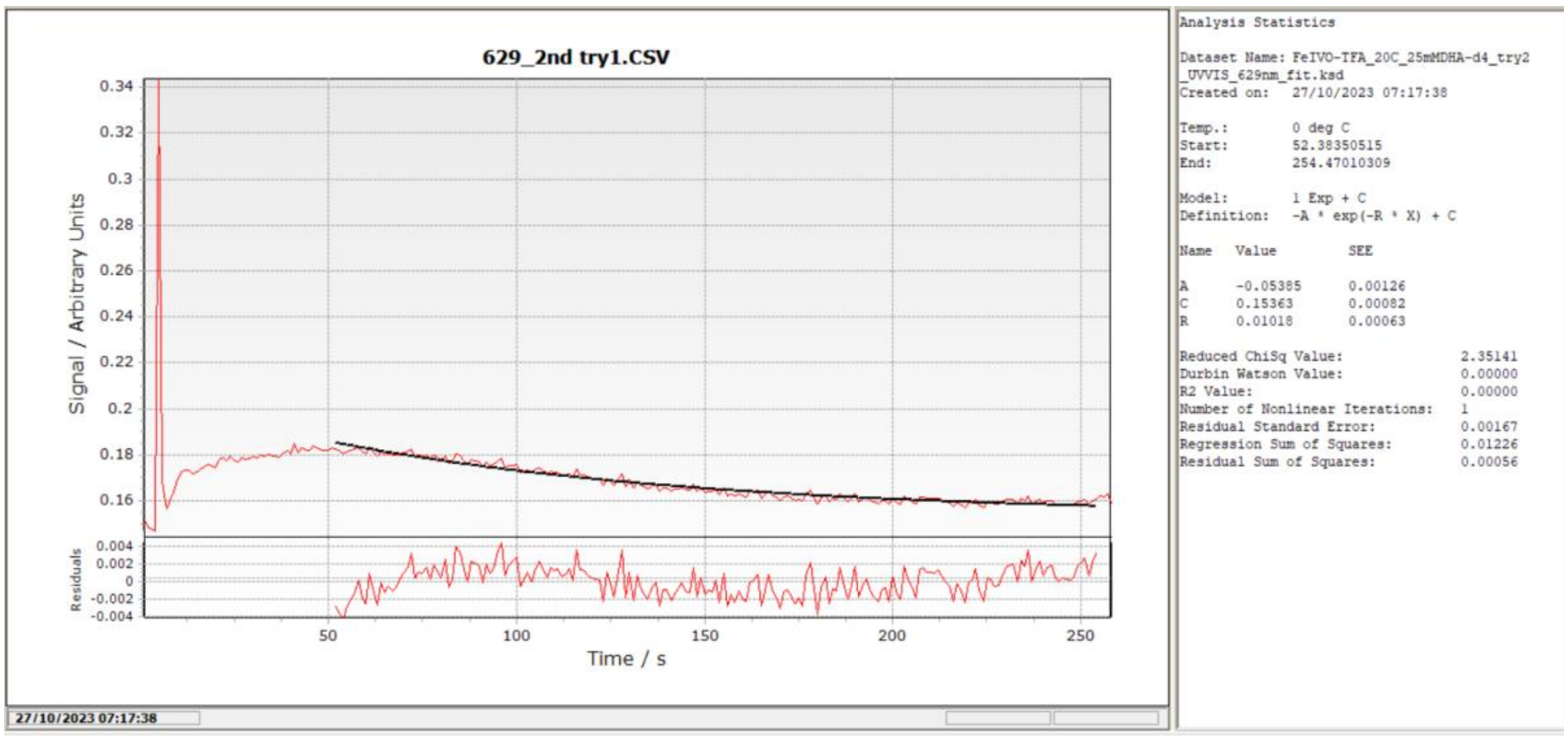

Figure S118. Exemplary trace (629 nm) and fit for the UV-Vis measurement of the reaction of **4** (1 mM) with 25 mM DHA-d_4 at 20 °C in a mixture of MeCN and DCM.

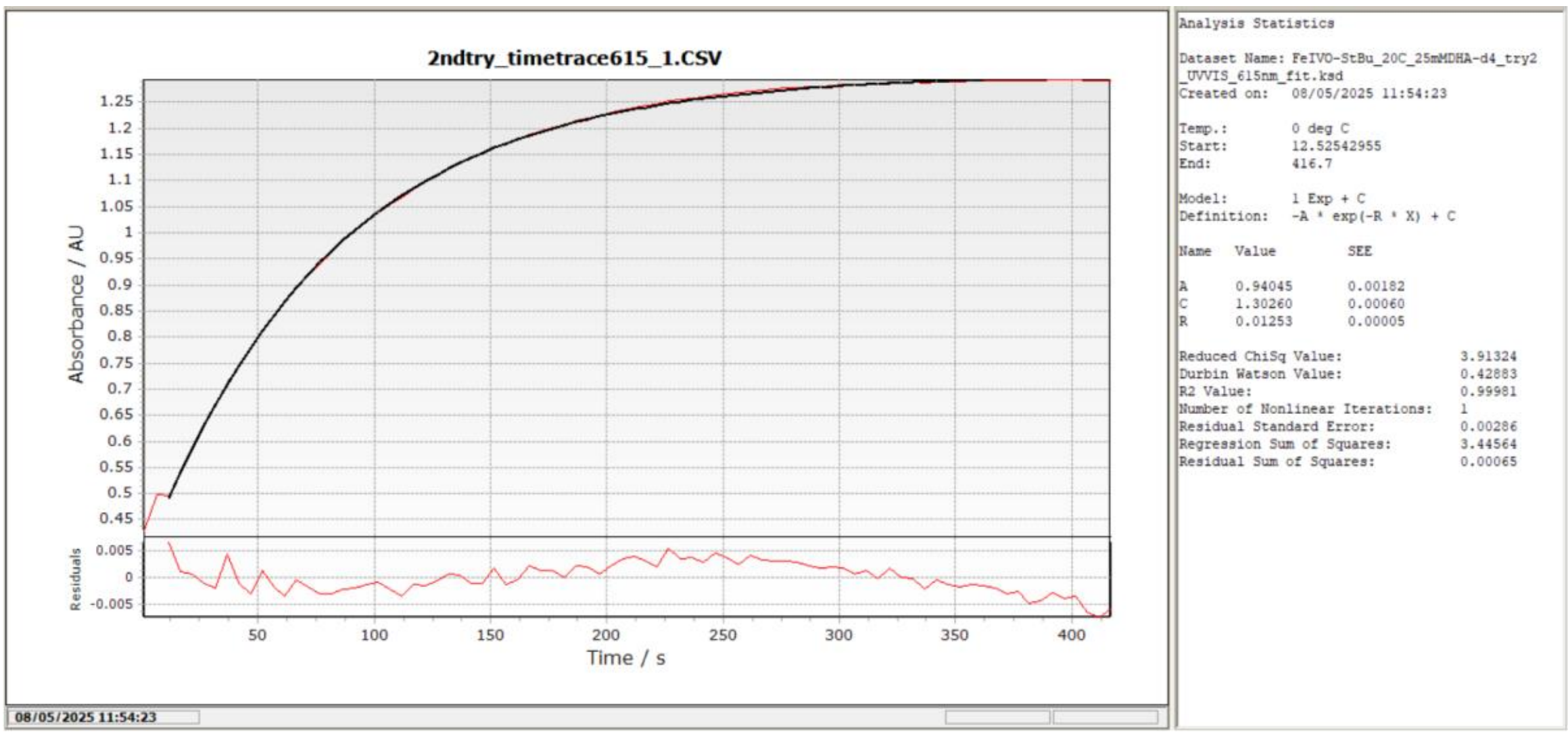

Figure S119. Exemplary trace (615 nm) and fit for the UV-Vis measurement of the reaction of **5a** (1 mM) with 25 mM DHA-d_4 at 20 °C in a mixture of MeCN and DCM.

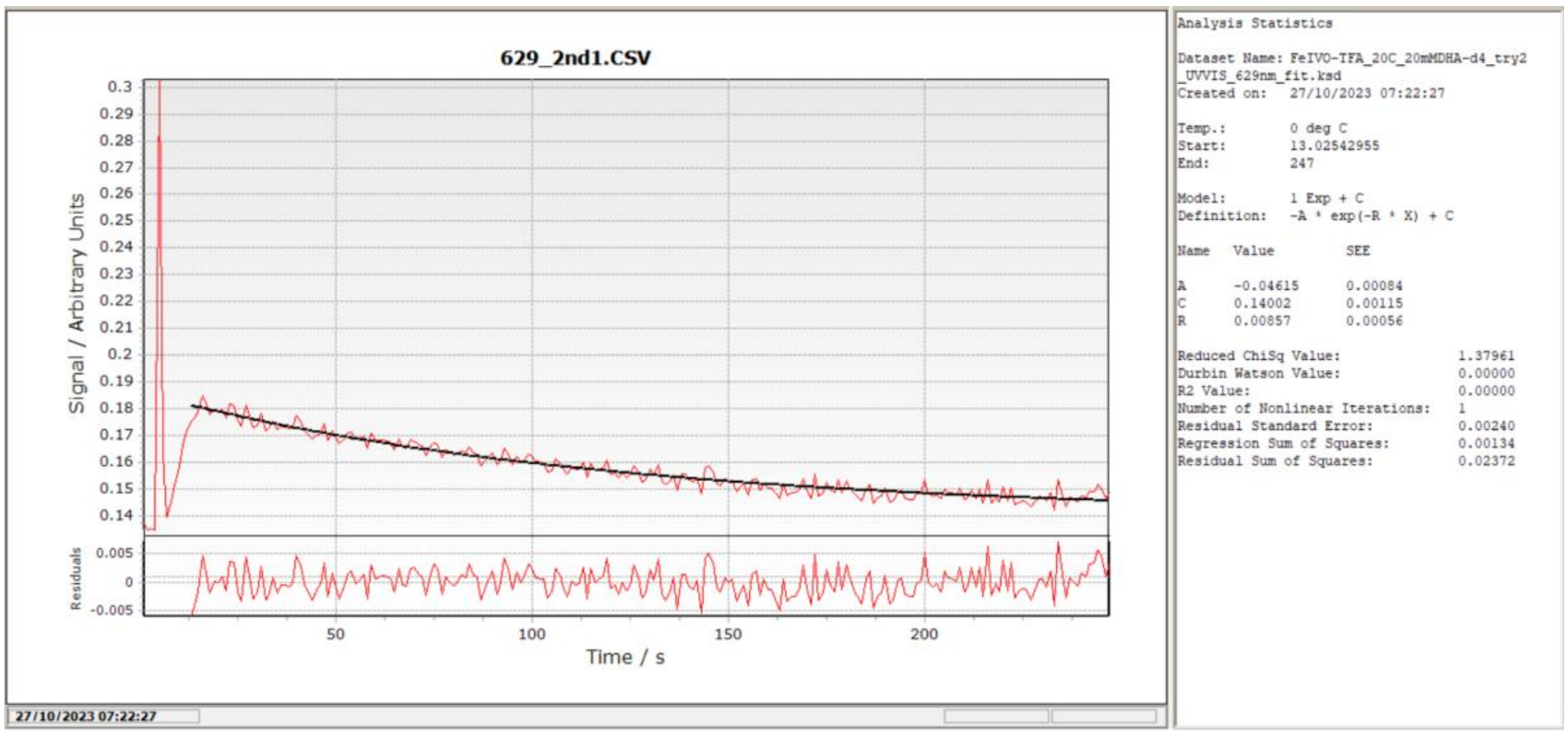

Figure S120. Exemplary trace (629 nm) and fit for the UV-Vis measurement of the reaction of **4** (1 mM) with 20 mM DHA-d_4 at 20 °C in a mixture of MeCN and DCM.

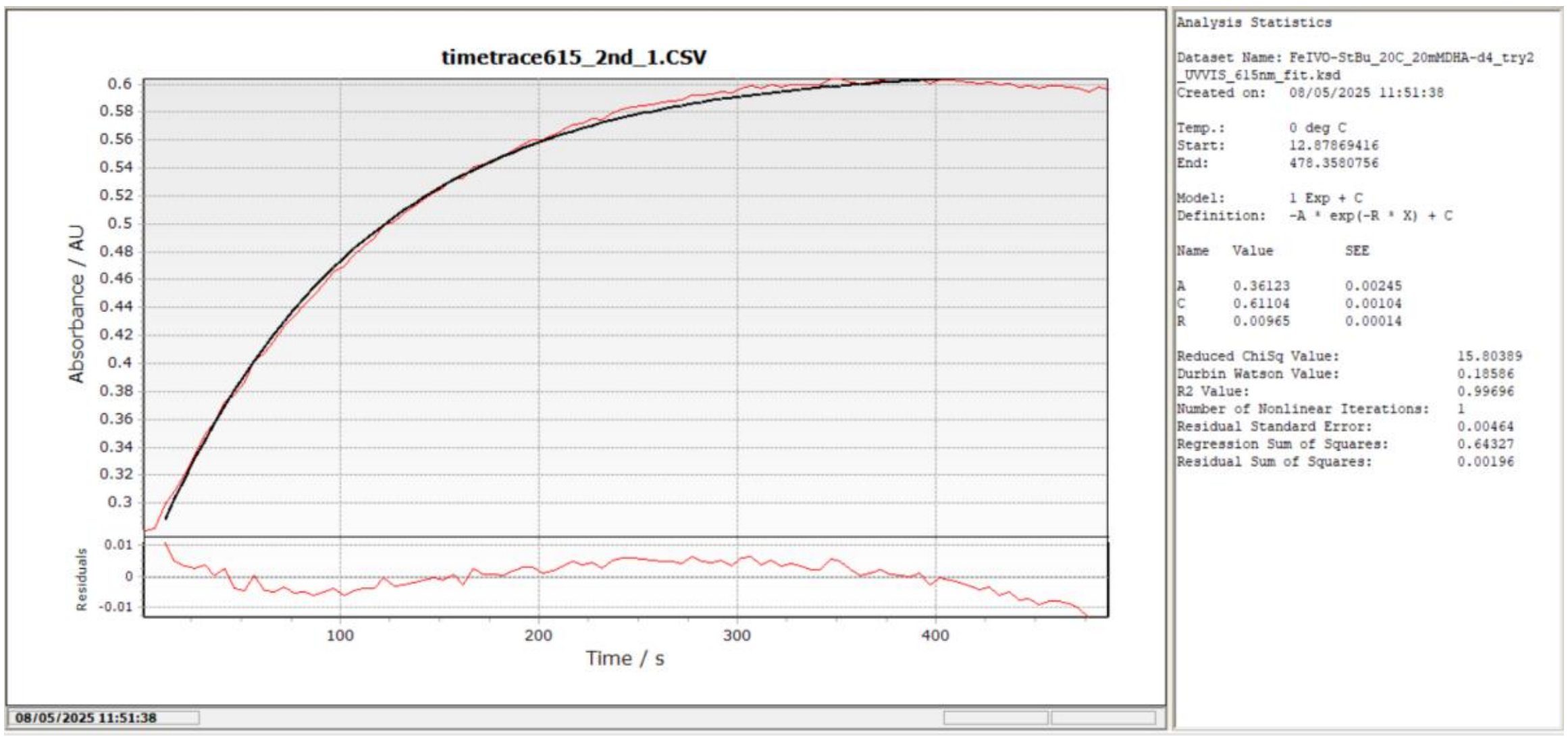

Figure S121. Exemplary trace (615 nm) and fit for the UV-Vis measurement of the reaction of **5a** (1 mM) with 20 mM DHA-d_4 at 20 °C in a mixture of MeCN and DCM.

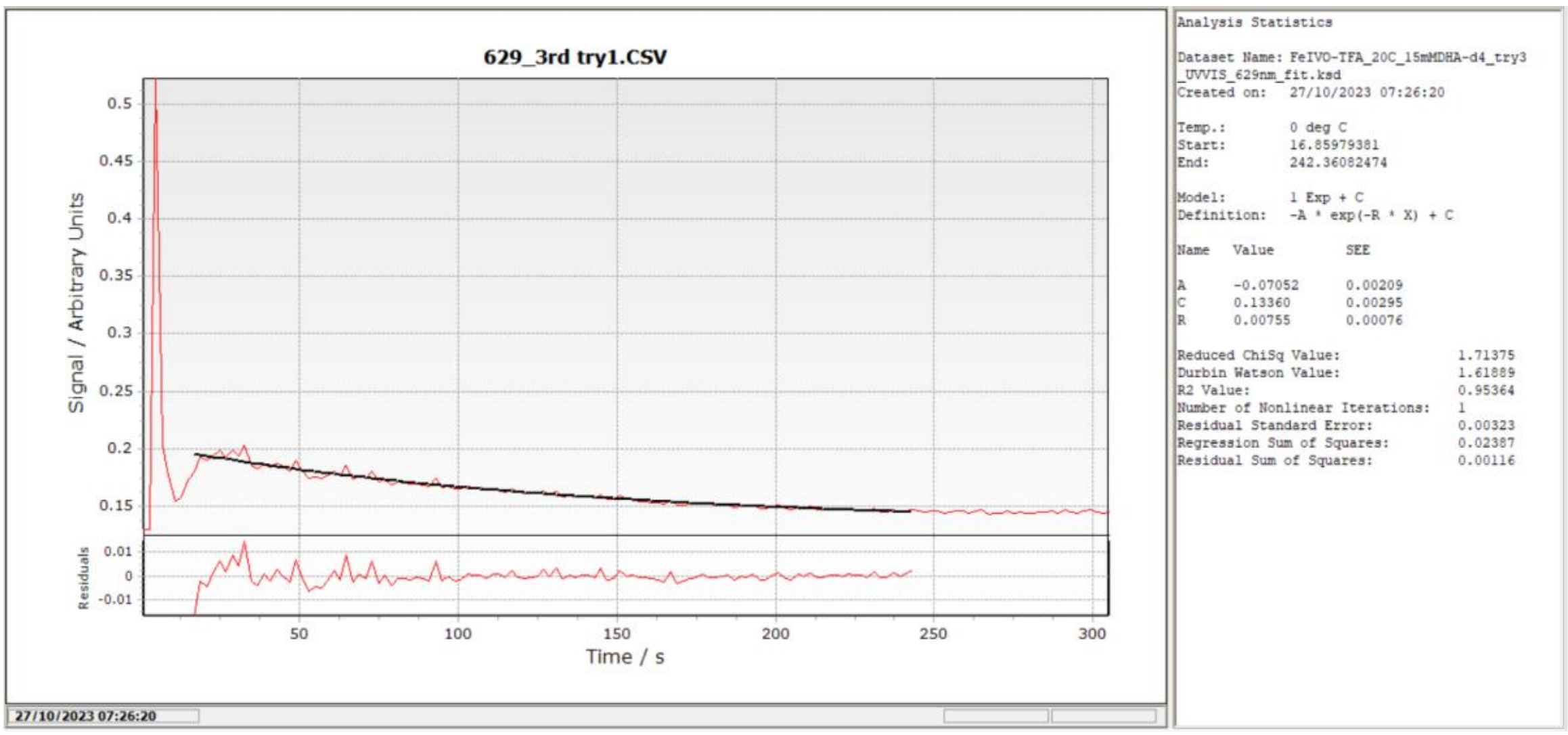

Figure S122. Exemplary trace (629 nm) and fit for the UV-Vis measurement of the reaction of **4** (1 mM) with 15 mM DHA-d_4 at 20 °C in a mixture of MeCN and DCM.

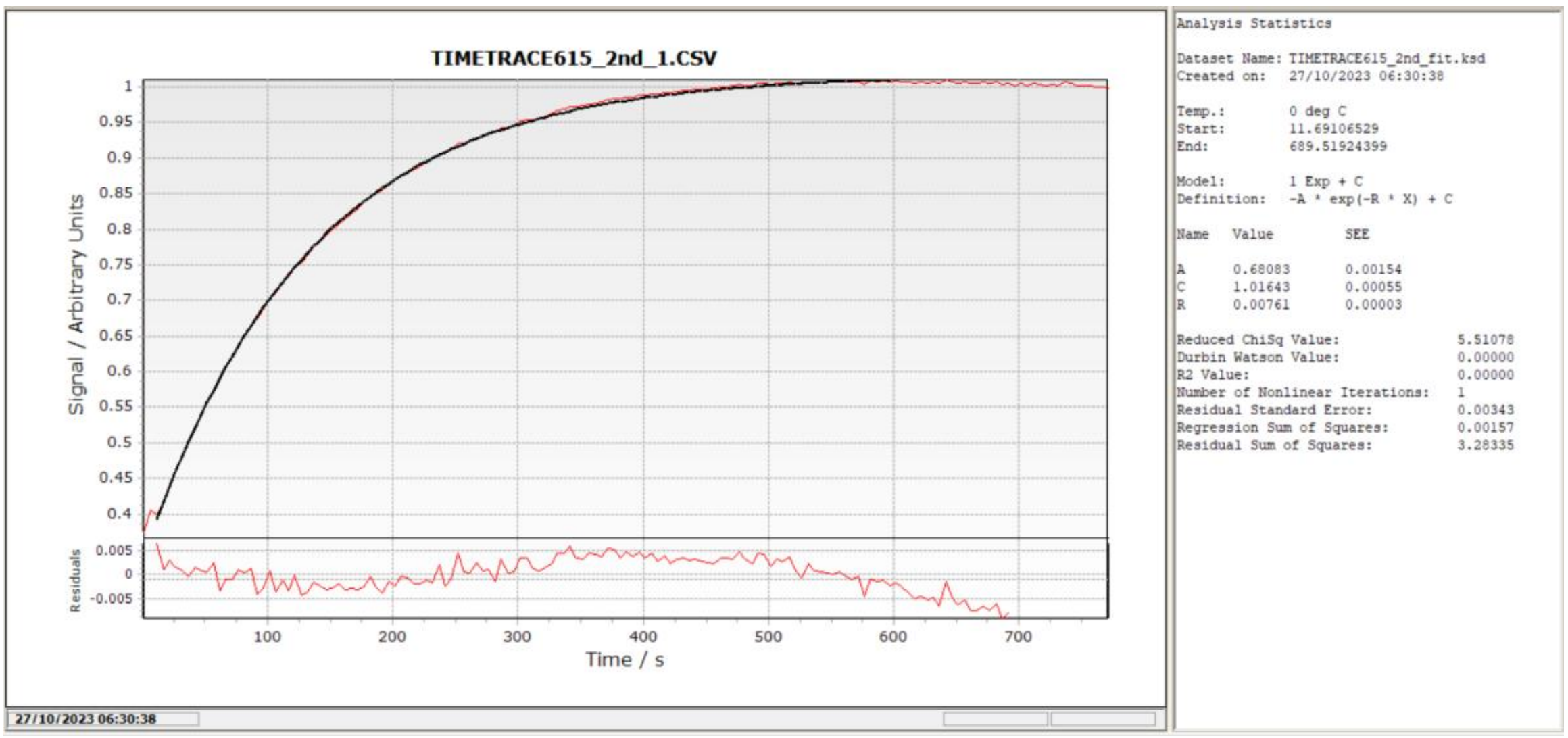

Figure S123. Exemplary trace (615 nm) and fit for the UV-Vis measurement of the reaction of **5a** (1 mM) with 15 mM DHA-d_4 at 20 °C in a mixture of MeCN and DCM.

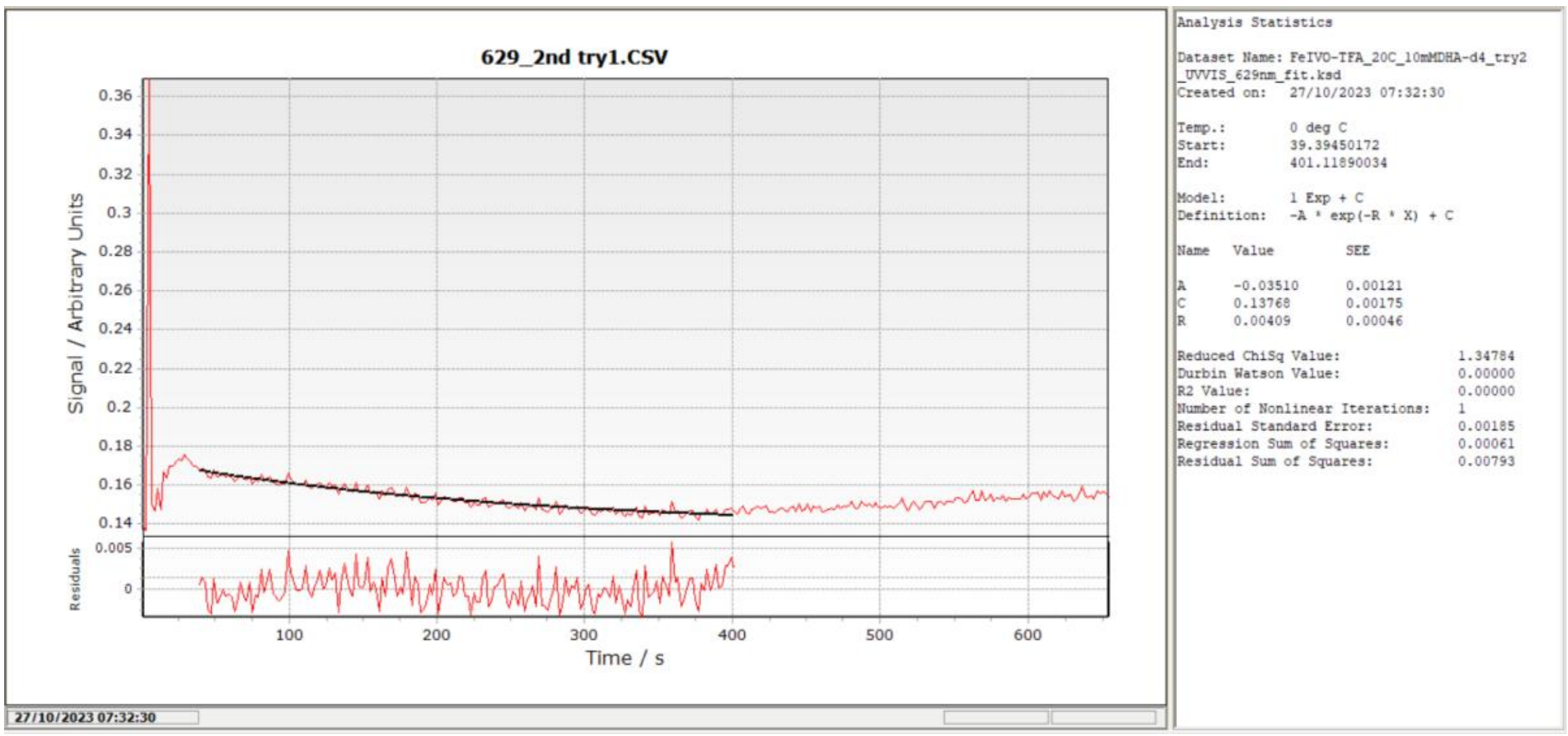

Figure S124. Exemplary trace (629 nm) and fit for the UV-Vis measurement of the reaction of **4** (1 mM) with 10 mM DHA-d_4 at 20 °C in a mixture of MeCN and DCM.

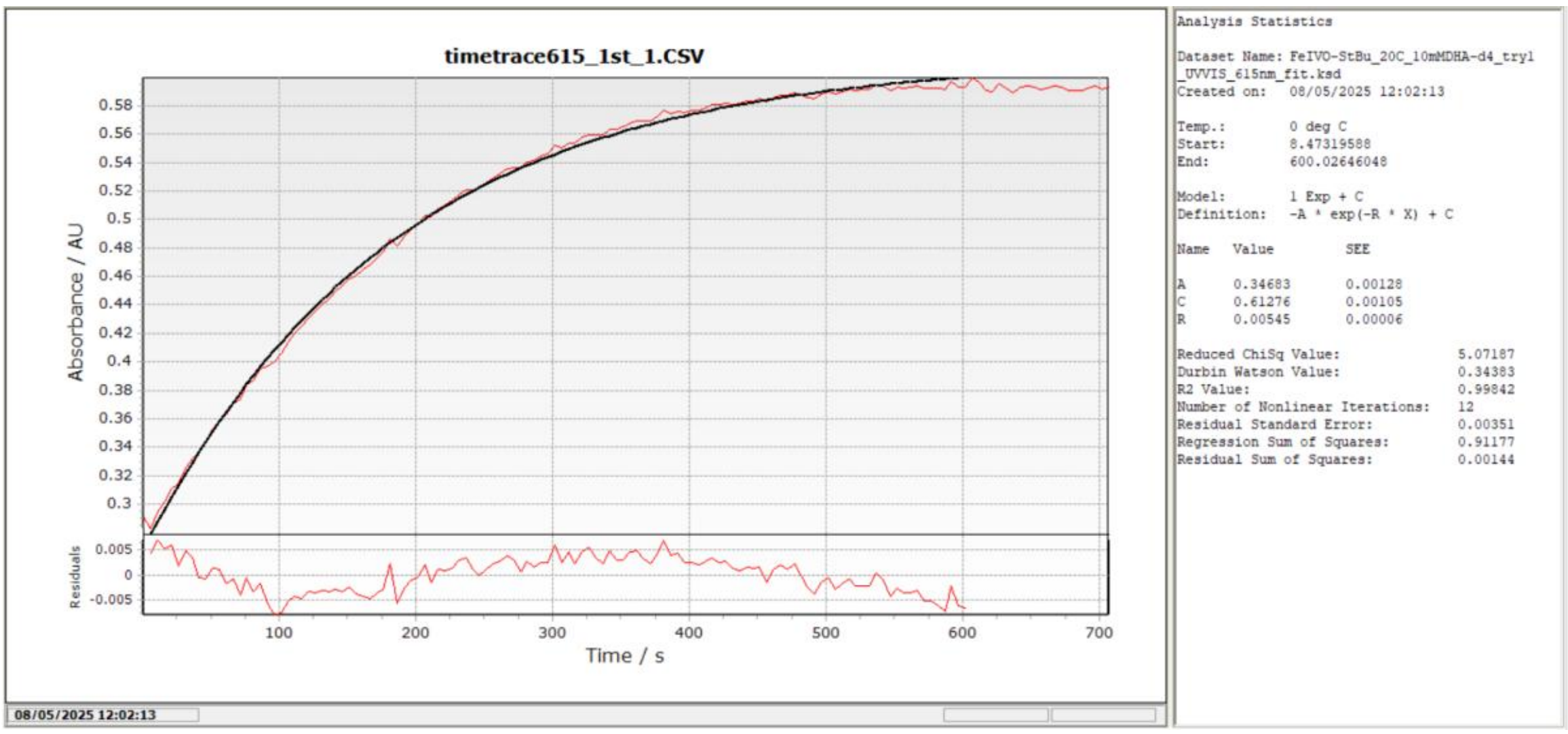

Figure S125. Exemplary trace (615 nm) and fit for the UV-Vis measurement of the reaction of **5a** (1 mM) with 10 mM DHA-d_4 at 20 °C in a mixture of MeCN and DCM.

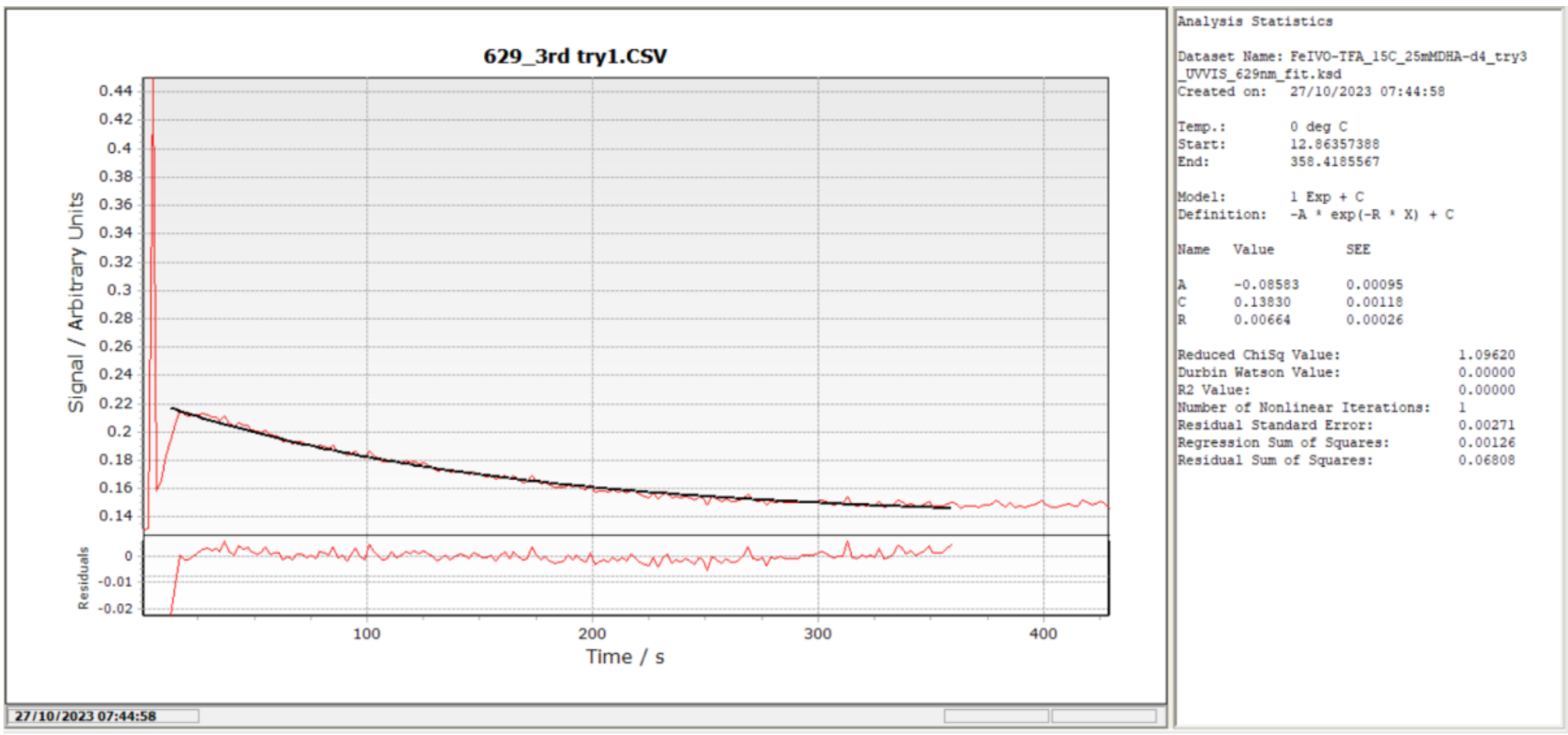

Figure S126. Exemplary trace (629 nm) and fit for the UV-Vis measurement of the reaction of **4** (1 mM) with 25 mM DHA-d_4 at 15 °C in a mixture of MeCN and DCM.

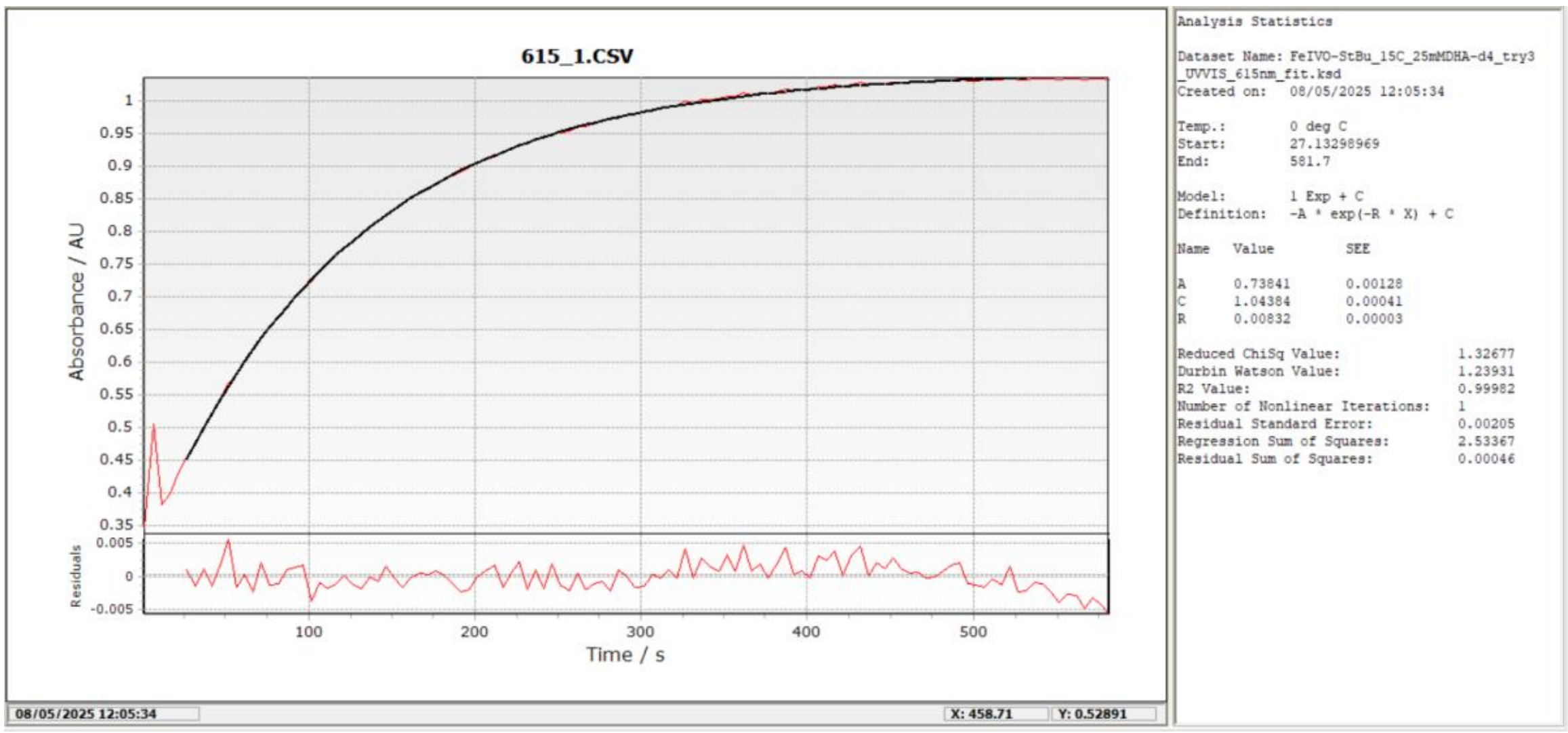

Figure S127. Exemplary trace (615 nm) and fit for the UV-Vis measurement of the reaction of **5a** (1 mM) with 25 mM DHA-d_4 at 15 °C in a mixture of MeCN and DCM.

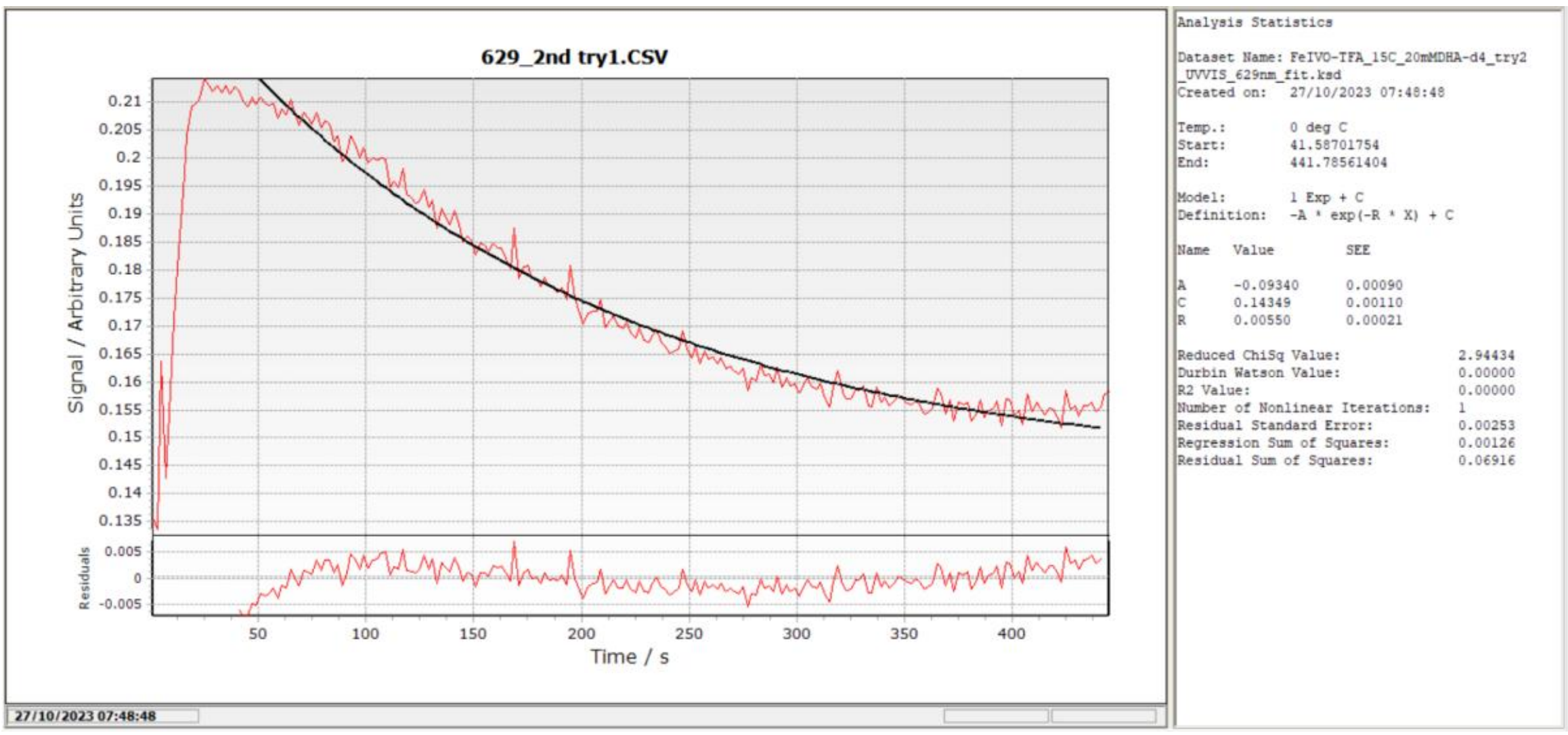

Figure S128. Exemplary trace (629 nm) and fit for the UV-Vis measurement of the reaction of **4** (1 mM) with 20 mM DHA-d_4 at 15 °C in a mixture of MeCN and DCM.

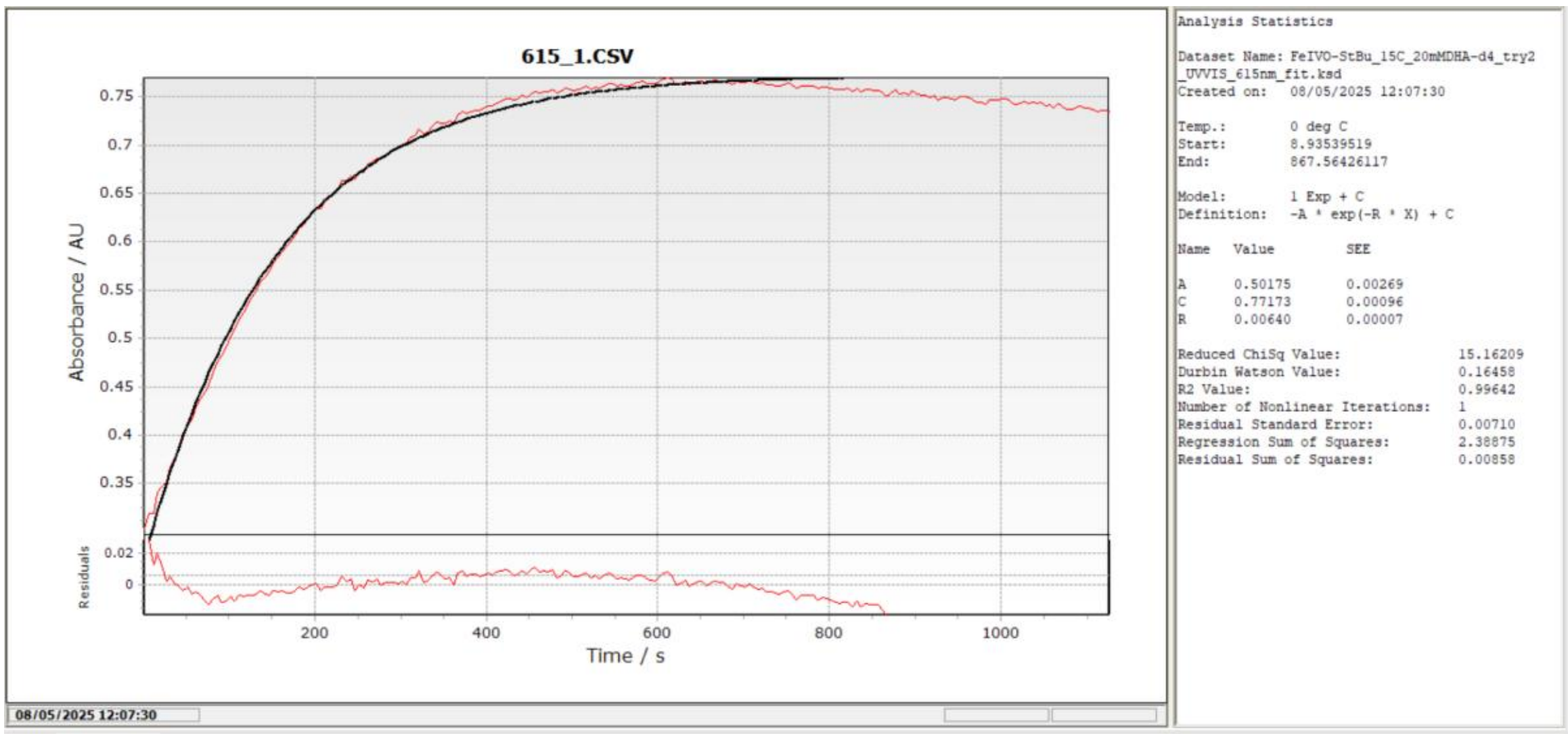

Figure S129. Exemplary trace (615 nm) and fit for the UV-Vis measurement of the reaction of **5a** (1 mM) with 20 mM DHA-d_4 at 15 °C in a mixture of MeCN and DCM.

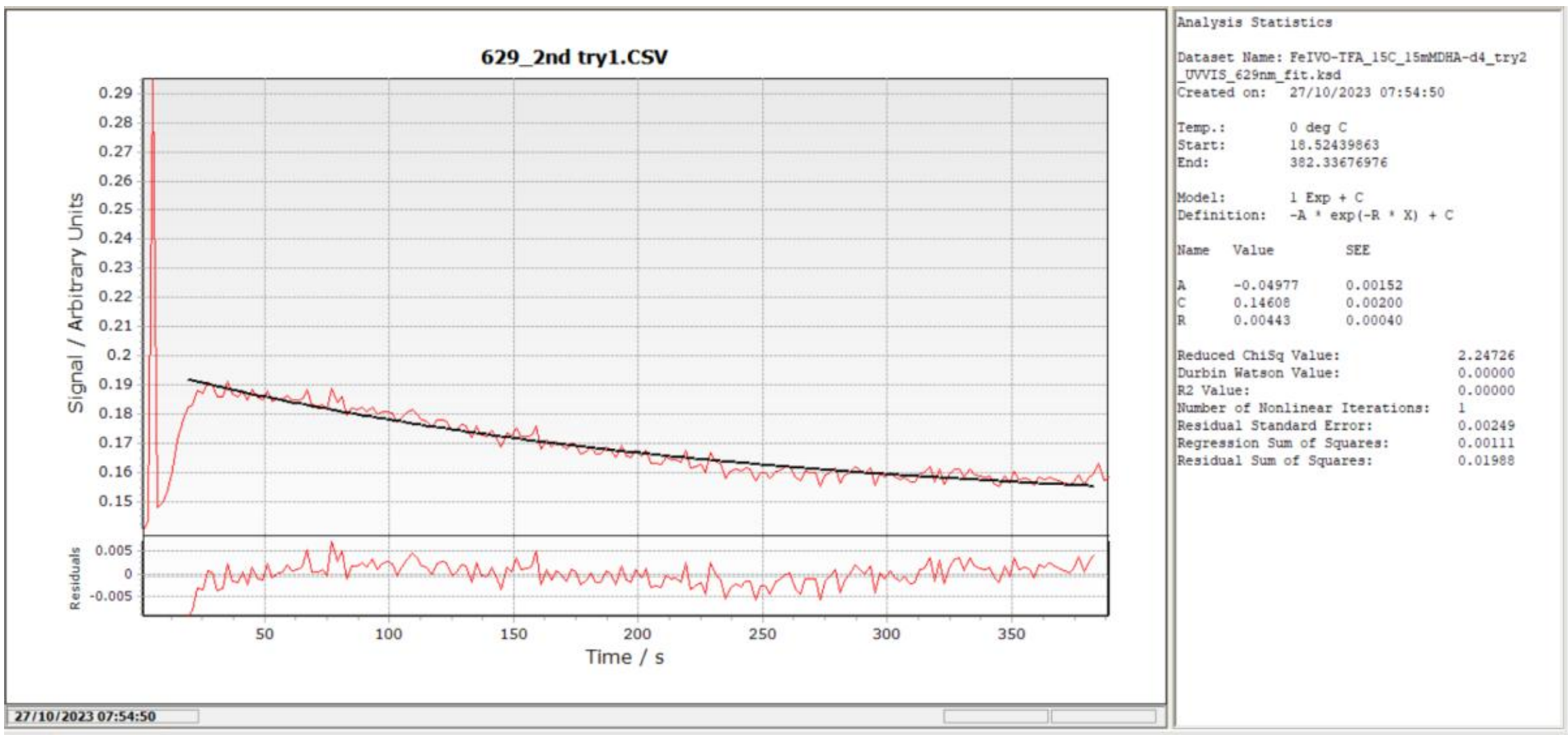

Figure S130. Exemplary trace (629 nm) and fit for the UV-Vis measurement of the reaction of **4** (1 mM) with 15 mM DHA-d_4 at 15 °C in a mixture of MeCN and DCM.

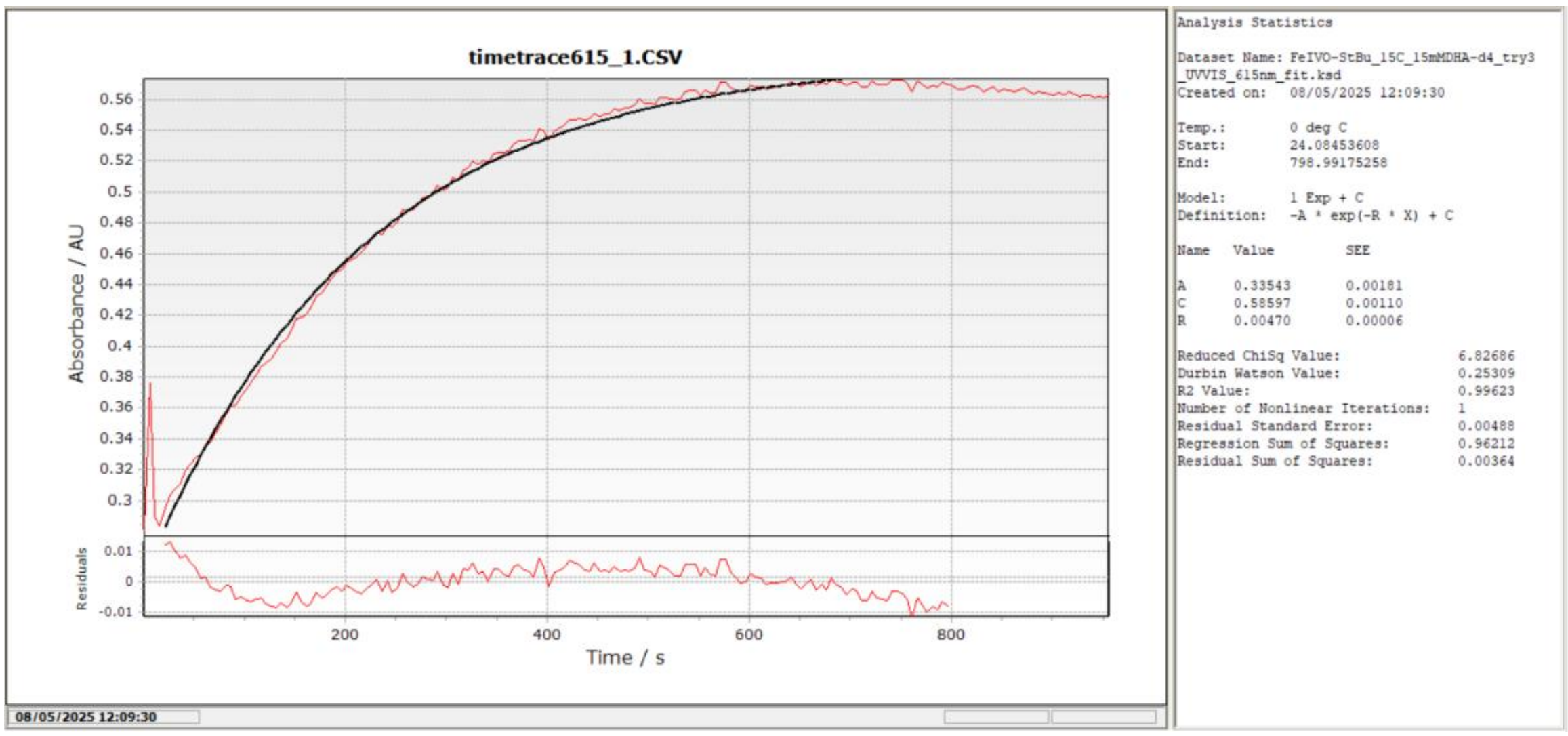

Figure S131. Exemplary trace (615 nm) and fit for the UV-Vis measurement of the reaction of **5a** (1 mM) with 15 mM DHA-d_4 at 15 °C in a mixture of MeCN and DCM.

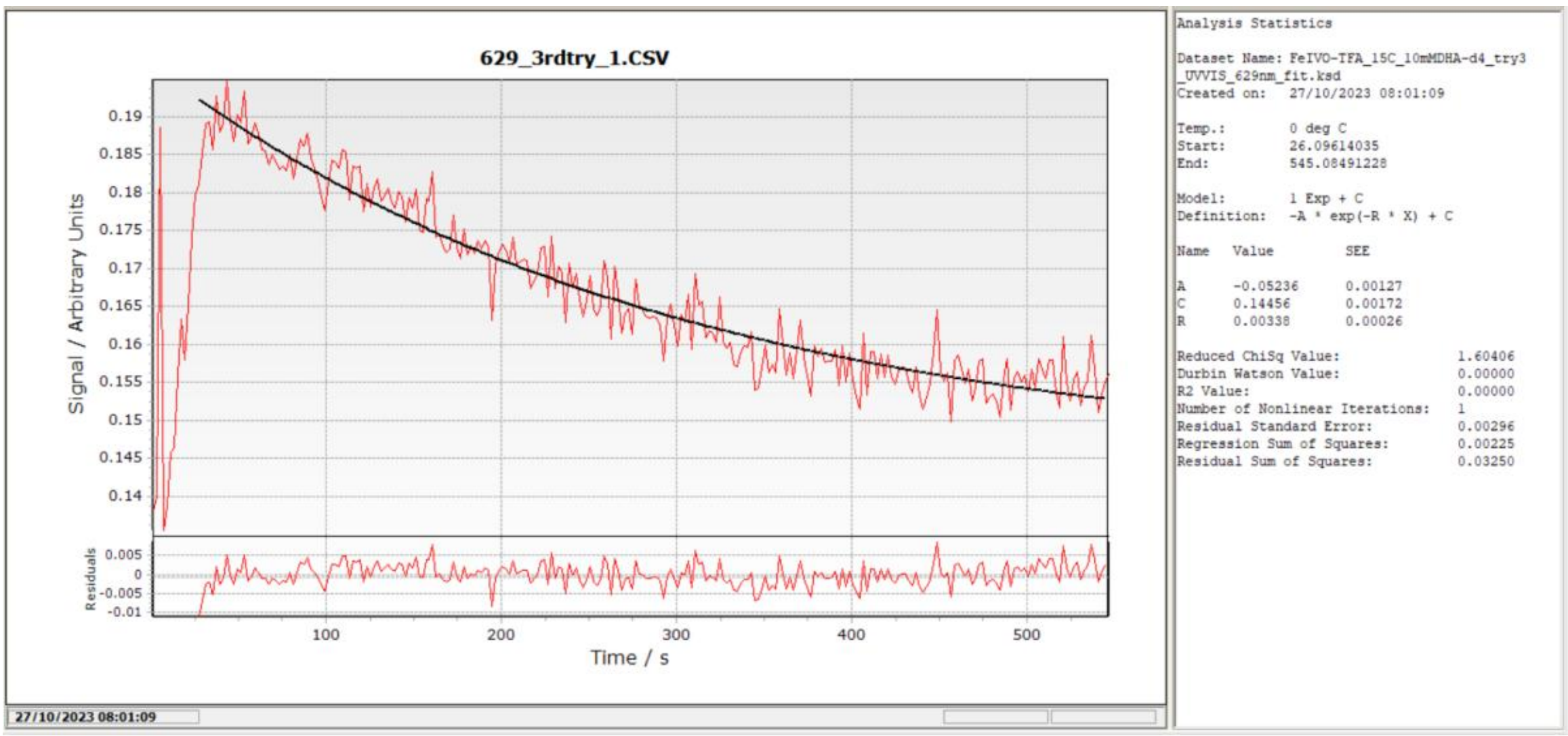

Figure S132. Exemplary trace (629 nm) and fit for the UV-Vis measurement of the reaction of **4** (1 mM) with 10 mM DHA-d_4 at 15 °C in a mixture of MeCN and DCM.

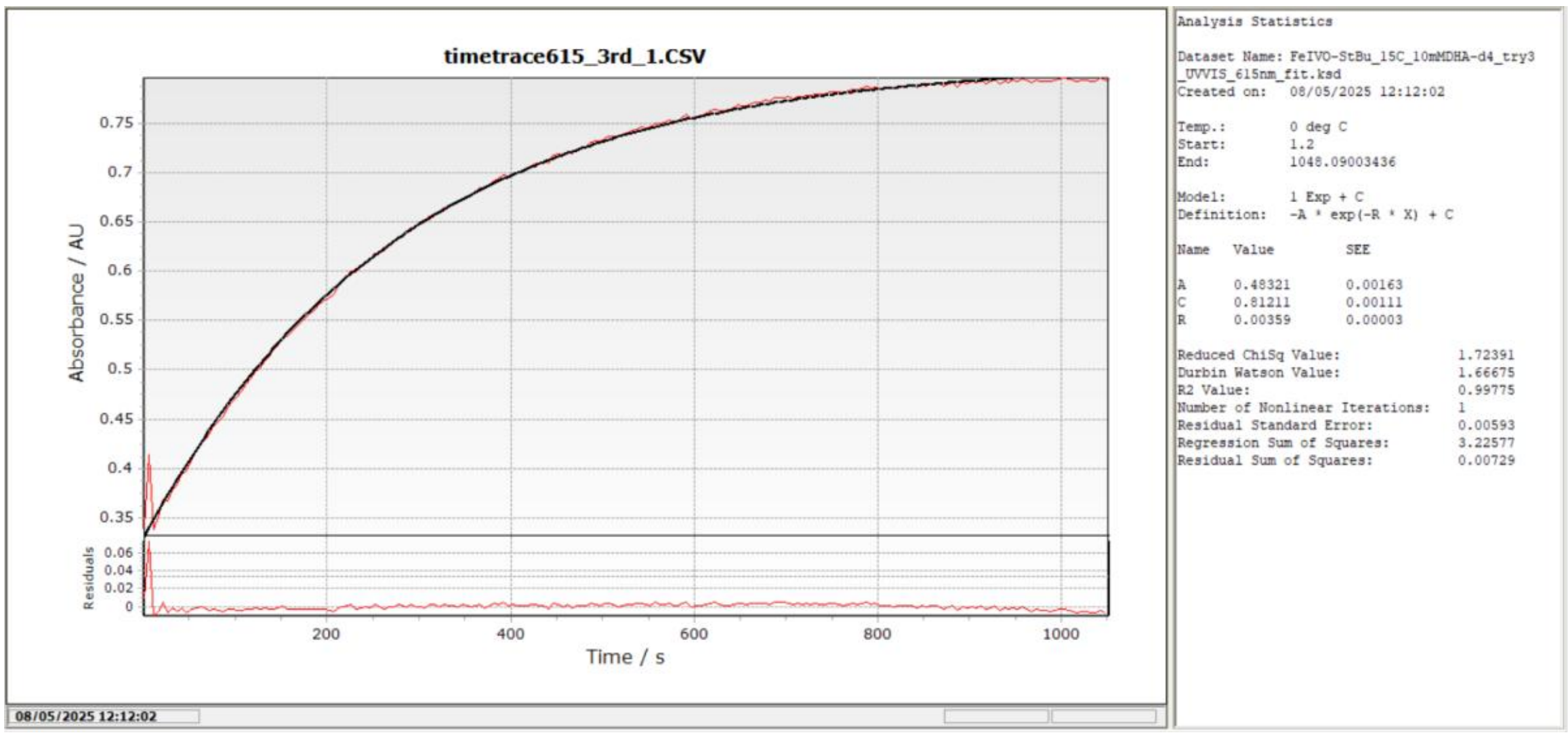

Figure S133. Exemplary trace (615 nm) and fit for the UV-Vis measurement of the reaction of **5a** (1 mM) with 10 mM DHA-d_4 at 15 °C in a mixture of MeCN and DCM.

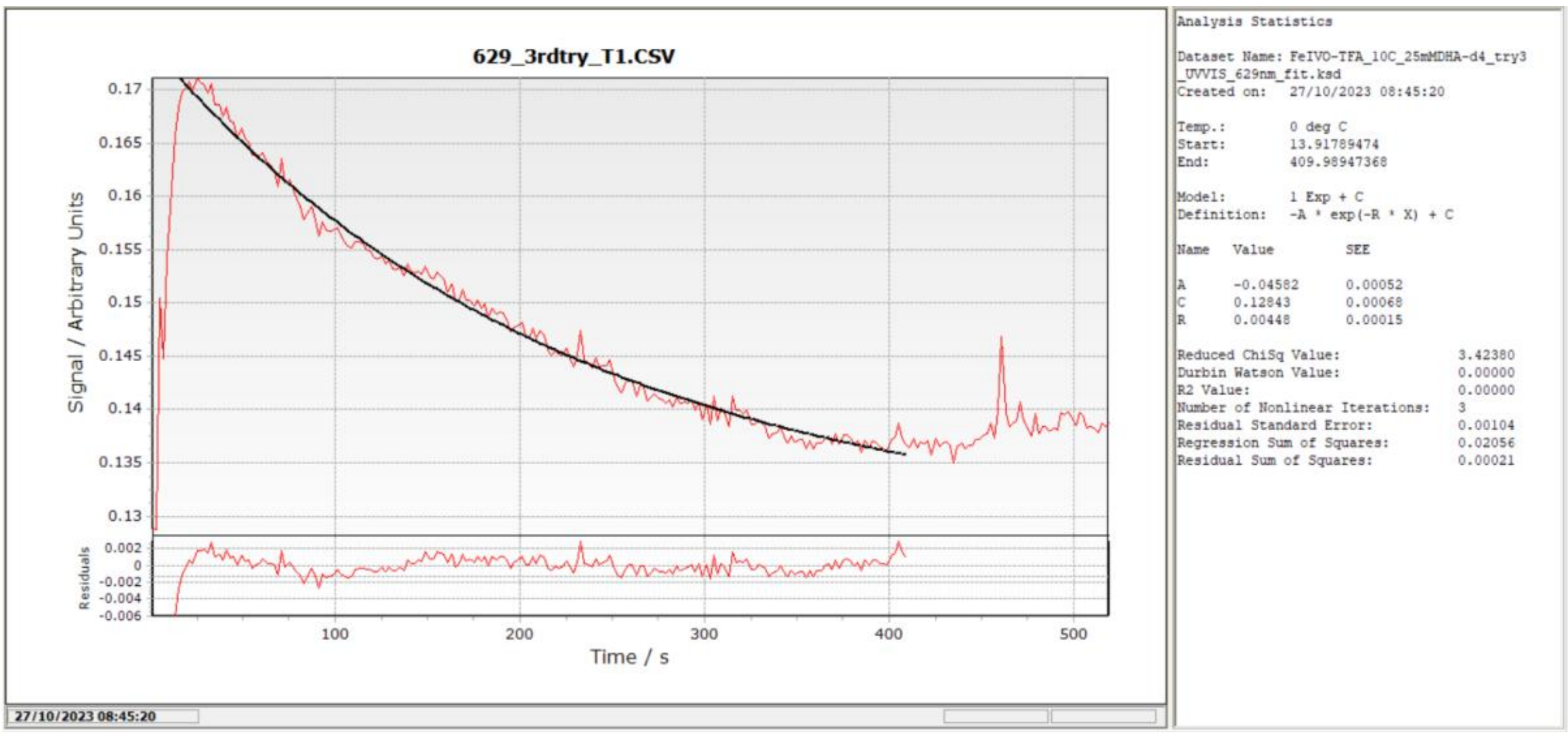

Figure S134. Exemplary trace (629 nm) and fit for the UV-Vis measurement of the reaction of **4** (1 mM) with 25 mM DHA-d_4 at 10 °C in a mixture of MeCN and DCM.

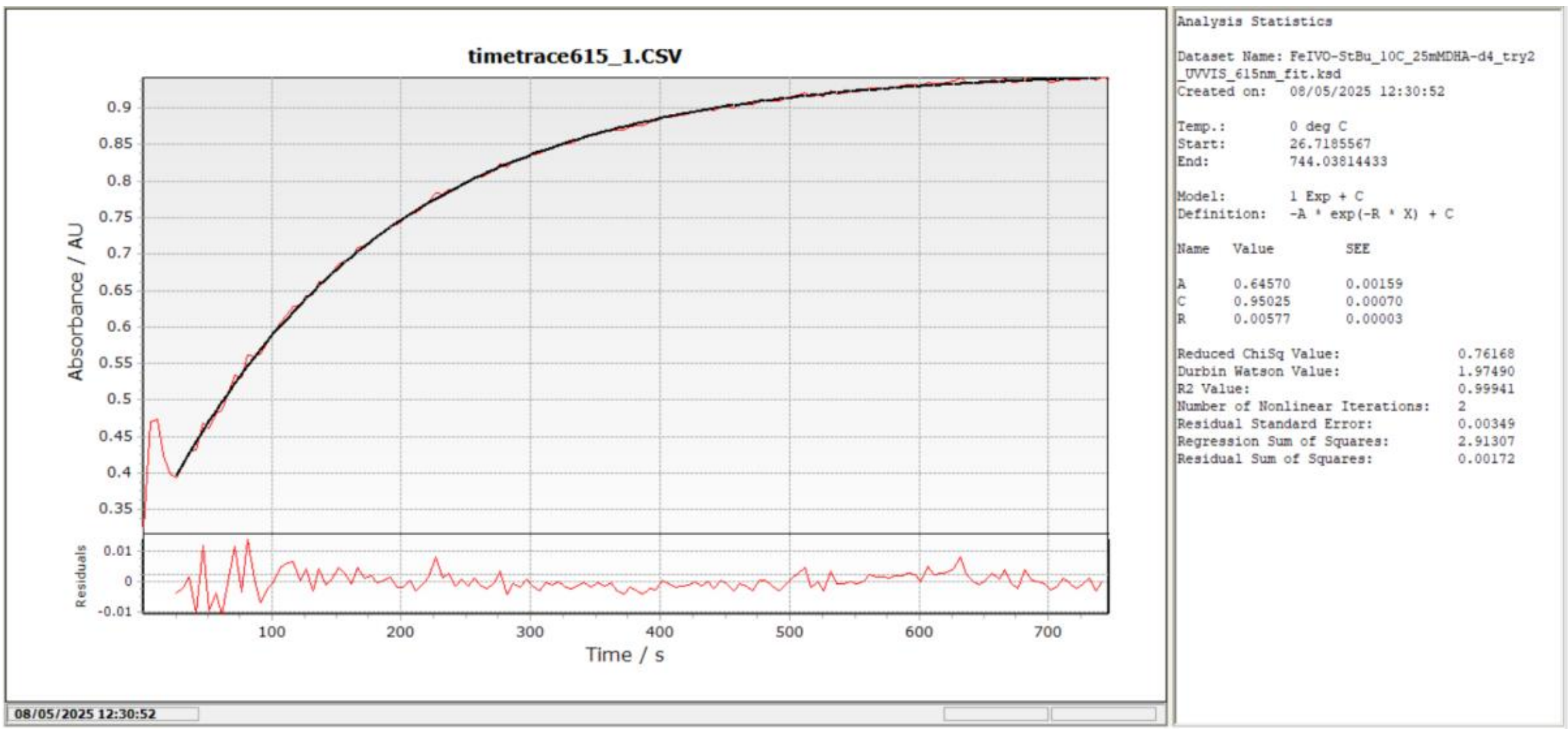

Figure S135. Exemplary trace (615 nm) and fit for the UV-Vis measurement of the reaction of **5a** (1 mM) with 25 mM DHA-d_4 at 10 °C in a mixture of MeCN and DCM.

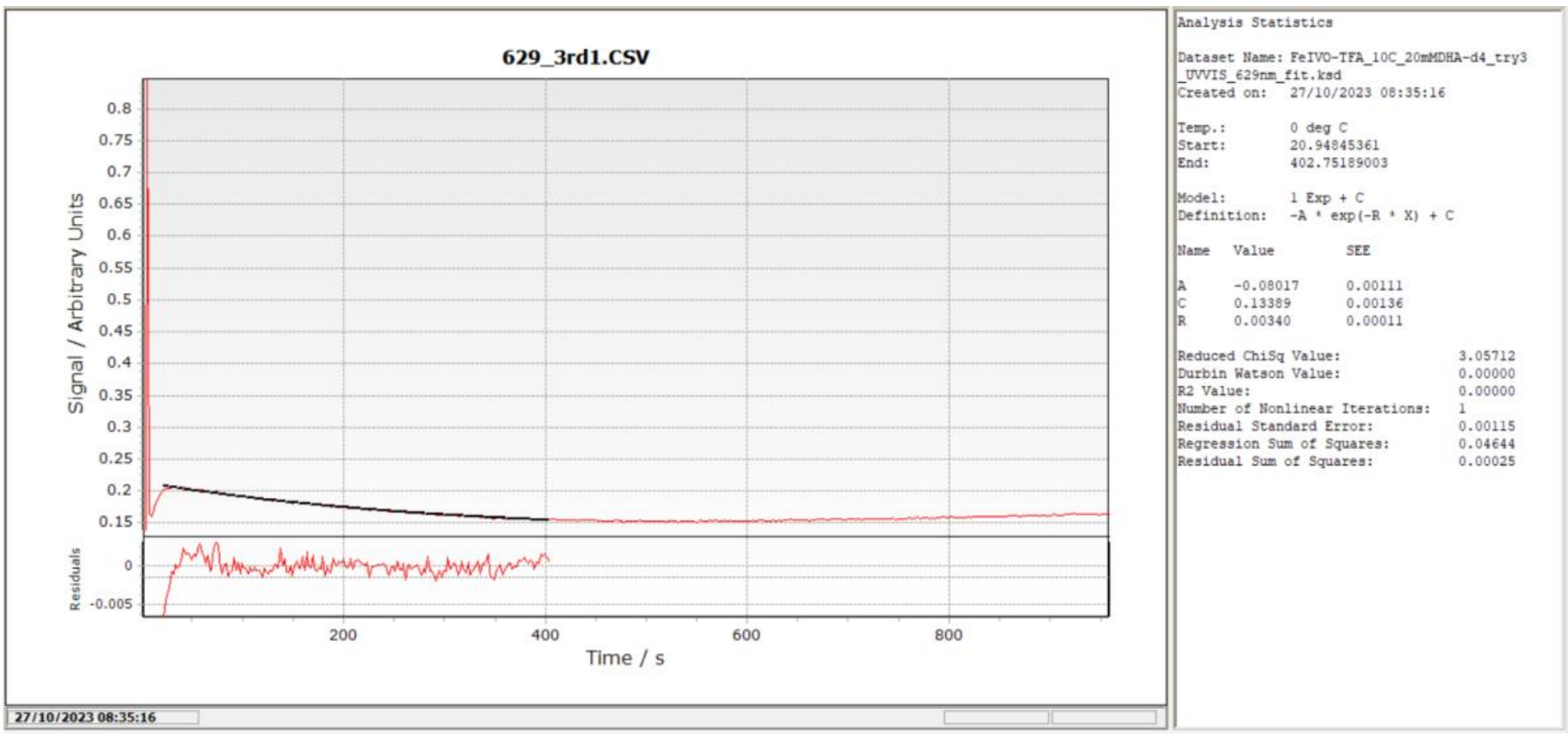

Figure S136. Exemplary trace (629 nm) and fit for the UV-Vis measurement of the reaction of **4** (1 mM) with 20 mM DHA-d_4 at 10 °C in a mixture of MeCN and DCM.

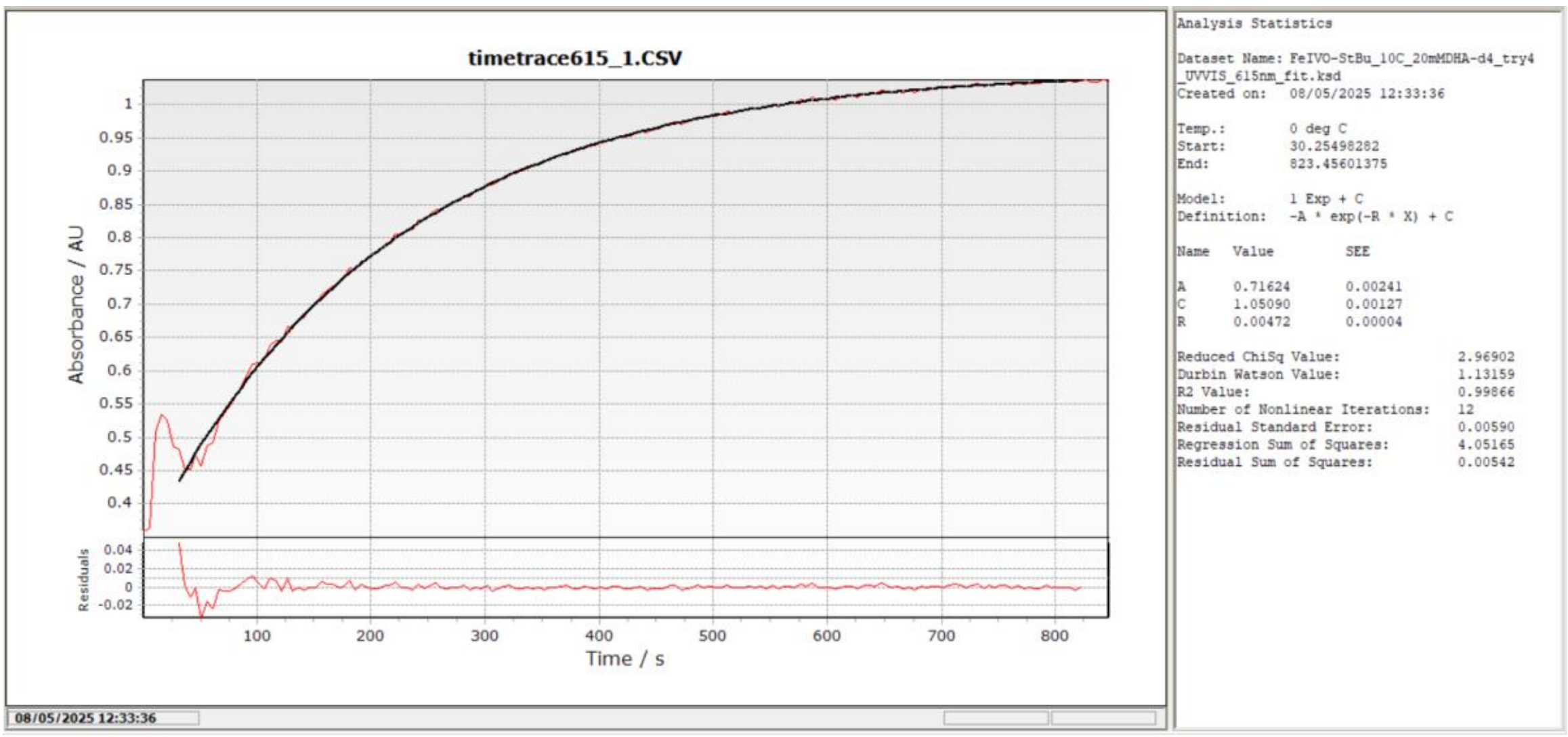

Figure S137. Exemplary trace (615 nm) and fit for the UV-Vis measurement of the reaction of **5a** (1 mM) with 20 mM DHA-d_4 at 10 °C in a mixture of MeCN and DCM.

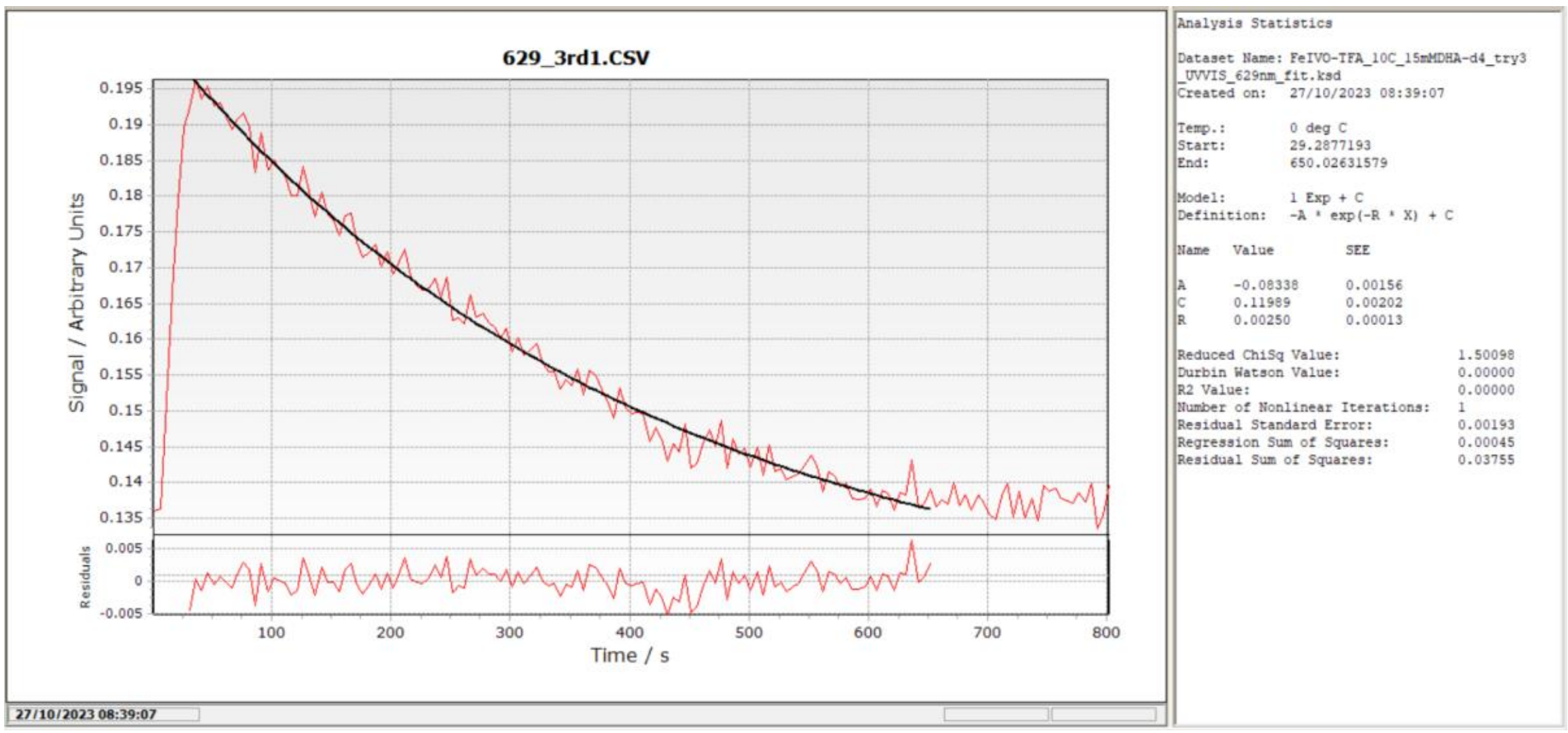

Figure S138. Exemplary trace (629 nm) and fit for the UV-Vis measurement of the reaction of **4** (1 mM) with 15 mM DHA-d_4 at 10 °C in a mixture of MeCN and DCM.

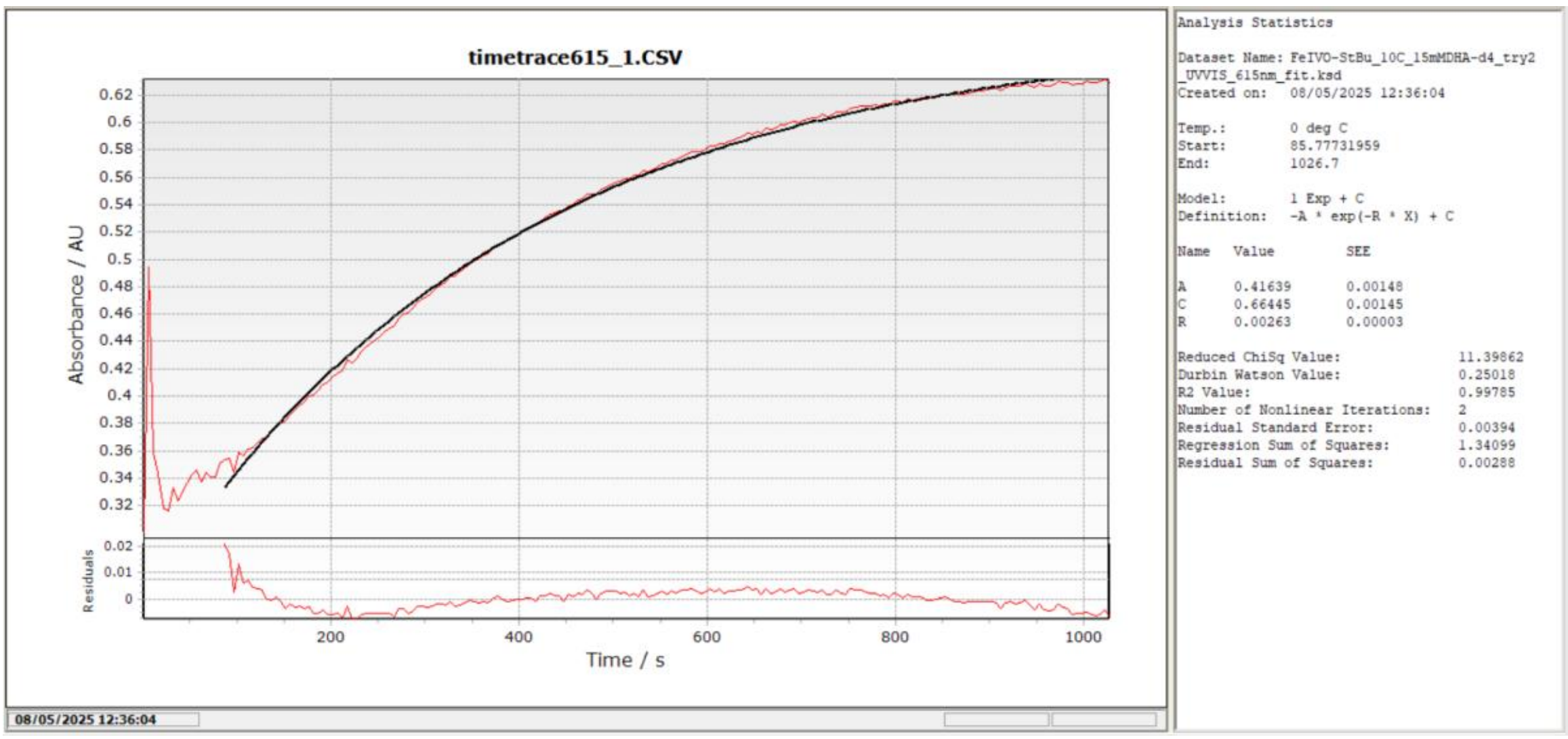

Figure S139. Exemplary trace (615 nm) and fit for the UV-Vis measurement of the reaction of **5a** (1 mM) with 15 mM DHA-d_4 at 10 °C in a mixture of MeCN and DCM.

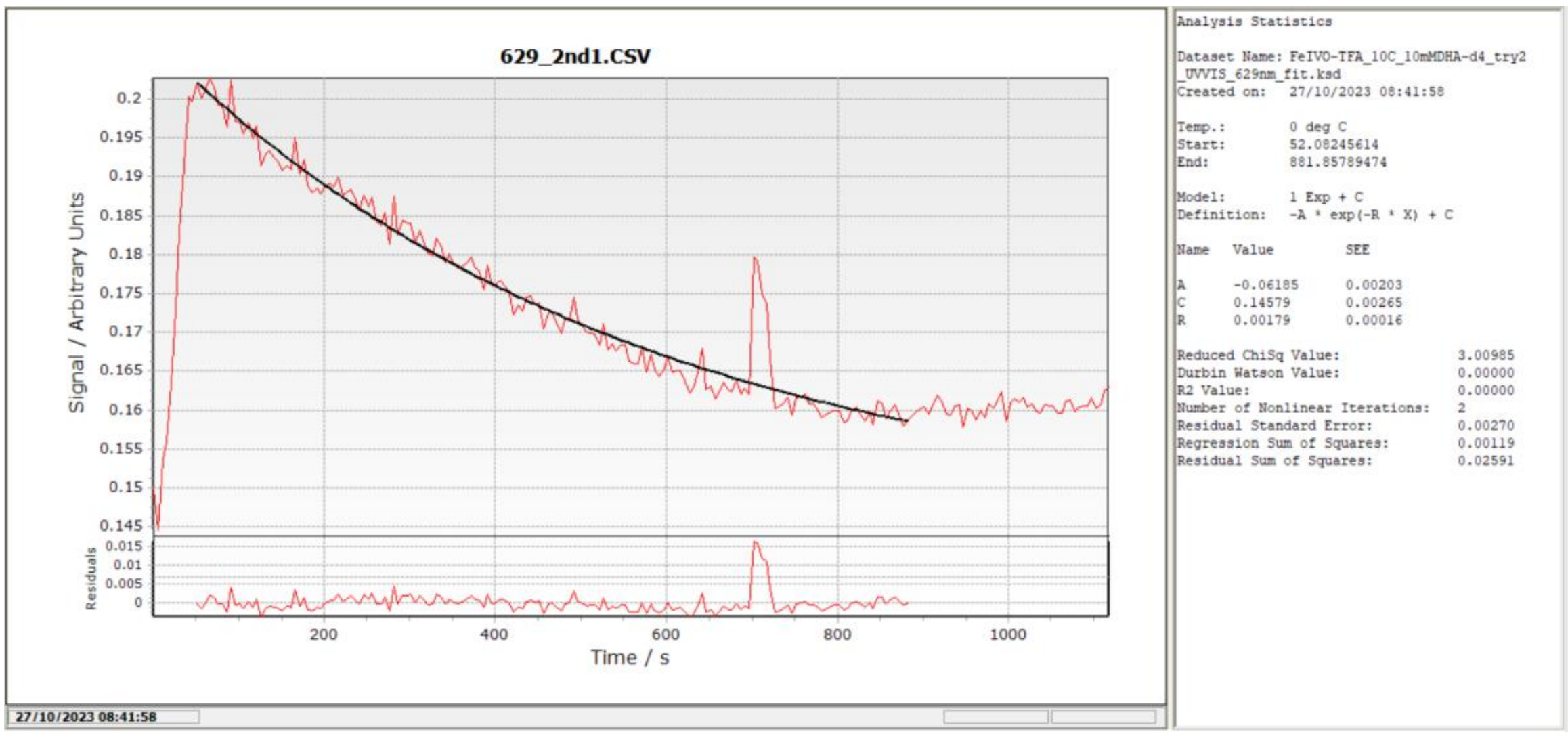

Figure S140. Exemplary trace (629 nm) and fit for the UV-Vis measurement of the reaction of **4** (1 mM) with 10 mM DHA-d_4 at 10 °C in a mixture of MeCN and DCM.

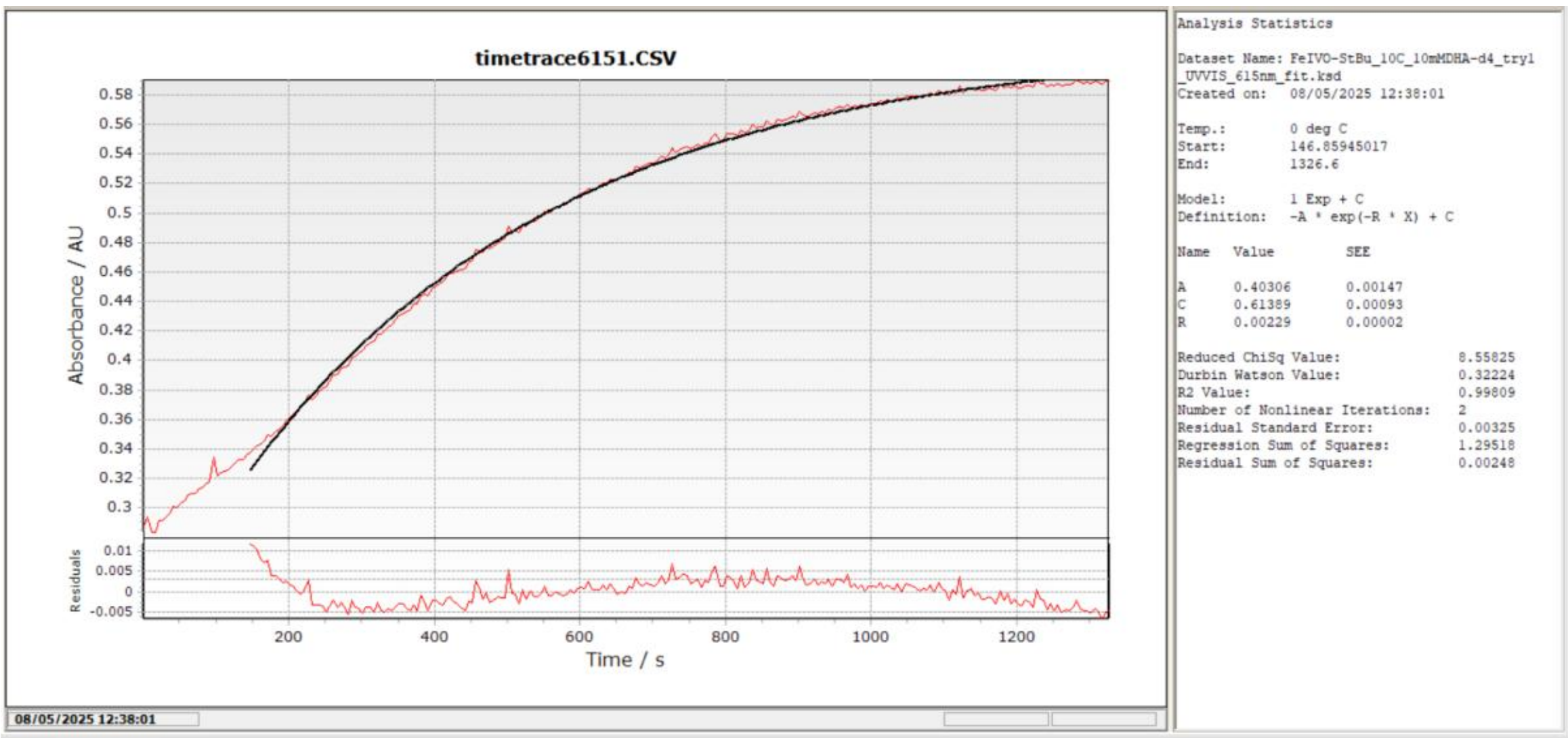

Figure S141. Exemplary trace (615 nm) and fit for the UV-Vis measurement of the reaction of **5a** (1 mM) with 10 mM DHA-d_4 at 10 °C in a mixture of MeCN and DCM.

Kinetic stopped-flow UV-Vis measurements with Xan:

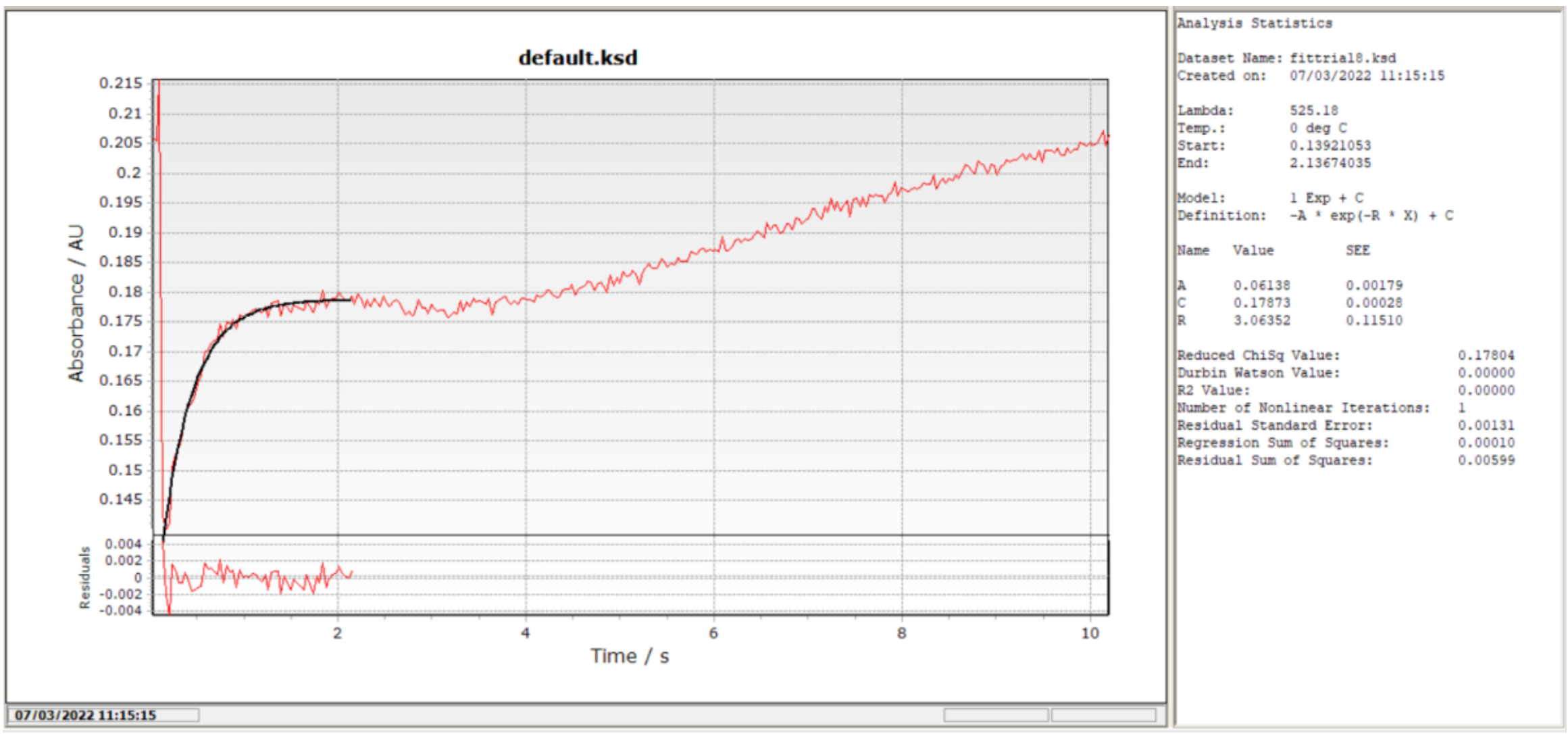

Figure S142. Exemplary trace (525 nm) and fit for the UV-Vis stopped-flow measurement of the reaction of **3** (1 mM) with 56.25 mM Xan at 25 °C in a mixture of MeCN and DCM.

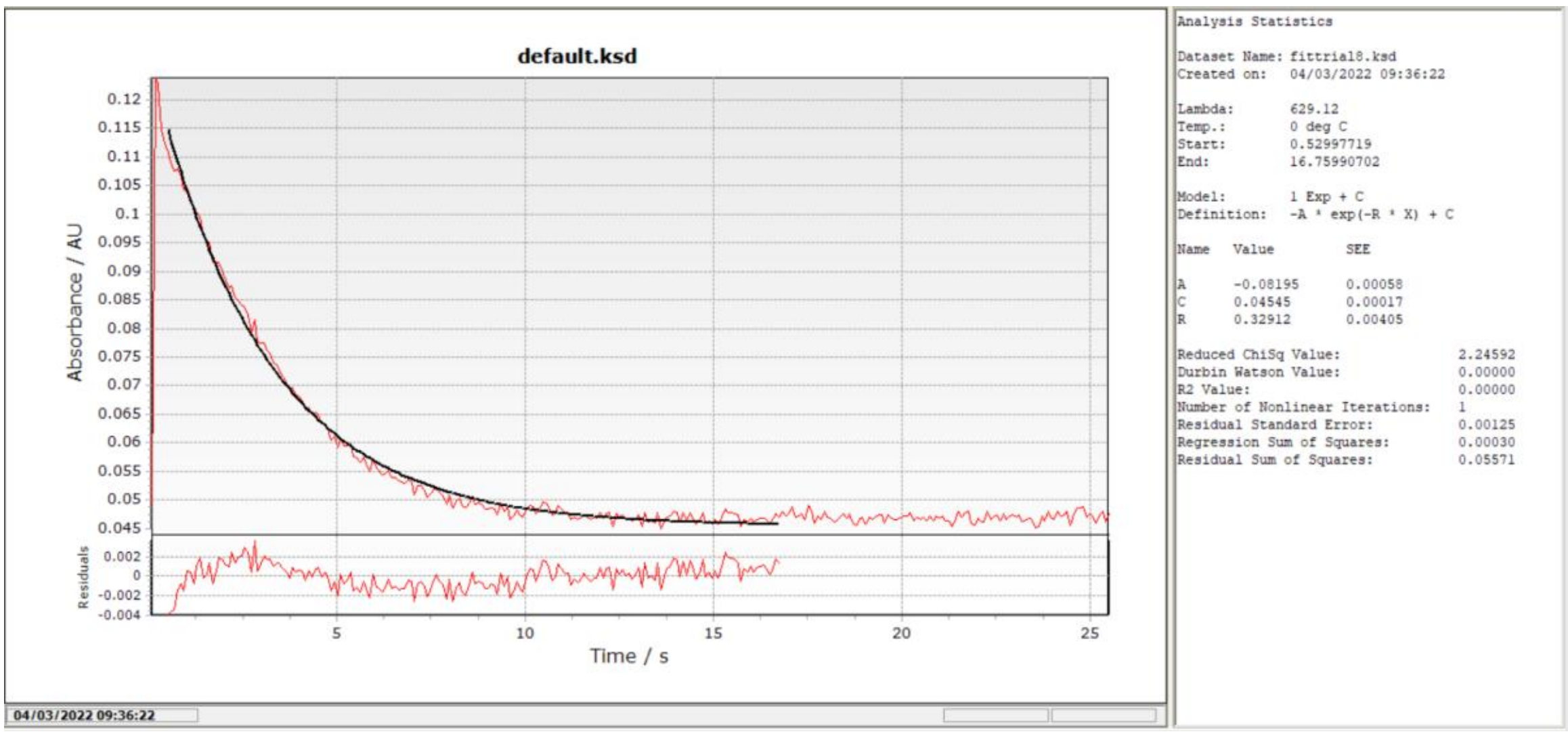

Figure S143. Exemplary trace (629 nm) and fit for the UV-Vis stopped-flow measurement of the reaction of **4** (1 mM) with 56.25 mM Xan at 25 °C in a mixture of MeCN and DCM.

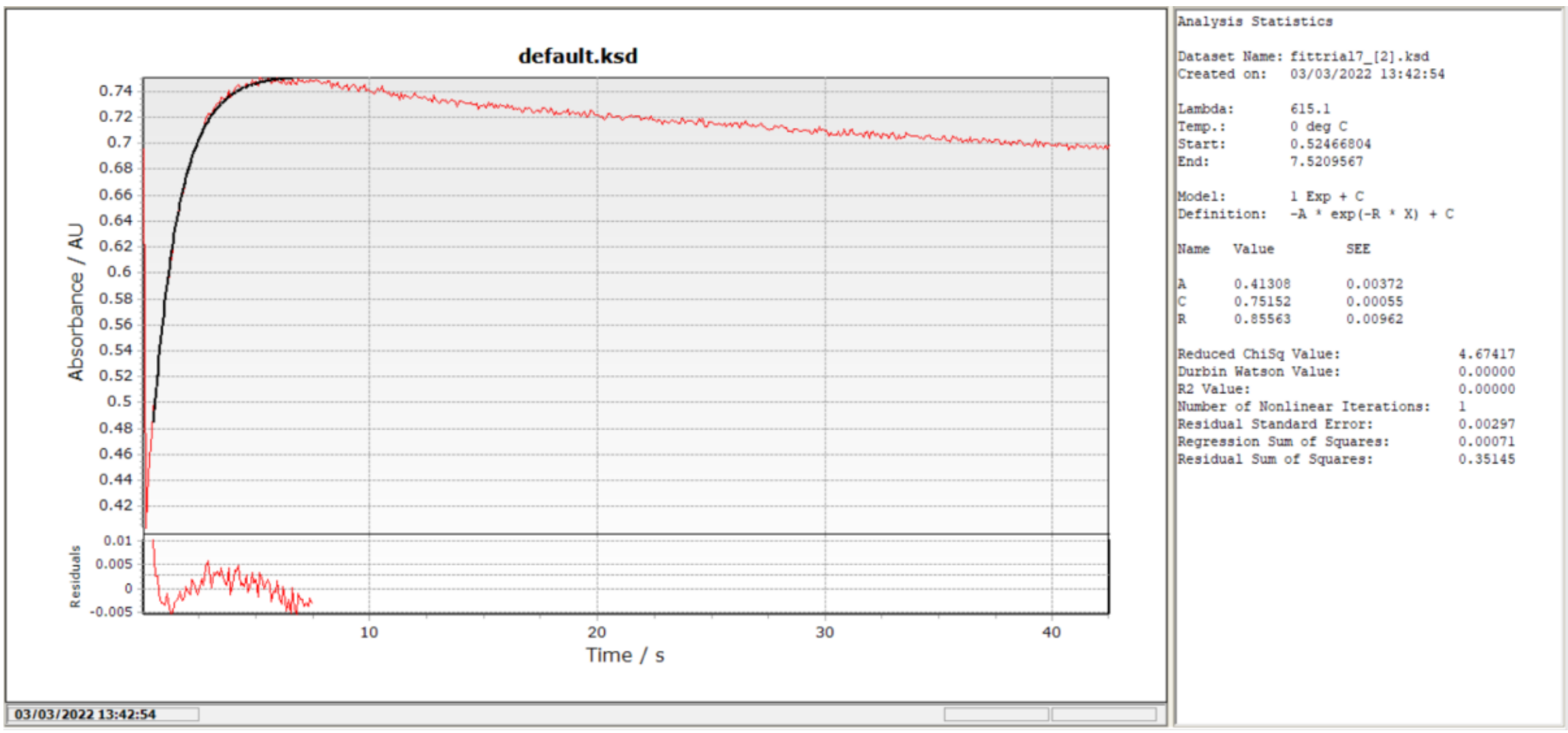

Figure S144. Exemplary trace (615 nm) and fit for the UV-Vis stopped-flow measurement of the reaction of **5a** (1 mM) with 56.25 mM Xan at 25 °C in a mixture of MeCN and DCM.

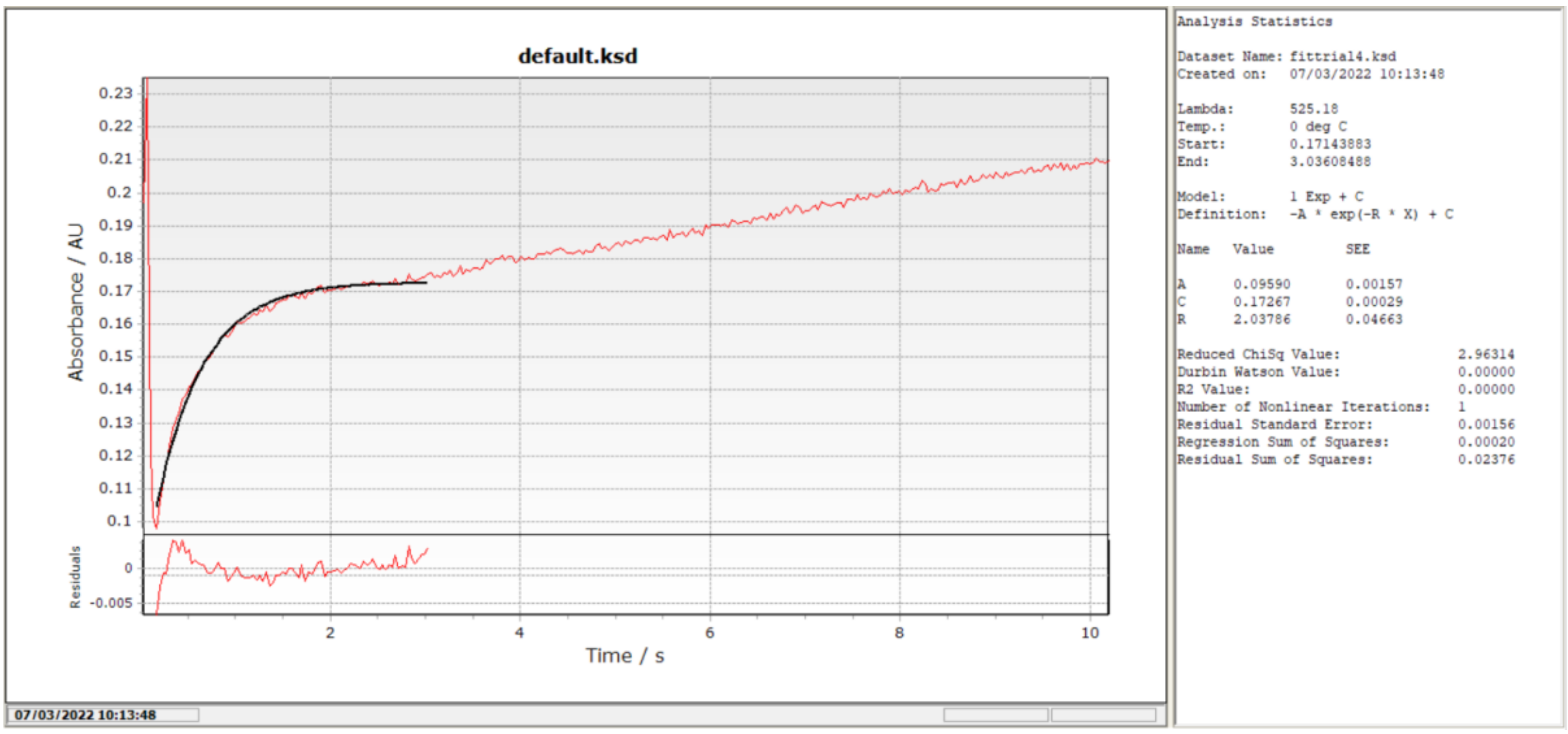

Figure S145. Exemplary trace (525 nm) and fit for the UV-Vis stopped-flow measurement of the reaction of **3** (1 mM) with 37.5 mM Xan at 25 °C in a mixture of MeCN and DCM.

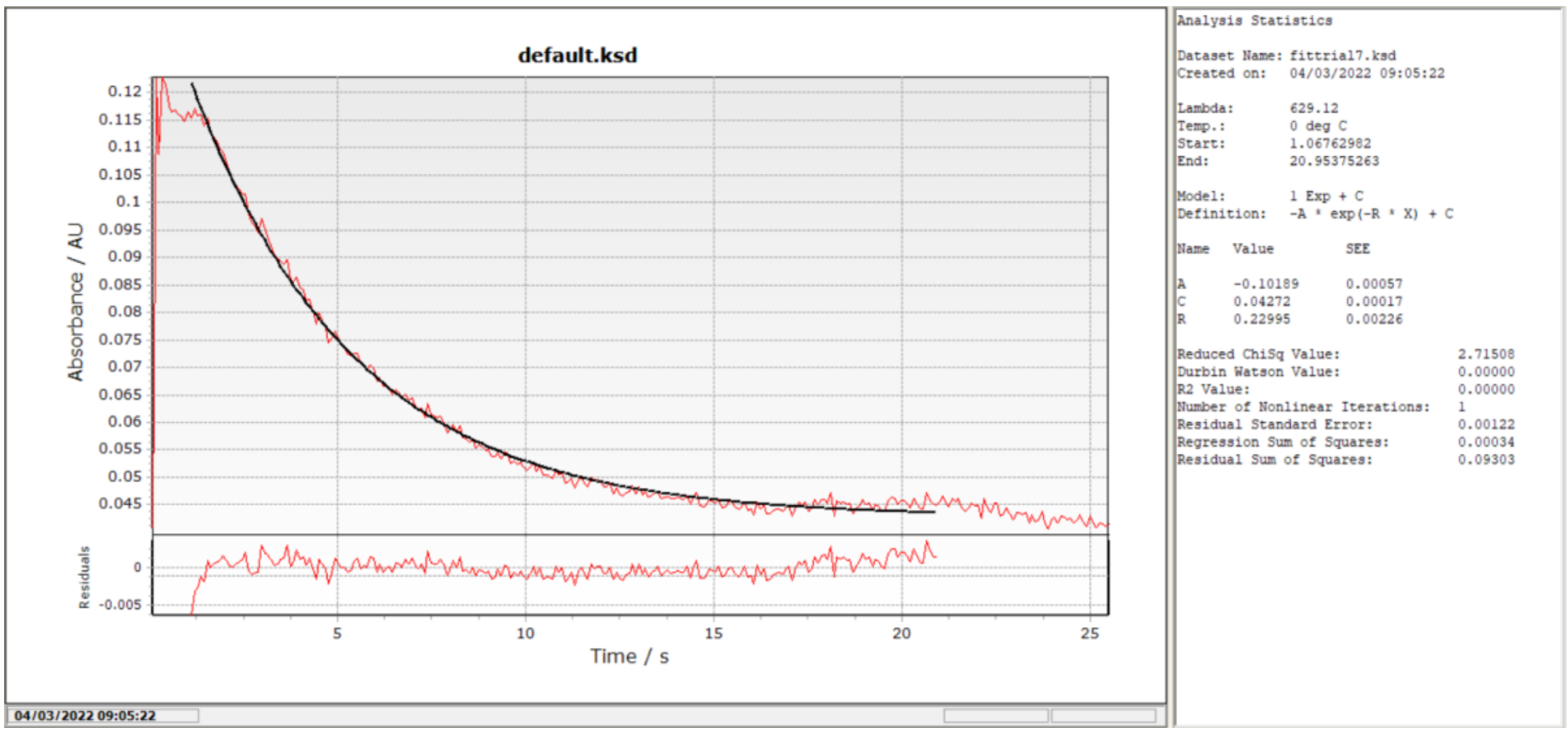

Figure S146. Exemplary trace (629 nm) and fit for the UV-Vis stopped-flow measurement of the reaction of **4** (1 mM) with 37.5 mM Xan at 25 °C in a mixture of MeCN and DCM.

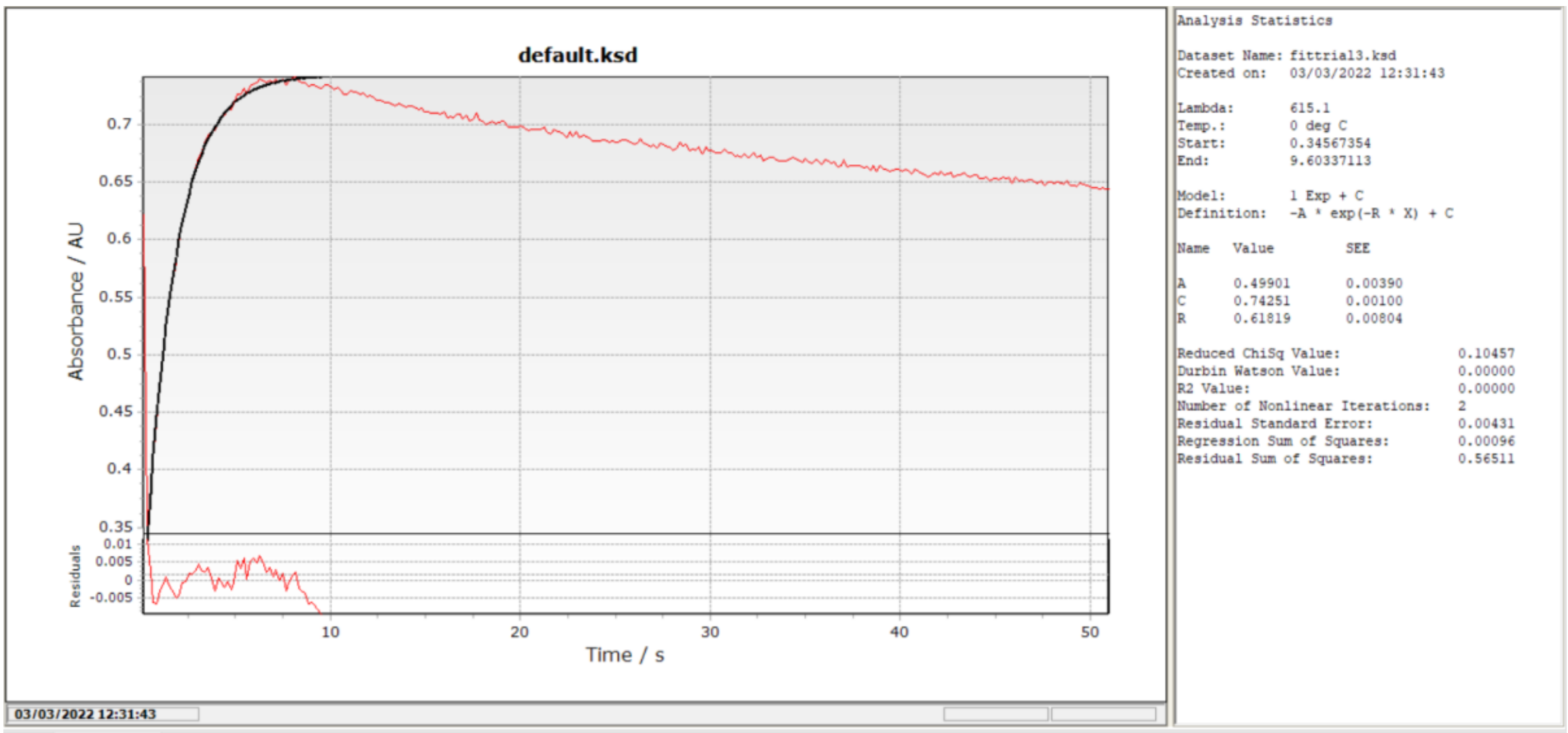

Figure S147. Exemplary trace (615 nm) and fit for the UV-Vis stopped-flow measurement of the reaction of **5a** (1 mM) with 37.5 mM Xan at 25 °C in a mixture of MeCN and DCM.

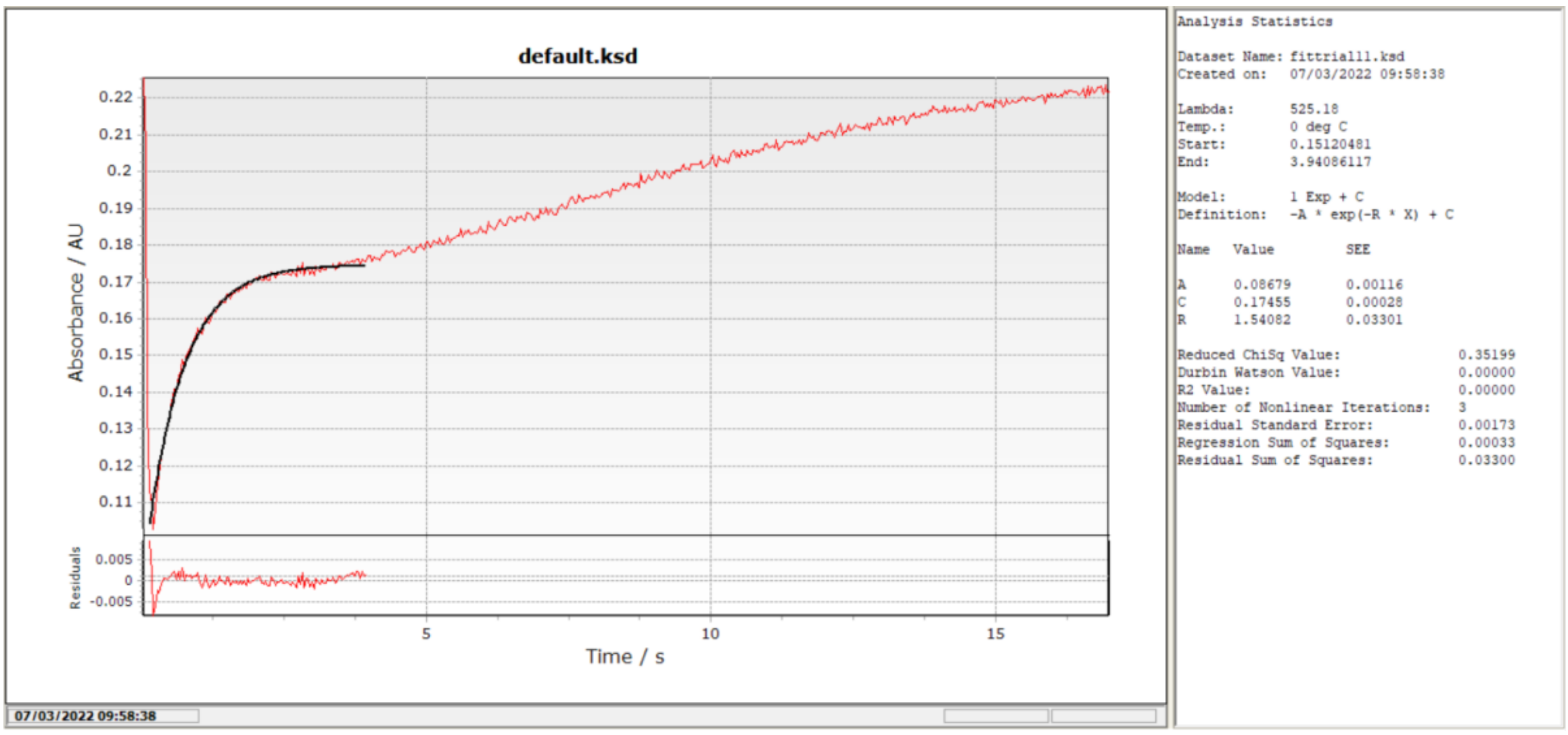

Figure S148. Exemplary trace (525 nm) and fit for the UV-Vis stopped-flow measurement of the reaction of **3** (1 mM) with 28.125 mM Xan at 25 °C in a mixture of MeCN and DCM.

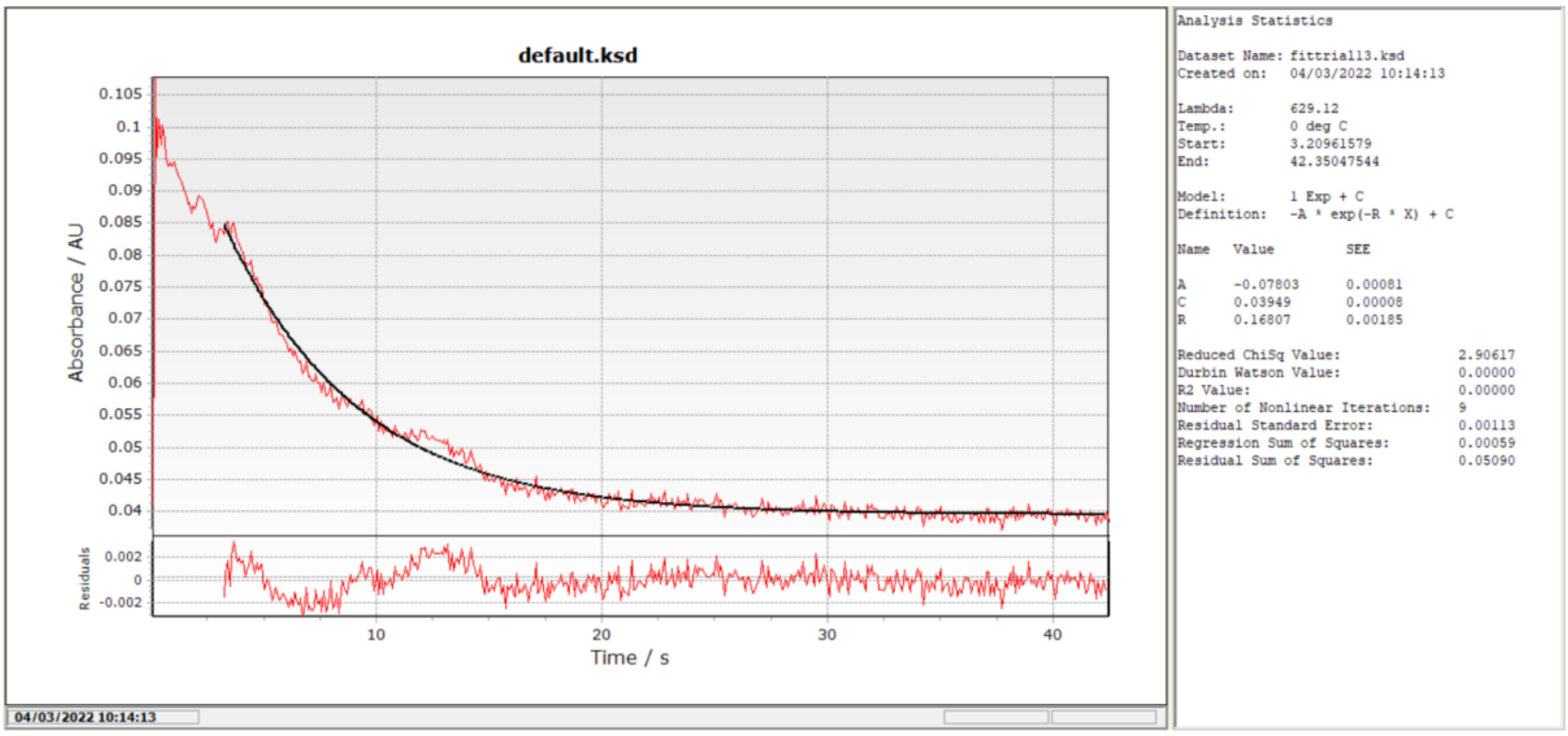

Figure S149. Exemplary trace (629 nm) and fit for the UV-Vis stopped-flow measurement of the reaction of **4** (1 mM) with 28.125 mM Xan at 25 °C in a mixture of MeCN and DCM.

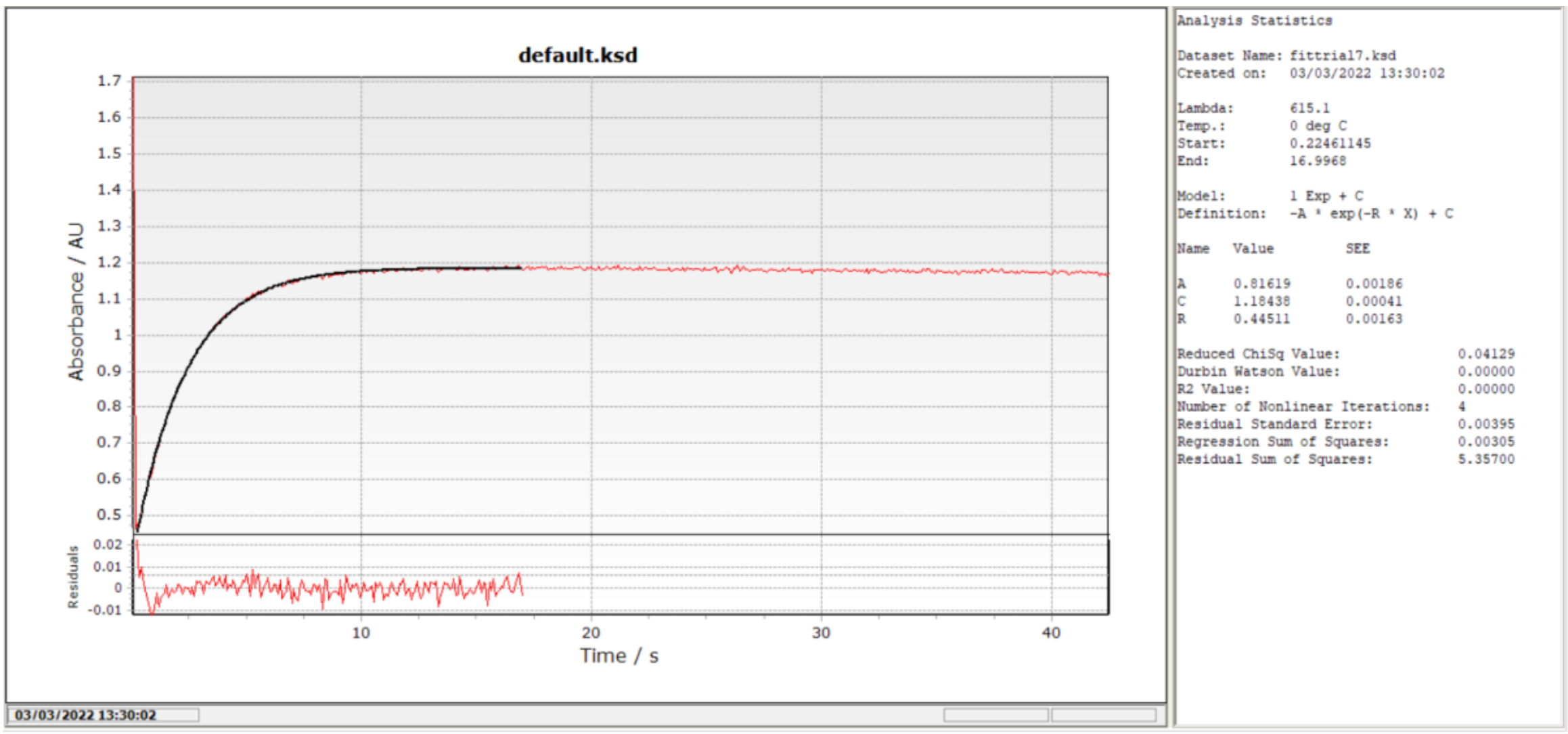

Figure S150. Exemplary trace (615 nm) and fit for the UV-Vis stopped-flow measurement of the reaction of **5a** (1 mM) with 28.125 mM Xan at 25 °C in a mixture of MeCN and DCM.

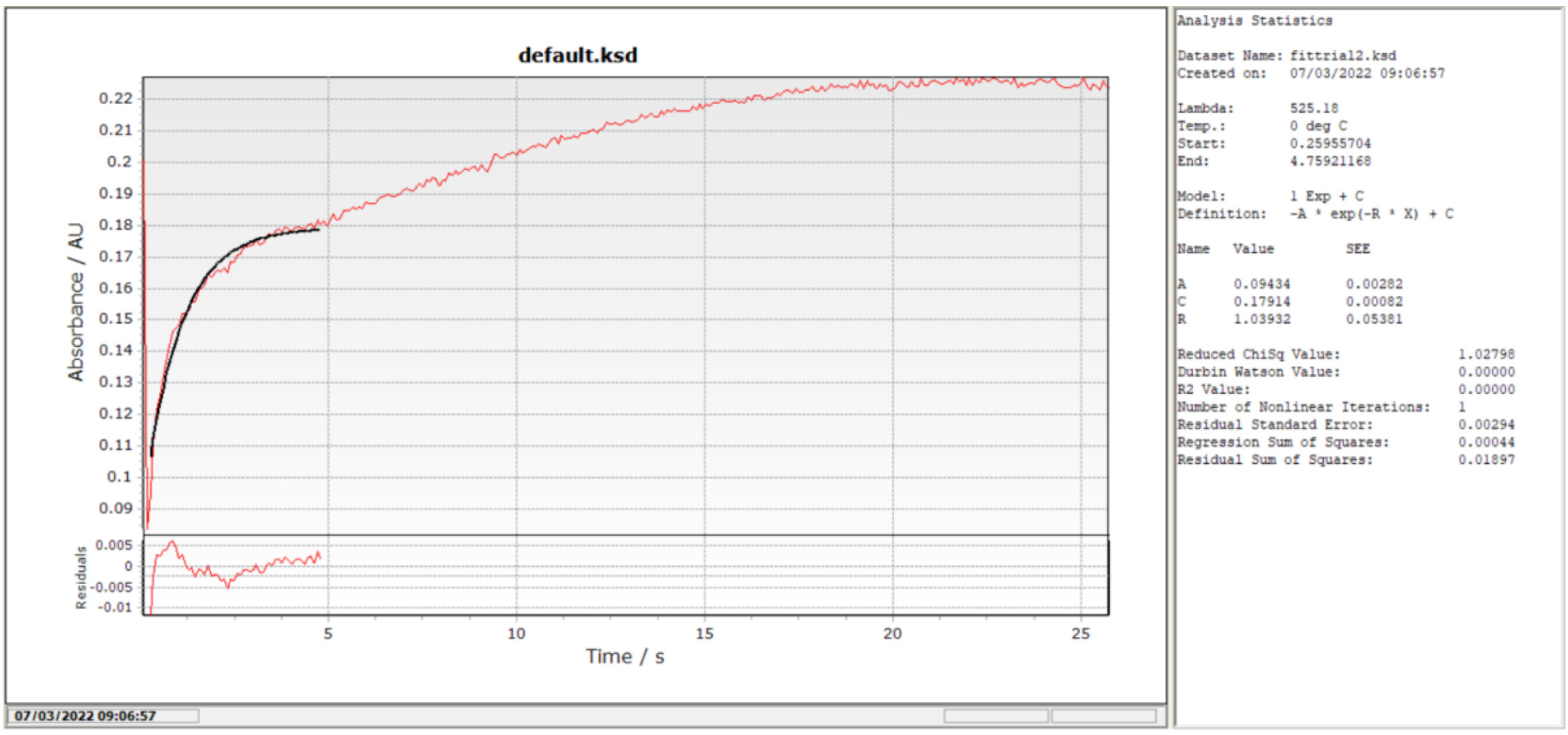

Figure S151. Exemplary trace (525 nm) and fit for the UV-Vis stopped-flow measurement of the reaction of **3** (1 mM) with 18.75 mM Xan at 25 °C in a mixture of MeCN and DCM.

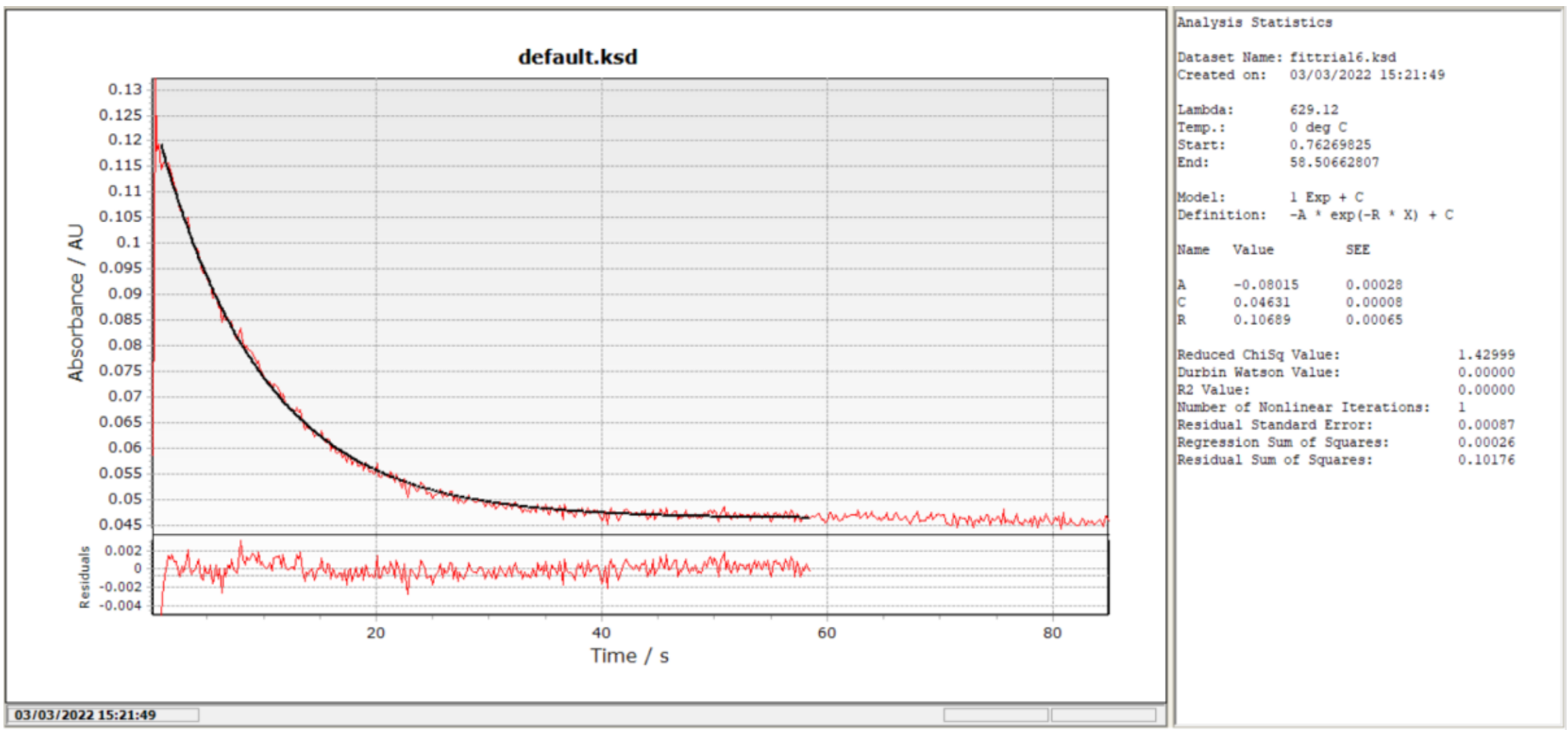

Figure S152. Exemplary trace (629 nm) and fit for the UV-Vis stopped-flow measurement of the reaction of **4** (1 mM) with 18.75 mM Xan at 25 °C in a mixture of MeCN and DCM.

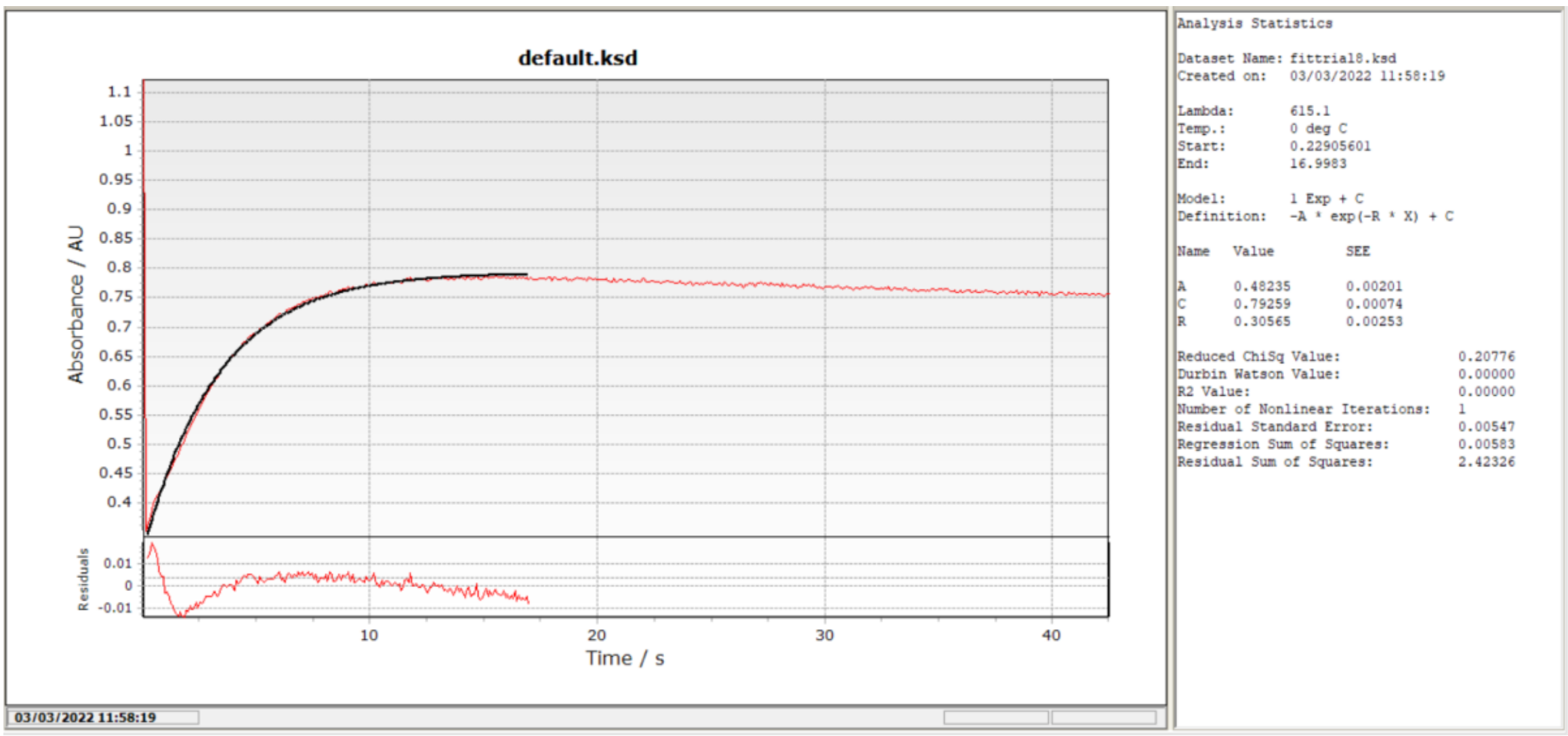

Figure S153. Exemplary trace (615 nm) and fit for the UV-Vis stopped-flow measurement of the reaction of **5a** (1 mM) with 18.75 mM Xan at 25 °C in a mixture of MeCN and DCM.

Kinetic stopped-flow UV-Vis measurements with $AcrH_2$:

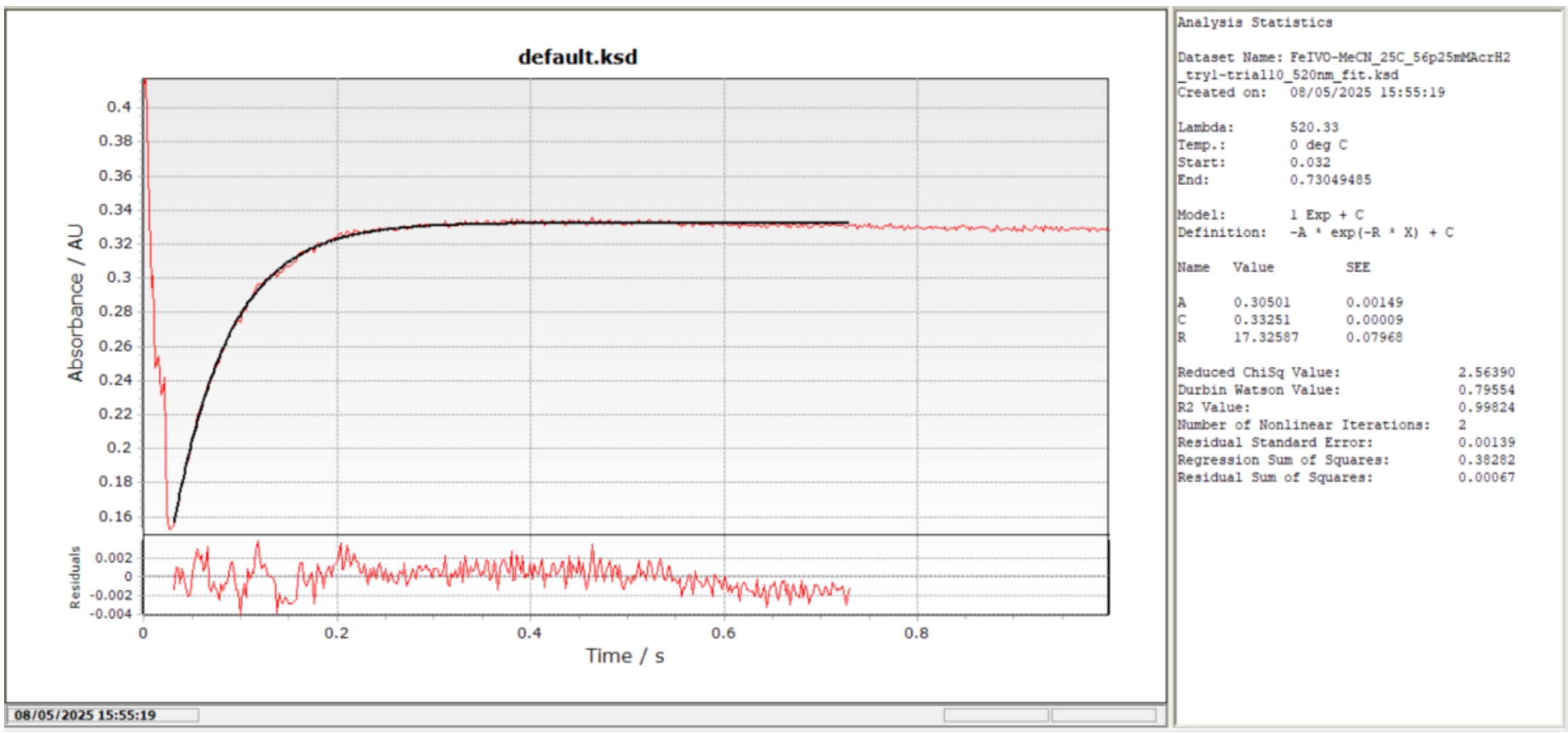

Figure S154. Exemplary trace (520 nm) and fit for the UV-Vis stopped-flow measurement of the reaction of **3** (1 mM) with 56.25 mM $AcrH_2$ at 25 °C in a mixture of MeCN and DCM.

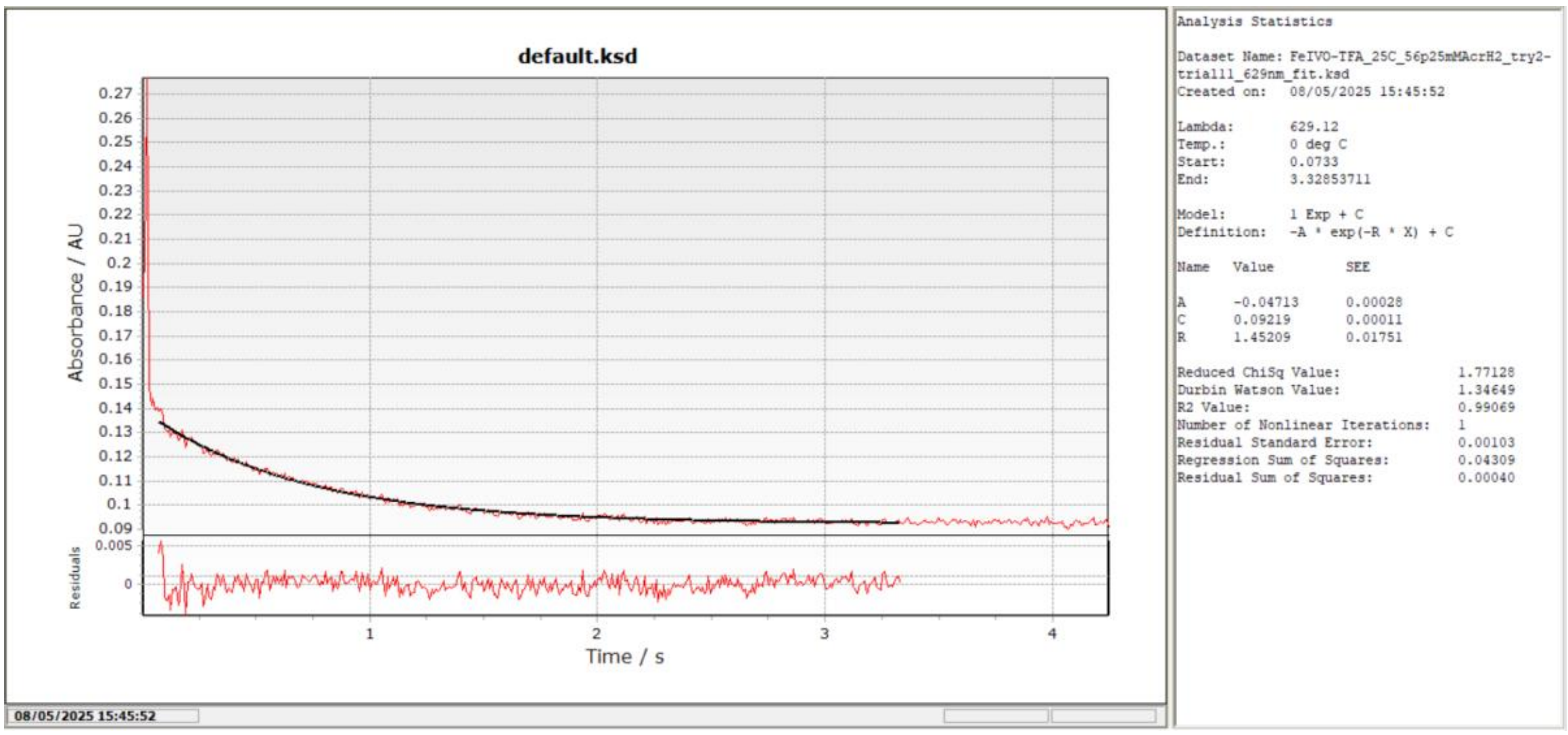

Figure S155. Exemplary trace (629 nm) and fit for the UV-Vis stopped-flow measurement of the reaction of **4** (1 mM) with 56.25 mM $AcrH_2$ at 25 °C in a mixture of MeCN and DCM.

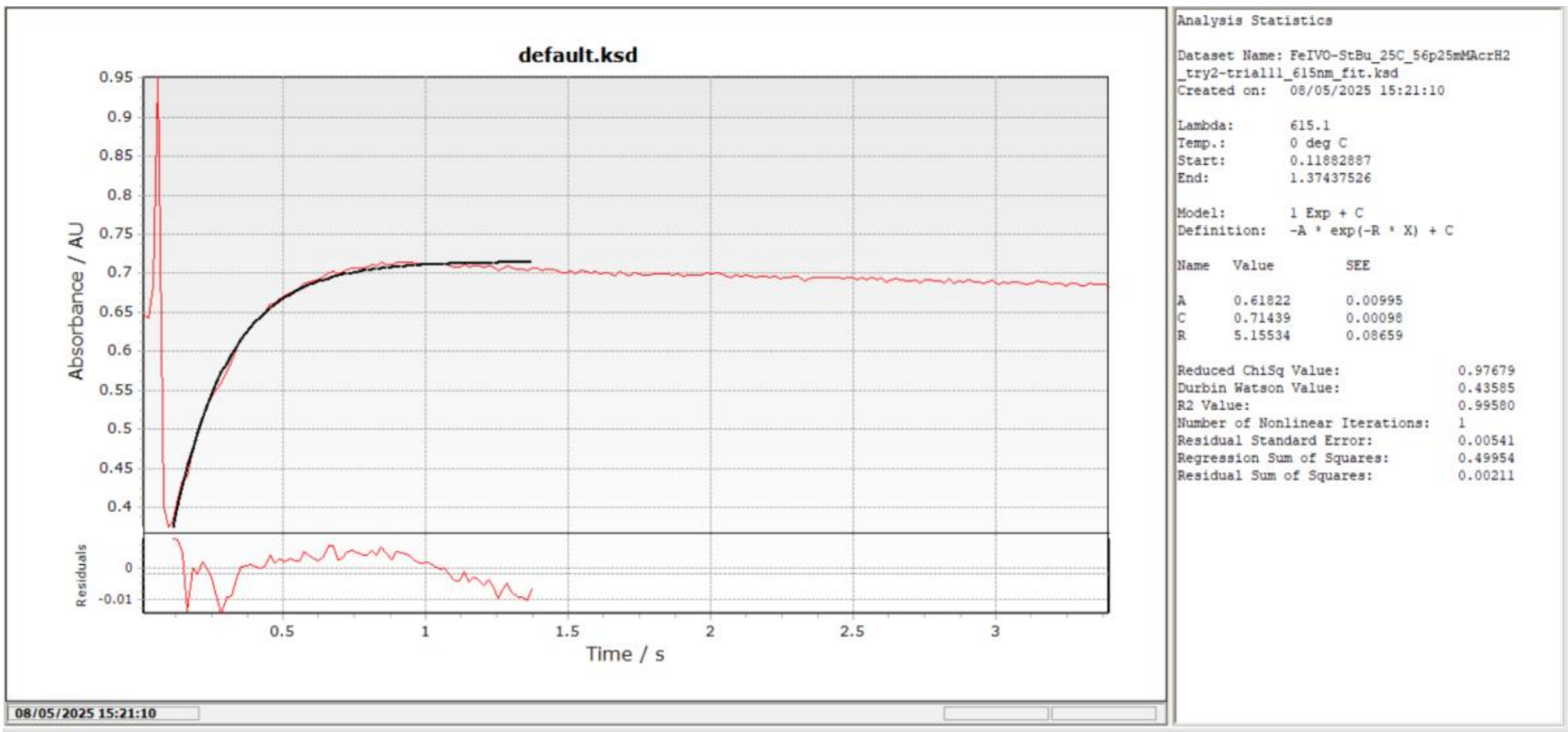

Figure S156. Exemplary trace (615 nm) and fit for the UV-Vis stopped-flow measurement of the reaction of **5a** (1 mM) with 56.25 mM $AcrH_2$ at 25 °C in a mixture of MeCN and DCM.

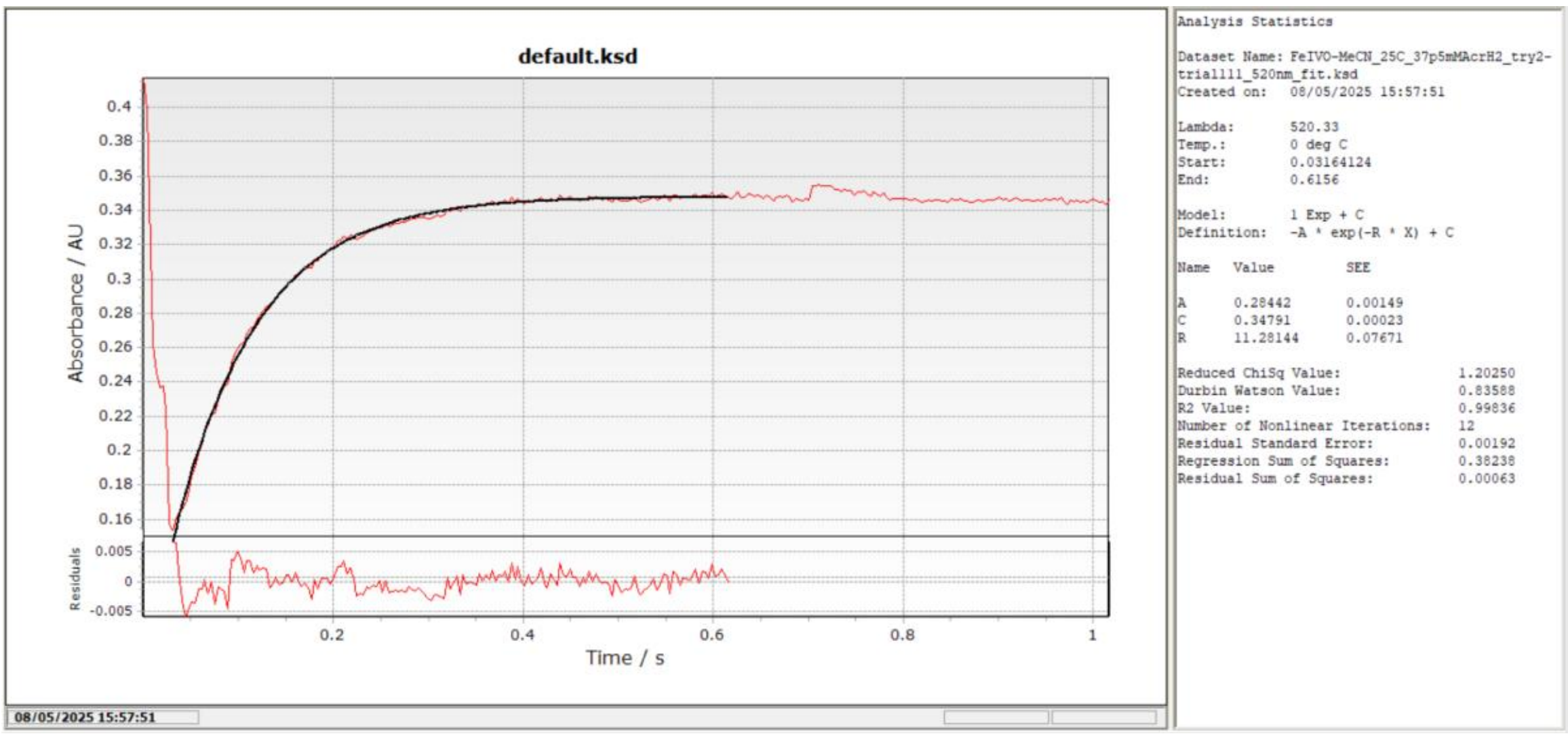

Figure S157. Exemplary trace (520 nm) and fit for the UV-Vis stopped-flow measurement of the reaction of **3** (1 mM) with 37.5 mM $AcrH_2$ at 25 °C in a mixture of MeCN and DCM.

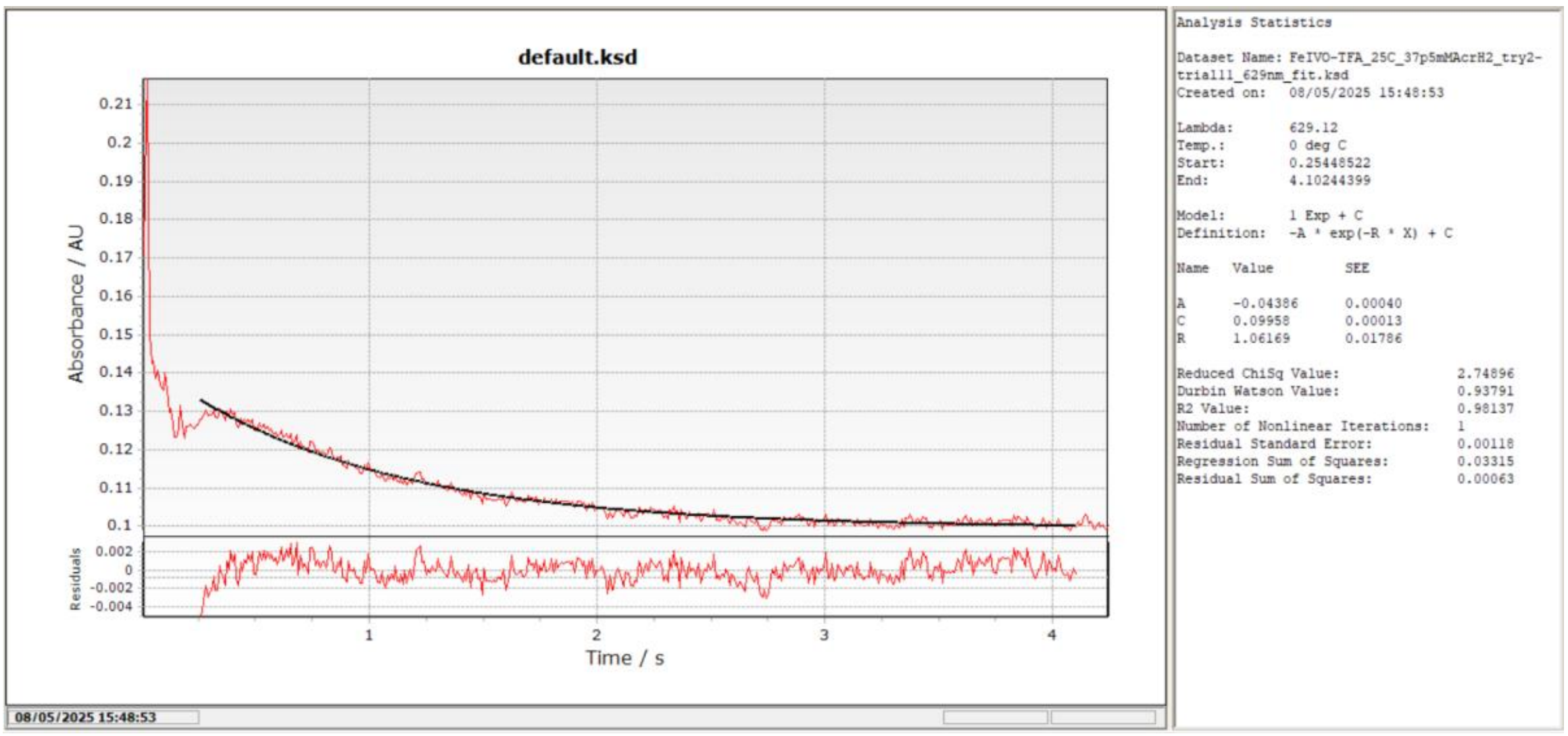

Figure S158. Exemplary trace (629 nm) and fit for the UV-Vis stopped-flow measurement of the reaction of **4** (1 mM) with 37.5 mM $AcrH_2$ at 25 °C in a mixture of MeCN and DCM.

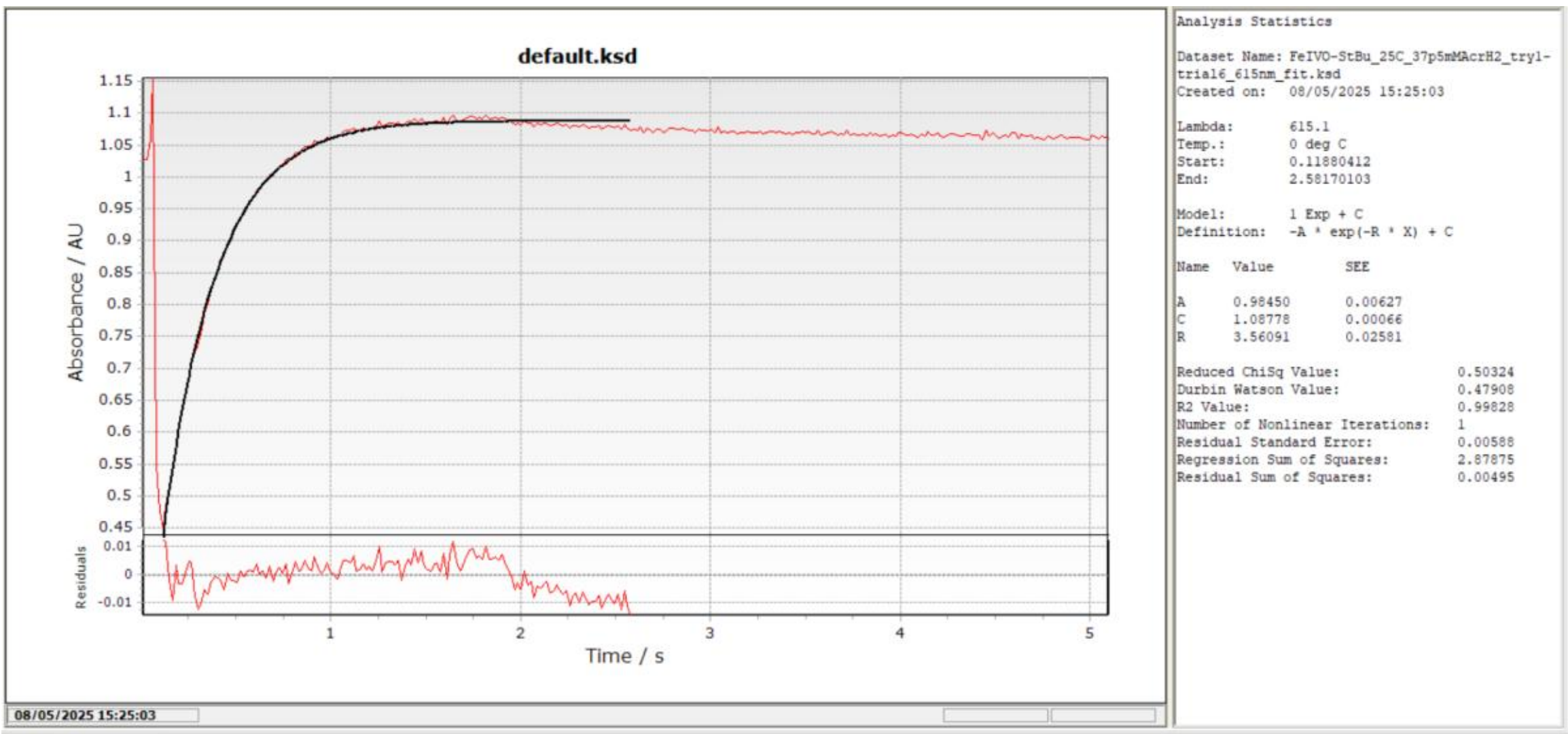

Figure S159. Exemplary trace (615 nm) and fit for the UV-Vis stopped-flow measurement of the reaction of **5a** (1 mM) with 37.5 mM $AcrH_2$ at 25 °C in a mixture of MeCN and DCM.

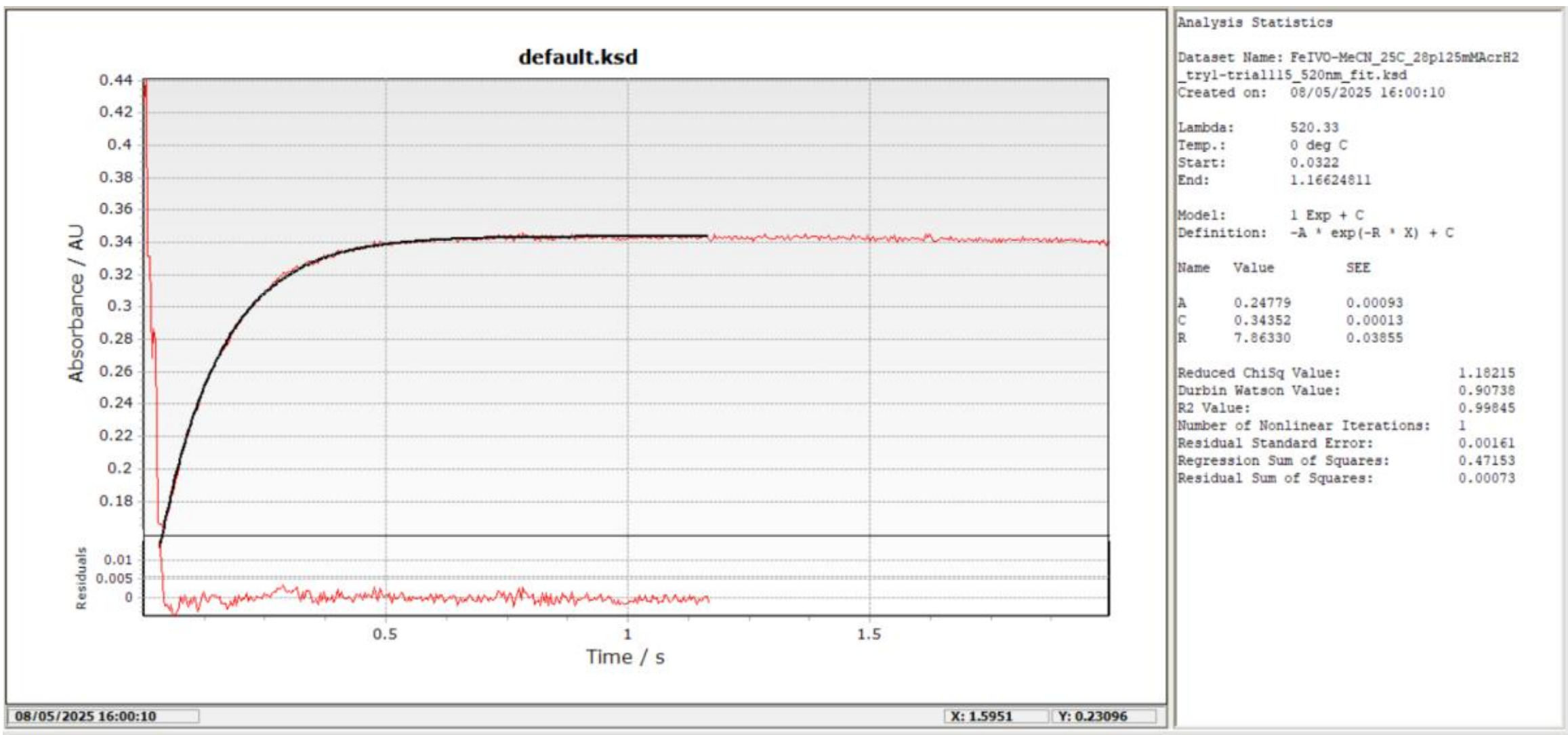

Figure S160. Exemplary trace (520 nm) and fit for the UV-Vis stopped-flow measurement of the reaction of **3** (1 mM) with 28.125 mM $AcrH_2$ at 25 °C in a mixture of MeCN and DCM.

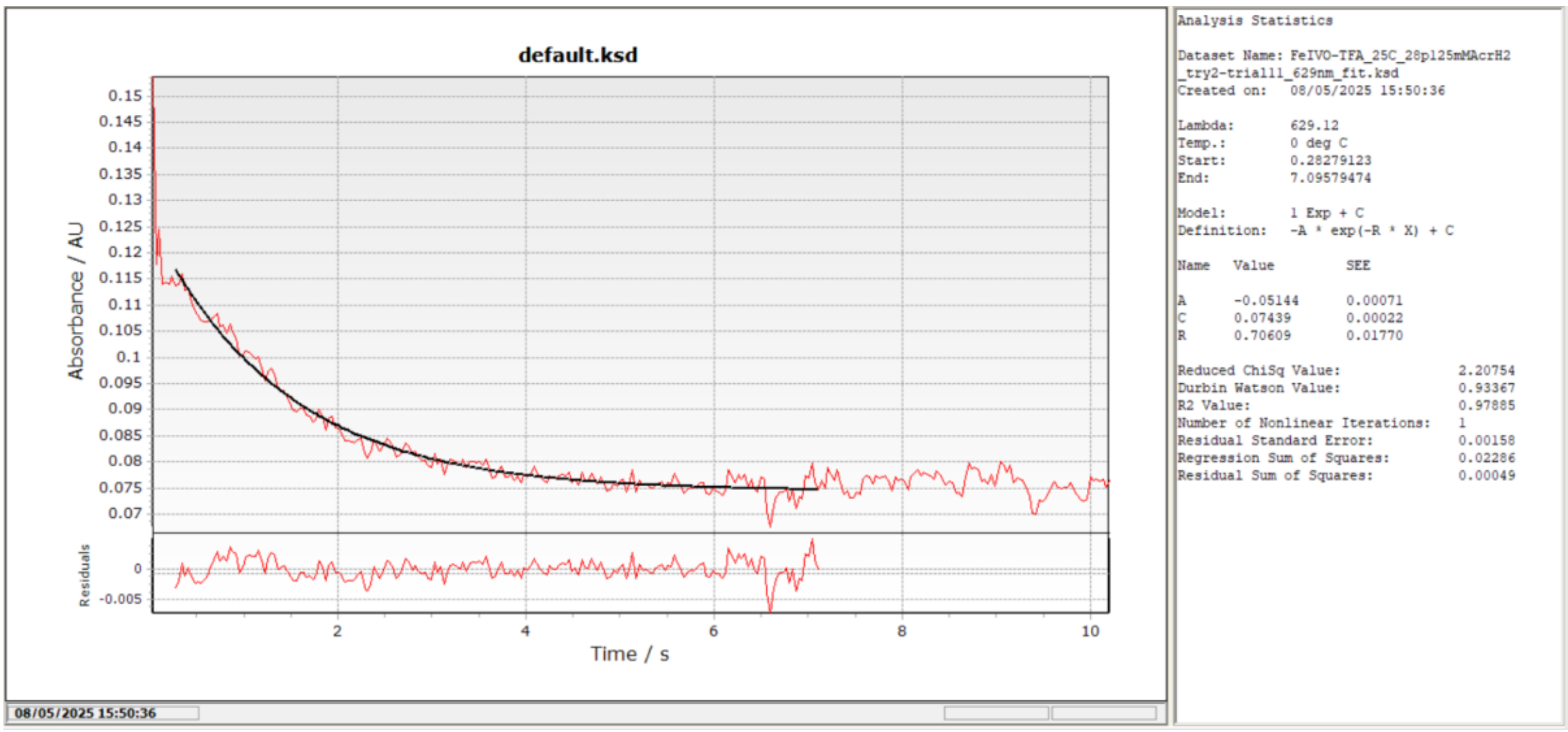

Figure S161. Exemplary trace (629 nm) and fit for the UV-Vis stopped-flow measurement of the reaction of **4** (1 mM) with 28.125 mM $AcrH_2$ at 25 °C in a mixture of MeCN and DCM.

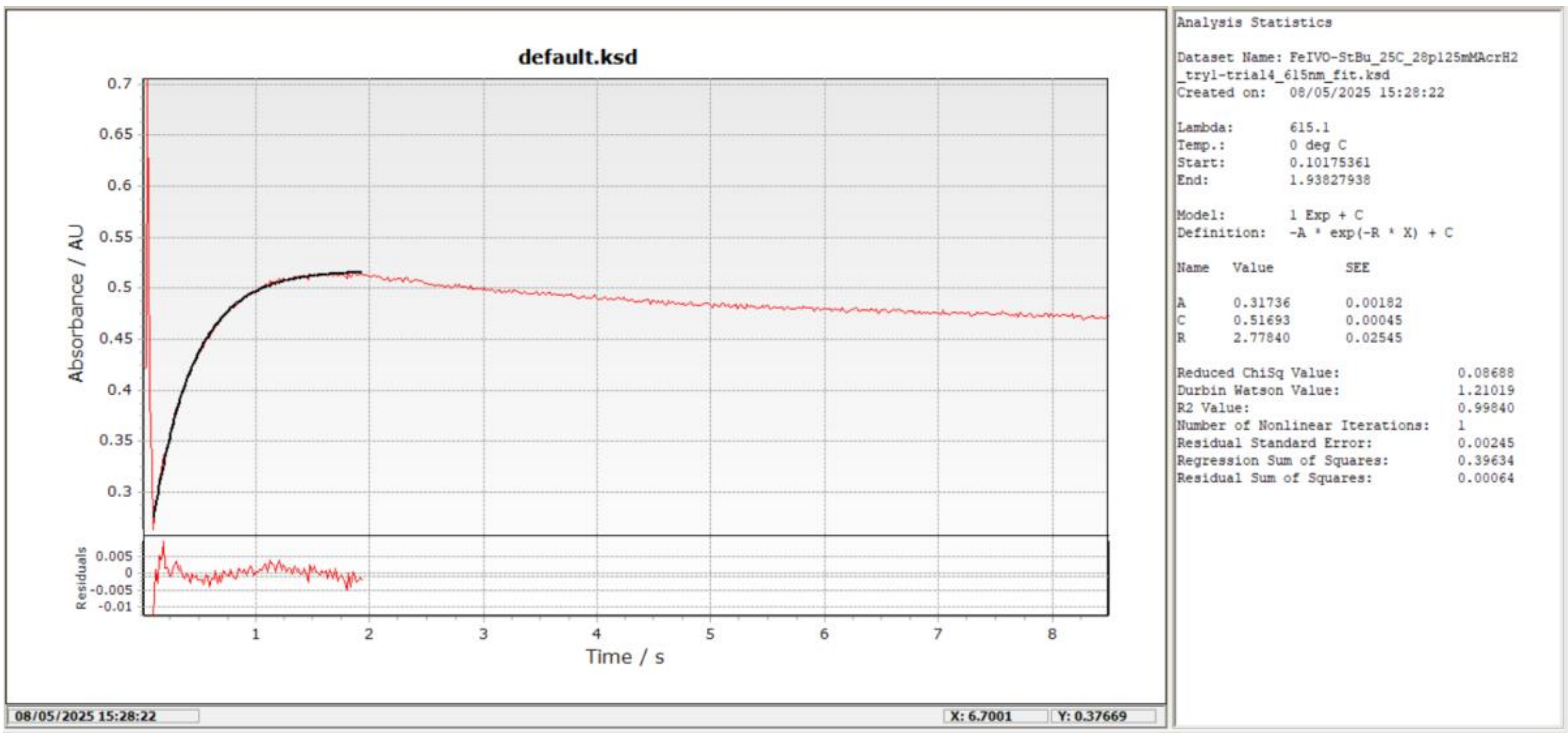

Figure S162. Exemplary trace (615 nm) and fit for the UV-Vis stopped-flow measurement of the reaction of **5a** (1 mM) with 28.125 mM $AcrH_2$ at 25 °C in a mixture of MeCN and DCM.

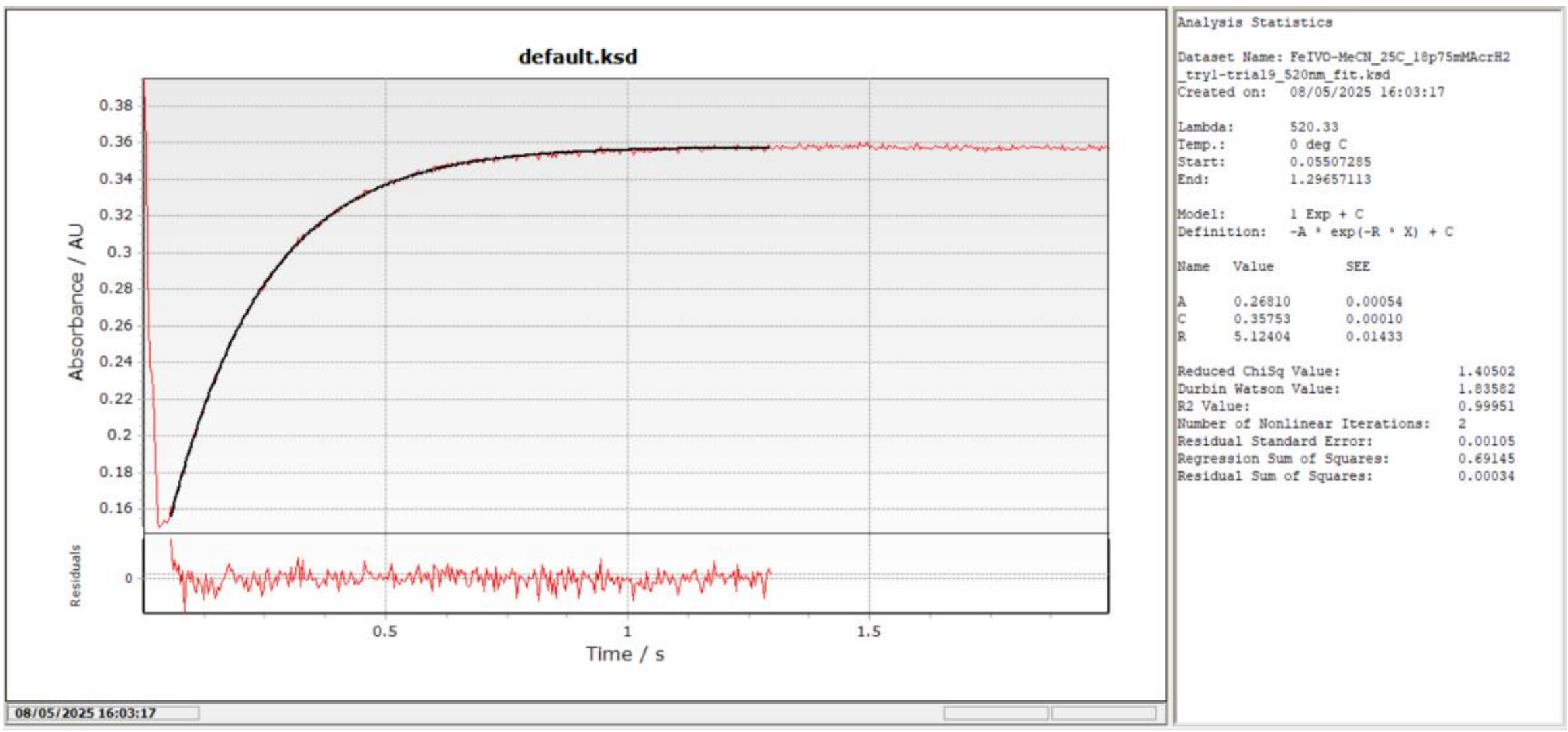

Figure S163. Exemplary trace (520 nm) and fit for the UV-Vis stopped-flow measurement of the reaction of **3** (1 mM) with 18.75 mM $AcrH_2$ at 25 °C in a mixture of MeCN and DCM.

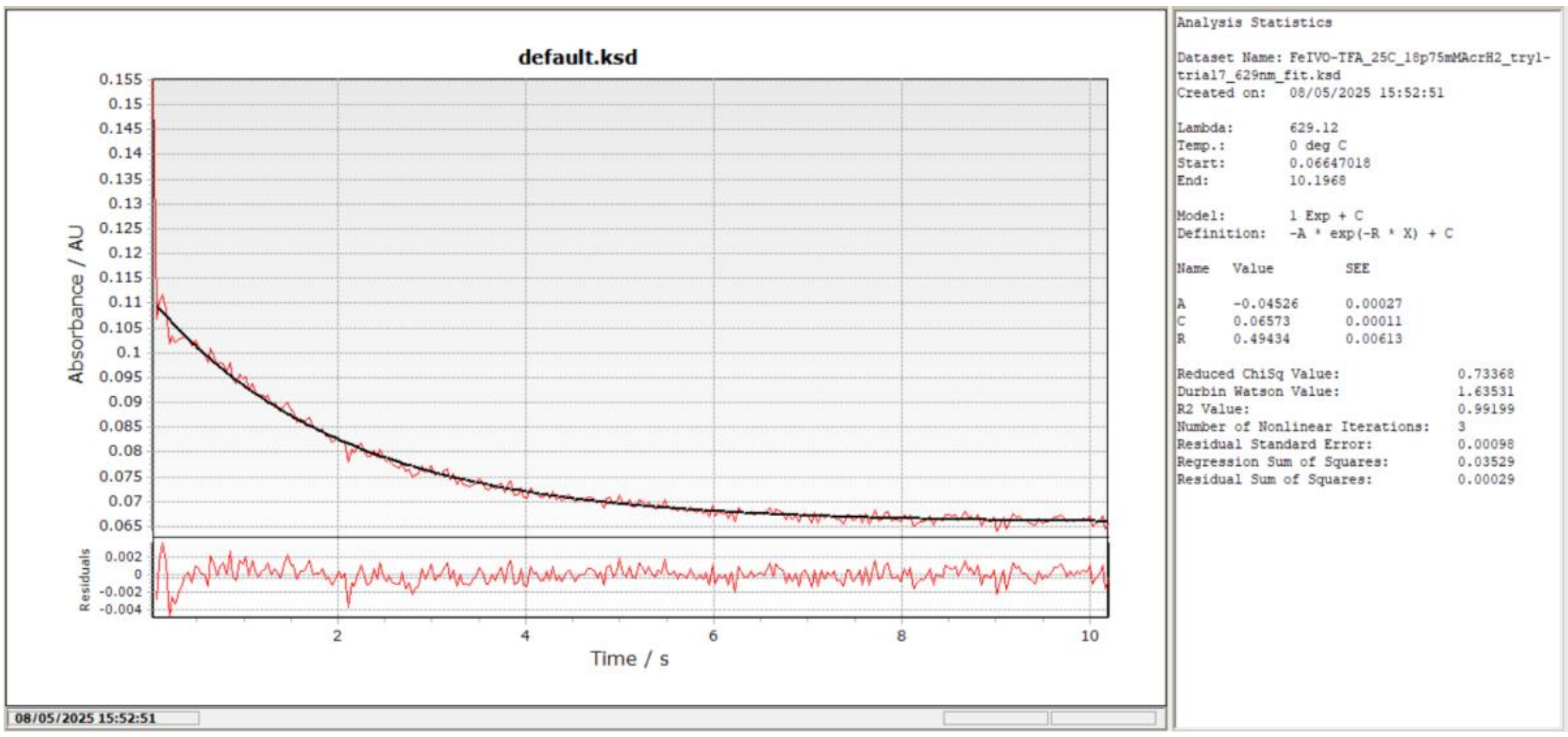

Figure S164. Exemplary trace (629 nm) and fit for the UV-Vis stopped-flow measurement of the reaction of **4** (1 mM) with 18.75 mM $AcrH_2$ at 25 °C in a mixture of MeCN and DCM.

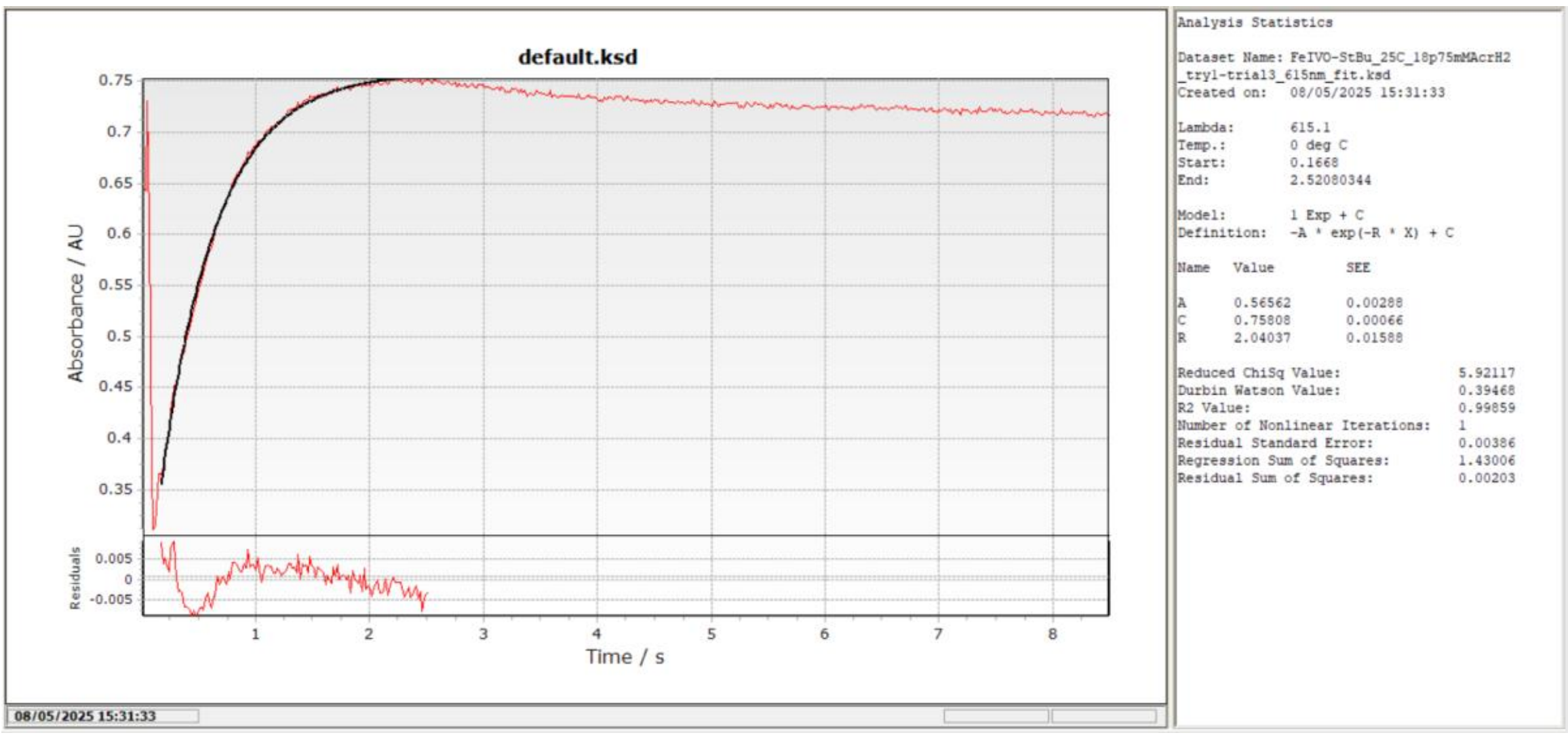

Figure S165. Exemplary trace (615 nm) and fit for the UV-Vis stopped-flow measurement of the reaction of **5a** (1 mM) with 18.75 mM $AcrH_2$ at 25 °C in a mixture of MeCN and DCM.

10 Optimized Coordinates

3' (S = 1)

26	0.015809000	0.025761000	0.341755000
8	0.048462000	0.017849000	1.959638000
6	0.245963000	1.947706000	0.018189000
6	-0.124298000	-1.961037000	0.013180000
6	-1.971896000	0.238719000	0.064829000
6	1.940867000	-0.069395000	-0.025325000
7	-2.882665000	1.152318000	0.478959000
7	-2.694009000	-0.657430000	-0.653730000
7	-1.146496000	-2.498449000	-0.698737000
7	0.629976000	-3.025629000	0.378668000
7	2.849406000	-0.856386000	0.572218000
7	2.637754000	0.707649000	-0.881291000
7	1.106977000	2.522245000	-0.848029000
7	-0.365420000	2.963009000	0.648865000
6	-4.153076000	0.822801000	0.025829000
1	-5.020367000	1.425787000	0.262461000
6	-4.034431000	-0.320504000	-0.697780000
1	-4.767919000	-0.911955000	-1.230899000
6	-2.117459000	-1.724795000	-1.447247000
1	-2.920841000	-2.392145000	-1.762128000
1	-1.643899000	-1.304440000	-2.341215000
6	-1.043483000	-3.874652000	-0.787248000
1	-1.762620000	-4.481063000	-1.322942000
6	0.079213000	-4.207258000	-0.098714000
1	0.530877000	-5.171107000	0.098676000
6	1.908007000	-3.100336000	1.109138000
1	2.624513000	-3.589663000	0.439318000
1	1.754177000	-3.757887000	1.971920000
6	2.495831000	-1.802559000	1.626447000
1	1.809706000	-1.300250000	2.312173000
1	3.409280000	-2.055018000	2.171444000
6	4.123798000	-0.589550000	0.091574000
1	5.000773000	-1.111228000	0.453251000
6	3.991245000	0.403110000	-0.834339000
1	4.723599000	0.911479000	-1.448588000
6	2.012395000	1.748183000	-1.684609000
1	1.463904000	1.300992000	-2.519886000
1	2.786114000	2.407485000	-2.079949000
6	1.029572000	3.906487000	-0.776734000
1	1.636389000	4.555730000	-1.395054000
6	0.095157000	4.184388000	0.177249000
1	-0.266461000	5.128750000	0.564021000
6	-1.327494000	2.751030000	1.726638000
1	-1.405255000	3.680915000	2.296263000
1	-0.929144000	1.973938000	2.382739000
6	-2.720467000	2.403128000	1.241394000
1	-3.368158000	2.336391000	2.122532000
1	-3.102710000	3.209677000	0.605224000

3' (S = 2)

26	0.014746000	0.020554000	0.504672000
8	0.029262000	-0.008841000	2.232101000
6	0.254476000	1.940727000	0.069100000
6	-0.082436000	-1.959181000	0.043358000
6	-1.961635000	0.280366000	0.089349000
6	1.934996000	-0.062971000	0.017830000
7	-2.885825000	1.176378000	0.496013000
7	-2.641036000	-0.608284000	-0.672832000
7	-1.090424000	-2.456199000	-0.711063000
7	0.651278000	-3.033098000	0.405251000
7	2.844735000	-0.869628000	0.584317000
7	2.600642000	0.677948000	-0.887225000
7	1.072316000	2.494776000	-0.844652000
7	-0.375475000	2.958331000	0.674591000
6	-4.138194000	0.844622000	-0.005982000
1	-5.017252000	1.438014000	0.210576000
6	-3.985510000	-0.285510000	-0.744695000
1	-4.697093000	-0.877071000	-1.306633000
6	-2.014248000	-1.640647000	-1.476917000
1	-2.795848000	-2.287769000	-1.876143000
1	-1.479896000	-1.174973000	-2.312317000
6	-1.003362000	-3.833525000	-0.822658000
1	-1.718865000	-4.418720000	-1.386137000
6	0.099812000	-4.196342000	-0.117159000
1	0.539009000	-5.169217000	0.063286000
6	1.916129000	-3.113583000	1.157907000
1	2.635388000	-3.623771000	0.507289000
1	1.742488000	-3.750764000	2.032040000
6	2.503102000	-1.805068000	1.654457000
1	1.811139000	-1.290192000	2.327630000
1	3.418637000	-2.041910000	2.202673000
6	4.096201000	-0.653064000	0.027259000
1	4.974680000	-1.199704000	0.345863000
6	3.944604000	0.333878000	-0.903811000
1	4.660254000	0.817842000	-1.556356000
6	1.953945000	1.704511000	-1.691074000
1	1.376872000	1.238300000	-2.496718000
1	2.715827000	2.354556000	-2.123121000
6	0.954482000	3.877215000	-0.828573000
1	1.533483000	4.517366000	-1.482131000
6	0.030672000	4.169095000	0.133524000
1	-0.355077000	5.118362000	0.483307000
6	-1.332236000	2.751078000	1.760247000
1	-1.394818000	3.676030000	2.339425000
1	-0.931254000	1.959241000	2.399953000
6	-2.732169000	2.414731000	1.280663000
1	-3.374086000	2.329514000	2.164268000
1	-3.121239000	3.229385000	0.659395000

3 (S = 1)

26	-0.002481000	0.029489000	0.002888000
8	0.022631000	0.001379000	1.647875000
6	0.280918000	1.992944000	-0.112147000
6	-0.180168000	-2.008404000	-0.135849000
6	-2.045011000	0.211135000	-0.067433000
6	1.976186000	-0.023428000	-0.171789000
7	-0.099906000	0.009713000	-2.068776000
7	-2.924368000	1.128207000	0.406953000
7	-2.834813000	-0.757944000	-0.596704000
7	-1.288586000	-2.600702000	-0.649166000
7	0.585812000	-3.047925000	0.278419000
7	2.845574000	-0.789554000	0.507229000
7	2.744629000	0.820930000	-0.899257000
7	1.216432000	2.631224000	-0.853442000
7	-0.309340000	2.957758000	0.611858000

6	-4.236896000	0.735351000	0.167522000
1	-5.080638000	1.337949000	0.478002000
6	-4.182207000	-0.460261000	-0.467810000
1	-4.961421000	-1.119167000	-0.828808000
6	-2.367078000	-1.915510000	-1.327824000
1	-3.200850000	-2.612048000	-1.427955000
1	-2.050079000	-1.620628000	-2.331367000
6	-1.225502000	-3.982882000	-0.567461000
1	-2.021195000	-4.624640000	-0.923208000
6	-0.036940000	-4.264177000	0.019386000
1	0.420461000	-5.208976000	0.283651000
6	1.944570000	-3.077189000	0.840490000
1	2.632552000	-3.393731000	0.046765000
1	1.944665000	-3.856752000	1.609132000
6	2.447844000	-1.801078000	1.479372000
1	1.695518000	-1.364712000	2.141698000
1	3.332980000	-2.052147000	2.069403000
6	4.158575000	-0.449409000	0.202919000
1	5.004708000	-0.957425000	0.647731000
6	4.096243000	0.577888000	-0.686502000
1	4.873143000	1.151084000	-1.176094000
6	2.205403000	1.935640000	-1.659958000
1	1.765128000	1.587200000	-2.595279000
1	3.016580000	2.626290000	-1.893347000
6	1.204257000	3.998874000	-0.606129000
1	1.881573000	4.684212000	-1.099513000
6	0.230161000	4.205412000	0.320669000
1	-0.117520000	5.111221000	0.800724000
6	-1.336524000	2.703568000	1.615240000
1	-1.409739000	3.596880000	2.241111000
1	-1.009431000	1.865220000	2.235696000
6	-2.703290000	2.447125000	1.019029000
1	-3.440568000	2.543596000	1.822564000
1	-2.931075000	3.212007000	0.266300000
6	-0.189629000	-0.025685000	-3.211732000
6	-0.307527000	-0.076340000	-4.654656000
1	-0.561292000	0.918563000	-5.038095000
1	-1.097137000	-0.784668000	-4.931607000
1	0.642985000	-0.405676000	-5.089804000

3 (*S* = 2)

26	0.003541236	-0.015891780	-0.087875234
8	0.005215105	-0.070000243	1.558865901
6	0.365379881	2.018170235	-0.132040777
6	-0.246859831	-2.081382423	-0.205676856
6	-2.256582410	0.281486570	-0.220224965
6	2.214136871	-0.116576574	-0.310641651
7	-0.085056043	0.004012983	-2.190295377
7	-3.101897965	1.176840419	0.328373087
7	-3.026795784	-0.805685347	-0.461418875
7	-1.439490833	-2.649200428	-0.503938567
7	0.547727860	-3.103025505	0.177351229
7	3.030715643	-0.835121220	0.475317671
7	2.980736239	0.890042375	-0.783704090
7	1.372446608	2.655678257	-0.768348334
7	-0.310832230	2.977336224	0.522708795
6	-4.391083448	0.662425748	0.425582361
1	-5.213810009	1.234886687	0.834887836
6	-4.344897002	-0.601296934	-0.072846960
1	-5.113599248	-1.355176412	-0.188195116
6	-2.549134889	-1.974761963	-1.167148568
1	-3.369683510	-2.687754222	-1.254643913
1	-2.233600113	-1.686086169	-2.173272067
6	-1.403401627	-4.022050052	-0.305994840
1	-2.260203826	-4.657897761	-0.488243599
6	-0.147000074	-4.307217824	0.122043611
1	0.315807681	-5.248626852	0.388785694
6	1.999501010	-3.110691295	0.428461346
1	2.517378858	-3.159107187	-0.536504625
1	2.196345618	-4.048554596	0.953536012
6	2.575544033	-1.975405255	1.267456334
1	1.846991171	-1.625954269	2.006480883
1	3.447605460	-2.361300360	1.801021434
6	4.312346056	-0.296532574	0.498292909
1	5.122052666	-0.742054124	1.062689215
6	4.279880077	0.810901359	-0.295283524
1	5.050157089	1.524895017	-0.558720280
6	2.406702726	1.977976681	-1.548341651
1	1.975550608	1.592384453	-2.474140052
1	3.184844533	2.701301366	-1.794372553
6	1.324392011	4.021200432	-0.526699070
1	2.049159179	4.710146224	-0.941510077
6	0.251060359	4.225187056	0.286264671
1	-0.149856560	5.131160433	0.722925876
6	-1.451068405	2.746868698	1.411702365
1	-1.527300079	3.627763374	2.054106819
1	-1.224916099	1.881322333	2.040723734
6	-2.784925142	2.560226533	0.694918924
1	-3.579975378	2.891418326	1.368635439
1	-2.826994499	3.191315493	-0.201008432
6	-0.164602264	-0.016534197	-3.334349137
6	-0.268118337	-0.044673129	-4.779449197
1	-0.514229634	0.957374409	-5.149129999
1	-1.057148550	-0.745326574	-5.075938384
1	0.686168827	-0.368634737	-5.210262241

4 (*S* = 1)

26	-0.109900000	0.044618000	-0.055615000
8	0.019630000	-0.024980000	1.582448000
6	0.169669000	2.012580000	-0.125905000
6	-0.339802000	-1.981144000	-0.208949000
6	-2.157392000	0.275307000	-0.013947000
6	1.839856000	-0.007035000	-0.360743000
8	-0.325075000	0.107416000	-2.150189000
7	-3.004126000	1.189849000	0.525133000
7	-2.986314000	-0.650997000	-0.558634000
7	-1.504544000	-2.532846000	-0.631126000
7	0.421132000	-3.044421000	0.144290000
7	2.749761000	-0.814739000	0.205263000
7	2.552388000	0.846539000	-1.132441000
7	1.057574000	2.673371000	-0.902138000
7	-0.364638000	2.955454000	0.670206000
6	-4.331955000	0.837404000	0.306577000
1	-5.152410000	1.445571000	0.665333000
6	-4.321775000	-0.331566000	-0.380104000
1	-5.124964000	-0.958241000	-0.746257000
6	-2.544788000	-1.799070000	-1.318251000
1	-3.397440000	-2.463940000	-1.463245000
1	-2.173994000	-1.475747000	-2.293252000
6	-1.479785000	-3.916265000	-0.561184000
1	-2.319503000	-4.530198000	-0.860687000
6	-0.259138000	-4.239685000	-0.066992000

1	0.186506000	-5.199950000	0.158972000
6	1.834736000	-3.104183000	0.539539000
1	2.420906000	-3.384103000	-0.344130000
1	1.920370000	-3.917831000	1.266741000
6	2.418494000	-1.856218000	1.170867000
1	1.732736000	-1.432246000	1.909094000
1	3.346940000	-2.140065000	1.673063000
6	4.033600000	-0.503846000	-0.228128000
1	4.905638000	-1.049941000	0.108269000
6	3.910373000	0.555050000	-1.073396000
1	4.649910000	1.124565000	-1.621506000
6	1.977940000	2.004041000	-1.804803000
1	1.466308000	1.717495000	-2.725127000
1	2.785976000	2.693774000	-2.052179000
6	1.070517000	4.031580000	-0.609309000
1	1.717673000	4.730316000	-1.124049000
6	0.160975000	4.210472000	0.386671000
1	-0.150060000	5.100962000	0.917826000
6	-1.329826000	2.671376000	1.724626000
1	-1.345792000	3.533432000	2.396924000
1	-0.981416000	1.795687000	2.278562000
6	-2.734965000	2.466114000	1.203470000
1	-3.419520000	2.518466000	2.056419000
1	-3.000233000	3.278188000	0.515053000
6	0.223732000	-0.224566000	-3.248255000
8	0.316772000	0.471755000	-4.256551000
6	0.841655000	-1.650527000	-3.330264000
9	1.317552000	-1.904273000	-4.543844000
9	1.857529000	-1.794160000	-2.459169000
9	-0.068453000	-2.595590000	-3.043749000

4 (*S* = 2)

26	-0.128922000	0.081253000	0.355931000
8	0.041548000	-0.060479000	2.074899000
6	0.116631000	2.036164000	0.089597000
6	-0.318061000	-1.895008000	-0.127111000
6	-2.150043000	0.382750000	0.166524000
6	1.745437000	0.031096000	-0.296820000
8	-0.260503000	-0.109164000	-3.106755000
7	-3.016369000	1.267533000	0.710566000
7	-2.923955000	-0.471927000	-0.541805000
7	-1.451778000	-2.367292000	-0.694689000
7	0.407692000	-2.992801000	0.174046000
7	2.684706000	-0.827059000	0.130307000
7	2.356174000	0.848210000	-1.174307000
7	0.891555000	2.678041000	-0.803570000
7	-0.440990000	2.992631000	0.850988000
6	-4.320071000	0.966187000	0.339612000
1	-5.162069000	1.557827000	0.675465000
6	-4.263019000	-0.137811000	-0.450795000
1	-5.039484000	-0.705460000	-0.947549000
6	-2.405459000	-1.526629000	-1.394187000
1	-3.240515000	-2.146423000	-1.721532000
1	-1.893335000	-1.082811000	-2.256291000
6	-1.448160000	-3.749193000	-0.753847000
1	-2.264835000	-4.314972000	-1.183257000
6	-0.270019000	-4.144356000	-0.204989000
1	0.149510000	-5.130755000	-0.053902000
6	1.793958000	-3.102792000	0.653501000
1	2.384870000	-3.517099000	-0.171102000
1	1.799674000	-3.829996000	1.472482000
6	2.438011000	-1.829223000	1.165452000
1	1.820365000	-1.359268000	1.937184000
1	3.403357000	-2.099606000	1.601396000
6	3.890282000	-0.581501000	-0.508697000
1	4.778623000	-1.168591000	-0.314193000
6	3.684220000	0.485085000	-1.333906000
1	4.352113000	1.011834000	-2.003064000
6	1.701956000	1.989971000	-1.797478000
1	1.079093000	1.647630000	-2.627876000
1	2.466641000	2.674674000	-2.165747000
6	0.814975000	4.050361000	-0.620907000
1	1.369804000	4.749118000	-1.233983000
6	-0.036381000	4.248627000	0.427094000
1	-0.372004000	5.158041000	0.909222000
6	-1.333383000	2.697866000	1.968718000
1	-1.326613000	3.559189000	2.642155000
1	-0.923573000	1.830674000	2.496058000
6	-2.767957000	2.457291000	1.541906000
1	-3.372233000	2.353380000	2.449490000
1	-3.144635000	3.323370000	0.985170000
6	0.807842000	-0.545509000	-3.606570000
8	1.721813000	0.075082000	-4.168805000
6	1.063158000	-2.066176000	-3.423799000
9	1.710981000	-2.603464000	-4.457307000
9	1.838007000	-2.255603000	-2.326521000
9	-0.059591000	-2.769905000	-3.235169000

5a (*S* = 1)

26	-0.020823000	0.086439000	0.052960000
8	-0.034471000	0.042497000	1.724902000
6	0.268321000	2.065479000	-0.016393000
6	-0.211495000	-1.947317000	-0.092219000
6	-2.078591000	0.272179000	-0.029627000
6	1.966027000	0.063209000	-0.026599000
16	-0.181550000	0.190723000	-2.425658000
7	-2.963195000	1.195820000	0.438378000
7	-2.879496000	-0.713735000	-0.511539000
7	-1.336464000	-2.545231000	-0.565956000
7	0.558588000	-2.994999000	0.312409000
7	2.809538000	-0.719873000	0.673149000
7	2.773019000	0.942891000	-0.667891000
7	1.242244000	2.731048000	-0.685146000
7	-0.337593000	3.013236000	0.725411000
6	-4.277856000	0.791061000	0.231013000
1	-5.119494000	1.398042000	0.538562000
6	-4.225831000	-0.421418000	-0.370628000
1	-5.005802000	-1.095276000	-0.701156000
6	-2.408651000	-1.859142000	-1.248913000
1	-3.238134000	-2.556353000	-1.376429000
1	-2.047010000	-1.514608000	-2.222648000
6	-1.275793000	-3.927303000	-0.481607000
1	-2.082858000	-4.566962000	-0.814614000
6	-0.072777000	-4.211361000	0.072886000
1	0.388775000	-5.156545000	0.328113000
6	1.919561000	-3.022178000	0.861396000
1	2.620245000	-3.263845000	0.051704000
1	1.946861000	-3.851715000	1.574888000
6	2.376212000	-1.771608000	1.582015000
1	1.579529000	-1.369405000	2.214412000
1	3.232601000	-2.036096000	2.207750000
6	4.135027000	-0.366365000	0.450294000

1	4.959393000	-0.887694000	0.919936000
6	4.112166000	0.696636000	-0.398285000
1	4.909359000	1.293255000	-0.822769000
6	2.263168000	2.049288000	-1.455072000
1	1.819301000	1.667876000	-2.380133000
1	3.077359000	2.743207000	-1.667558000
6	1.236890000	4.085475000	-0.379849000
1	1.943361000	4.781125000	-0.814297000
6	0.225526000	4.265733000	0.510955000
1	-0.135498000	5.155030000	1.011597000
6	-1.397725000	2.740770000	1.684907000
1	-1.477764000	3.612172000	2.340344000
1	-1.103144000	1.870555000	2.278794000
6	-2.741524000	2.521633000	1.027191000
1	-3.516225000	2.654952000	1.789072000
1	-2.906379000	3.280467000	0.251493000
6	0.791712000	-1.025322000	-3.473235000
6	1.104568000	-0.282375000	-4.782608000
1	1.732234000	0.598095000	-4.598006000
1	1.641988000	-0.946508000	-5.475962000
1	0.183953000	0.051244000	-5.277549000
6	2.100907000	-1.473437000	-2.816997000
1	1.915329000	-1.958557000	-1.855467000
1	2.609271000	-2.198838000	-3.468540000
1	2.785901000	-0.636658000	-2.656040000
6	-0.058288000	-2.264117000	-3.790396000
1	0.470663000	-2.908144000	-4.508541000
1	-0.247793000	-2.863713000	-2.894188000
1	-1.020926000	-1.984249000	-4.235270000

5a (*S* = 2)

26	-0.035661000	0.043451000	-0.016152000
8	-0.111268000	-0.055990000	1.650938000
6	0.322731000	2.178031000	-0.010422000
6	-0.260370000	-2.073269000	-0.192304000
6	-2.246252000	0.318458000	-0.151471000
6	2.139882000	0.032915000	-0.035569000
16	-0.151863000	0.222311000	-2.464493000
7	-3.109416000	1.250412000	0.307641000
7	-3.027026000	-0.753815000	-0.431384000
7	-1.468207000	-2.626021000	-0.461690000
7	0.509450000	-3.107709000	0.213170000
7	2.946270000	-0.802828000	0.641205000
7	2.954373000	0.997976000	-0.510170000
7	1.409902000	2.817864000	-0.498228000
7	-0.336429000	3.111099000	0.700864000
6	-4.417370000	0.772212000	0.308260000
1	-5.253633000	1.374837000	0.639538000
6	-4.366310000	-0.502340000	-0.158391000
1	-5.144237000	-1.237950000	-0.320468000
6	-2.550533000	-1.927439000	-1.135573000
1	-3.382830000	-2.622649000	-1.252174000
1	-2.192158000	-1.609868000	-2.119471000
6	-1.460728000	-3.997448000	-0.240631000
1	-2.332200000	-4.618830000	-0.402961000
6	-0.207054000	-4.302000000	0.183948000
1	0.237010000	-5.247484000	0.468534000
6	1.947899000	-3.102498000	0.512997000
1	2.509928000	-3.132227000	-0.428037000
1	2.144017000	-4.042314000	1.035453000
6	2.456890000	-1.958704000	1.389445000
1	1.670230000	-1.618988000	2.070612000
1	3.295371000	-2.331372000	1.983101000
6	4.272569000	-0.387484000	0.573444000
1	5.079331000	-0.932477000	1.047658000
6	4.277675000	0.765202000	-0.154093000
1	5.086479000	1.425324000	-0.441411000
6	2.440192000	2.132383000	-1.260173000
1	1.995860000	1.768149000	-2.192449000
1	3.255131000	2.826630000	-1.469043000
6	1.433606000	4.150166000	-0.104619000
1	2.225371000	4.830451000	-0.392234000
6	0.316560000	4.338406000	0.651028000
1	-0.062148000	5.218998000	1.154617000
6	-1.536311000	2.839909000	1.489294000
1	-1.685426000	3.701887000	2.144626000
1	-1.345332000	1.958998000	2.110647000
6	-2.800989000	2.640717000	0.657126000
1	-3.654406000	3.005197000	1.235545000
1	-2.745230000	3.240283000	-0.259653000
6	0.816703000	-0.996316000	-3.502547000
6	0.835652000	-0.379274000	-4.911224000
1	1.348682000	0.590114000	-4.910960000
1	1.364774000	-1.048869000	-5.605200000
1	-0.182067000	-0.229804000	-5.292754000
6	2.253668000	-1.182138000	-3.009877000
1	2.274209000	-1.597476000	-1.999443000
1	2.786560000	-1.878708000	-3.673171000
1	2.805683000	-0.236701000	-3.001786000
6	0.115475000	-2.359590000	-3.557233000
1	0.642870000	-3.026240000	-4.255157000
1	0.111185000	-2.845577000	-2.577031000
1	-0.920281000	-2.260797000	-3.903304000

CHD

6	-3.297463303	0.029643438	1.723484309
6	-3.295251814	-1.311315502	2.409301053
6	-3.301686125	1.196625881	2.381066179
6	-3.305180064	1.303889502	3.881133925
6	-3.304161559	-0.033922923	4.567967423
6	-3.299647539	-1.201449471	3.911405248
1	-2.441237060	1.895520092	4.214827763
1	-4.175370372	1.891873693	4.210820296
1	-2.421658465	-1.900584636	2.078986926
1	-4.162721324	-1.906533882	2.073795270
1	-3.306819457	-0.025820739	5.659900750
1	-3.302528363	2.134168885	1.821461944
1	-3.295228116	0.024831618	0.631504465
1	-3.299038631	-2.137326615	4.473793322

^{3}**RC (3′)**

26	2.752548082	2.687365292	15.422587279
8	2.018555345	1.246669758	15.425198686
6	1.408027330	3.693534797	14.421037297
6	1.883699421	3.443799263	17.001811707
6	4.381291198	2.315064853	16.544658590
6	3.868810793	2.578413778	13.749575970
6	5.742490038	2.796128627	12.490576263
7	5.196821289	2.855180731	13.759583880
7	3.582443651	2.342210272	12.446816172
6	4.719624379	2.463653230	11.661356094
6	5.919384472	3.373796437	14.904578270

7	5.628154192	2.638710617	16.119869796
7	4.563418225	1.837435161	17.798166226
6	5.908172076	1.851389688	18.141770219
6	6.584178075	2.367935981	17.083091477
6	3.594124196	1.328015647	18.780347388
6	2.120501375	1.476827632	18.449302392
7	1.700995443	2.852006865	18.193544724
7	1.292698357	4.652706275	17.097859873
6	0.746842918	4.834591596	18.362011441
6	1.004325680	3.688380811	19.055087082
6	1.227771481	5.589628712	15.984609865
7	0.859300525	4.876117338	14.769523216
7	0.790772746	3.324847152	13.286843061
6	-0.144302534	4.276987196	12.904780304
6	-0.100212321	5.265256805	13.843929464
6	1.051598699	2.040603428	12.642426023
6	2.306158445	2.019132640	11.788168761
1	4.700319785	2.286323964	10.593583196
1	6.789740329	2.987965508	12.295874630
1	6.988833551	3.295609768	14.701796220
1	5.673343048	4.432419799	15.046514895
1	7.638171615	2.563330757	16.932202030
1	6.260652603	1.486820743	19.098148035
1	3.816276882	1.836478189	19.724866024
1	3.789308609	0.258609286	18.924248035
1	1.854606232	0.884640677	17.571280876
1	1.554984010	1.102650577	19.306890945
1	0.744918786	3.395628237	20.064574732
1	0.233827756	5.743757858	18.648438704
1	2.195757863	6.083709808	15.851408677
1	0.470303715	6.345219613	16.198098603
1	-0.654818058	6.190247502	13.935559700
1	-0.759327863	4.163989309	12.020983728
1	0.207922146	1.813900152	11.984913587
1	1.092792410	1.282291757	13.427413035
1	2.393577970	1.013703241	11.359893195
1	2.199647023	2.725042881	10.957480608
6	4.670319142	-0.771867959	13.131750430
6	3.739462465	-1.152519378	14.243914379
6	5.956622358	-0.454660993	13.319746856
6	6.618254124	-0.442758018	14.671527713
6	5.672265897	-0.787205852	15.790878105
6	4.385773425	-1.100779594	15.596939840
1	7.478045199	-1.135682480	14.676061154
1	7.072989177	0.545505169	14.855435192
1	3.327109645	-2.156062256	14.061977684
1	2.869500428	-0.486688117	14.246301102
1	4.254477131	-0.757827127	12.122627324
1	6.575400679	-0.180382235	12.464622295
1	3.748873571	-1.327578532	16.453217364
1	6.077527768	-0.765140833	16.803381587

^{3}TS$_σ$ (3′)

26	3.845539000	2.428350000	15.135882000
8	3.901142000	0.783566000	14.688479000
1	5.060134000	-0.041586000	14.544877000
6	2.522564000	3.042126000	13.834254000
6	2.330820000	2.338568000	16.366320000
6	5.033153000	2.625593000	16.779021000
6	5.242669000	3.394756000	14.011413000
6	7.057715000	4.693722000	13.589341000
7	6.199237000	4.191772000	14.552018000
7	5.503338000	3.414725000	12.681265000
6	6.620074000	4.192062000	12.405666000
6	6.192140000	4.644582000	15.928683000
7	6.024866000	3.548848000	16.862355000
7	5.103114000	1.952645000	17.953490000
6	6.138732000	2.435536000	18.741558000
6	6.719592000	3.453677000	18.055647000
6	4.285109000	0.848307000	18.478402000
6	3.113972000	0.388797000	17.631203000
7	2.144420000	1.443064000	17.349372000
7	1.226886000	3.113394000	16.362245000
6	0.342413000	2.716243000	17.357891000
6	0.926724000	1.654393000	17.982468000
6	1.031005000	4.199205000	15.413235000
7	1.402871000	3.752460000	14.078947000
7	2.513743000	2.789248000	12.515628000
6	1.394702000	3.350743000	11.914692000
6	0.688025000	3.965014000	12.906112000
6	3.550670000	1.984000000	11.876971000
6	4.815316000	2.758841000	11.558466000
1	7.003908000	4.309603000	11.400363000
1	7.884599000	5.350150000	13.828617000
1	7.144529000	5.134793000	16.137066000
1	5.386325000	5.373654000	16.070528000
1	7.545756000	4.107414000	18.304604000
1	6.370487000	2.008561000	19.708844000
1	3.929480000	1.166076000	19.465042000
1	4.942864000	-0.016440000	18.626036000
1	3.451631000	-0.007451000	16.670767000
1	2.603425000	-0.407445000	18.179568000
1	0.582687000	1.032086000	18.798906000
1	-0.604550000	3.212898000	17.525833000
1	1.639938000	5.062592000	15.701865000
1	-0.021008000	4.488001000	15.413194000
1	-0.241699000	4.519019000	12.884378000
1	1.196089000	3.254110000	10.854689000
1	3.154092000	1.599065000	10.933531000
1	3.760328000	1.141477000	12.539938000
1	5.520830000	2.058592000	11.095775000
1	4.596768000	3.543305000	10.825287000
6	7.659782000	0.477389000	16.139567000
6	6.700571000	-0.418314000	15.841618000
6	6.136404000	-0.553124000	14.483379000
1	5.880566000	-1.579190000	14.205103000
6	8.278873000	1.377919000	15.111213000
1	9.379620000	1.312580000	15.163632000
1	8.070315000	2.431572000	15.367557000
6	7.814001000	1.096332000	13.712483000
6	6.854644000	0.195069000	13.431367000
1	6.558023000	0.020420000	12.396647000
1	8.296431000	1.648645000	12.905770000
1	8.025256000	0.563862000	17.162946000

^{3}Int$_σ$ (3′)

26	4.212649000	2.641044000	15.150773000
8	4.313372000	0.788212000	15.331107000
1	5.212303000	0.426608000	15.347793000
6	7.096787000	-0.618495000	15.387499000
6	2.771983000	2.834794000	13.810759000
6	2.776992000	3.087500000	16.431288000

6	5.509302000	3.398303000	16.517290000
6	5.508584000	3.097364000	13.658923000
6	1.505349000	2.479026000	11.988072000
7	2.663704000	2.132068000	12.670362000
7	1.678046000	3.620841000	13.862015000
6	0.878640000	3.427123000	12.741881000
6	1.407999000	4.522910000	14.972461000
7	1.680151000	3.845280000	16.232071000
7	2.675228000	2.620565000	17.686971000
6	1.517564000	3.089510000	18.293259000
6	0.884607000	3.870217000	17.371202000
6	3.657779000	1.696121000	18.248589000
6	4.902906000	2.375554000	18.789624000
7	5.660814000	3.218477000	17.851310000
7	6.462011000	4.306614000	16.188481000
6	7.209723000	4.688745000	17.289109000
6	6.705553000	3.991697000	18.340211000
6	6.540042000	4.977536000	14.905525000
7	6.465913000	4.049627000	13.794037000
7	5.659969000	2.644397000	12.391432000
6	6.709693000	3.293583000	11.755137000
6	7.216593000	4.191110000	12.639773000
6	4.889309000	1.640690000	11.640673000
6	3.632476000	1.102048000	12.301058000
1	7.002215000	3.054379000	10.740841000
1	8.026198000	4.904780000	12.553653000
1	7.492078000	5.508384000	14.850421000
1	5.727972000	5.709538000	14.825456000
1	8.015633000	5.408965000	17.225913000
1	6.996696000	3.970666000	19.382636000
1	4.640815000	3.016962000	19.638098000
1	5.572570000	1.590117000	19.159986000
1	3.912431000	0.977741000	17.464785000
1	3.189575000	1.159433000	19.078136000
1	1.241403000	2.818082000	19.304312000
1	-0.043859000	4.424712000	17.420120000
1	2.033089000	5.418103000	14.887441000
1	0.357880000	4.817565000	14.945573000
1	-0.047345000	3.966230000	12.587879000
1	1.224455000	2.017401000	11.049967000
1	3.154703000	0.422206000	11.590344000
1	3.874898000	0.544509000	13.209597000
1	5.546130000	0.786286000	11.438099000
1	4.638421000	2.101878000	10.679212000
6	8.173968000	1.222782000	16.549296000
6	8.722297000	1.776813000	15.268664000
6	7.423469000	0.077169000	16.578976000
6	7.515128000	-0.106025000	14.133232000
6	8.270566000	1.033107000	14.047860000
1	6.529772000	-1.538891000	15.434995000
1	8.476437000	2.849390000	15.177515000
1	9.831345000	1.786992000	15.310796000
1	8.582066000	1.414387000	13.076658000
1	7.222835000	-0.631021000	13.223893000
1	7.060351000	-0.307508000	17.532020000
1	8.411033000	1.748875000	17.472778000

^{3}TS$_\pi$ (3′)

26	4.051619000	2.679794000	15.141869000
8	4.096844000	1.005736000	14.798800000
1	5.170696000	0.333718000	14.813395000
6	2.817462000	3.202928000	13.716218000
6	2.449511000	2.666054000	16.264809000
6	5.206179000	2.748978000	16.799392000
6	5.605669000	3.329618000	14.024061000
6	1.789381000	3.407991000	11.725308000
7	2.853817000	2.850160000	12.420235000
7	1.718574000	3.976750000	13.849135000
6	1.067129000	4.126933000	12.631834000
6	1.293912000	4.520831000	15.131306000
7	1.380886000	3.484145000	16.149984000
7	2.149545000	1.821211000	17.265263000
6	0.898315000	2.105779000	17.795177000
6	0.408490000	3.164528000	17.088726000
6	3.039399000	0.732712000	17.657221000
6	4.181173000	1.168378000	18.554299000
7	5.152970000	2.127736000	18.005007000
7	6.301453000	3.548066000	16.886647000
6	6.936568000	3.425687000	18.109035000
6	6.210820000	2.519013000	18.813369000
6	6.640560000	4.561708000	15.908602000
7	6.635242000	4.037535000	14.557947000
7	5.915566000	3.239209000	12.705641000
6	7.126821000	3.859968000	12.435310000
6	7.581473000	4.374255000	13.607189000
6	5.191939000	2.619128000	11.584156000
6	3.868936000	1.945060000	11.890133000
1	7.557392000	3.871872000	11.442215000
1	8.473315000	4.940203000	13.844013000
1	7.640179000	4.938618000	16.131302000
1	5.931981000	5.394579000	15.982234000
1	7.827820000	3.984778000	18.363937000
1	6.354739000	2.114066000	19.806800000
1	3.781245000	1.614204000	19.472497000
1	4.733816000	0.265752000	18.839774000
1	3.410356000	0.265028000	16.742533000
1	2.450368000	-0.001942000	18.213034000
1	0.467318000	1.532192000	18.605969000
1	-0.526098000	3.705151000	17.166299000
1	1.926160000	5.371373000	15.406264000
1	0.259583000	4.858432000	15.051608000
1	0.165500000	4.716089000	12.522386000
1	1.633811000	3.237475000	10.667456000
1	3.493089000	1.524735000	10.953261000
1	3.995382000	1.135053000	12.611992000
1	5.850096000	1.859836000	11.145560000
1	5.044041000	3.402623000	10.831981000
6	7.831517000	0.461110000	16.488997000
6	8.602254000	1.229526000	15.455596000
6	6.757344000	-0.294617000	16.180322000
6	6.241599000	-0.397050000	14.813997000
6	7.080039000	0.189900000	13.766643000
6	8.156428000	0.952596000	14.049523000
1	5.767760000	-1.346292000	14.560195000
1	9.683096000	1.029634000	15.558900000
1	8.521211000	2.312736000	15.662824000
1	8.173287000	0.527593000	17.521432000
1	6.232332000	-0.839971000	16.964964000
1	8.742305000	1.391068000	13.242343000
1	6.797548000	0.012679000	12.728704000

^{3}Int$_\pi$ (3′)

26	4.010213347	2.704531483	15.138690999
8	4.036788974	0.926435119	14.771887148
1	4.942692729	0.581804873	14.828147828
6	2.770504085	3.249471540	13.716557421
6	2.404556256	2.714343595	16.268768997
6	5.188163542	2.703336474	16.774042814
6	5.584179480	3.270967983	14.015844521
6	1.737218653	3.471835228	11.729804477
7	2.796857075	2.899101863	12.418527175
7	1.682319377	4.038123466	13.854626426
6	1.028409748	4.199593564	12.640982816
6	1.269212792	4.585782282	15.140007495
7	1.347535143	3.548870009	16.160363952
7	2.097676900	1.876043766	17.274044930
6	0.855159786	2.180907889	17.811225568
6	0.377307966	3.246921845	17.106143953
6	2.962402964	0.758555060	17.644044879
6	4.132849333	1.143571049	18.528688273
7	5.118973218	2.091781065	17.983770496
7	6.281421032	3.504221796	16.869714720
6	6.893113500	3.401446833	18.106181661
6	6.158867644	2.500072440	18.808001187
6	6.637744480	4.508892411	15.887916264
7	6.617382524	3.982018951	14.538205260
7	5.880558154	3.178790867	12.694639353
6	7.083098100	3.811053420	12.409036659
6	7.548362336	4.327929525	13.575338048
6	5.132357165	2.574923561	11.579392745
6	3.781345314	1.958326470	11.889302048
1	7.500765929	3.826881221	11.410533353
1	8.440031206	4.898319059	13.801971306
1	7.645835058	4.865407031	16.106408711
1	5.947501230	5.357131775	15.962692699
1	7.778025432	3.966488565	18.369870069
1	6.286245248	2.107836555	19.808777195
1	3.763731796	1.579225542	19.464255070
1	4.665032255	0.219183978	18.782185946
1	3.305798593	0.289878346	16.718741292
1	2.359831938	0.035987512	18.201300246
1	0.418905583	1.614910295	18.624599065
1	-0.548439063	3.801739207	17.189909192
1	1.912089254	5.429052859	15.412345834
1	0.238574988	4.935482983	15.064372963
1	0.134002836	4.800415553	12.535464613
1	1.572599708	3.304176367	10.672826491
1	3.390494814	1.551293690	10.952717324
1	3.877125180	1.145507096	12.613158015
1	5.757410647	1.783586601	11.148972113
1	5.018756199	3.356953855	10.820024199
6	7.945561603	0.486830744	16.523342500
6	8.706965876	1.278316043	15.502481272
6	7.000498237	-0.437460290	16.165702862
6	6.689825805	-0.682977924	14.804209558
6	7.350034842	0.051150684	13.786026937
6	8.304728893	0.985103650	14.088232103
1	5.969360746	-1.447176184	14.541258216
1	9.795591432	1.110101940	15.628021151
1	8.607803834	2.361145249	15.709026331
1	8.170703407	0.662382470	17.573794101
1	6.474454130	-0.999337210	16.937436972
1	8.803252282	1.537305255	13.293649053
1	7.089833463	-0.137555944	12.744573137

^{3}RC (3)

26	2.848952479	2.944448743	15.443776219
8	2.157990734	1.471863807	15.447684714
6	1.370538967	3.810689835	14.431738300
6	1.843763230	3.582973402	17.035229938
6	4.494757940	2.423454367	16.579878459
6	3.986414130	2.686813637	13.727911869
7	3.869748830	5.165666718	15.378417009
6	5.879445374	2.640585423	12.471024308
7	5.340222215	2.784423339	13.738752379
7	3.682844594	2.457295691	12.426721416
6	4.828555345	2.432554053	11.641432805
6	4.342341607	6.199036825	15.209482403
6	4.935702964	7.505005817	14.990815626
6	6.159064962	3.165438124	14.870304637
7	5.763208704	2.528530566	16.108018903
7	4.645832198	1.915952734	17.827204441
6	5.987592250	1.715158404	18.126264257
6	6.697316350	2.105758194	17.039732673
6	3.656246077	1.622752901	18.875422083
6	2.199787339	1.602406457	18.465911046
7	1.648305263	2.925606853	18.190990140
7	1.086034993	4.699726421	17.128558498
6	0.428215661	4.754967213	18.351348714
6	0.788812049	3.628933672	19.025809528
6	0.897863432	5.635917800	16.032080410
7	0.661950353	4.902891408	14.798360279
7	0.772520112	3.358888907	13.316519501
6	-0.299602861	4.168484072	12.962087973
6	-0.373032369	5.148493348	13.904115511
6	1.165938173	2.127603721	12.639283888
6	2.385765403	2.280820720	11.756512573
1	4.788939332	2.245382622	10.575999286
1	6.944742998	2.683509281	12.283589666
1	7.186763225	2.868787903	14.653672694
1	6.136829426	4.251356927	14.994189178
1	7.761993574	2.115571692	16.845246183
1	6.309264324	1.303808790	19.074297073
1	3.805536993	2.347083481	19.685407805
1	3.900175365	0.630676465	19.270357267
1	2.045231079	0.980330968	17.580459077
1	1.631476711	1.177243833	19.297360173
1	0.507759974	3.264662519	20.005588104
1	-0.226108235	5.573688407	18.622218219
1	1.771669268	6.281574648	15.929970685
1	0.023836402	6.253031078	16.244911533
1	-1.056773310	5.979563950	14.021218179
1	-0.911358767	3.969108913	12.091502464
1	0.333905271	1.824583773	11.998110925
1	1.318262306	1.358360683	13.400418116
1	2.464513287	1.375489673	11.144446937
1	2.247704629	3.128772981	11.075014074
1	6.006752812	7.392859979	14.786337360
1	4.799588943	8.126490984	15.883123434
1	4.451661063	7.986703272	14.133455367
6	4.616192129	-0.768143477	13.130169749
6	3.707162923	-1.032864240	14.292419680
6	5.936736279	-0.590011747	13.250294397
6	6.662513639	-0.635537673	14.568418872

6	5.743591146	-0.873871720	15.737088607
6	4.422543522	-1.045087182	15.610785687
1	7.448686070	-1.410648773	14.539585862
1	7.223168601	0.303636385	14.718093850
1	3.175405838	-1.984791868	14.144420189
1	2.919832069	-0.272569227	14.330271547
1	4.153835716	-0.713786196	12.143041903
1	6.537627862	-0.388884419	12.362460371
1	3.809660780	-1.197723035	16.500384371
1	6.199033426	-0.890657765	16.727574235

$^3TS_\pi$ (**3**)

26	2.775597321	3.223072430	14.957859891
8	1.722205982	1.841446311	14.966052446
6	1.304034688	4.327774595	14.217915427
6	2.019972536	3.806759218	16.698067939
6	4.362364076	2.258525885	15.822799342
6	3.580322630	2.829904123	13.116452646
7	3.938319215	4.875785543	14.980098425
6	5.237708007	2.418390136	11.608763328
7	4.913490945	2.640284896	12.936906796
7	3.068508527	2.721414981	11.863188299
6	4.066525962	2.470606256	10.929706961
6	4.635846435	5.786510995	14.996004783
6	5.520618218	6.933966532	15.020456686
6	5.928767829	2.789504692	13.954924740
7	5.568205636	2.157105651	15.204228299
7	4.560440137	1.642358689	17.016262195
6	5.862050900	1.170700066	17.131831680
6	6.503519616	1.496792709	15.984057127
6	3.661659863	1.449443967	18.163592870
6	2.183671278	1.668945983	17.927496185
7	1.815034144	3.074172464	17.807598636
7	1.465389920	5.012214249	16.971240001
6	0.919267384	5.039423340	18.249388109
6	1.148191674	3.809091505	18.782057721
6	1.321688235	6.060260617	15.974850006
7	0.822270431	5.481677376	14.739054729
7	0.482535794	4.044100194	13.190923804
6	-0.504358183	5.014047891	13.051165725
6	-0.293874200	5.925212615	14.038929317
6	0.579927161	2.830225806	12.389522887
6	1.692737806	2.869143958	11.365820853
1	3.848585766	2.335425221	9.878189515
1	6.252536072	2.239024737	11.278156919
1	6.844070905	2.319991269	13.590074509
1	6.140039483	3.849714251	14.120583324
1	7.517914068	1.316753785	15.652181599
1	6.202418150	0.633663103	18.007663830
1	4.009938572	2.098391038	18.976582985
1	3.792655312	0.412604258	18.491893112
1	1.842543084	1.153607207	17.026344607
1	1.649200837	1.262663519	18.790266677
1	0.894936460	3.392343409	19.748410696
1	0.425867615	5.914573170	18.652480146
1	2.274723640	6.564001221	15.805649732
1	0.599790885	6.793461575	16.337067734
1	-0.825511268	6.829555416	14.305762961
1	-1.260361624	4.962517919	12.277789047
1	-0.362301597	2.720434135	11.845882577
1	0.695556178	1.985072071	13.072518465
1	1.524015470	2.045555403	10.663662782
1	1.640433832	3.804052426	10.794217433
1	4.951905530	7.842109094	14.789146576
1	6.314387574	6.804277585	14.275629353
1	5.968189053	7.028908203	16.016436225
6	4.824930214	-1.167052299	14.855935341
6	5.331863262	-0.969787464	13.455360439
6	3.538389096	-0.959496315	15.199616818
6	2.530489025	-0.492255866	14.242123917
6	2.962981657	-0.444758162	12.842098191
6	4.243154912	-0.646860130	12.471338330
1	1.529999715	-0.904437618	14.396191987
1	2.213196815	0.712976610	14.588102700
1	5.885259043	-1.866546085	13.124422320
1	6.101604841	-0.175459613	13.441512060
1	3.220822513	-1.129723612	16.228771642
1	5.542450473	-1.498617715	15.605517479
1	4.520902304	-0.585866102	11.419632588
1	2.209826172	-0.225795258	12.084925601

$^3Int_\pi$ (**3**)

26	4.125580756	3.233688212	15.096566846
8	4.252430603	1.410310985	15.289319439
1	5.184281713	1.155525146	15.315904924
6	6.854275931	-0.352298914	15.388606492
6	2.644346639	2.977207268	13.801918399
6	2.638451912	3.249802975	16.409076824
6	5.577188897	3.593754137	16.497608001
6	5.579712164	3.310785602	13.647619274
7	3.941385211	5.194024397	14.878127670
6	1.334434915	2.175965253	12.144245852
7	2.586186030	2.139861004	12.749437343
7	1.411806194	3.536309098	13.866618307
6	0.584333568	3.058102143	12.857833343
6	0.990876399	4.386609694	14.967074942
7	1.406186730	3.782976776	16.221864080
7	2.568772132	2.640908286	17.607903774
6	1.311488882	2.796267513	18.181424573
6	0.569021795	3.516629256	17.298714275
6	3.652141232	1.855473418	18.188236669
6	4.768814027	2.696090471	18.767838416
7	5.710933069	3.308013734	17.818760096
7	6.723230541	4.259503998	16.195958403
6	7.557174510	4.395336040	17.293807050
6	6.914393890	3.789071010	18.321111714
6	7.000710339	4.902630492	14.931374456
7	6.712983521	4.044991182	13.803154299
7	5.704615113	2.791632986	12.398322601
6	6.886229057	3.204247738	11.793961758
6	7.527115560	4.000843683	12.683135493
6	4.788928652	1.956603994	11.606768104
6	3.676477349	1.255208725	12.354166649
6	3.848412689	6.327787076	14.729611808
6	3.726146476	7.758430890	14.535338759
1	7.166030467	2.880557113	10.799898475
1	8.475816116	4.519460191	12.630749038
1	8.061396307	5.158320113	14.904435457
1	6.428856254	5.831103740	14.851353932
1	8.516863028	4.892602273	17.238749922
1	7.204656575	3.643813489	19.353615731
1	4.348791145	3.496608685	19.389515415

1	5.364471393	2.050747819	19.421858722
1	4.023933243	1.175772302	17.417345649
1	3.224979236	1.268093380	19.005292829
1	1.061726088	2.378323300	19.148164721
1	-0.458200584	3.855836018	17.334762283
1	1.412382453	5.387881745	14.863368969
1	-0.097238693	4.464271645	14.956334706
1	-0.442591316	3.381201138	12.745162855
1	1.092703603	1.571389750	11.279380350
1	3.256685353	0.498657053	11.685968490
1	4.052245050	0.765290707	13.256069804
1	5.403886240	1.184691214	11.131675620
1	4.369919952	2.580175705	10.807697675
1	4.697221056	8.178070682	14.248127988
1	3.389562661	8.227633174	15.466995128
6	8.383041881	1.143146122	16.542189157
6	9.000993549	1.593558509	15.251395674
6	7.372438073	0.219124052	16.577754899
6	7.371134741	0.048057316	14.130879218
6	8.382723123	0.966456778	14.037272679
1	6.067381862	-1.093581555	15.440988933
1	8.960207082	2.697170393	15.173397949
1	10.091841317	1.394808121	15.265343185
1	2.996026504	7.958394678	13.742257836
1	8.767464172	1.260362323	13.062634311
1	6.951370231	-0.387010022	13.223710983
1	6.953209193	-0.086093382	17.536665123
1	8.769023723	1.570227466	17.465871673

$^{3}\mathrm{TS}_{\sigma}$ (**3**)

26	3.839453982	2.473094976	15.154215720
8	3.907841578	0.830524163	14.703247145
1	5.050461326	-0.004761716	14.558509220
6	2.512774666	3.065765418	13.843377874
6	2.314961069	2.355538664	16.373377476
6	5.036169357	2.650030298	16.794266375
6	5.256192503	3.410486248	14.024851954
7	3.513574014	6.326393019	15.055391748
6	7.087443201	4.685272460	13.594184748
7	6.226123008	4.195311084	14.560288337
1	3.330411937	7.319032971	12.070609127
7	5.514994832	3.423369400	12.693106293
6	6.641480318	4.186295075	12.412351551
6	3.582000272	6.336823706	13.904857021
6	3.669543583	6.349616048	12.452998425
6	6.241124035	4.641403983	15.939655785
7	6.053392666	3.546237667	16.869056097
7	5.108026163	1.965845776	17.962359498
6	6.166723835	2.416481483	18.738035652
6	6.763327772	3.424257575	18.050367998
6	4.267659975	0.883078659	18.497489792
6	3.116092276	0.406858078	17.635083916
7	2.132969408	1.446142861	17.344913395
7	1.190436448	3.100663123	16.356104731
6	0.298686789	2.671608534	17.331101760
6	0.899027838	1.619730642	17.957022631
6	0.975625659	4.184555137	15.410229903
7	1.376241822	3.754309019	14.078087002
7	2.515235787	2.807576521	12.524469259
6	1.386799593	3.342787505	11.915171481
6	0.663599711	3.947523985	12.900929652
6	3.555758007	1.998209658	11.894767862
6	4.821783617	2.767970845	11.572930272
1	7.024694071	4.295922261	11.405930055
1	7.923855224	5.330690677	13.829958914
1	7.207201694	5.106417270	16.141846650
1	5.451691571	5.386698921	16.085472887
1	7.610042249	4.054372368	18.290949720
1	6.401807997	1.976589988	19.698712357
1	3.889567782	1.228158751	19.466691398
1	4.917216556	0.019849798	18.683697810
1	3.470817677	0.015832686	16.678608464
1	2.610364408	-0.396956009	18.176752924
1	0.557111065	0.981675700	18.762108499
1	-0.663517793	3.142532055	17.485983225
1	1.560132901	5.061614534	15.706187542
1	-0.084022975	4.442602715	15.396924275
1	-0.276324602	4.483616485	12.871849031
1	1.195217472	3.237636492	10.854719069
1	3.160932817	1.607161848	10.953242572
1	3.763057627	1.160909361	12.564880404
1	5.524268666	2.063936384	11.111581693
1	4.608530761	3.548834708	10.833854354
1	3.034763790	5.564600612	12.029039878
1	4.707487233	6.189064959	12.141890346
6	7.673570089	0.492834714	16.123079759
6	6.697567460	-0.391196680	15.845292089
6	6.120253348	-0.534799666	14.494063198
1	5.844205699	-1.559489586	14.230797271
6	8.296638117	1.371008529	15.078004518
1	9.396660816	1.287680825	15.119763506
1	8.109374100	2.430914027	15.324808217
6	7.812988668	1.081214295	13.687283562
6	6.838398286	0.190089676	13.426364935
1	6.529332166	0.007219906	12.396718745
1	8.295492895	1.616587803	12.869249537
1	8.049946757	0.585918741	17.141746647
1	6.281530747	-1.010684022	16.640241269

$^{3}\mathrm{Int}_{\sigma}$ (**3**)

26	3.805603035	2.415808313	15.148108977
8	3.904212451	0.616904394	14.664950113
1	4.801444582	0.257749180	14.592165619
6	2.462063043	3.095375774	13.861340524
6	2.271232035	2.399367483	16.394681100
6	5.008733797	2.567763894	16.772943014
6	5.222289780	3.320528437	14.011158699
7	3.565153334	6.338412348	15.024651314
6	7.019877499	4.631889127	13.560896258
7	6.184452858	4.119054644	14.538144116
1	3.362614362	7.321982213	12.036290186
7	5.460313889	3.342326779	12.676408651
6	6.566201402	4.130215477	12.382651924
6	3.615376449	6.343009240	13.873199688
6	3.681804260	6.346431018	12.420099384
6	6.211130616	4.565021740	15.918109206
7	6.013893646	3.476035261	16.854091639
7	5.058241373	1.904048002	17.952863728
6	6.088878476	2.384411845	18.748886722
6	6.691650313	3.386089020	18.057655977
6	4.205532888	0.827109845	18.481288288
6	3.012595854	0.416239877	17.639079969

7	2.071737982	1.502484625	17.375490482
7	1.176983765	3.187274779	16.392829862
6	0.286415451	2.797813747	17.385188209
6	0.855922489	1.725977247	18.006631396
6	0.983626729	4.271874520	15.442134891
7	1.353584467	3.823675898	14.106217730
7	2.444472347	2.839182289	12.541183231
6	1.328646339	3.411952005	11.943197257
6	0.636842856	4.041925507	12.936173503
6	3.444582352	1.981718003	11.906532200
6	4.745039223	2.687739658	11.568701768
1	6.933063137	4.251193813	11.371219095
1	7.848139038	5.291281287	13.786875255
1	7.184249902	5.017887701	16.114986819
1	5.433647107	5.322821061	16.065914248
1	7.525056590	4.029825071	18.308410912
1	6.303371644	1.965897394	19.723834816
1	3.869328483	1.155153340	19.471130565
1	4.831968623	-0.061871920	18.621457758
1	3.330381649	0.017679760	16.671639223
1	2.481886358	-0.369082057	18.184069782
1	0.501027230	1.104052868	18.818713511
1	-0.654932818	3.305030302	17.553088755
1	1.597508350	5.133117456	15.724750533
1	-0.068026776	4.561544446	15.441425619
1	-0.284743115	4.609467062	12.915080753
1	1.120490062	3.311409629	10.885373727
1	3.025502372	1.603377918	10.970340373
1	3.619218415	1.137643675	12.578974574
1	5.416304337	1.943268941	11.124462007
1	4.567816146	3.460630410	10.812412164
1	3.020266130	5.575800068	12.010857102
1	4.711612551	6.158276491	12.097903376
6	7.745246045	0.539040109	16.140191351
6	6.959746256	-0.512698362	15.748766939
6	6.685372312	-0.755685437	14.379066062
1	6.094620301	-1.614389706	14.086540074
6	8.383919540	1.462812967	15.146999369
1	9.488220383	1.410574269	15.243950383
1	8.172517159	2.514939766	15.409193018
6	7.988298287	1.188413887	13.727074581
6	7.195185929	0.123612023	13.390155212
1	6.947912020	-0.055974165	12.343789353
1	8.373280440	1.852608106	12.955100887
1	7.942941106	0.714291367	17.196551194
1	6.528370074	-1.174679885	16.499523976

^{3}RC (4)

26	2.828072910	3.071522150	15.312685296
8	1.984420757	1.736582704	14.901025134
6	1.862660102	4.227145258	14.035038391
6	1.465109509	3.725896892	16.597601795
6	3.916670805	2.177887938	16.822974473
6	4.405778675	2.774175455	14.028256608
6	6.588746265	2.581338559	13.431032288
7	5.682734167	2.664242184	14.475844250
7	4.522781096	2.723587588	12.678751450
6	5.851971648	2.615978719	12.294305035
6	6.134305394	2.779610034	15.850542821
7	5.269501600	2.115049907	16.799663358
7	3.576956488	1.525415547	17.964953458
6	4.706146569	1.080743182	18.642004328
6	5.779422429	1.455262405	17.903815082
6	2.268303478	1.310053464	18.599427885
6	1.040521424	1.592721095	17.762153396
7	0.807230277	3.013649597	17.529115409
7	0.892589084	4.949538859	16.616464064
6	-0.114089889	5.015918538	17.571381139
6	-0.164490957	3.785957422	18.153525403
6	1.211983107	5.981690096	15.644626754
7	1.244124528	5.395098969	14.313155163
7	1.605887949	3.987192756	12.739542045
6	0.861700478	5.019241405	12.180596591
6	0.628780892	5.913604575	13.180868662
6	2.056765885	2.785614017	12.047549455
6	3.509420171	2.843919845	11.622356160
1	6.146150627	2.555934474	11.254547772
1	7.655315994	2.493148359	13.591713389
1	7.118477074	2.310726222	15.911479470
1	6.231161281	3.837222502	16.115535045
1	6.840699406	1.307016305	18.055027444
1	4.638788872	0.532315067	19.572447129
1	2.239103554	1.913907001	19.514684083
1	2.228913641	0.256778564	18.897489101
1	1.102114182	1.096710793	16.790077897
1	0.175167711	1.201636510	18.303904494
1	-0.800619625	3.393731658	18.936843686
1	-0.693240732	5.914003823	17.743941446
1	2.181521546	6.421609910	15.875137881
1	0.435955275	6.748002093	15.674055444
1	0.086154955	6.850077266	13.191599729
1	0.558250782	5.017486818	11.141473319
1	1.451651618	2.677568108	11.143369859
1	1.869724141	1.929389562	12.701179328
1	3.683309910	2.021482962	10.919427459
1	3.700301189	3.781980903	11.087544783
6	5.923416985	-1.056592019	15.651216708
6	6.725345110	-0.846595491	14.394484725
6	4.586345398	-1.104695103	15.673304716
6	3.730119518	-0.946847667	14.452764140
6	4.524945622	-0.690744724	13.206705172
6	5.862288626	-0.647606774	13.176733112
1	3.099150551	-1.838693600	14.317384453
1	3.022566792	-0.125266022	14.615194939
1	7.409272180	-1.699675201	14.236950501
1	7.401337420	0.017158224	14.521183802
1	6.482400478	-1.161025788	16.582324491
1	4.069508003	-1.245567208	16.623761876
1	6.376162968	-0.449851529	12.235254155
1	3.960610639	-0.530492880	12.286205710
8	4.036035802	5.033855383	15.841481637
6	4.943401344	5.714308690	15.272462058
8	6.111940836	5.864886044	15.631675374
6	4.520531941	6.463173206	13.976119563
9	5.517786102	7.172856976	13.461085559
9	4.110904582	5.598898428	13.026179734
9	3.498213394	7.301777197	14.215035216

$^{3}TS_{\pi}$ (4)

26	2.996952092	3.061147903	15.272044223
8	2.152445166	1.625820027	14.821244207
6	1.923562533	4.139121991	14.007113312

6	1.573686323	3.577425159	16.561039329
6	4.139615191	2.174232065	16.758031440
6	4.570306058	2.825909812	13.966834873
6	6.746710105	2.609717132	13.340675445
7	5.855777006	2.723949768	14.394537190
7	4.671043669	2.765057015	12.614586454
6	5.993672123	2.635081115	12.213237026
6	6.307812876	2.879748465	15.763640871
7	5.494994659	2.150001916	16.711306156
7	3.844108083	1.504615839	17.904256509
6	4.997002256	1.079762127	18.550648788
6	6.045826667	1.489505359	17.795253197
6	2.554662622	1.233652459	18.555735984
6	1.306765226	1.413412739	17.718777237
7	0.961735982	2.810052679	17.482404908
7	0.912961857	4.757556169	16.596861302
6	-0.103493135	4.738987724	17.542855751
6	-0.068227531	3.501239931	18.109271475
6	1.168751727	5.821384806	15.641116551
7	1.222156094	5.256696690	14.301925903
7	1.666072033	3.896287910	12.710098395
6	0.835973858	4.873152948	12.172835288
6	0.550543851	5.737442878	13.185528082
6	2.198815278	2.746877999	11.989565467
6	3.646629181	2.908896656	11.571487920
1	6.273753237	2.560345005	11.170390942
1	7.814809177	2.514421446	13.489083650
1	7.326137553	2.491423543	15.823545379
1	6.321027733	3.942831186	16.024007526
1	7.113857169	1.368342453	17.923701654
1	4.963757398	0.515904560	19.473688487
1	2.492315587	1.861343092	19.453358467
1	2.582949092	0.189674118	18.885933329
1	1.406688558	0.922662716	16.747374948
1	0.475403963	0.956483913	18.262392283
1	-0.678173179	3.054267584	18.884025258
1	-0.746746735	5.590948134	17.722842663
1	2.117656086	6.304626226	15.871028061
1	0.355505665	6.547145225	15.688629448
1	-0.061655528	6.629768652	13.213694205
1	0.519104738	4.858581729	11.137785772
1	1.605708099	2.626640223	11.078991020
1	2.063226885	1.862968367	12.617851460
1	3.859462813	2.145271562	10.815543792
1	3.787277665	3.888632270	11.099340984
6	5.229618949	-1.139453770	15.885821912
6	6.224507624	-0.822133738	14.804908973
6	3.897394161	-1.058069352	15.694208845
6	3.311219124	-0.626518611	14.423153250
6	4.248764369	-0.482311033	13.307610462
6	5.585336280	-0.556976527	13.470605913
1	2.359312955	-1.097765999	14.169112080
1	2.805168449	0.561973214	14.641814349
1	6.964368379	-1.637395092	14.717197563
1	6.835768198	0.049255106	15.105630492
1	5.623601078	-1.443181223	16.855124432
1	3.217802626	-1.302222891	16.511181656
1	6.247525970	-0.421739205	12.616007866
1	3.835009678	-0.290488728	12.317011661
8	4.077536123	5.083284924	15.808640095
6	4.944011622	5.788399860	15.207923205
8	6.126629877	5.956786731	15.509515630
6	4.441364040	6.543887568	13.944528927
9	5.374518906	7.338176889	13.430943060
9	4.076858298	5.676424835	12.978370304
9	3.367686466	7.300418758	14.224689708

^{3}Int$_{\pi}$ (4)

26	3.177225985	3.064216496	15.326559483
8	2.365998686	1.480871275	14.873597889
6	2.045960387	4.030034111	14.019280717
6	1.686127003	3.505917865	16.570922798
6	4.336682568	2.192215776	16.822426383
6	4.767606016	2.815024393	14.025660938
6	6.951776131	2.628859055	13.409716012
7	6.054759739	2.752431268	14.458164688
7	4.873402145	2.711754371	12.676298619
6	6.200453129	2.602589131	12.280655835
6	6.487849984	2.938793292	15.830489546
7	5.693198910	2.177212405	16.770315565
7	4.049362894	1.495745990	17.953994464
6	5.207805803	1.062551946	18.587423395
6	6.252462364	1.495655728	17.838063109
6	2.762381560	1.257467960	18.624302612
6	1.512704082	1.349241227	17.772640634
7	1.094642830	2.718471171	17.490454451
7	0.955738862	4.645803462	16.570684379
6	-0.085610678	4.581572417	17.486479442
6	0.007358579	3.357345065	18.075439277
6	1.179062049	5.702846137	15.598709568
7	1.276299771	5.112932431	14.273024433
7	1.805461203	3.726434723	12.730653363
6	0.913049299	4.627837047	12.161593246
6	0.572789529	5.508244165	13.143274262
6	2.405280004	2.585814681	12.047754498
6	3.845494193	2.811257948	11.630614096
1	6.484754477	2.507079478	11.240562611
1	8.021546357	2.562619114	13.561723369
1	7.521696928	2.596423870	15.905266159
1	6.448413138	4.003051762	16.083185213
1	7.321792001	1.379407945	17.960980805
1	5.181277689	0.485028704	19.502312846
1	2.687416556	1.950525062	19.471723693
1	2.810508847	0.243168538	19.033632017
1	1.647075792	0.834955237	16.817228578
1	0.700638343	0.868592900	18.324853450
1	-0.596491223	2.887645041	18.841506202
1	-0.783507033	5.396087197	17.633794454
1	2.104325959	6.229295480	15.831306731
1	0.335939381	6.395044294	15.621276170
1	-0.096648753	6.359048348	13.141022720
1	0.596514306	4.555554404	11.128828700
1	1.824304579	2.406728146	11.138937563
1	2.315053835	1.716049849	12.704063350
1	4.093768938	2.054920753	10.878528895
1	3.945846096	3.794714389	11.155810052
6	4.855994535	-1.223236591	15.729356606
6	5.864039723	-0.786972253	14.707187057
6	3.579079121	-1.578575870	15.380276154
6	3.134910255	-1.508872743	14.035704047
6	4.009589115	-1.025185690	13.031362866
6	5.299186066	-0.664477301	13.322369133

1	2.119977735	-1.787318264	13.784396128
1	3.024913248	0.777265906	14.820848024
1	6.722540299	-1.490677864	14.702816479
1	6.331254485	0.164461420	15.013822255
1	5.173952129	-1.267155523	16.769949357
1	2.881311931	-1.904840445	16.151413534
1	5.954785028	-0.286936396	12.539662825
1	3.640821520	-0.930204107	12.009908061
8	4.160578520	5.113446837	15.842761671
6	5.014298421	5.818932669	15.221973221
8	6.193729478	6.013130051	15.516909679
6	4.495556338	6.533038464	13.941281377
9	5.389436058	7.382507237	13.445588877
9	4.215711949	5.634075567	12.974944244
9	3.368918926	7.220064294	14.184863943

^{3}TS$_\sigma$ (4)

26	3.777377000	2.797757000	15.191525000
8	3.947100000	1.111083000	14.784818000
1	4.946449000	0.333783000	14.680073000
6	2.422356000	3.167926000	13.801665000
6	2.194096000	2.566224000	16.359626000
6	5.038804000	2.794325000	16.825068000
6	5.306326000	3.526688000	14.018581000
6	7.321946000	4.481344000	13.572191000
7	6.396382000	4.142765000	14.545063000
7	5.569072000	3.477855000	12.688751000
6	6.796868000	4.055798000	12.396418000
6	6.545091000	4.559076000	15.926125000
7	6.186749000	3.516533000	16.863043000
7	5.066689000	2.080486000	17.981579000
6	6.210742000	2.358032000	18.716588000
6	6.921675000	3.271117000	18.009526000
6	4.092230000	1.145700000	18.560755000
6	3.009252000	0.624205000	17.643046000
7	2.005740000	1.625513000	17.302208000
7	1.017290000	3.224139000	16.276802000
6	0.092461000	2.712366000	17.176770000
6	0.723233000	1.697630000	17.830684000
6	0.750913000	4.235442000	15.268508000
7	1.220106000	3.756925000	13.978183000
7	2.454855000	2.809563000	12.508382000
6	1.294822000	3.202196000	11.852655000
6	0.507874000	3.803517000	12.787564000
6	3.577021000	2.103382000	11.903823000
6	4.742624000	3.010719000	11.568242000
1	7.184346000	4.102655000	11.386814000
1	8.254838000	4.979191000	13.802966000
1	7.595415000	4.809018000	16.087483000
1	5.937524000	5.451913000	16.104261000
1	7.868360000	3.755790000	18.210824000
1	6.416579000	1.875855000	19.663424000
1	3.639242000	1.632218000	19.433662000
1	4.659704000	0.281701000	18.923329000
1	3.431436000	0.244634000	16.708753000
1	2.499130000	-0.191540000	18.162209000
1	0.377950000	1.025673000	18.606068000
1	-0.910846000	3.108567000	17.266761000
1	1.269970000	5.158671000	15.524329000
1	-0.325225000	4.404998000	15.213285000
1	-0.476947000	4.246650000	12.716485000
1	1.132149000	3.010933000	10.799616000
1	3.222930000	1.657873000	10.970217000
1	3.877775000	1.305136000	12.587624000
1	5.408847000	2.461965000	10.894268000
1	4.380574000	3.894709000	11.029354000
6	7.643322000	0.345505000	16.222728000
6	6.568118000	-0.407339000	15.915416000
6	5.980032000	-0.422403000	14.571430000
1	5.500523000	-1.358073000	14.280458000
6	8.353263000	1.195124000	15.206983000
1	9.442100000	1.017915000	15.253774000
1	8.251842000	2.260929000	15.480983000
6	7.852295000	0.989349000	13.805553000
6	6.774998000	0.229703000	13.524415000
1	6.447272000	0.116112000	12.490438000
1	8.394442000	1.487179000	13.001974000
1	8.029194000	0.355387000	17.241705000
1	6.085413000	-1.005545000	16.689201000
8	3.509367000	5.238081000	15.654047000
6	3.917375000	6.276087000	15.060775000
8	4.816355000	7.053561000	15.399376000
6	3.196191000	6.603743000	13.722809000
9	3.592951000	7.760475000	13.202134000
9	3.438142000	5.644580000	12.803306000
9	1.863528000	6.665316000	13.885951000

^{3}Int$_\sigma$ (4)

26	3.847002796	2.486657775	15.170169941
8	3.934670213	0.669364073	14.684143133
1	4.832619057	0.310930484	14.636243728
6	2.501824018	3.090037183	13.840868150
6	2.275880302	2.446624413	16.387638600
6	5.064426709	2.536739206	16.805047127
6	5.318522182	3.335864876	14.043576182
6	7.224136492	4.492420691	13.620579684
7	6.354134803	4.023577729	14.586824936
7	5.563331924	3.363292346	12.710071334
6	6.727028613	4.065694470	12.432305144
6	6.457180991	4.427301264	15.974619717
7	6.145481965	3.350742778	16.893685859
7	5.070611434	1.847390823	17.975291895
6	6.141323928	2.227568407	18.770819621
6	6.820693773	3.184951007	18.089177771
6	4.140864448	0.837526872	18.505720185
6	2.977125993	0.438747771	17.623045330
7	2.047817187	1.529939915	17.345274569
7	1.168512303	3.215995079	16.354527678
6	0.243967783	2.796659096	17.300921239
6	0.804304929	1.724708478	17.930308693
6	0.978567270	4.270697845	15.368851110
7	1.355350940	3.765138010	14.057235840
7	2.491032786	2.772780827	12.535735413
6	1.353211808	3.269526625	11.915450892
6	0.630171359	3.901734443	12.882910842
6	3.560265851	2.008828009	11.902774198
6	4.782204418	2.843662309	11.577184086
1	7.095852403	4.188508549	11.422189223
1	8.101899774	5.077029754	13.862736357
1	7.485819478	4.742475578	16.158693446
1	5.788256486	5.274863858	16.146831356
1	7.709067996	3.750069266	18.340440603

1	6.326639704	1.777000712	19.737333758
1	3.769231684	1.220568321	19.463327837
1	4.721412256	-0.068721829	18.713903546
1	3.325653031	0.050674672	16.661446398
1	2.425616125	-0.349231417	18.143051297
1	0.428779092	1.086145413	18.719880575
1	-0.711901033	3.286224482	17.436228071
1	1.617482854	5.125207664	15.611051299
1	-0.072887800	4.559970015	15.353158273
1	-0.315545637	4.426218376	12.841269367
1	1.156757733	3.121902231	10.861072514
1	3.175512124	1.607243578	10.961274397
1	3.809717211	1.175632974	12.566121511
1	5.456677565	2.224372970	10.974879317
1	4.488543756	3.706371088	10.968223648
6	7.841204859	0.407368753	16.205323268
6	6.991195674	-0.598035972	15.828647971
6	6.677428692	-0.823592587	14.464337332
1	6.031122635	-1.646003775	14.184857201
6	8.503246665	1.303151340	15.201226036
1	9.605703677	1.204637255	15.279048002
1	8.338551741	2.363297527	15.464999856
6	8.070298463	1.048655020	13.788179769
6	7.215098076	0.028382480	13.466532926
1	6.935501003	-0.133856713	12.425545998
1	8.468972998	1.697187877	13.009914517
1	8.066634728	0.569455317	17.257909288
1	6.539097797	-1.236511551	16.587597307
8	3.602679856	5.792452169	15.694339367
6	4.146210019	6.452553268	14.771295602
8	5.281253796	6.947220795	14.723241909
6	3.294349424	6.611981502	13.481166656
9	3.618876231	7.699533319	12.783115687
9	3.500333137	5.544695863	12.671838758
9	1.978216454	6.664595058	13.730716856

^{3}RC (5a)

26	2.786207000	3.114889000	15.337890000
8	2.086427000	1.599603000	15.337244000
6	1.247618000	3.905222000	14.377752000
6	1.748058000	3.641471000	16.953974000
6	4.435401000	2.544541000	16.444241000
6	3.870599000	2.755997000	13.635239000
6	5.737425000	2.515539000	12.350141000
7	5.228492000	2.773110000	13.611669000
7	3.535361000	2.441808000	12.354635000
6	4.662409000	2.307301000	11.553129000
6	6.071350000	3.212330000	14.697669000
7	5.701922000	2.615474000	15.957127000
7	4.606646000	2.044360000	17.698418000
6	5.951318000	1.839157000	17.984082000
6	6.646532000	2.200919000	16.879734000
6	3.631573000	1.732617000	18.750053000
6	2.187753000	1.623974000	18.315820000
7	1.563628000	2.917589000	18.076210000
7	0.904535000	4.692990000	17.085919000
6	0.209843000	4.643035000	18.287318000
6	0.635258000	3.516437000	18.919818000
6	0.686456000	5.654744000	16.025221000
7	0.474864000	4.946005000	14.775023000
7	0.625447000	3.414915000	13.287165000
6	-0.511959000	4.153179000	12.978244000
6	-0.612530000	5.122249000	13.927480000
6	1.026459000	2.186451000	12.611409000
6	2.223834000	2.356585000	11.704018000
1	4.592864000	2.055495000	10.502926000
1	6.799923000	2.488601000	12.146673000
1	7.099788000	2.927636000	14.468448000
1	5.987326000	4.301469000	14.793578000
1	7.706832000	2.187636000	16.663083000
1	6.281458000	1.433982000	18.931810000
1	3.727594000	2.484392000	19.543908000
1	3.926889000	0.766683000	19.173958000
1	2.094956000	1.031438000	17.400592000
1	1.630920000	1.134570000	19.119520000
1	0.361519000	3.087526000	19.875121000
1	-0.510137000	5.398887000	18.574295000
1	1.570784000	6.290325000	15.942263000
1	-0.201426000	6.246499000	16.252629000
1	-1.344712000	5.905356000	14.077996000
1	-1.141375000	3.917199000	12.129804000
1	0.183142000	1.865198000	11.994222000
1	1.221680000	1.430703000	13.378049000
1	2.261050000	1.490469000	11.034435000
1	2.093617000	3.248970000	11.077801000
6	4.482138000	-0.797422000	13.360172000
6	3.821614000	-0.945550000	14.697372000
6	5.802580000	-0.654418000	13.197867000
6	6.779967000	-0.628475000	14.343153000
6	6.120276000	-0.840795000	15.680215000
6	4.798190000	-0.979323000	15.834315000
1	7.570707000	-1.382500000	14.184680000
1	7.326064000	0.332533000	14.347696000
1	3.194148000	-1.849488000	14.711314000
1	3.125667000	-0.110223000	14.851695000
1	3.829278000	-0.785762000	12.485556000
1	6.213467000	-0.528570000	12.195239000
1	4.384051000	-1.109393000	16.835868000
1	6.771437000	-0.858589000	16.554712000
1	5.370483000	7.925762000	13.224051000
6	3.911764000	6.502692000	13.991308000
6	5.274209000	7.212012000	14.056024000
1	6.099832000	6.493334000	13.985132000
1	5.386055000	7.768535000	14.995002000
6	3.794980000	5.744077000	12.666795000
1	3.822754000	6.459052000	11.831451000
1	4.620461000	5.041155000	12.525912000
6	2.796677000	7.557232000	14.060027000
1	2.934482000	8.300957000	13.261129000
1	1.808843000	7.106321000	13.916282000
1	2.804839000	8.087751000	15.020066000
1	2.851022000	5.194802000	12.605885000
16	3.842545000	5.379544000	15.496217000

^{3}TS$_\pi$ (5a)

26	2.865723296	3.238340601	14.894318389
8	1.772784039	1.867410828	14.925512095
6	1.414995010	4.358925426	14.154678032
6	2.103524890	3.836655165	16.637858859
6	4.451630411	2.253353150	15.777937477
6	3.654214513	2.748256576	13.068126887
16	4.372737427	5.202233953	15.021400037

6	5.271954560	2.166082213	11.568543341
7	4.972225011	2.471290145	12.884600810
7	3.124772150	2.589278432	11.823498336
6	4.098275412	2.242378888	10.895961469
6	5.998986537	2.660744223	13.880630909
7	5.643097506	2.072703168	15.147855553
7	4.646675984	1.662075627	16.989438678
6	5.930181391	1.142903024	17.102892837
6	6.564032911	1.403856246	15.934593421
6	3.752482171	1.521712089	18.144179019
6	2.274374052	1.699887160	17.879974510
7	1.881122947	3.094443189	17.743396060
7	1.506910270	5.023603120	16.907161838
6	0.925240174	5.033619676	18.167661133
6	1.170082554	3.807316539	18.702160526
6	1.402843323	6.078588268	15.920961764
7	0.924571179	5.513671489	14.671808046
7	0.593676020	4.078257555	13.120873713
6	-0.385996877	5.053949073	12.969432388
6	-0.181061574	5.962900681	13.960063401
6	0.655403331	2.845245644	12.346472768
6	1.767130190	2.825391446	11.322484171
1	3.862615679	2.065205774	9.854658360
1	6.273085986	1.915076437	11.243088497
1	6.916966824	2.189925771	13.523902802
1	6.153271482	3.735700847	14.031137783
1	7.563541751	1.170172308	15.591332879
1	6.262330626	0.621468927	17.991224929
1	4.079146566	2.221990225	18.923529778
1	3.902352384	0.508238153	18.531512289
1	1.966286527	1.172717362	16.973444979
1	1.730127141	1.288454818	18.734298229
1	0.902908578	3.380035395	19.660139470
1	0.401256102	5.895905060	18.559696558
1	2.392408890	6.515846781	15.771848044
1	0.691790533	6.829537756	16.268187841
1	-0.710957769	6.869497696	14.222645265
1	-1.133433037	5.006833291	12.187514072
1	-0.292403924	2.751100209	11.809509873
1	0.754848286	2.014254557	13.049902222
1	1.551218072	2.018543419	10.613964586
1	1.768931972	3.765412364	10.755353594
6	4.752966954	-1.238196409	14.917462806
6	5.250481433	-1.101955128	13.503920171
6	3.475444007	-0.984496363	15.265949636
6	2.468146228	-0.527002383	14.306096985
6	2.878940170	-0.567391135	12.901461060
6	4.148008322	-0.816838712	12.520238479
1	1.453887081	-0.884588348	14.498635808
1	2.199520235	0.745751820	14.601681096
1	5.801284453	-2.011869256	13.205022725
1	6.016985460	-0.305651785	13.451064533
1	3.169769259	-1.104804950	16.306240664
1	5.472148357	-1.551510277	15.673559980
1	4.409326396	-0.811806288	11.462362241
1	2.119881887	-0.368965223	12.143554525
6	4.510023299	6.378423018	13.562867157
6	4.026085458	5.766111127	12.246123226
1	4.626255345	4.899869653	11.955433753
1	2.978838103	5.458083269	12.312637920
1	4.108155161	6.512708040	11.442604277
6	3.709501487	7.660458521	13.838724188
1	3.915236181	8.407552403	13.057590915
1	2.630999883	7.468979505	13.829685413
1	3.979345420	8.100055553	14.806810339
6	6.000646580	6.734476307	13.443914174
1	6.378219052	7.174620253	14.375393658
1	6.603276819	5.846350839	13.218025644
1	6.153228175	7.465403030	12.635577193

^{3}Int$_\pi$ (5a)

26	4.200165666	3.413741372	15.079295399
8	4.424024940	1.577521373	15.385667115
1	5.358547157	1.385813237	15.521330018
6	6.633234634	-0.892618818	15.089928215
6	2.755273255	2.962704052	13.809163592
6	2.684475004	3.416843752	16.376218719
6	5.631773312	3.864982152	16.491701623
6	5.681762651	3.477119955	13.655518636
6	1.525726995	1.931174749	12.208040785
7	2.769713761	2.043572468	12.819540565
7	1.473722284	3.406953512	13.831649682
6	0.697705222	2.793633816	12.857500339
6	0.993690313	4.327540546	14.844468814
7	1.415691358	3.841932879	16.143289034
7	2.621613258	2.848900897	17.600831934
6	1.340837614	2.928717008	18.136780044
6	0.571132266	3.553326835	17.206349031
6	3.725932308	2.145886399	18.239826432
6	4.795212555	3.068055038	18.783794543
7	5.773209310	3.582734984	17.818279537
7	6.808633237	4.470739848	16.178193919
6	7.667224407	4.566129617	17.260210619
6	7.008479418	4.001251750	18.301648793
6	7.064356090	5.108703924	14.910901889
7	6.815938641	4.213706369	13.805316924
7	5.849668709	2.898003584	12.432322932
6	7.044311835	3.290066764	11.839135206
6	7.659863489	4.125337275	12.710828546
6	4.971304779	1.999358873	11.674264078
6	3.934529738	1.239808035	12.473064235
1	7.349583309	2.926823126	10.866525234
1	8.606071408	4.648405380	12.660429813
1	8.109550556	5.420289180	14.878480106
1	6.400813016	5.975527127	14.822306279
1	8.649728667	5.014429269	17.188001119
1	7.301070925	3.855806061	19.333532883
1	4.328122363	3.920857592	19.293268421
1	5.367370932	2.513001379	19.533987341
1	4.144296783	1.445148287	17.511482294
1	3.307500638	1.586239822	19.080690430
1	1.098058533	2.532973798	19.114624592
1	-0.479829457	3.812693969	17.201919137
1	1.429069378	5.317493683	14.675437202
1	-0.096144453	4.365981711	14.810045545
1	-0.352387411	3.019053680	12.721885549
1	1.343217625	1.248532020	11.387708343
1	3.584825485	0.404685452	11.859865870
1	4.363117993	0.853400552	13.402586997
1	5.625124218	1.264523090	11.193028718
1	4.485308354	2.576883443	10.876952613
6	7.822366356	0.892577510	16.236716764

6	8.494967785	1.329914974	14.968476048
6	6.966561337	-0.178193042	16.268016489
6	7.163199886	-0.478700712	13.844213316
6	8.036109157	0.574075011	13.755851347
1	5.944945794	-1.726284751	15.138777505
1	8.359960388	2.413985808	14.813729797
1	9.595735196	1.233155242	15.076971061
1	8.432321988	0.880443471	12.789056826
1	6.863651285	-1.007459988	12.939103313
1	6.511674659	-0.476010761	17.212914348
1	8.048735250	1.443671526	17.148677926
6	3.782922420	6.565113755	13.103143060
6	5.174922822	7.020940692	12.642572815
1	5.840897569	6.168327255	12.478611927
1	5.096651024	7.565343280	11.689487349
1	5.638375611	7.690850403	13.376820254
6	3.209792020	5.598443493	12.062670377
1	3.207557790	6.083838173	11.075710262
1	3.814194283	4.690715871	11.989447528
6	2.865046966	7.792620057	13.219106911
1	2.793436265	8.305890182	12.248424677
1	1.853064640	7.502768950	13.527516849
1	3.253920529	8.509143526	13.953441466
1	2.181024333	5.309366774	12.293601496
16	3.873722574	5.832140432	14.829476602
